# Global, Regional, and National Burden of Cardiovascular Diseases Associated with Particulate Matter Pollution: A Systematic Analysis of Deaths and Disability-Adjusted Life Years with Projections to 2030

**DOI:** 10.31083/RCM27056

**Published:** 2025-04-17

**Authors:** Yi He, Qiongyue Zhang, Ting Zhou, Ying Lan

**Affiliations:** ^1^Department of Cardiovascular Medicine, Center for Circadian Metabolism and Cardiovascular Disease, Southwest Hospital, Third Military Medical University (Army Medical University), 400038 Chongqing, China; ^2^Key Laboratory of Geriatric Cardiovascular and Cerebrovascular Disease, Ministry of Education of China, 400038 Chongqing, China; ^3^Department of Nephrology, Daping Hospital, Army Medical Center, Third Military Medical University (Army Medical University), 400042 Chongqing, China; ^4^Department of Critical Care Medicine, Affiliated Hospital of Chengdu University, 610081 Chengdu, Sichuan, China

**Keywords:** cardiovascular diseases, fine particulate matter, Global Burden of Disease, ischemic heart disease, stroke

## Abstract

**Background:**

This research assesses how fine particulate matter (PM2.5) pollution influences cardiovascular diseases (CVDs) globally.

**Methods:**

Utilizing data from the 2021 Global Burden of Disease (GBD) study, we assessed the impact of PM2.5 pollution on CVDs in individuals aged 25 and older. The health burden was quantified using measures such as disability-adjusted life years (DALYs), age-standardized rates (ASRs), and the effective annual percentage change (EAPC). Joinpoint regression models were used to describe the temporal trends of CVD burdens, while the Bayesian age–period–cohort (BAPC) models were employed to project the CVD burdens through 2030. Frontier analysis was conducted to identify potential areas for improvement and gaps between the development statuses of different countries. Decomposition analysis was applied to assess the impact of population growth, aging, and epidemiological changes on the burden of CVDs.

**Results:**

Despite a decline in ASRs for both sexes, males continued to bear a disproportionate burden of CVDs. While substantial reductions in ASRs have been noted in Western Europe and High-income North America, smaller decreases in the EAPC have been seen in South Asia, Oceania, and Western Sub-Saharan Africa; however, Oceania faces the highest mortality burden. An inverse relationship between the sociodemographic index (SDI) and ASRs is evident nationally. Meanwhile, Afghanistan and Egypt reported elevated ASRs, and Iceland recorded the lowest rate. Projections suggest a potential reversal in ASRs by 2021. A decomposition analysis revealed that intracerebral hemorrhage poses the greatest burden in middle SDI regions, while ischemic heart disease is notably burdensome in high SDI and high-middle SDI regions.

**Conclusions:**

This study highlights the disproportionate burden of CVDs associated with PM2.5 pollution, particularly in males and lower SDI regions, with significant regional disparities and projections indicating potential reversals in trends.

## 1. Introduction

The 2021 Global Burden of Disease (GBD) report revealed that fine particulate matter (PM2.5) pollution stands as one of the most significant global health threats, contributing to 8.0% of the total disability-adjusted life years (DALYs) worldwide. In 2021, PM2.5 pollution was categorized as a Level 3 contributor to the burden, with two primary forms: household air pollution and ambient particulate matter pollution. Moreover, while global summary exposure values and risk-attributable DALYs for household air pollution were shown to decline significantly, those for ambient particulate matter and ozone pollution increased [1]. This alarming situation remains largely driven by rapid industrialization, uncontrolled urbanization, and a heavy reliance on fossil fuels, all of which exacerbate air pollution and elevate the incidence of respiratory and cardiovascular diseases (CVDs) [2].

A nationwide cohort study in Denmark demonstrated that PM2.5 pollution, environmental noise exposure, and limited green space independently increased the risk of myocardial infarction, with PM2.5 showing the strongest association [3]. A prior analysis based on data from the 2019 GBD study also examined the spatiotemporal distribution of the ischemic heart disease burden attributable to ambient PM2.5 pollution. The finding revealed significant regional heterogeneity in burden trends: low–middle sociodemographic index (SDI) regions experienced the greatest increases, with an effective annual percentage change (EAPC) in age-standardized mortality rates (ASMRs) of 3.73 (95% CI: 3.56–3.90) and age-standardized disability rates (ASDRs) of 3.83 (95% CI: 3.64–4.02). Furthermore, the burden was notably higher among males and older populations [4].

Although previous studies have highlighted the significant health risks associated with CVDs, research on the long-term trends on the burden of CVDs related to PM2.5, as well as its age- and sex-specific variations, remains limited [5, 6]. Our study utilizes data from the updated 2021 GBD report to systematically analyze the burden of CVDs associated with PM2.5 exposure from 1990 to 2021. Particular attention is given to key factors, such as age and sex, to provide a comprehensive assessment of the impact of PM2.5 on DALYs and deaths at global, regional, and national levels.

## 2. Methods

### 2.1 Overview and Data Sources

The 2021 GBD study is the largest and most comprehensive observational epidemiological survey to date. It thoroughly assesses health losses across 204 countries and territories from 1990 to 2021, covering 371 diseases and injuries alongside 88 risk factors. The original data and methodologies of the GBD study have been detailed in previous publications [7]. Data on the burden of CVDs related to PM2.5 exposure were obtained using the results tool provided on the Institute for Health Metrics and Evaluation (IHME) website.

### 2.2 Definition

In the 2021 GBD study, PM2.5 is defined as the annual average mass concentration of particulate matter with an aerodynamic diameter of less than 2.5 micrometers per cubic meter of air, weighted by population. CVDs related to PM2.5 include ischemic heart disease and stroke. Ischemic heart disease encompasses acute myocardial infarction, chronic stable angina, chronic ischemic heart disease, and heart failure attributable to ischemic heart disease. Stroke was defined based on World Health Organization (WHO) criteria and was further categorized into three subtypes for separate estimation: (1) ischemic stroke, (2) intracerebral hemorrhage, and (3) subarachnoid hemorrhage [8, 9]. DALYs incorporate both years of potential life lost due to premature mortality and years of healthy life lost due to disability. Uncertainty intervals (UIs) are calculated by drawing 1000 samples from the posterior distribution of the model, with 95% UIs defined as the 2.5th and 97.5th percentiles of the distribution [10]. Additionally, based on SDI, the 204 countries and territories worldwide are further classified into five levels.

### 2.3 Statistical Analysis

In view of previous literature, our study included individuals aged 25 and older [11]. We employed rigorous statistical methods to monitor the burden and trends in CVDs associated with PM2.5 exposure.

The EAPC is a commonly used metric to summarize the trends in age-standardized rates (ASRs) over a specific period [12]. Our analysis utilized a joinpoint regression model to identify significant trends over time [13]. Initially introduced by Kim *et al*. in 2000 [14], the joinpoint regression model is designed to analyze the temporal patterns of disease distribution by constructing a segmented regression framework [15]. This model optimizes trend data for each segment, enabling a comprehensive examination of disease variations over time and providing valuable insights at a global level. The results of the model are typically summarized using two key metrics: annual percentage change (APC), which evaluates trends within individual segments, and average annual percentage change (AAPC), which captures the overall trend across the entire study period [16].

We also employed frontier analysis to assess the burden of CVDs associated with PM2.5 exposure and compared the performance of countries and territories with the lowest achievable burden. This method constructs a non-linear frontier that reflects the minimal possible burden based on the SDI. Non-parametric data envelope analysis was applied, following the methodology outlined in prior research [17]. The gap between the ASRs in a country and its frontier highlights the potential health improvements that could be realized given the current level of development in that country.

Given the various subtypes of CVDs, we also employed decomposition analysis to examine the contribution of each factor to the overall burden of CVDs across different SDI regions. Decomposition analysis is a method that disentangles the effects of various factors on overall variation, thereby revealing significant heterogeneity in demographic and epidemiological trends. We applied the Das Gupta decomposition method, which differentiates the impacts of changes in age structure, population size, and epidemiological trends [18].

We then employed the Bayesian age–period–cohort (BAPC) models to project trends in the burden of CVDs associated with PM2.5 exposure through 2030 [19]. These models utilize the integrated nested Laplace approximations (INLAs) to derive marginal posterior distributions, effectively overcoming challenges related to mixing and convergence commonly encountered with traditional Bayesian methods, such as Markov chain Monte Carlo sampling [20]. The analysis used the BAPC and INLAs packages in R statistical software (version 4.3.2, R Foundation for Statistical Computing, Vienna, Austria).

## 3. Results

### 3.1 Global Burden and Trends

Between 1990 and 2021, the ASRs of CVDs associated with PM2.5 pollution decreased, and the total number of DALYs and deaths increased, reaching 99.6378 million and 4.4825 million, respectively. When accounting for global population growth, the ASDR has shown significant declines. The ASMR also decreased substantially, reaching 97.1 (95% UI: 77.4–117.1) per 100,000 with an EAPC of –1.92 (95% CI: –2.06 to –1.79). Among these, deaths related to ischemic heart disease reached 905,600 (95% UI: 692,500–1,145,400), with DALYs totaling 54.6757 million (95% UI: 41.3847 million–67.9976 million). Stroke-related deaths numbered 1.9897 million (95% UI: 1.5279 million–2.4966 million), and DALYs amounted to 42.3041 million (95% UI: 34.5101 million–50.5094 million). Among stroke subtypes, subarachnoid hemorrhage showed the most significant decline in both ASRs. The EAPC for ASDR was –4.2 (95% CI: –4.42 to –3.98), and for ASMR, the EAPC was –4.64 (95% CI: –4.91 to –4.36) (**Supplementary Tables 1,2**). Among the GBD regions, Oceania exhibited the highest ASRs for CVDs associated with PM2.5 exposure. High-income North America had the lowest ASMR at 8.1 (95% UI: 3.8–13.2) per 100,000, while Australasia recorded the lowest ASDR at 397.0 (95% UI: 13.7–1088.6) per 100,000 (**Supplementary Tables 1,2**).

### 3.2 Temporal Trends

**Fig. 1.**
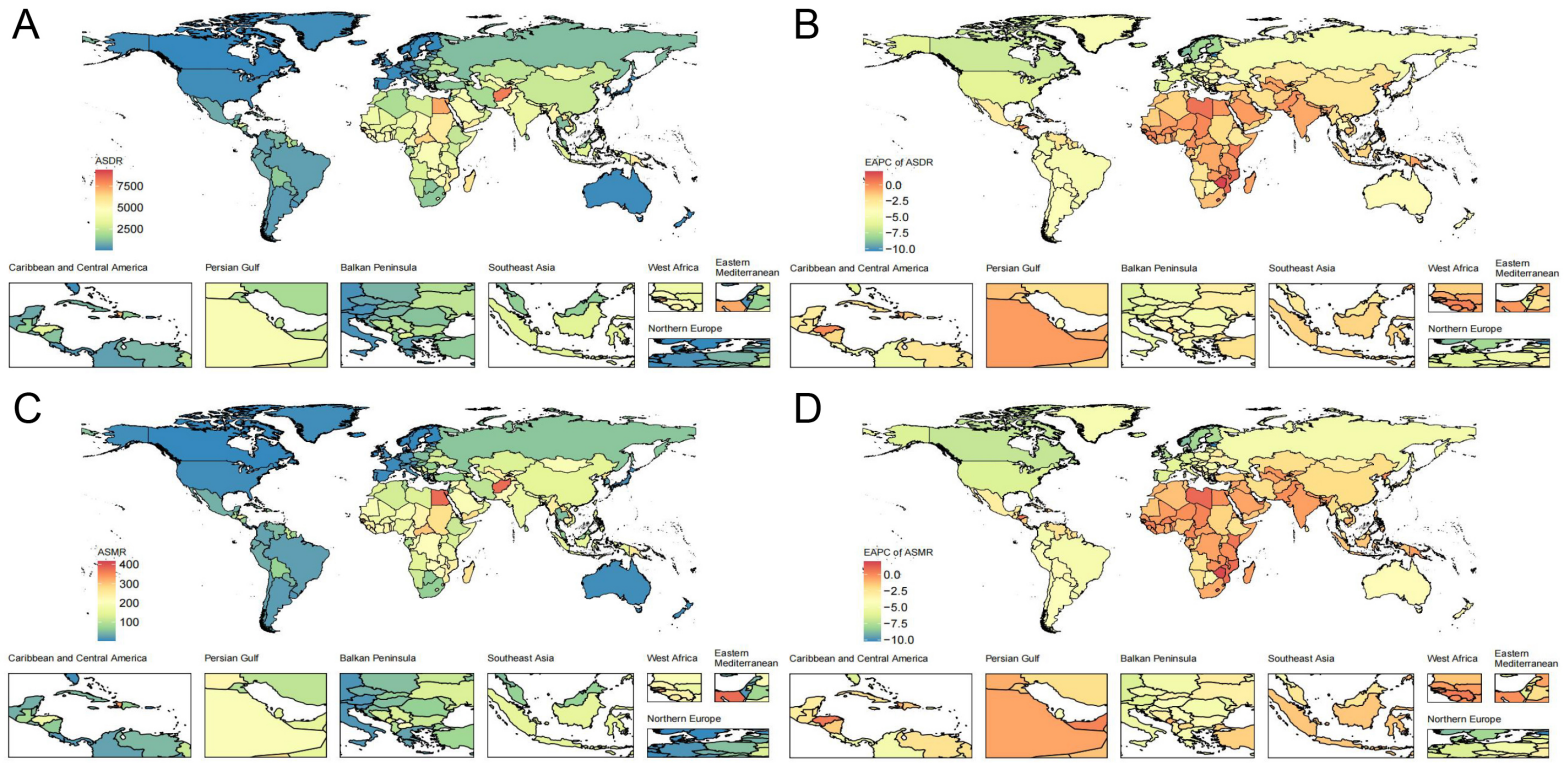
**The global CVDs burden associated with PM2.5 pollution for both sexes in 204 countries and territories**. (A) Global distribution of ASDRs for CVDs associated with PM2.5 pollution in 2021. (B) Global distribution of EAPCs of ASDRs for CVDs related to PM2.5 pollution from 1990 to 2021. (C) Global distribution of ASMRs for CVDs pertaining to PM2.5 pollution in 2021. (D) Global distribution of EAPCs of ASMRs for CVDs associated with PM2.5 pollution from 1990 to 2021. ASDR, age-standardized disability rate; CVDs, cardiovascular diseases; PM2.5, fine particulate matter; EAPC, effective annual percentage change; ASMR, age-standardized mortality rate.

**Fig. 2.**
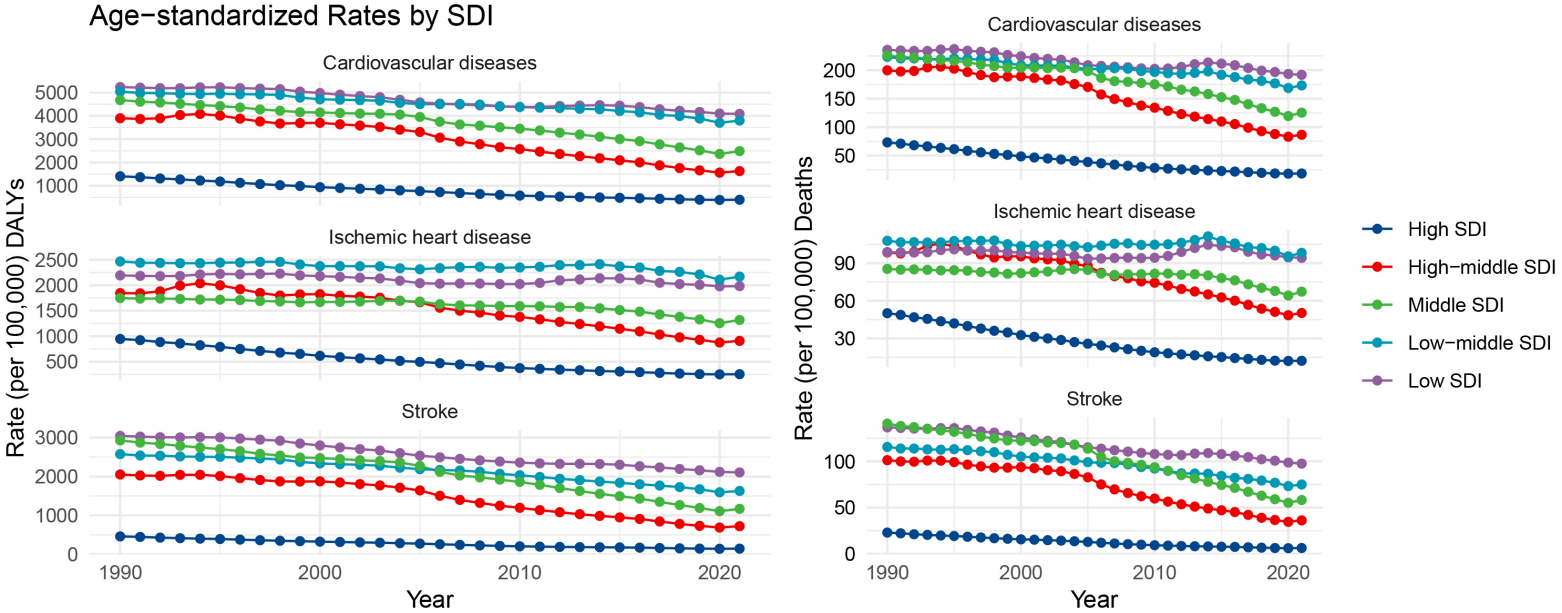
**Temporal trends of ASDRs and ASMRs associated with PM2.5 pollution for CVDs, ischemic heart disease, and stroke by SDI regions from 1990 to 2021**. SDI, sociodemographic index; ASDRs, age-standardized disability rates; CVDs, cardiovascular diseases; PM2.5, fine particulate matter; ASMRs, age-standardized mortality rates; DALYs, disability-adjusted life years.

**Fig. 3.**
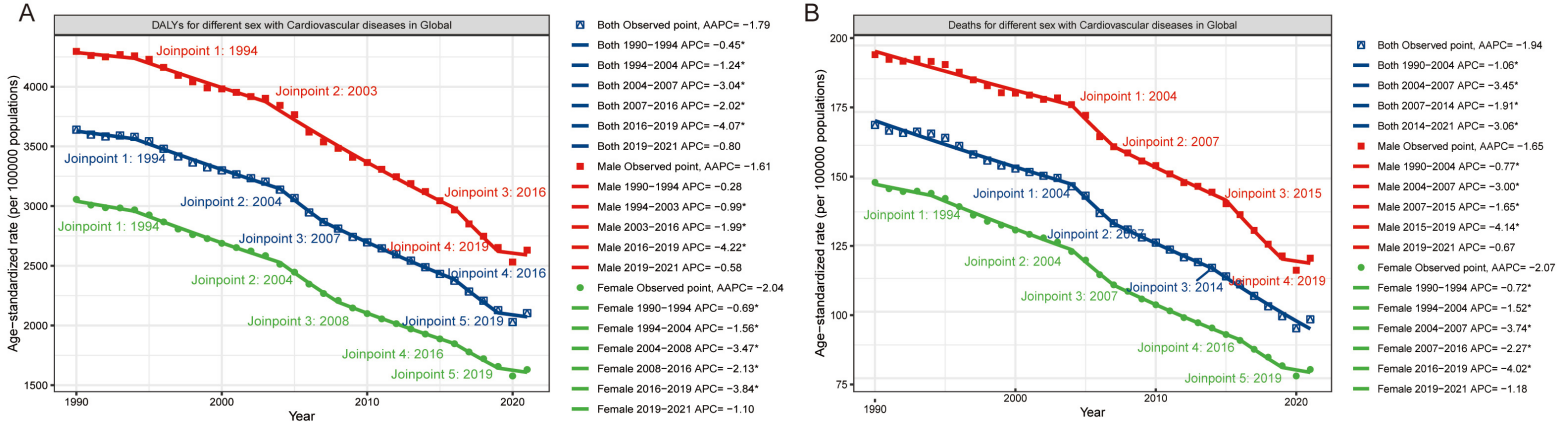
**Temporal trends of (A) ASDRs and (B) ASMRs associated with PM2.5 pollution for CVDs by gender from 1990 to 2021 using the joinpoint regression model**. ASDRs, age-standardized disability rates; CVDs, cardiovascular diseases; PM2.5, fine particulate matter; ASMRs, age-standardized mortality rates; DALYs, disability-adjusted life years; APC, annual percentage change; AAPC, average annual percentage change. * indicates that the annual percent change (APC) is significantly different from zero at the alpha = 0.05 level.

Over the past three decades, CVDs associated with PM2.5 pollution have emerged as a significant global health challenge. Data analyses revealed that deaths and DALYs due to CVDs have surged by 34.8% and 26.3%, respectively (**Supplementary Tables 1,2** and Fig. 1). Notably, from 1990 to 2021, the ASRs for ischemic heart disease in low–middle SDI regions were higher than those in low SDI regions. High–middle SDI regions initially also had relatively high ASRs yet showed the most significant improvements over time. Comparatively, middle SDI regions initially had higher ASRs for stroke, but these decreased substantially, with low SDI regions now bearing the heaviest stroke burden (Fig. 2). Furthermore, the declines in ASRs were not uniform across all periods, with certain years exhibiting more pronounced reductions. The most significant decrease in the ASDR occurred between 2016 and 2019 (APC = –4.07%), while the largest drop in the ASMR occurred between 2004 and 2007 (Fig. 3).

**Fig. 4.**
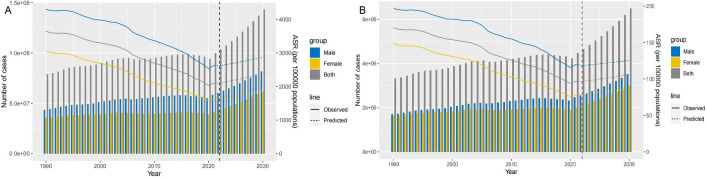
**Projects the ASRs and numbers of CVDs by gender from 1990 to 2030 based on the BAPC model**. (A) ASDRs. (B) ASMRs. ASRs, age-standardized rates; BAPC, Bayesian age–period–cohort; CVDs, cardiovascular diseases; ASMRs, age-standardized mortality rates; ASDRs, age-standardized disability rates.

The projections from 1990 to 2030 indicate that CVDs associated with PM2.5 pollution will continue to pose a significant threat to global health, with both DALYs and deaths expected to rise steadily. Furthermore, these projections highlight a gender disparity; the CVDs burden in absolute numbers is consistently lower for females compared to males (Fig. 4 and **Supplementary Fig. 1**).

### 3.3 Regional Burden and Trends of CVDs Associated with PM2.5

Among the 21 GBD regions, Western Europe exhibited the most significant decline in ASDRs, with an EAPC of –6.25 (95% CI: –6.44 to –6.05), closely followed by High-income North America. Surprisingly, Tropical Latin America also showed a notable decrease in ASDR, with an EAPC of –4.74 (95% CI: –4.89 to –4.59). Meanwhile, the declines in ASDRs in Southern Sub-Saharan Africa, South Asia, and Oceania were less pronounced. Regarding mortality rates, High-income North America experienced the steepest decline in EAPC at –6.41 (95% CI: –6.82 to –5.99) and reported the lowest ASMR at 8.1 (95% UI: 3.8–13.2) per 100,000. Oceania bore the heaviest mortality burden, with an ASMR of 232.1 (95% UI: 172–299.8) per 100,000 (**Supplementary Tables 1,2** and Fig. 1).

Statistical data from 1990 to 2021 indicate that as the SDI of countries gradually improved, the ASRs of CVDs associated with PM2.5 exposure declined. However, considerable disparities remain between countries. For instance, the ASRs in countries such as the Solomon Islands, Vanuatu, Afghanistan, and Egypt are higher than expected. In contrast, the ASRs in Finland, New Zealand, Canada, Iceland, and Ireland closely align with expected values (**Supplementary Figs. 2,3**).

At the national level, the burden of CVDs measured by DALYs increased significantly in Lesotho, with an EAPC of 2.13 (95% CI: 1.58–2.69), while Estonia experienced the largest reduction in both DALYs (–10.32, 95% CI: –10.96 to –9.68) and deaths (–10.18, 95% CI: –10.83 to –9.53). High SDI countries such as the Maldives, Norway, and Sweden also showed notable reductions. In the case of a decline in EAPC in most countries, Lesotho (EAPC = 2.01, 95% CI: 1.47–2.55) and Zimbabwe (EAPC = 1.74, 95% CI: 1.19–2.28) experienced slight increases in their mortality burden (**Supplementary Tables 3,4**). In 2021, China and India had the highest DALYs and death burden due to CVDs associated with PM2.5 exposure, far surpassing other countries. The Solomon Islands exhibited the highest ASDR (9406.8, 95% UI: 7106.3–12,313.7) per 100,000, followed by Vanuatu, while Iceland had the lowest (53.2, 95% UI: 9.7–117.4) per 100,000 (**Supplementary Table 3**). Indeed, Vanuatu recorded the highest ASMR (391.8, 95% UI: 303.4–481.7) per 100,000, while Iceland again had the lowest (3.0, 95% UI: 0.5–6.8) per 100,000 (**Supplementary Table 4**).

### 3.4 Sex-Specific Burden of CVDs and Trends Associated with PM2.5

The ASDRs for CVDs associated with PM2.5 exposure decreased significantly in both sexes from 1990 to 2021. In males, the ASDR dropped from 4296.3 (95% UI: 3601.8–5021) per 100,000 in 1990 to 2631 (95% UI: 2110–3182.2) per 100,000 in 2021, while in females, the ASDR decreased over the same period from 3056.3 (95% UI: 2528.1–3627.9) per 100,000 to 1631.1 (95% UI: 1315.8–1956.4) per 100,000. However, while ASMR decreased for both sexes, the burden of CVDs remained higher in males than in females (**Supplementary Tables 5,6**).

Joinpoint regression models revealed that the most significant declines in ASRs for both sexes occurred between 2016 and 2019 (Fig. 3). Regarding the CVD subtypes, the greatest decline in the ASRs was observed for subarachnoid hemorrhage, which currently has the lowest burden among the CVD subtypes (**Supplementary Tables 1,2**). Decomposition analysis revealed that the high burden observed in the middle SDI regions was primarily due to a higher proportion of intracerebral hemorrhage in both sexes, while in the high–middle SDI and high SDI regions, ischemic heart disease was the predominant cause (**Supplementary Fig. 4**).

Gender differences in global ASRs for CVDs associated with PM2.5 pollution in 2021 are illustrated in **Supplementary Fig. 1**. Afghanistan bore the heaviest burden of ASRs for males, while Egypt had the highest ASR burden for females. Regionally, all five SDI territories show a downward trend in ASR burden for CVDs. Meanwhile, although the low SDI region exhibited the largest EAPC decrease for males, it remained the region with the highest burden. For females, the high SDI region showed the most significant EAPC decrease, while the EAPC decreased the least in areas with low SDI and had the heaviest disease burden (**Supplementary Tables 5,6**).

### 3.5 Age-Specific CVDs Burden and Trends Associated with PM2.5

Historical data from 1990 to 2010 showed a cumulative rise in DALYs and deaths across all age subgroups. However, from 2010 to 2020, this trend gradually decreased, although a potential rebound was observed in 2021. The older male population, particularly those aged 70 and above, accounted for most DALYs and deaths (**Supplementary Fig. 5**; **Supplementary Tables 7,8**). In high SDI regions, DALYs and mortality rates rose significantly with age but demonstrated an overall declining trend in recent years, with the most notable decrease in older people. High–middle SDI regions also showed an upward trend in DALYs and mortality with age, and the increase was slightly more pronounced than in regions with a high SDI. In middle SDI regions, the burden of CVDs rose substantially with age, particularly among individuals aged 60 and above. Compared to high and high–middle SDI regions, the decline in DALYs and mortality among older adults in middle SDI regions was less prominent. Low–middle and low SDI regions exhibited severe upward trends in CVDs burden, with low SDI regions experiencing the highest DALYs and mortality rates among those aged 85 and above males across all SDI groups (**Supplementary Fig. 6**; **Supplementary Tables 9,10**).

### 3.6 Frontier Analysis

**Fig. 5.**
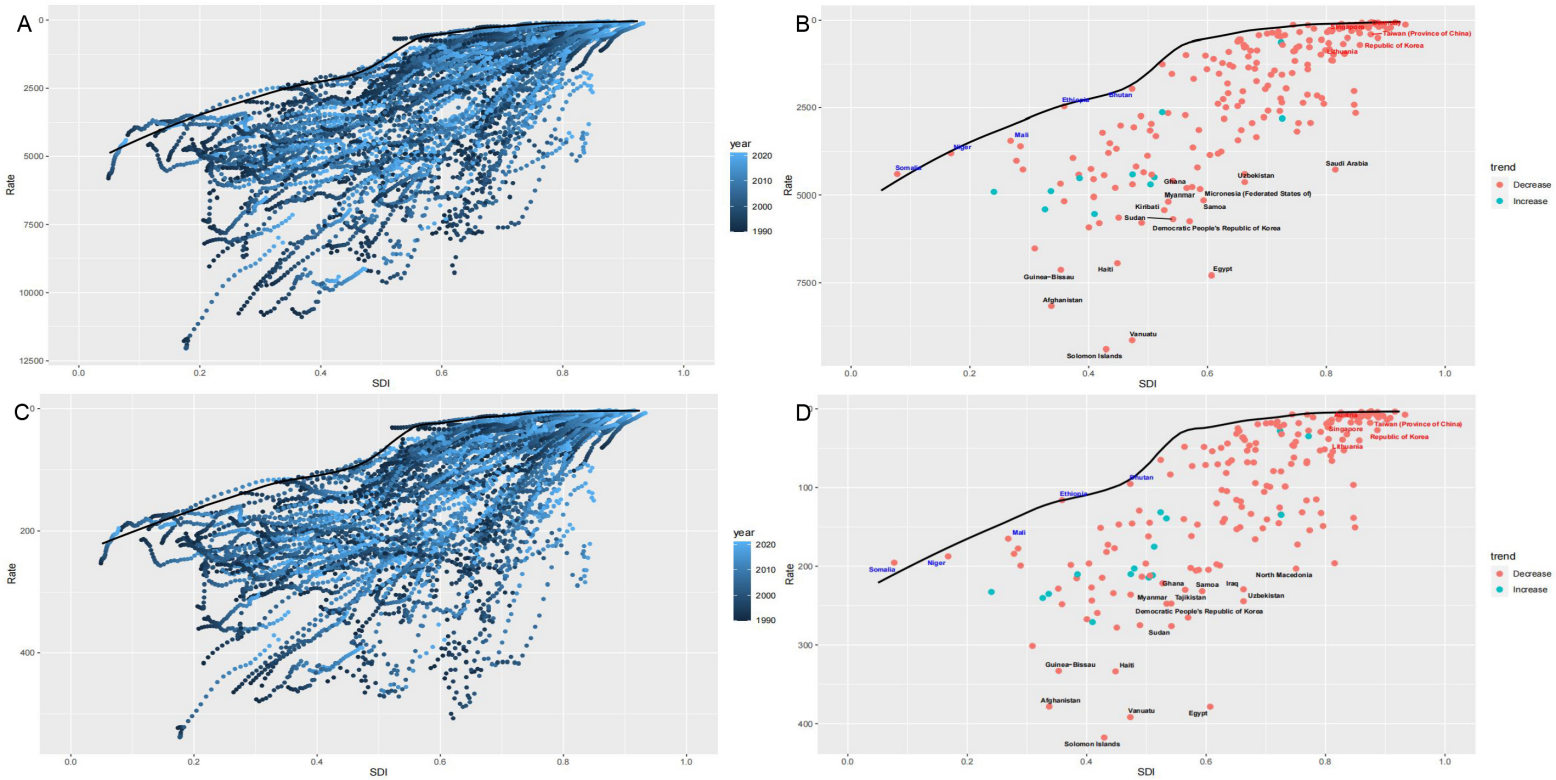
**The frontier analysis, denoted by the solid black lines, examines the relationship between the SDI and ASRs for DALYs (A,B) and deaths (C,D) from CVDs associated with PM2.5 exposure**. In graphs (A) and (C), the color gradient indicates the temporal progression, with the darkest shades representing the year 1990 and the lightest shades corresponding to 2021. In graphs (B) and (D), each point represents a specific country or territory in 2021, with the 15 countries exhibiting the greatest deviation from the frontier marked in black. Countries with low SDIs and minimal deviation from the frontier are highlighted in blue, while those with high SDIs but substantial deviation relative to their development level are emphasized in red. DALYs, disability-adjusted life years; SDI, sociodemographic index; CVDs, cardiovascular diseases; ASRs, age-standardized rates; PM2.5, fine particulate matter.

The frontier analysis results up to 2021 highlighted significant disparities between different countries and territories, whereby the Solomon Islands, Vanuatu, Afghanistan, and Egypt exhibited notably higher ASRs of related CVD burdens. Countries with lower SDIs, including Somalia, Niger, Mali, and Ethiopia, appear closer to their respective frontiers. Conversely, countries and territories with higher SDI values, such as Singapore, Taiwan (China), and the Republic of Korea, are positioned further from the ASR frontiers (Fig. 5).

## 4. Discussion

This study comprehensively analyzed the global burden of CVDs and their subtypes associated with PM2.5 exposure. Our analysis of 204 countries and territories indicates that as the SDI increases, the ASRs for CVDs associated with PM2.5 tend to decline, highlighting the critical role of economic and social development in mitigating the health impacts of environmental pollutants such as PM2.5. In Sub-Saharan Africa and South Asia, although the EAPC showed a downward trend, this decline contrasts sharply with the significant reductions observed in Western Europe. This disparity can be partly attributed to differences in environmental factors, particularly in Sub-Saharan Africa and South Asia, where deforestation and burning of natural vegetation for agricultural land are more common. These activities exacerbate air pollution, negatively impacting cardiovascular health [21]. Moreover, data from the WHO showed significant differences in the allocation of healthcare resources: in certain low-SDI and middle-SDI regions, up to 21% of areas still lack critical medical equipment such as computed tomography (CT) scanners [22]. Interestingly, countries such as Somalia, Niger, and Mali, despite facing economic challenges, appear to be more effective in managing the impact of environmental pollutants on cardiovascular health. Research has shown that various intervention strategies at the household and community levels, including indoor smoking bans, air purification measures, and the use of improved stoves, can effectively reduce exposure to indoor air pollutants [23]. Alternatively, China bears the heaviest disease burden, driven by factors such as increased population density, industrialization, and urbanization, which collectively exacerbate the burden of CVDs. In densely populated areas, the high energy demand from residents and significant vehicle emissions contribute to the deterioration of outdoor air quality [24]. Additionally, urbanization has led to the conversion of natural land for transportation and industrial, reducing the ecosystem’s capacity to absorb and purify particulate matter [25]. However, the ASRs for CVDs associated with PM2.5 have also declined in China, likely due to policy interventions and increased public awareness, particularly following the updated national ambient air quality standards in 2012, which enhanced controls on PM2.5 pollution [26].

In Oceania, Vanuatu recorded the highest ASMRs for CVDs, and the Solomon Islands had the highest ASDR. Oceania faces the heaviest burden of mortality, which has likely been exacerbated by the severe impact of wildfires in recent years. These wildfires have significantly contributed to environmental air pollution. Indeed, studies by Williams VA highlight the direct correlation between inhaling wildfire smoke and an increased incidence of CVDs [27]. Additionally, a population-based time-series study conducted in Perth by Adeleh Shirangi further confirmed these effects, showing that increased PM2.5 concentrations significantly raise the risk of cardiovascular hospitalizations and arrhythmias [28]. Numerous in vitro studies have demonstrated that PM2.5 can activate pathways that generate reactive oxygen species, affecting vascular inflammation, atherosclerosis, vasomotor balance, coagulation, and platelet activation [29]. Furthermore, animal studies suggest that PM2.5 exposure depletes circulating endothelial progenitor cells, impeding vascular repair processes [30]. Another potential mechanism involves changes in autonomic nervous system balance, with experimental research linking PM2.5 exposure to sympathetic activation and parasympathetic inhibition. Meanwhile, epidemiological studies have confirmed the association between air pollution exposure and changes in heart rate variability [31].

The significant decline in the ASMRs for CVDs in Tropical Latin America is encouraging. This reduction may be attributed to the decrease in smoking rates, which is an important source of PM2.5 exposure. The Framework Convention on Tobacco Control by the WHO has been one of the most notable public health achievements of the past two decades, driving national policies and securing both political and financial support for tobacco control [32]. Despite optimistic projections that countries in the Americas may meet the WHO’s target of reducing tobacco use to below 15% by 2025, tobacco remains a leading cause of disability and mortality in the region [33]. However, effective policy interventions to address this persistent public health issue include increasing taxes on tobacco products, restricting advertising and promotional activities, and mandating the implementation of health warning labels, further disseminating the dangers of smoking through various media channels, such as television, radio, and social media, maybe an impactful strategy for reducing tobacco consumption.

A meta-analysis of 69 studies has already shown the relationship between long-term exposure to PM2.5 and the risk of ischemic heart disease and stroke. Specifically, for every 10 µg/m^3^ increase in PM2.5 concentration, the risk of ischemic heart disease mortality increased by 23%, while the risk of a first-time stroke rose by 13%. Furthermore, the association between long-term PM2.5 exposure and ischemic stroke was found to be stronger compared to hemorrhagic stroke [34]. Socioeconomic development plays a decisive role as a covariate in the risk factors associated with the occurrence of ischemic heart disease and stroke [35]. Decomposition analysis within our study also suggests that a larger proportion of the ischemic heart disease burden is associated with PM2.5 exposure in regions with high SDIs and high–middle SDIs. However, in areas with a middle SDI, hemorrhagic stroke accounts for a higher proportion of stroke-related mortality and DALYs compared to ischemic stroke. Several factors may explain this: In middle SDI regions, gaps in public health interventions, lifestyle factors such as high salt intake, smoking, and alcohol consumption, and inadequate management of hypertension could contribute to an elevated risk of hemorrhagic stroke. Additionally, data collection and reporting biases, as well as differences in patient age distribution, may also contribute to the higher prevalence of hemorrhagic stroke in these regions.

From 1990 to 2021, the ASRs for CVDs associated with PM2.5 exposure showed an overall declining trend in both sexes, with females experiencing a more pronounced decrease in the EAPC. These gender differences may be attributed to factors such as genetic and hormonal variations in vascular hemodynamics, as well as differences in behavioral risk factors such as smoking habits and stress levels [36, 37]. The significant decline in EAPC among females was particularly prominent in regions with high SDI, suggesting that lower SDI regions should prioritize improving female health and reducing exposure to environmental pollutants.

Our study found that older populations bear a heavier burden of CVDs associated with exposure to PM2.5. This may be attributed to the higher likelihood of pre-existing comorbidities in older individuals. Hypertension is a known risk factor for subarachnoid hemorrhage, and studies suggest that PM2.5 can trigger a series of pathophysiological responses that contribute to hypertension [38]. Previous research has also reported that for every 10 µg/m^3^ increase in PM2.5 exposure, the adjusted risk ratio for developing hypertension increases by 1.13, along with an observed increase in the burden of ischemic stroke [39]. In addition, pre-existing chronic conditions such as dyslipidemia and diabetes exacerbate the risks associated with PM2.5 exposure by amplifying systemic inflammation. Another reason for the increased burden of PM2.5-related diseases among older adults is their lower health awareness and reduced likelihood of adopting preventive measures [40]. With the WHO projecting that the global population aged 60 and older will double by 2050, rapid demographic changes, driven by socioeconomic development, have led to a higher proportion of the population at greater risk for PM2.5-related health issues [15, 41].

Undoubtedly, our study has inherent limitations. First, our analysis is based on secondary data from the GBD study, which inevitably introduces potential concerns regarding data quality and accuracy. Second, PM2.5 is a mixture of multiple components with varying physical characteristics. A study suggest that, even under identical environmental conditions and concentrations, the effects of different PM2.5 components from various sources may differ regarding their impact on disease risk [42]. Third, the GBD 2021 study provides data on the overall burden of CVDs, particularly focusing on two subtypes: ischemic heart disease and stroke. Fourth, while the predictive models in our study broadly describe future trends, they do not provide precise forecasts. The BAPC and joinpoint regression models rely on assumptions that may oversimplify the trends in CVDs. Specifically, these models assume that the effects of age, periods, and cohorts on CVDs, DALYs and mortality are additive and independent, which may not fully capture the potential interactions between these factors. Moreover, these models assume that changes in CVDs trends are smooth and continuous, potentially overlooking abrupt shifts caused by external factors such as policy changes, environmental influences, or medical advancements.

## 5. Conclusions

Our study underscores that substantial regional disparities remain despite global advances in mitigating the burden of CVDs associated with PM2.5 exposure. Moreover, there is potential for a rebound post-2021, with significant variations observed across different age groups, sexes, and SDI regions. Given the anticipated increase in global PM2.5 exposure levels, it is imperative to implement sustainable and environmentally conscious strategies to address this emerging challenge.

## Availability of Data and Materials

The datasets generated and/or analysed during the current study are available in the Global Health Data Exchange (GHDx) at https://vizhub.healthdata.org/gbd-results.

## Supplementary Material

Supplementary material associated with this article can be found, in the online version, at https://doi.org/10.31083/RCM27056.
