# Supplementary material for: Global, Regional, and National Burden of Cardiovascular Diseases Associated with Particulate Matter Pollution: A Systematic Analysis of Deaths and Disability-Adjusted Life Years with Projections to 2030
Source: Rev Cardiovasc Med. 2025 Apr 17;26(4):27056. doi: 10.31083/RCM27056 (PMC12059744; doi:10.31083/RCM27056)
Supplement: Supplementary file 1 [file 2153-8174-26-4-27056-s1.zip › Supplementary Figures.docx]

Supplementary Figure 1: Gender differences in ASRs and numbers for CVDs associated with PM2.5 pollution globally in 2021. (A) DALYs of CVDs (Male). (B) ASDRs of CVDs (Male). (C) Deaths of CVDs (Male). (D) ASMRs of CVDs (Male). (E) DALYs of CVDs (Female). (F) ASDRs of CVDs (Female). (G) Deaths of CVDs (Female). (H) ASMRs of CVDs (Female).

Supplementary Figure 2: (A) Correlation between regions and SDI for ASDRs of CVDs associated with PM2.5 pollution from 1990 to 2021. (B) Correlation between 204 countries and territories and SDI for ASDRs of CVDs associated with PM2.5 pollution from 1990 to 2021.

Supplementary Figure 3: (A) Correlation between regions and SDI for ASMRs of CVDs associated with PM2.5 pollution from 1990 to 2021. (B) Correlation between 204 countries and territories and SDI for ASMRs of CVDs associated with PM2.5 pollution from 1990 to 2021.

Supplementary Figure 4: Decomposition analysis of CVDs associated with PM2.5 pollution: Changes in DALYs and deaths driven by population growth, aging, and epidemiological transitions between 1990 and 2021 at the global level and by SDI quintile. The black dot represents the total change resulting from all components combined. Positive values indicate an increase in CVDs related (A) DALYs or (B) deaths associated with each factor, while negative values reflect a decrease in (A) DALYs or (B) deaths due to the respective determinant.

Supplementary Figure 5: The Burden of CVDs associated with PM2.5 pollution and its trends by age group in global from 1990-2021. (A) Changes in DALYs in each age group (25 to 95+ years, 5 years apart); (B) Changes in deaths in each age group (25 to 95+ years, 5 years apart).

Supplementary Figure 6: The Burden of CVDs associated with PM2.5 pollution and its trends by age group in different SDI regions from 1990-2021. (A, C, E ,G ,I) Trends in DALYs by age group in high to low SDI regions; (B, D, F ,H ,J) Trends in deaths by age group in high to low SDI regions.
