# Supplementary material for: Global, Regional, and National Burden of Cardiovascular Diseases Associated with Particulate Matter Pollution: A Systematic Analysis of Deaths and Disability-Adjusted Life Years with Projections to 2030
Source: Rev Cardiovasc Med. 2025 Apr 17;26(4):27056. doi: 10.31083/RCM27056 (PMC12059744; doi:10.31083/RCM27056)
Supplement: Supplementary file 1 [file 2153-8174-26-4-27056-s1.zip › Supplementary Table(s)-final.docx]

Supplementary Table 1 DALYs of CVDs Attributable to PM2.5 Pollution by GBD Regions and CVD Subtypes, 1990 and 2021

|  | Numbers of DALYs (95%UI)_1990 | Age-standardized rate (95%UI)_1990 | Numbers of DALYs (95%UI)_2021 | Age-standardized rate (95%UI)_2021 | Estimated annual percentage changes (95%CI) |
| --- | --- | --- | --- | --- | --- |
| Cardiovascular diseases | 78872929.2 (66741690-91531379.3) | 3640.1 (3063.5-4240.2) | 99637837.1 (80830863.5-118931693.9) | 2104 (1705.1-2513.5) | -1.9 (-2.02 - -1.77) |
| Ischemic heart disease | 36568811.7 (28209134.6-45598949.1) | 1695.9 (1300.6-2123.8) | 54675670.1 (41384693.5-67997588.5) | 1156.3 (874.3-1438.7) | -2.04 (-2.2 - -1.88) |
| Stroke | 44962167 (34723690.9-55702959.9) | 1944.2 (1581.6-2328.4) | 42304117.5 (34510112.8-50509393.9) | 947.7 (731.6-1174.7) | -2.49 (-2.67 - -2.31) |
| Intracerebral hemorrhage | 24071140.4 (19469343.2-28893157.4) | 1069.3 (864-1285.1) | 24015341.8 (18387234.3-29952177.5) | 501.5 (383.9-625.9) | -2.58 (-2.82 - -2.34) |
| Ischemic stroke | 14141351.8 (11202268.4-17271963.3) | 697.7 (550.8-855.7) | 18295352.1 (14175330.3-22797306.3) | 390.5 (302.3-487.1) | -1.31 (-1.4 - -1.22) |
| Subarachnoid hemorrhage | 4091625.3 (2728757-5460251.1) | 177.2 (117-237.2) | 2651473.1 (1948391-3551332.1) | 55.7 (40.9-74.6) | -4.2 (-4.42 - -3.98) |
| High SDI | 8531364.7 (5882057.4-11659917.1) | 73.1 (49.3-101.3) | 4419388.9 (3234384.6-5743336.2) | 1407 (968.4-1921.6) | -4.29 (-4.38 - -4.21) |
| High-middle SDI | 20436983.6 (15935734.7-24953231.4) | 199.8 (152.4-248.5) | 17518215.6 (13571113-22261183.5) | 3897.5 (3022.1-4779.3) | -3.15 (-3.47 - -2.84) |
| Middle SDI | 25936668 (21948754.8-30349120.7) | 226.2 (189.6-266.3) | 35817251.1 (28294777.6-44222332.5) | 4678 (3947.5-5479.9) | -2.07 (-2.25 - -1.88) |
| Low-middle SDI | 17329837.9 (14769480.2-19990588.4) | 223.4 (189.6-258.6) | 30380359.2 (25117293-35574143.9) | 5042.6 (4295.7-5815.9) | -0.88 (-0.96 - -0.81) |
| Low SDI | 6540402.9 (5521730.3-7727610.6) | 235.6 (197.9-277.1) | 11425503.9 (9476227.5-13368646.1) | 5237 (4421.8-6170.4) | -0.86 (-0.93 - -0.79) |
| Andean Latin America | 312233.7 (244829.7-383535.4) | 128.3 (100.7-157) | 274209.7 (188680.1-383370.5) | 2702.3 (2121.7-3314.2) | -4.3 (-4.54 - -4.06) |
| Australasia | 50811.4 (1748.5-139371.9) | 21.9 (0.8-60.2) | 44476 (25542.4-66354.8) | 397 (13.7-1088.6) | -3.9 (-4.46 - -3.33) |
| Caribbean | 420823.9 (300637.6-575930.5) | 137.1 (94.4-192.2) | 550668.8 (374040.3-752446.1) | 2942.8 (2090.7-4041.2) | -1.39 (-1.6 - -1.17) |
| Central Asia | 1195900 (772618.2-1686054.7) | 238.6 (153.1-335.2) | 1431146.6 (1073615.8-1806375.7) | 4771.2 (3076.9-6726.7) | -1.71 (-2.1 - -1.32) |
| Central Europe | 3572446 (2311884.6-4837752.7) | 241.2 (155.6-326.8) | 1522029.8 (1148029.8-2052008.8) | 4528 (2928.9-6129.8) | -4.6 (-4.85 - -4.34) |
| Central Latin America | 919916.7 (638019.3-1212454.2) | 103.1 (71.3-136.2) | 1135777.4 (796302.7-1552206.4) | 2031.7 (1406.1-2682.7) | -3.14 (-3.28 - -3.01) |
| Central Sub-Saharan Africa | 647568.1 (499309.4-823384.6) | 255.6 (199.9-318.5) | 1182638.7 (865084.9-1556423.4) | 5454.4 (4250.6-6849.6) | -1.08 (-1.14 - -1.02) |
| East Asia | 24088329.8 (19630124.5-28721518.7) | 280.3 (228.9-334.9) | 29566224.4 (22417299.1-37672115.4) | 5444.3 (4441.7-6490) | -2.3 (-2.62 - -1.98) |
| Eastern Europe | 6153495 (3271707.2-9097475.1) | 230.4 (122.2-339.7) | 2797506.8 (1769814.3-4130981.2) | 4214.5 (2239.5-6230.8) | -4.34 (-4.92 - -3.76) |
| Eastern Sub-Saharan Africa | 2003936.9 (1652006.4-2435042.8) | 215.9 (176.8-261.1) | 3300546.9 (2682850.5-3934494.9) | 4837.3 (3985.2-5855.6) | -1.19 (-1.25 - -1.13) |
| High-income Asia Pacific | 808579 (237528-1536996.7) | 38.6 (10.6-74.7) | 710535.7 (429616.5-1019890.6) | 754.8 (219.2-1438.7) | -3.46 (-3.78 - -3.15) |
| High-income North America | 1843521.2 (742097.4-3226827) | 49.6 (19.7-87.5) | 575238.2 (271250-934262) | 952.9 (383.3-1668) | -6.24 (-6.63 - -5.86) |
| North Africa and Middle East | 4774495.5 (3842206.4-5688147.1) | 250.6 (200.7-298.6) | 8132960.9 (6389063-9933749.6) | 5196.7 (4179.2-6187) | -1.54 (-1.61 - -1.47) |
| Oceania | 115694.5 (85519.3-152184.9) | 293.5 (222.9-375.6) | 235289.2 (171300.2-309741.8) | 6771 (5085.4-8766.8) | -0.76 (-0.79 - -0.73) |
| South Asia | 15603270.3 (13123889.9-18198537.3) | 203.8 (170.6-237.9) | 31263672.8 (25942856.3-36879755.8) | 4724.8 (3971-5503) | -0.67 (-0.77 - -0.58) |
| Southeast Asia | 7702975.4 (6330854.7-9061953.6) | 245.2 (199.3-290.7) | 10202762.6 (7621700.6-13104164.6) | 5406.5 (4431.1-6371.7) | -2.18 (-2.42 - -1.94) |
| Southern Latin America | 454923.7 (247283-690297) | 92.5 (50.6-140.6) | 233587.6 (135896.1-347002.2) | 1830.1 (995.6-2777.2) | -4.25 (-4.49 - -4.02) |
| Southern Sub-Saharan Africa | 338946.6 (261435.8-420993.9) | 101.5 (77-127.2) | 567046.6 (429611-715280.2) | 2237.1 (1720.8-2779.5) | -0.59 (-1.07 - -0.11) |
| Tropical Latin America | 1193491.6 (698013.7-1751174.1) | 112.1 (66.8-162.5) | 798762.6 (472151.5-1159966.6) | 2360.4 (1388.5-3450.5) | -4.74 (-4.89 - -4.59) |
| Western Europe | 4361300.4 (2159095.5-6947941.7) | 75.2 (37.1-120.9) | 1144443.1 (784857.1-1546998.5) | 1362.1 (673.6-2169.9) | -6.25 (-6.44 - -6.05) |
| Western Sub-Saharan Africa | 2310269.5 (1883593.7-2727622.8) | 237.3 (193.6-281.3) | 3968312.5 (3192123.7-4824695.6) | 4921 (4019.5-5809.3) | -0.89 (-1 - -0.78) |

Supplementary Table 2 Deaths of CVDs Attributable to PM2.5 Pollution by GBD Regions and CVDs Subtypes, 1990 and 2021

|  | Numbers of Deaths (95%UI)_1990 | Age-standardized rate (95%UI)_1990 | Numbers of Deaths (95%UI)_2021 | Age-standardized rate (95%UI)_2021 | Estimated annual percentage changes (95%CI) |
| --- | --- | --- | --- | --- | --- |
| Cardiovascular diseases | 3325404.4 (2781279-3893703.7) | 170.1 (140.8-200.7) | 4482496.2 (3581389.8-5401009.8) | 97.1 (77.4-117.1) | -1.92 (-2.06 - -1.79) |
| Ischemic heart disease | 681183 (533957.6-845046.6) | 37.4 (29.1-46.7) | 905602.4 (692455.4-1145389.9) | 19.9 (15.2-25.3) | -2.19 (-2.36 - -2.03) |
| Stroke | 1755017.2 (1421712.8-2117710.3) | 88.5 (71.3-107.3) | 1989686.3 (1527933.1-2496562.2) | 43 (33-54) | -2.51 (-2.71 - -2.31) |
| Intracerebral hemorrhage | 932433.8 (751623.3-1126050.5) | 44.5 (35.8-53.8) | 995650.4 (756883-1250012.4) | 21.2 (16.1-26.6) | -2.54 (-2.81 - -2.27) |
| Ischemic stroke | 1570387.1 (1194581.6-1970410.8) | 81.6 (61.4-103.2) | 2492809.9 (1860741.3-3118378.6) | 54.1 (40.3-67.8) | -1.37 (-1.46 - -1.28) |
| Subarachnoid hemorrhage | 141400.5 (87801.5-192026.3) | 6.6 (4.1-9) | 88433.5 (63858.7-118884) | 1.9 (1.4-2.5) | -4.64 (-4.91 - -4.36) |
| High SDI | 446201.4 (302227.9-617409.2) | 73.1 (49.3-101.3) | 237576.9 (168511-313295.9) | 18.5 (13.2-24.2) | -4.7 (-4.81 - -4.6) |
| High-middle SDI | 940336.2 (724812-1161085.4) | 199.8 (152.4-248.5) | 922495.6 (709522.7-1172497) | 86.5 (66.4-109.9) | -3.01 (-3.31 - -2.71) |
| Middle SDI | 1040204.5 (877004.3-1222832.6) | 226.2 (189.6-266.3) | 1647692.8 (1284967.8-2047526.4) | 125.4 (97.4-155.9) | -1.91 (-2.12 - -1.7) |
| Low-middle SDI | 648976.5 (552313.9-749729.5) | 223.4 (189.6-258.6) | 1226912.9 (1011226.2-1433722.7) | 173.2 (142.4-202.3) | -0.78 (-0.86 - -0.7) |
| Low SDI | 245189.5 (206554.3-288837.8) | 235.6 (197.9-277.1) | 444246.5 (369339.5-519892) | 191.8 (159.1-224.6) | -0.65 (-0.74 - -0.56) |
| Andean Latin America | 13175.8 (10327.3-16145.1) | 128.3 (100.7-157) | 12758.3 (8733.4-17887.3) | 40.6 (27.8-56.9) | -4.21 (-4.47 - -3.96) |
| Australasia | 2729 (94.7-7507.9) | 21.9 (0.8-60.2) | 2791.3 (1595.1-4206.4) | 8.2 (4.7-12.3) | -3.85 (-4.37 - -3.34) |
| Caribbean | 18296.4 (12721.5-25530.4) | 137.1 (94.4-192.2) | 24251.6 (16235.7-33417.9) | 81 (54.4-111.5) | -1.6 (-1.8 - -1.41) |
| Central Asia | 54552.1 (35123.4-76621.7) | 238.6 (153.1-335.2) | 65599.1 (48971.5-82933.4) | 178.3 (132.8-225.4) | -1.46 (-1.81 - -1.1) |
| Central Europe | 177291.3 (114395.7-240385.3) | 241.2 (155.6-326.8) | 90168.6 (67662.3-121852.2) | 68.7 (51.5-92.8) | -4.39 (-4.65 - -4.14) |
| Central Latin America | 40212.8 (27831.1-53024.6) | 103.1 (71.3-136.2) | 54983.5 (38288.1-74617.2) | 41.8 (29.1-56.7) | -3.1 (-3.25 - -2.96) |
| Central Sub-Saharan Africa | 24284.1 (18789.8-30655.8) | 255.6 (199.9-318.5) | 45323 (33188.1-59678.2) | 200.3 (147.3-263) | -0.96 (-1.02 - -0.9) |
| East Asia | 995635.1 (812885.6-1189868) | 280.3 (228.9-334.9) | 1516109.2 (1149660.7-1927742.3) | 143.8 (109-182.3) | -2.04 (-2.4 - -1.68) |
| Eastern Europe | 310543.7 (164997.1-457950.1) | 230.4 (122.2-339.7) | 153473.4 (96502.1-226979.4) | 77.8 (48.9-115) | -4.34 (-4.87 - -3.79) |
| Eastern Sub-Saharan Africa | 74408.1 (61196.4-90065.9) | 215.9 (176.8-261.1) | 125700.9 (101645.4-150363.9) | 165.3 (132.6-198.5) | -1.02 (-1.07 - -0.97) |
| High-income Asia Pacific | 38250.1 (10744.8-73632.4) | 38.6 (10.6-74.7) | 41800.6 (24250.5-61134.6) | 12.7 (7.5-18.3) | -3.83 (-4.17 - -3.49) |
| High-income North America | 99492.3 (39514.7-175468.4) | 49.6 (19.7-87.5) | 31507.2 (14766-51578.8) | 8.1 (3.8-13.2) | -6.41 (-6.82 - -5.99) |
| North Africa and Middle East | 191713 (154070.7-228321.6) | 250.6 (200.7-298.6) | 339832 (267054.8-412267.2) | 161.2 (126.7-194.7) | -1.42 (-1.49 - -1.36) |
| Oceania | 3929.2 (2935.3-5110.5) | 293.5 (222.9-375.6) | 8135.6 (5964.9-10626.6) | 232.1 (172-299.8) | -0.75 (-0.79 - -0.71) |
| South Asia | 563019.8 (472606.9-656295.1) | 203.8 (170.6-237.9) | 1250304.5 (1036109.8-1473194) | 171.1 (141.3-201.5) | -0.5 (-0.63 - -0.38) |
| Southeast Asia | 292409.4 (239025.7-345248.8) | 245.2 (199.3-290.7) | 414478.2 (309182-530637) | 131.9 (98.3-168.6) | -2.09 (-2.35 - -1.83) |
| Southern Latin America | 21634.5 (11817.1-32853.4) | 92.5 (50.6-140.6) | 11828.2 (6839.6-17596.7) | 23.7 (13.7-35.3) | -4.28 (-4.53 - -4.03) |
| Southern Sub-Saharan Africa | 13193.9 (10076.3-16482.1) | 101.5 (77-127.2) | 23178.1 (17477.8-29272.6) | 86.7 (64.8-109.7) | -0.44 (-0.94 - 0.07) |
| Tropical Latin America | 48310.6 (28586.6-70369.2) | 112.1 (66.8-162.5) | 34779.9 (20435.3-50464.3) | 25.2 (14.8-36.5) | -4.81 (-4.95 - -4.67) |
| Western Europe | 247398.3 (122263.1-397275.9) | 75.2 (37.1-120.9) | 74452.9 (49960.6-101264) | 11.5 (7.8-15.6) | -6.23 (-6.45 - -6.02) |
| Western Sub-Saharan Africa | 94924.9 (77406.9-112449.8) | 237.3 (193.6-281.3) | 161040.1 (129640.3-194802.9) | 188.5 (152.4-226.2) | -0.77 (-0.87 - -0.68) |

Supplementary Table 3 DALYs of CVDs Attributable to PM2.5 Pollution by 204 Countries and Territories, 1990 and 2021

| Location | Numbers of DALYs (95%UI)_1990 | Age-standardized rate (95%UI)_1990 | Numbers of DALYs (95%UI)_2021 | Age-standardized rate (95%UI)_2021 | Estimated annual percentage changes (95%CI) |
| --- | --- | --- | --- | --- | --- |
| Afghanistan | 445283 (321899.7-577717.9) | 11787.5 (8577.2-15214.4) | 478413 (348445.3-645872.3) | 8169.5 (6076.4-10677.7) | -1.44 (-1.61 - -1.27) |
| Albania | 50567.6 (40390.3-60579) | 4865.1 (3890.5-5808.1) | 36133.7 (22668-54802.9) | 1552.3 (972.5-2354.2) | -3.76 (-4.08 - -3.43) |
| Algeria | 179039.8 (120710.5-244452.2) | 3099 (2100.7-4200.5) | 349133.5 (229676.2-487453.1) | 2028.4 (1342.7-2817.8) | -1.71 (-1.94 - -1.48) |
| American Samoa | 77.5 (0-278.4) | 594.1 (0-2138.5) | 165.3 (17-380.9) | 626.1 (64.5-1438.7) | -0.04 (-0.25 - 0.17) |
| Andorra | 188 (74.4-342.6) | 624.7 (249.2-1139.6) | 116.8 (59.5-193.1) | 131 (66.9-216.9) | -4.8 (-5.15 - -4.46) |
| Angola | 117200.3 (86682.6-155954.4) | 5619.6 (4212.9-7368.8) | 183981.1 (117767.7-256997.2) | 3002.9 (1947.9-4149.3) | -2.45 (-2.62 - -2.29) |
| Antigua and Barbuda | 435.1 (104.3-928.1) | 1460.8 (349.9-3113.8) | 437.6 (158.5-783.7) | 774.6 (280.8-1386.7) | -2.23 (-2.51 - -1.96) |
| Argentina | 310614.1 (150567.6-493852.4) | 1794.4 (869.6-2853.4) | 143077.5 (74071.1-227931.8) | 463.5 (240-738.1) | -4.36 (-4.65 - -4.07) |
| Armenia | 57883.3 (33856.4-84507.8) | 4104.6 (2396.5-5992.4) | 61127.4 (42276.4-81717.6) | 2584.7 (1788.3-3455.7) | -2.08 (-2.3 - -1.86) |
| Australia | 42647.2 (1349.6-116172.6) | 400.5 (12.7-1090.4) | 38421.4 (22593.9-56499.1) | 148.7 (87.5-218.6) | -3.87 (-4.46 - -3.27) |
| Austria | 110855.8 (58480.3-171380.5) | 1681.6 (887-2599.8) | 30316.4 (20868.1-40990.7) | 277.3 (191.7-374.1) | -6.17 (-6.39 - -5.95) |
| Azerbaijan | 129130.8 (64285.3-194527.1) | 4872 (2430.9-7322.6) | 140384.6 (74988.3-218105.3) | 2770.7 (1488.4-4289) | -2.32 (-2.64 - -2) |
| Bahamas | 1245.3 (229.3-2632.4) | 1420.5 (262.3-3000) | 1832.3 (653.8-3541.7) | 815.6 (291.1-1574.7) | -1.88 (-2.09 - -1.67) |
| Bahrain | 7081.9 (5566.7-8616.1) | 7837.7 (6205.8-9457.3) | 12662.7 (9526.6-16175.6) | 2930.8 (2233.9-3682.4) | -3.79 (-4.17 - -3.41) |
| Bangladesh | 1763463.9 (1407034.9-2167449.9) | 6519.1 (5228.1-7977.5) | 3258478.8 (2405398.5-4172563.8) | 4347.4 (3224.6-5538.2) | -1.16 (-1.32 - -0.99) |
| Barbados | 2415.7 (641.7-4796.5) | 1506.2 (400.5-2985.8) | 2446.8 (1023.3-4205.7) | 866.7 (362.6-1490.9) | -1.9 (-2.26 - -1.54) |
| Belarus | 306268.7 (170227.7-441108.2) | 4411.1 (2451.7-6355.6) | 189840.6 (129261.6-262615.3) | 2150.1 (1463.3-2976.3) | -3.09 (-3.6 - -2.58) |
| Belgium | 119944 (61821.4-187103.4) | 1420.7 (732.3-2217.4) | 26367.2 (17880.1-35395.1) | 193.9 (132.4-259.8) | -6.41 (-6.72 - -6.11) |
| Belize | 1112.3 (643.3-1670.5) | 2137.7 (1236.4-3208.2) | 1808.5 (904.4-2900.6) | 1097.6 (547.5-1761.7) | -2.61 (-3 - -2.23) |
| Benin | 52457.8 (41266.7-65158.3) | 4900.2 (3862.3-6065.3) | 107139.4 (80239.9-138154.6) | 3936.3 (2986.2-5024.4) | -0.69 (-0.81 - -0.57) |
| Bermuda | 251.1 (0-659.9) | 747.8 (0-1965) | 109.4 (20.3-210.8) | 143.4 (26.5-276.2) | -5.97 (-6.4 - -5.54) |
| Bhutan | 5419.8 (3762.3-7336.2) | 3936 (2737.4-5291.1) | 6540.1 (4428.1-9003.5) | 1957.5 (1335.7-2675) | -2.49 (-2.66 - -2.31) |
| Bolivia (Plurinational State of) | 79615.3 (56584.2-111496.4) | 4490.4 (3224.2-6235.2) | 67930.5 (42354.5-107208.4) | 1394.8 (874.4-2185.7) | -4.04 (-4.13 - -3.95) |
| Bosnia and Herzegovina | 120224.8 (100816.3-140027.9) | 5686.9 (4761.2-6614.1) | 77298.6 (53605.4-106646.1) | 2235.5 (1545.8-3091) | -3.42 (-3.58 - -3.26) |
| Botswana | 12260.3 (7864.1-16915.8) | 4229.4 (2757.2-5755.6) | 10026.7 (5433.5-17049.6) | 1319 (725.7-2235.4) | -3.6 (-3.87 - -3.33) |
| Brazil | 1158956.9 (670969.1-1707036) | 2347.2 (1365.7-3446.3) | 770091.3 (449820.5-1116880.4) | 553.3 (323-802.8) | -4.77 (-4.92 - -4.62) |
| Brunei Darussalam | 367.8 (24.2-948.7) | 605.5 (39.9-1565) | 556.4 (115.9-1118.5) | 286.2 (59.8-573.1) | -2.12 (-2.91 - -1.33) |
| Bulgaria | 333735.6 (203614.7-473996.2) | 5677.7 (3463.3-8049.3) | 161350.9 (119154.1-234554.1) | 2128.2 (1568-3093.6) | -3.74 (-4.14 - -3.34) |
| Burkina Faso | 87931 (67025.4-111589.3) | 3804.7 (2905-4811.8) | 177657.4 (130396.5-234174.2) | 3598 (2670.4-4688.7) | 0.04 (-0.04 - 0.12) |
| Burundi | 95518.3 (68672.3-127215.7) | 7338.3 (5310.9-9699) | 115734.2 (84606.1-151576.2) | 4267.9 (3153-5559.9) | -2.27 (-2.52 - -2.01) |
| Cabo Verde | 3770.2 (2885.7-4661.6) | 2996.1 (2288-3711.7) | 6404.3 (4475.6-8680.1) | 2655.7 (1868.6-3579.4) | -0.9 (-1.37 - -0.43) |
| Cambodia | 170563.9 (133527.7-212785.8) | 6852.3 (5389.6-8497.8) | 304995.1 (223928.8-399019.2) | 4693.7 (3485.4-6057.2) | -1.4 (-1.48 - -1.33) |
| Cameroon | 101736.1 (75249.1-132616.4) | 4258.6 (3173.6-5505) | 285234.1 (197565-402498.1) | 4181.7 (2966.5-5822.3) | 0.01 (-0.41 - 0.43) |
| Canada | 104906.6 (29622.8-201254.8) | 589.9 (166.6-1131.6) | 30790.3 (11040.2-55278.7) | 76.3 (27.4-136.9) | -6.9 (-7.26 - -6.53) |
| Central African Republic | 47090.2 (34408-64987.1) | 7601.5 (5660.1-10252.2) | 78911.8 (53808.5-113313.6) | 6526.3 (4599.5-9068.7) | -0.59 (-0.66 - -0.52) |
| Chad | 70095.4 (54093.5-87746.3) | 4582.4 (3536.2-5725.8) | 154602.7 (112597.5-206559.5) | 4912.2 (3624.5-6478.6) | 0.15 (-0.06 - 0.36) |
| Chile | 116635.3 (82160.3-151207.9) | 2185.1 (1537.8-2834.3) | 80539.6 (53699.7-109459.8) | 571.8 (381.3-777) | -4.2 (-4.36 - -4.04) |
| China | 23431250.9 (19033094.8-27943481.1) | 5511.7 (4485.2-6569.7) | 28445610.4 (21417113.3-36450392.3) | 2595 (1955-3317.6) | -2.34 (-2.66 - -2.01) |
| Colombia | 238671.9 (160621.9-310690.8) | 2464.1 (1661-3208.7) | 169172.5 (106059.9-246085.8) | 551 (346-801.1) | -5.17 (-5.28 - -5.06) |
| Comoros | 5344.5 (3736.9-7057.1) | 4971 (3557.2-6482.1) | 8039.5 (5784.1-10687.6) | 3053.7 (2210.4-4038.7) | -1.9 (-2.16 - -1.64) |
| Congo | 41162.8 (30145-54045) | 7226.4 (5378.2-9331) | 60324.2 (41140.9-83318.8) | 4208.9 (2947.8-5641.5) | -2.14 (-2.32 - -1.96) |
| Cook Islands | 51 (1.8-141.3) | 737.3 (26.4-2036.4) | 32.9 (0-81.8) | 240.7 (0-598.9) | -3.55 (-4.09 - -3.01) |
| Costa Rica | 14860.8 (8712.4-20532.8) | 1551.3 (909-2143.2) | 11735.6 (7546.5-16665.8) | 383.7 (247.1-544.7) | -4.43 (-4.67 - -4.2) |
| Côte d'Ivoire | 111655.8 (84098.4-145693.1) | 5139.2 (3948.7-6566.2) | 270203.8 (193103.6-368194.8) | 4430.4 (3238.3-5927.6) | -0.57 (-0.79 - -0.36) |
| Croatia | 106522.8 (58378.4-157979.9) | 3446.7 (1889.1-5113) | 43576 (31646.8-56477) | 848.8 (615.2-1104.4) | -4.52 (-4.78 - -4.26) |
| Cuba | 110024.7 (43792.4-200559.1) | 1985.8 (790.1-3619.5) | 112150.2 (55149-183049.7) | 1028.8 (506.6-1679.6) | -2.3 (-2.59 - -2) |
| Cyprus | 7102.9 (3314.4-11741.8) | 2055 (961.3-3407.8) | 5140.4 (3572.6-7012.6) | 502.3 (348.4-683.3) | -5.04 (-5.27 - -4.82) |
| Czechia | 281155.8 (151932.8-420444.9) | 3727.6 (2012.3-5576.7) | 81948.1 (59342.2-106458.1) | 671.6 (486.5-874) | -5.23 (-5.53 - -4.93) |
| Democratic People's Republic of Korea | 518859.4 (380411.7-684955.7) | 6088 (4478.1-7939.1) | 1028189.2 (760327.2-1323501.2) | 5743.1 (4234.1-7396.2) | -0.16 (-0.34 - 0.02) |
| Democratic Republic of the Congo | 425963.1 (314489.3-561485.4) | 5229.9 (3907.8-6806) | 841931.1 (594769.2-1145199.6) | 4414.7 (3155.2-5952) | -0.64 (-0.68 - -0.6) |
| Denmark | 71514.3 (33121.2-118306.6) | 1582 (731.7-2612.9) | 10833.4 (6519.1-15671.4) | 160.6 (96.7-232.1) | -7.78 (-8.02 - -7.53) |
| Djibouti | 2741.7 (1815.5-3894.7) | 3640.2 (2459.9-5058.7) | 9534.3 (5844.7-14463.1) | 2739.2 (1711.5-4086.3) | -0.95 (-1.07 - -0.83) |
| Dominica | 926.1 (562.9-1290.7) | 2867.4 (1745-3997.6) | 520 (232.9-893.3) | 1156.8 (517.9-1985.1) | -2.8 (-3.28 - -2.33) |
| Dominican Republic | 58962.7 (39953.4-78861.2) | 2887.6 (1969.3-3841.3) | 84909.1 (31336.5-150136.8) | 1522.9 (563-2690.9) | -1.47 (-1.82 - -1.13) |
| Ecuador | 69558.2 (48907.8-89111.1) | 2341.5 (1646.6-2994.1) | 61170.4 (36241.7-92402.4) | 693.2 (411.6-1044.2) | -4.5 (-5.01 - -3.98) |
| Egypt | 1191516.7 (934349.6-1459975.4) | 8542.2 (6698.2-10464.8) | 2359204.4 (1779875-3017587.9) | 7295.1 (5544-9245.5) | -0.21 (-0.44 - 0.01) |
| El Salvador | 48461.7 (38668.9-58557.9) | 2889.6 (2310.5-3479.5) | 34608.9 (20898.1-50230.9) | 988.9 (597.8-1436.5) | -3.55 (-3.73 - -3.37) |
| Equatorial Guinea | 7203 (5177.2-9527.4) | 6883.7 (5010.6-8992.1) | 6178.2 (3113.4-10387.3) | 2320.3 (1200.7-3822.5) | -4.21 (-4.94 - -3.48) |
| Eritrea | 42042.5 (30435-56015.8) | 6366.4 (4662.6-8328.9) | 66126.4 (45974-91857.2) | 4251.7 (3034.7-5760.3) | -1.26 (-1.36 - -1.17) |
| Estonia | 31370.6 (12905.1-52081.4) | 2839.6 (1169-4715.8) | 2686.4 (824.1-5328.7) | 169.9 (52.2-337.3) | -10.32 (-10.96 - -9.68) |
| Eswatini | 6468.4 (4608.8-8582.6) | 4319.4 (3106.2-5664.9) | 9481.1 (4865.2-15823.4) | 3131.2 (1641.5-5105.1) | -0.6 (-1.22 - 0.02) |
| Ethiopia | 580375.1 (450583.1-786863.1) | 5137.9 (3995.7-6846) | 591867.4 (464329.2-732242.4) | 2460.8 (1932.3-3037.3) | -2.79 (-2.93 - -2.65) |
| Fiji | 14135.9 (8877.5-18885.8) | 6471.1 (4094.2-8598.8) | 12412.2 (3964.1-22302.9) | 2933.2 (945-5232.5) | -2.62 (-2.79 - -2.44) |
| Finland | 28278.9 (4897-58555.6) | 721.7 (125-1496) | 5125.8 (676.8-11076.4) | 68.4 (9.1-147.4) | -7.89 (-8.36 - -7.42) |
| France | 348261.7 (162315.1-577421) | 750.1 (348.7-1244.2) | 110235.6 (71921.7-154082) | 130.2 (85.3-181.7) | -5.71 (-6 - -5.41) |
| Gabon | 8948.8 (4943.3-13788.4) | 2943.1 (1626.3-4525.1) | 11312.4 (6451.7-17759) | 2083.7 (1200.9-3224.4) | -1.12 (-1.26 - -0.97) |
| Gambia | 9912.4 (7357.6-12903.4) | 5195.1 (3913.8-6669.3) | 29316.1 (21309.4-38024.8) | 5534 (4069-7091.2) | 0.1 (-0.03 - 0.23) |
| Georgia | 151814.1 (69149.3-235739.5) | 4566.5 (2083.3-7087.8) | 64267.4 (39435.6-92697.5) | 1950 (1200.4-2813.5) | -3.96 (-4.81 - -3.1) |
| Germany | 1294904.1 (658504.5-2037560.5) | 1826.4 (929-2873.2) | 289510.4 (193778.1-396866.2) | 255 (171.4-349.2) | -6.32 (-6.51 - -6.12) |
| Ghana | 229218.7 (176940.6-287658.6) | 6611.2 (5155.4-8214) | 438758.4 (315974.7-569049.4) | 4801.3 (3497.7-6168.3) | -1 (-1.14 - -0.86) |
| Greece | 154050.8 (83198.5-239225.9) | 1892.3 (1021.6-2940.5) | 75588 (55987-96401.3) | 554.4 (410.7-707.4) | -4.36 (-4.75 - -3.96) |
| Greenland | 116.1 (0.5-316.1) | 637.7 (3.1-1734) | 64.7 (2.4-174.2) | 178.1 (6.7-476.7) | -4.7 (-5.16 - -4.25) |
| Grenada | 1299.3 (745.2-1955.9) | 3320.8 (1906.6-5006.8) | 819.7 (327.9-1448.2) | 1342.5 (537.9-2371.3) | -2.83 (-3.19 - -2.47) |
| Guam | 284.7 (0-778) | 697.4 (0-1912.3) | 734 (348.5-1146.3) | 650.8 (308.8-1015.7) | 0.47 (-0.24 - 1.19) |
| Guatemala | 60623.3 (49392.4-71281.7) | 3273.5 (2669.8-3842.7) | 89523.2 (59084.7-118914.3) | 1528.7 (1009.6-2022.5) | -2.67 (-2.98 - -2.36) |
| Guinea | 85625.6 (64880.7-108460) | 4769.2 (3609.3-6028.4) | 149867.6 (108881.9-202121.4) | 4890.5 (3600.1-6529.7) | 0.34 (0.2 - 0.47) |
| Guinea-Bissau | 18218.6 (13377.7-24384.8) | 8204 (6103.9-10814.1) | 29029.1 (21232.4-38481.2) | 7142.4 (5323.9-9227.2) | -0.34 (-0.41 - -0.28) |
| Guyana | 11539.3 (6721.2-17698.4) | 5405.3 (3157-8288.9) | 8658 (4061.6-14431.6) | 2453.3 (1151.4-4071.2) | -2.27 (-2.39 - -2.14) |
| Haiti | 172087.9 (135384.7-213542.3) | 9726.4 (7723.9-11966.1) | 276138.5 (196831.5-377416.4) | 6951.3 (5016-9355.6) | -0.94 (-1.01 - -0.87) |
| Honduras | 39197.2 (30446.8-48411.9) | 3405.7 (2652.6-4183.5) | 110017.1 (77845.6-148045.1) | 3306.4 (2364.8-4407) | 0.12 (-0.1 - 0.34) |
| Hungary | 308884 (169905.6-467019.2) | 3966.3 (2182.3-5993.5) | 106556 (75258.9-158749.3) | 974.1 (688-1451.3) | -4.63 (-4.86 - -4.39) |
| Iceland | 599.9 (47-1399) | 373.8 (29.3-871.4) | 178.7 (32.7-395.2) | 53.2 (9.7-117.4) | -6.66 (-7.06 - -6.26) |
| India | 12133364.3 (10081126.9-14349294.9) | 4523.4 (3750.9-5346.9) | 24228447 (19906650.8-28896856.3) | 3633.7 (2982.4-4330.3) | -0.66 (-0.78 - -0.54) |
| Indonesia | 3069824.1 (2442039.6-3736757.2) | 5453.9 (4292.6-6670.2) | 4069821.2 (2780336.7-5648871.5) | 3135.7 (2144.1-4314.8) | -1.77 (-2.11 - -1.43) |
| Iran (Islamic Republic of) | 523905 (417379.6-631749.8) | 3909.5 (3100.7-4708.4) | 814713 (638617.9-983331.3) | 1965.3 (1536.9-2371.6) | -2.27 (-2.37 - -2.17) |
| Iraq | 263036.9 (191780.3-343500.1) | 5918.3 (4327.6-7699.6) | 548455.9 (374371.6-759206) | 4398.3 (3044.1-5969.9) | -1.22 (-1.36 - -1.07) |
| Ireland | 29379 (11741.4-51466.2) | 1320.7 (528.1-2316.5) | 5469 (2750.3-8581.8) | 123.4 (62-193.6) | -7.76 (-8.04 - -7.47) |
| Israel | 45215.7 (25901.5-68207.6) | 1730 (989.4-2614.7) | 22637.5 (16959-28478.1) | 319.6 (240.3-401.6) | -5.99 (-6.17 - -5.81) |
| Italy | 690062.5 (397410.2-1016554.8) | 1427.9 (820.5-2105.3) | 252109.8 (182613.6-324288.5) | 278.7 (203.7-356.6) | -5.46 (-5.66 - -5.25) |
| Jamaica | 21172.3 (13555.9-27819.6) | 2133 (1367.2-2801.7) | 14918 (8246.9-22901.3) | 854.9 (472.7-1313.5) | -2.31 (-3.07 - -1.55) |
| Japan | 467318.4 (93828.2-963332.2) | 521 (104.4-1075) | 439292.9 (235983.3-668071.2) | 219 (119.6-331.2) | -2.96 (-3.28 - -2.64) |
| Jordan | 19642.9 (14428.5-25242) | 2678.1 (1969.4-3423.8) | 61368.7 (43630.4-81580.1) | 1551.3 (1108.6-2045.6) | -2.3 (-2.63 - -1.97) |
| Kazakhstan | 243991.3 (106441.3-402426.9) | 3634.3 (1589.8-5987) | 208207 (143729.4-281673.2) | 2350 (1622.3-3177.5) | -2.61 (-3.19 - -2.02) |
| Kenya | 109336.5 (82461.5-138764.5) | 2505.3 (1888.6-3174) | 311888.2 (239194.1-401135.8) | 2630.7 (2010.4-3384.3) | 0.45 (0.22 - 0.69) |
| Kiribati | 1829 (1410.5-2301.1) | 8278.4 (6362.8-10402.2) | 2376.2 (1689.2-3251.3) | 5422.2 (3904.8-7326.1) | -1.43 (-1.63 - -1.23) |
| Kuwait | 14064 (11016.8-16979.8) | 3700 (2888.3-4462.7) | 37895.4 (27839.2-49156.7) | 2021.5 (1488.5-2614.7) | -1.6 (-2.19 - -1.02) |
| Kyrgyzstan | 90182.7 (66637.8-114897.2) | 5673.2 (4192.4-7222.8) | 95164.5 (71030.5-121135.5) | 3848.8 (2873.6-4892.7) | -1.51 (-2.03 - -0.99) |
| Lao People's Democratic Republic | 126311 (96134.9-161986.1) | 10766.5 (8252.8-13685.8) | 148618.5 (98458.5-200920.5) | 5786.5 (3867.4-7720.7) | -2.17 (-2.24 - -2.11) |
| Latvia | 94239.9 (53339-135391.1) | 4842.7 (2740.2-6961.1) | 21812.5 (14222.3-32451.1) | 953.5 (621.2-1419.4) | -6.3 (-6.81 - -5.79) |
| Lebanon | 25923 (15834.3-38539.6) | 2219.4 (1362.7-3286.4) | 30324.6 (19223.7-44152.7) | 878 (556.2-1278.4) | -3.1 (-3.48 - -2.73) |
| Lesotho | 13803.3 (10014.8-17948.9) | 3101.8 (2249.4-4034.1) | 25906 (16900.1-37647.5) | 4491.5 (2984.1-6413.8) | 2.13 (1.58 - 2.69) |
| Liberia | 30964.8 (23824.9-38678.5) | 5001 (3874.7-6204.6) | 54642.5 (39663-73946) | 4675.8 (3460.2-6219.5) | -0.33 (-0.45 - -0.21) |
| Libya | 26326.8 (18147.4-36562.3) | 2467.4 (1705.9-3415.8) | 84297.9 (57161-119018.7) | 2806.7 (1912.4-3937) | 0.79 (0.4 - 1.18) |
| Lithuania | 82619.3 (42665.3-126753.9) | 3369.3 (1739.6-5170.8) | 23317.2 (13963-33646.8) | 697.2 (417.5-1007.5) | -5.52 (-5.9 - -5.14) |
| Luxembourg | 3944.1 (1719.1-6540.2) | 1329.2 (579.4-2206.9) | 801.7 (447.1-1190.1) | 129.9 (72.5-192.9) | -7.35 (-7.67 - -7.03) |
| Madagascar | 194299.7 (156021.1-237832.9) | 6767.3 (5448.2-8254.3) | 383222.5 (275985.6-514075.3) | 5919.4 (4315.1-7812.6) | -0.56 (-0.62 - -0.5) |
| Malawi | 93827.1 (72353.5-117102) | 4500.4 (3472.3-5592.5) | 185629.6 (139540.2-239959.6) | 4518.3 (3425.2-5797.9) | -0.26 (-0.51 - -0.02) |
| Malaysia | 168514.4 (81626.7-263396.9) | 3262.8 (1581.3-5088.5) | 234048.5 (156448.9-324248.6) | 1504.2 (1005.6-2082.9) | -2.68 (-2.91 - -2.45) |
| Maldives | 3475.2 (2604.7-4448.2) | 6771.6 (5079-8572) | 1171.7 (648.7-1860.5) | 600 (333.3-949.4) | -9 (-9.44 - -8.56) |
| Mali | 91139.2 (69899.9-116033.3) | 4245.8 (3275.4-5363.3) | 164103.7 (120132.3-213683.3) | 3441.1 (2551.6-4440.6) | -0.56 (-0.67 - -0.44) |
| Malta | 3683.1 (1693.6-5999.8) | 1597.1 (734.8-2606) | 1858.1 (1272-2482) | 340.4 (233.9-454.7) | -5.07 (-5.45 - -4.69) |
| Marshall Islands | 568.1 (385.4-792.6) | 5960 (4059.4-8265.1) | 1011.2 (651.5-1547) | 4771.4 (3111.3-7203.1) | -0.39 (-0.58 - -0.21) |
| Mauritania | 32605.5 (24613.4-41402) | 6034.8 (4572.8-7624.9) | 44241.5 (31485.3-59705.7) | 3871.3 (2789.8-5167) | -1.63 (-1.81 - -1.46) |
| Mauritius | 6148.5 (2831.6-9833.1) | 1519.1 (700.2-2426.9) | 4295 (1489.7-7676.7) | 443 (153.7-791) | -4.52 (-4.98 - -4.06) |
| Mexico | 381714.5 (249185-511581.1) | 1713.7 (1118.3-2296.6) | 513773 (327890.5-733222.5) | 753.2 (480.7-1075.5) | -2.95 (-3.17 - -2.74) |
| Micronesia (Federated States of) | 2455.3 (1770.9-3244.6) | 8775.6 (6373.9-11524.7) | 2076.7 (1288.4-3156) | 4832.2 (3021.5-7279.3) | -1.97 (-2 - -1.93) |
| Monaco | 207.8 (32.7-418.9) | 521.8 (81.7-1055.3) | 117.3 (58.4-189.4) | 208.5 (103-340.4) | -2.86 (-3.69 - -2.02) |
| Mongolia | 45658 (36476.2-55051.1) | 8191.7 (6554.6-9831.5) | 46004.3 (31734.4-62809.6) | 3810.8 (2623-5171.7) | -2.95 (-3.44 - -2.45) |
| Montenegro | 12240.7 (7034.6-17706.3) | 3637.1 (2089.3-5258.4) | 12029.1 (8100.3-20284.6) | 2389.9 (1605.9-4030) | -1.69 (-2.02 - -1.37) |
| Morocco | 335896.9 (247610.4-432653.9) | 4273.7 (3159.2-5488.3) | 496309.1 (334665.3-679715.4) | 2713.5 (1841-3687.5) | -1.35 (-1.58 - -1.13) |
| Mozambique | 154253.2 (118365.1-194729.8) | 4762.3 (3652.9-5997) | 338471.1 (237422.9-454486.6) | 5399.8 (3838.4-7168.1) | 0.92 (0.72 - 1.13) |
| Myanmar | 1264221.9 (961359.6-1631212.2) | 9684.9 (7441.1-12369.3) | 1367108.5 (988923.2-1792673.8) | 5180.3 (3763.7-6778.5) | -2.28 (-2.46 - -2.1) |
| Namibia | 14977.8 (10397.7-19525.8) | 4482.6 (3122.9-5805.8) | 17057.6 (8926.7-28239.3) | 2387.1 (1260.8-3905.1) | -2.34 (-2.76 - -1.9) |
| Nauru | 35.4 (0-130.4) | 1226.3 (0-4485.5) | 43.8 (5.4-99.4) | 1216.2 (153-2729.1) | -1.26 (-2.65 - 0.16) |
| Nepal | 261201.9 (195361-336029.2) | 4951.1 (3732.8-6329.8) | 478904.6 (351715.4-625635.6) | 3790.4 (2800.5-4933.3) | -0.67 (-0.86 - -0.48) |
| Netherlands | 160443.1 (81548-250741.8) | 1458.3 (740.8-2278.6) | 38379.8 (26664.7-51648) | 190 (132.2-256) | -7.04 (-7.3 - -6.78) |
| New Zealand | 8164.3 (190.3-23992.5) | 380.3 (8.9-1118.1) | 6054.6 (2407.4-10194.7) | 127.9 (50.9-215.3) | -4.11 (-4.5 - -3.72) |
| Nicaragua | 20080.6 (16287.5-24094) | 2362.6 (1916.8-2825.9) | 32935.5 (23814.3-43869.6) | 1253.3 (907.2-1663.9) | -1.88 (-2.13 - -1.64) |
| Niger | 62267.7 (45342.8-82012.3) | 4208.1 (3083.1-5491.3) | 163714.9 (115692.6-223926.5) | 3799.9 (2725.9-5121) | -0.24 (-0.33 - -0.15) |
| Nigeria | 1128096.3 (873983.7-1418621.8) | 4831.4 (3762.7-6043.7) | 1492554.4 (1130113.4-1961317.8) | 3145.6 (2415.3-4065) | -1.57 (-1.73 - -1.41) |
| Niue | 34.6 (13-57.9) | 2841.1 (1067.7-4782.6) | 8.1 (0.8-18) | 693 (66.6-1547.2) | -5.75 (-6.3 - -5.2) |
| North Macedonia | 62806.6 (43934.8-79251.1) | 6408.9 (4474.3-8083.8) | 49575.1 (36545.9-65243.7) | 3176 (2352.2-4163.9) | -2.65 (-3.18 - -2.12) |
| Northern Mariana Islands | 92.1 (0-257.8) | 779.7 (0-2123.6) | 218.4 (102-355.7) | 761.6 (356.1-1240.8) | 0.1 (-0.54 - 0.76) |
| Norway | 32270.2 (9793.7-58753.3) | 847.3 (256.7-1540.3) | 3711.5 (1365.3-6595.7) | 63 (23.2-112) | -8.85 (-9.18 - -8.53) |
| Oman | 22262.2 (14979.8-31349.3) | 5815.7 (3954-8101.5) | 32127 (21927.9-42878.8) | 2928 (2021.2-3872.3) | -1.81 (-2 - -1.63) |
| Pakistan | 1439820.4 (1106397.6-1756262.7) | 4606.6 (3537.3-5619) | 3291302.3 (2500756.8-4192476.6) | 4696.3 (3595.2-5941.1) | -0.12 (-0.32 - 0.09) |
| Palau | 48 (0.2-132.4) | 857.9 (3.5-2368) | 89.8 (10-188.6) | 734 (80.5-1539) | 0.01 (-0.83 - 0.87) |
| Palestine | 17657 (11920.4-24348) | 3910.3 (2653.7-5356.8) | 31819.2 (21628.5-42127.9) | 2490.3 (1697.3-3282.2) | -1.57 (-1.88 - -1.27) |
| Panama | 12918.4 (7502.6-17802.4) | 1594.9 (925.9-2198.5) | 8830.6 (4733.7-14382) | 357.8 (192-582.5) | -4.93 (-5.13 - -4.74) |
| Papua New Guinea | 72095.3 (50145.4-99949.3) | 6940.4 (4946-9416.8) | 171986.5 (119207.7-234581.6) | 5797.6 (4085.3-7783.4) | -0.55 (-0.61 - -0.49) |
| Paraguay | 34534.7 (21845.9-45220.7) | 2819.5 (1782.7-3685.9) | 28671.3 (9191.5-53006.7) | 896.1 (287.1-1652.3) | -3.68 (-3.97 - -3.38) |
| Peru | 163060.2 (125495.7-204575.9) | 2403.3 (1857.1-3004.7) | 145108.8 (96450.6-208654.5) | 768.3 (511.2-1103.1) | -4.29 (-4.6 - -3.99) |
| Philippines | 792675.7 (643726.8-944099) | 4823 (3921.9-5743) | 1594472.4 (1160726.5-2036508.8) | 3427 (2496.8-4365.7) | -1.08 (-1.26 - -0.9) |
| Poland | 1124429 (745194.4-1479899.7) | 4800.3 (3182.4-6318.6) | 392816.4 (299218.8-505429.2) | 974 (742.9-1252.4) | -5.33 (-5.51 - -5.15) |
| Portugal | 86702.7 (32960.8-151805.7) | 1186.9 (451.8-2079.2) | 18480.5 (9935.5-28396.9) | 131.1 (70.6-201) | -7.51 (-7.94 - -7.08) |
| Puerto Rico | 5520.8 (0.1-17695.6) | 283.8 (0-909.8) | 3857.6 (985.1-7213.2) | 100.1 (25.5-187.4) | -3.88 (-4.39 - -3.37) |
| Qatar | 4535 (3429.3-5745.9) | 7812.7 (6041.7-9687.1) | 12904 (8796.7-17805.7) | 2417.7 (1687.1-3249.1) | -4.34 (-5.01 - -3.67) |
| Republic of Korea | 309693.7 (128173.6-532577.5) | 2069.3 (859.6-3548.2) | 253065.7 (169656.2-344356.9) | 505.1 (338-688.3) | -5 (-5.29 - -4.7) |
| Republic of Moldova | 156988.9 (123628.9-190242.3) | 7156.8 (5630.6-8686.3) | 63774.2 (40667.9-91224.1) | 1940.9 (1238.3-2775.8) | -5.25 (-5.69 - -4.8) |
| Romania | 627851.4 (365603.9-887043.1) | 4443.2 (2581.3-6275.6) | 263436.8 (188278.3-374322.9) | 1261.8 (900.3-1792.1) | -4.93 (-5.34 - -4.51) |
| Russian Federation | 3719363.2 (1781916.8-5682809.3) | 3965.1 (1899.4-6060) | 1481512.2 (880279.6-2310603.4) | 1136.9 (675.8-1773.7) | -4.95 (-5.59 - -4.31) |
| Rwanda | 126477.8 (94220.8-166400.1) | 7982.2 (5981.5-10396.9) | 116957.2 (82515.5-158848.9) | 3511.3 (2489.4-4732.4) | -3.73 (-4.17 - -3.29) |
| Saint Kitts and Nevis | 278.4 (65.9-546.6) | 1381.5 (327-2714.3) | 120.8 (42.7-216.8) | 338.7 (119.6-603.1) | -4.52 (-5.06 - -3.98) |
| Saint Lucia | 1339.4 (722.9-2049.1) | 2981.9 (1611.2-4549) | 1124.3 (472.9-1917.5) | 862.5 (362.8-1470.8) | -4.31 (-4.8 - -3.82) |
| Saint Vincent and the Grenadines | 1158.2 (689.9-1783.2) | 3011.5 (1794.6-4629.4) | 956.3 (383.3-1686.8) | 1270.8 (509-2241.6) | -2.78 (-3.12 - -2.43) |
| Samoa | 3086.4 (2332.2-3954.9) | 6640.4 (5042.8-8431.1) | 4119.5 (2661-5610.9) | 5140.8 (3336.5-6955.5) | -0.75 (-0.81 - -0.69) |
| San Marino | 114.4 (43-202.7) | 574.2 (215.7-1019.6) | 55.7 (27.5-93) | 120.5 (58.8-202.8) | -4.82 (-5.32 - -4.32) |
| Sao Tome and Principe | 1348.6 (1045.8-1650) | 3894.9 (3030.2-4755.5) | 1780.6 (1312.7-2369.5) | 2953.7 (2221.4-3863.6) | -1.01 (-1.31 - -0.7) |
| Saudi Arabia | 164959 (114454.6-225775.8) | 4908.1 (3444-6627.6) | 571456.9 (402333.1-767618.5) | 4270.5 (3128.7-5519.8) | -0.34 (-0.59 - -0.08) |
| Senegal | 95091.2 (74595.7-118063.9) | 5427.7 (4278.6-6698.4) | 187357.1 (137882.5-245579.3) | 4545.7 (3369.4-5918.6) | -0.61 (-0.68 - -0.53) |
| Serbia | 336684.8 (206076-449955.5) | 6449.3 (3936.9-8608.5) | 206902.4 (152947.1-286007.5) | 2225.6 (1643.2-3076.5) | -4.24 (-4.68 - -3.8) |
| Seychelles | 228.7 (66.8-415.8) | 734.3 (214.9-1335.5) | 270.6 (93.5-501.8) | 427.3 (147.6-793.1) | -2.28 (-2.56 - -1.99) |
| Sierra Leone | 61520.3 (46533.7-78326.1) | 5528 (4204.5-6996.2) | 107322.9 (77168.8-142418.1) | 5167 (3782.6-6761.2) | 0.02 (-0.18 - 0.21) |
| Singapore | 31199 (12436-50690.6) | 2551.5 (1015.8-4150.9) | 17620.8 (9372.7-26538) | 374.2 (199.1-564) | -5.94 (-6.35 - -5.53) |
| Slovakia | 125474.1 (67753.5-188594.1) | 3861.1 (2081.9-5801.9) | 60126.5 (43735.3-77908.6) | 1149.2 (834.4-1491.2) | -3.78 (-4.05 - -3.52) |
| Slovenia | 24738.7 (13419.8-37588.6) | 1825.7 (988.7-2773.5) | 8126.5 (5698.3-10884.6) | 307.5 (215.2-413) | -5.96 (-6.16 - -5.76) |
| Solomon Islands | 8179.2 (5260.1-11024.6) | 10713.6 (7312.9-14046.8) | 18931.2 (14000.9-25232.9) | 9406.8 (7106.3-12313.7) | -0.38 (-0.5 - -0.27) |
| Somalia | 80398.6 (55411-113322.3) | 5811.4 (4133.2-7908.1) | 153869.8 (102200.6-222577.3) | 4397.9 (2996.5-6199.1) | -0.93 (-0.97 - -0.88) |
| South Africa | 228900.6 (166201.6-295604.2) | 1915.5 (1382-2476.9) | 342275.6 (246532.2-449737.2) | 1380.1 (990.8-1809.4) | -1.17 (-1.66 - -0.67) |
| South Sudan | 63216.9 (44356.8-86145) | 4498.4 (3176.8-6096.2) | 85559.2 (58367.6-122458.8) | 4015.3 (2792.4-5639.6) | -0.59 (-0.85 - -0.33) |
| Spain | 258061.7 (113850.6-439725.2) | 878.7 (387.8-1497.2) | 80797.2 (50536.3-114927.4) | 141.2 (88.5-200.4) | -5.94 (-6.27 - -5.61) |
| Sri Lanka | 279323.8 (230777.5-331658.3) | 4989.4 (4146.9-5897.3) | 265148.7 (135703.2-442367.6) | 1864.7 (953.3-3111.2) | -2.97 (-3.44 - -2.5) |
| Sudan | 509761.1 (388082.3-648050.2) | 9796 (7502.9-12359.3) | 629968 (437328.4-869731.7) | 5682.9 (4040.8-7721.1) | -1.93 (-2.01 - -1.86) |
| Suriname | 4833.1 (2389-7585.6) | 3417.3 (1697.4-5348.8) | 6350.2 (2936.7-10427.7) | 1805 (835.5-2966.9) | -2.4 (-2.76 - -2.04) |
| Sweden | 62399.8 (16154.3-118914.3) | 721.8 (186.6-1375.5) | 8841.2 (2568.9-16749.1) | 67.2 (19.6-127.4) | -7.93 (-8.33 - -7.52) |
| Switzerland | 65824.9 (31527-107207.7) | 1115.2 (533.7-1813.9) | 13948.4 (8632.9-19897.4) | 123.2 (76.8-175.5) | -7.1 (-7.33 - -6.88) |
| Syrian Arab Republic | 140550.8 (96304.2-192640.3) | 4710.7 (3235.6-6422.4) | 255111.3 (170581.6-360997.3) | 3752.1 (2542-5254.4) | -0.94 (-1.14 - -0.73) |
| Taiwan (Province of China) | 138219.5 (58705.9-243007.6) | 1689.2 (719.4-2959.5) | 92424.8 (71023.8-117044.3) | 403.8 (310.7-511.3) | -4.08 (-4.37 - -3.8) |
| Tajikistan | 103833.2 (80817.8-126339.3) | 7045.3 (5484.1-8571.2) | 131436.9 (98948.6-166695.5) | 4596.9 (3476.8-5792) | -1.8 (-2.14 - -1.47) |
| Thailand | 645525.4 (480220-815002.3) | 3328.1 (2479.7-4185.1) | 697750.6 (482379.9-968683.2) | 1209.4 (836.2-1680.5) | -4 (-4.35 - -3.64) |
| Timor-Leste | 9224.6 (6913.7-11806.8) | 5789.3 (4413.8-7276.2) | 22034.5 (13407.5-30623.1) | 4795.1 (2932.1-6630.4) | -0.79 (-0.97 - -0.61) |
| Togo | 36538.3 (28475.5-45609) | 5409.5 (4250.4-6684) | 104335.7 (73990.2-141107.2) | 5050.1 (3651.8-6731) | -0.31 (-0.48 - -0.14) |
| Tokelau | 4.9 (0.2-16.8) | 679.5 (32.3-2337.9) | 3.5 (0-8.8) | 429.2 (3.6-1093.3) | -2.21 (-2.54 - -1.87) |
| Tonga | 1132.3 (847.4-1447.8) | 3734.2 (2794.5-4760.6) | 1000.7 (586.6-1490.5) | 2253.3 (1324-3344.2) | -1.45 (-1.58 - -1.32) |
| Trinidad and Tobago | 11718.6 (2148.3-23739.7) | 2627.9 (481.2-5321.1) | 14642 (5167.3-26971.2) | 1397.2 (492.7-2571.9) | -2.51 (-2.87 - -2.16) |
| Tunisia | 69366.3 (48705-93308.6) | 2695 (1892.9-3611.5) | 122313.9 (77355.2-178677.6) | 1737.4 (1099.4-2528.8) | -1.58 (-1.81 - -1.34) |
| Turkey | 550771.5 (396208.8-732921.1) | 2929.7 (2111.3-3881.2) | 710848.5 (511355-915606.7) | 1430.9 (1033-1839.4) | -2.27 (-2.6 - -1.94) |
| Turkmenistan | 34090.6 (10034.5-63996.1) | 3374.7 (996-6344.7) | 70856.8 (41414.8-109969.4) | 3336.5 (1952.9-5161.5) | -0.59 (-0.92 - -0.27) |
| Tuvalu | 280.8 (206.4-361.4) | 7545.9 (5567.6-9680.8) | 95.7 (54.2-152.3) | 1671.4 (948.8-2651.7) | -5.25 (-5.4 - -5.1) |
| Uganda | 151557.2 (111788.2-198653.6) | 4374.3 (3236.1-5694.6) | 258308.8 (184110.8-344133.6) | 3209.8 (2318.6-4244.1) | -1.69 (-1.98 - -1.39) |
| Ukraine | 1762644.4 (951299.4-2582029.1) | 4668.8 (2522.6-6848.8) | 1014563.7 (568997-1612065.7) | 2360.7 (1319.1-3759.2) | -3.11 (-3.68 - -2.53) |
| United Arab Emirates | 13472.6 (9561.1-18284.4) | 5076 (3677.2-6717.7) | 46364.1 (31683.1-62494.8) | 2650.4 (1888.8-3480.4) | -0.76 (-1.27 - -0.25) |
| United Kingdom | 783706.7 (358401.9-1274830.6) | 1577.9 (721.5-2565.8) | 142815.1 (90608.7-200269.5) | 197.1 (125.4-276.7) | -7.31 (-7.57 - -7.05) |
| United Republic of Tanzania | 232440.8 (177787-297662.2) | 3912.1 (2988.2-5015) | 506200.2 (365236.3-675583.9) | 3670.2 (2678-4854.9) | -0.36 (-0.43 - -0.28) |
| United States of America | 253.7 (5.7-694) | 572.8 (12.9-1564.4) | 234.1 (102.8-397.7) | 252.4 (110.5-430.7) | -2.61 (-2.99 - -2.23) |
| United States Virgin Islands | 1738456.3 (710806.1-3029377.7) | 990.5 (404.7-1725.8) | 544374.2 (259977.1-879219.4) | 172.2 (82.5-277.8) | -6.18 (-6.57 - -5.79) |
| Uruguay | 27652.7 (10762.5-47711.3) | 1311 (510-2263) | 9957.6 (3845.3-17360) | 322.8 (125-564.6) | -4.9 (-5.17 - -4.63) |
| Uzbekistan | 339316 (232822.5-455631.1) | 5429.3 (3722.2-7290.3) | 613697.6 (444176.7-795954.3) | 4629.2 (3348.2-5993.7) | -0.6 (-0.95 - -0.25) |
| Vanuatu | 3923.3 (2919.7-5147.1) | 10610.6 (8068.6-13646.6) | 9419.7 (7146.3-11757.7) | 9154.2 (7036-11328.6) | -0.59 (-0.65 - -0.53) |
| Venezuela (Bolivarian Republic of) | 103388.2 (51692.5-166190.5) | 1905.3 (951.6-3064.3) | 165181.1 (90432.5-262238.4) | 1003.7 (550-1589.7) | -2.46 (-2.62 - -2.3) |
| Viet Nam | 1155795.2 (856042.6-1493289.2) | 5344.3 (3965.4-6889.2) | 1478796.5 (1007744.3-1977674.6) | 2815.7 (1930.9-3738) | -2.04 (-2.25 - -1.84) |
| Yemen | 246831.3 (172066.3-332053) | 9049.3 (6433-12005.6) | 439684.1 (298935.7-617972.1) | 5639.2 (3866-7814.4) | -1.76 (-1.87 - -1.66) |
| Zambia | 70674.8 (52893.5-90235.4) | 4596.5 (3459.2-5832.4) | 166265 (115207.3-224212.1) | 4415.5 (3124.1-5821.3) | -0.36 (-0.5 - -0.21) |
| Zimbabwe | 62536.3 (47705.3-78890.7) | 3079.2 (2351.2-3876.7) | 162299.6 (117962.7-220453.9) | 4407.8 (3265.1-5847.8) | 1.95 (1.37 - 2.55) |

Supplementary Table 4 Deaths of CVDs Attributable to PM2.5 Pollution by 204 Countries and Territories, 1990 and 2021

| Location | Numbers of Deaths (95%UI)_1990 | Age-standardized rate (95%UI)_1990 | Numbers of Deaths (95%UI)_2021 | Age-standardized rate (95%UI)_2021 | Estimated annual percentage changes (95%CI) |
| --- | --- | --- | --- | --- | --- |
| Afghanistan | 17407.9 (12738.8-22350.1) | 523 (385.5-666.4) | 17075.5 (12600.7-22483.8) | 378.3 (283.1-485.5) | -1.25 (-1.41 - -1.09) |
| Albania | 2532.4 (2023.2-3027.1) | 275.9 (220.4-328.9) | 2230.7 (1393.9-3366.7) | 99.2 (61.9-149.5) | -3.35 (-3.67 - -3.02) |
| Algeria | 7584.3 (5137.5-10290.7) | 177.5 (120.8-238.8) | 17096.1 (11294-23766.1) | 124.6 (83-172.2) | -1.28 (-1.47 - -1.09) |
| American Samoa | 2.7 (0-9.6) | 26.5 (0-95.6) | 6.4 (0.7-14.6) | 27.8 (2.9-63.4) | -0.03 (-0.23 - 0.17) |
| Andorra | 9 (3.5-16.4) | 33.5 (13.5-61.5) | 7.1 (3.5-11.7) | 7.3 (3.7-12.1) | -4.64 (-5 - -4.28) |
| Angola | 4246.5 (3155.7-5618.3) | 257.2 (192.9-335.2) | 6983.5 (4487.5-9723.8) | 147 (95.4-203) | -2.25 (-2.42 - -2.08) |
| Antigua and Barbuda | 23 (5.6-49) | 73.1 (17.7-155.8) | 21.5 (7.8-38.6) | 41.9 (15.3-74.9) | -1.96 (-2.24 - -1.69) |
| Argentina | 14612.4 (7027.8-23241.2) | 89.5 (43-142.4) | 7255.7 (3717.3-11558.5) | 22.7 (11.7-36.2) | -4.29 (-4.59 - -3.99) |
| Armenia | 2764.2 (1614.1-4029.4) | 218.4 (127-318.5) | 3339 (2282.5-4467.6) | 140.5 (96-188) | -2.08 (-2.34 - -1.83) |
| Australia | 2307.3 (74.5-6292.8) | 22.3 (0.7-60.8) | 2416.1 (1411.2-3571.5) | 8.3 (4.9-12.3) | -3.88 (-4.43 - -3.32) |
| Austria | 6362.1 (3359.2-9881.7) | 93.4 (49.3-145.1) | 1966.6 (1320.2-2672.9) | 15.8 (10.7-21.5) | -6.06 (-6.3 - -5.82) |
| Azerbaijan | 5669.1 (2844.4-8496.7) | 243.9 (122.8-364.9) | 6564.6 (3531.2-10136.4) | 151.8 (82-233.7) | -1.77 (-2.05 - -1.48) |
| Bahamas | 50.3 (9.3-106.3) | 63.9 (11.8-134.9) | 77.2 (27.5-148.9) | 38.3 (13.7-73.9) | -1.8 (-2.02 - -1.58) |
| Bahrain | 261.6 (206.7-317.2) | 424.3 (336.6-511.3) | 464.9 (350.4-592.2) | 172.4 (132.1-215) | -3.38 (-3.81 - -2.95) |
| Bangladesh | 67342.1 (53881.5-82638.8) | 284.4 (227.8-348.3) | 142723.4 (106352.7-181218.8) | 213.3 (159.7-269) | -0.88 (-1.15 - -0.62) |
| Barbados | 136.7 (36.6-271.4) | 81.9 (21.9-162.4) | 135 (56.3-231.8) | 47.1 (19.6-80.9) | -1.88 (-2.26 - -1.51) |
| Belarus | 15648.7 (8711.8-22548.5) | 232.5 (129.4-335.3) | 10402.4 (7092-14442.1) | 115.1 (78.4-159.8) | -2.94 (-3.37 - -2.5) |
| Belgium | 6812.4 (3494.4-10725.1) | 78.6 (40.3-124) | 1650.4 (1093.9-2237.4) | 10.4 (7-14) | -6.45 (-6.78 - -6.13) |
| Belize | 51.6 (29.9-77.3) | 102 (59.1-152.6) | 79.2 (39.2-127.1) | 53.8 (26.6-86.4) | -2.51 (-2.92 - -2.1) |
| Benin | 2303.3 (1810.7-2853.1) | 237.2 (186.3-293.5) | 4530.2 (3422.7-5797) | 198.3 (151.5-251.3) | -0.54 (-0.65 - -0.43) |
| Bermuda | 12.4 (0-32.5) | 39.4 (0-103.5) | 6.4 (1.2-12.4) | 7.6 (1.4-14.7) | -5.99 (-6.42 - -5.56) |
| Bhutan | 193.6 (133.4-261.8) | 174.1 (118.4-235.6) | 294.1 (202.2-399.5) | 95.3 (65.9-128.8) | -2.13 (-2.31 - -1.96) |
| Bolivia (Plurinational State of) | 3219.6 (2312-4474.1) | 214.1 (155.5-293.2) | 3041.8 (1904.6-4785.7) | 71.3 (45-110.9) | -3.74 (-3.82 - -3.65) |
| Bosnia and Herzegovina | 5366.3 (4483.2-6235.5) | 292.1 (242.7-339.9) | 4422.9 (3053.4-6071.9) | 125.4 (86.3-172.4) | -3.14 (-3.33 - -2.95) |
| Botswana | 480.2 (311.5-654.3) | 205.3 (134.8-277.6) | 406.3 (222.2-687.5) | 65.4 (36.1-110.5) | -3.44 (-3.73 - -3.16) |
| Brazil | 46740.4 (27328.8-68403.4) | 111.3 (65.5-162.1) | 33455.1 (19454.6-48473.3) | 24.7 (14.4-35.9) | -4.85 (-4.99 - -4.71) |
| Brunei Darussalam | 13.7 (0.9-35.8) | 28.8 (1.9-75) | 19.8 (4.1-39.9) | 13.9 (2.9-27.9) | -1.84 (-2.63 - -1.04) |
| Bulgaria | 16233.9 (9922.6-23038.6) | 333.5 (203.7-471.9) | 9160.9 (6787.4-13325.2) | 116.4 (86.1-169.3) | -3.87 (-4.27 - -3.46) |
| Burkina Faso | 3498.1 (2656.2-4441) | 179.2 (136-227.4) | 7385.6 (5461.2-9647.6) | 177.4 (132.5-229.2) | 0.23 (0.12 - 0.34) |
| Burundi | 3681.1 (2665.5-4861.8) | 323.4 (235.1-424.9) | 4284.3 (3131.7-5617.7) | 199.1 (146.7-260.8) | -2.08 (-2.31 - -1.84) |
| Cabo Verde | 180.8 (137.1-223.9) | 138.4 (104.6-171.6) | 318.1 (224.2-425.9) | 139.2 (98.5-185.9) | -0.51 (-1.04 - 0.01) |
| Cambodia | 6400.7 (5025.5-7955.5) | 311.5 (245-385.5) | 12653.5 (9359.2-16331.5) | 236.1 (176.8-300.3) | -1.04 (-1.1 - -0.97) |
| Cameroon | 3992.1 (2958.8-5195.5) | 202.2 (150.7-260.4) | 11081.1 (7772.1-15563.5) | 202.9 (146.2-280.2) | 0.1 (-0.29 - 0.5) |
| Canada | 5598.2 (1595.3-10797.6) | 31.8 (9.1-61.3) | 1808.2 (642.7-3271.1) | 4 (1.4-7.2) | -7.04 (-7.45 - -6.63) |
| Central African Republic | 1690.8 (1251-2297.6) | 343.8 (258.2-455.8) | 2745.6 (1901.7-3875.1) | 301.3 (214.4-411.8) | -0.5 (-0.57 - -0.44) |
| Chad | 2962.4 (2276.9-3707.5) | 214.8 (164.4-268.7) | 6030.7 (4407.3-7996.1) | 232.7 (172.6-304.5) | 0.2 (0.01 - 0.39) |
| Chile | 5611.1 (3937.9-7284) | 115.3 (80.8-150) | 4001.5 (2652.1-5452.8) | 27.7 (18.4-37.7) | -4.43 (-4.6 - -4.26) |
| China | 969369 (788693-1159092.5) | 284.8 (232.1-340.3) | 1467819.3 (1107876.5-1874318.8) | 145.4 (109.6-185) | -2.05 (-2.42 - -1.68) |
| Colombia | 10161.9 (6860.4-13248.4) | 119.5 (80.8-156) | 8806.7 (5493.2-12855.3) | 28.2 (17.6-41.1) | -4.98 (-5.09 - -4.86) |
| Comoros | 201 (143.1-264) | 225.5 (163.7-292.6) | 330.8 (237.3-440.8) | 145.8 (104.7-193.4) | -1.71 (-1.94 - -1.47) |
| Congo | 1561.1 (1163-2013.3) | 334.7 (252.9-424.3) | 2291.1 (1585.1-3095.4) | 205.6 (145.4-271.4) | -1.92 (-2.09 - -1.75) |
| Cook Islands | 2 (0.1-5.4) | 33.3 (1.2-91.3) | 1.5 (0-3.7) | 10.8 (0-26.8) | -3.64 (-4.16 - -3.12) |
| Costa Rica | 725.3 (425-1000.8) | 80.7 (47.2-111.4) | 573.4 (365.3-815.4) | 18.4 (11.8-26.2) | -4.62 (-4.86 - -4.37) |
| Côte d'Ivoire | 3995.7 (3025.6-5167.1) | 242.1 (187.6-305.3) | 10200.4 (7373-13791.8) | 214.6 (159.4-283.9) | -0.44 (-0.64 - -0.23) |
| Croatia | 5668 (3099.1-8406.5) | 199.3 (108.9-295.8) | 2816.2 (2041.1-3643.2) | 51.5 (37.2-66.8) | -4.37 (-4.61 - -4.13) |
| Cuba | 5536.8 (2202.6-10038.2) | 104.9 (41.7-190.1) | 6131.2 (2996.2-9980.9) | 53.1 (26-86.5) | -2.38 (-2.67 - -2.1) |
| Cyprus | 394.1 (183.7-650.8) | 139.2 (65.2-231.2) | 300.2 (209.2-408.6) | 32.4 (22.6-43.9) | -5.22 (-5.43 - -5) |
| Czechia | 14602.3 (7885.7-21849.2) | 196.8 (106.1-294.8) | 5057 (3605.6-6590.7) | 39.2 (27.9-51.1) | -4.9 (-5.24 - -4.55) |
| Democratic People's Republic of Korea | 20470.2 (14925.1-26757.7) | 291 (211.6-375.5) | 43989.1 (32395.5-56302.6) | 265.2 (193.4-340.2) | -0.25 (-0.44 - -0.06) |
| Democratic Republic of the Congo | 16127.6 (11938.8-21127.6) | 248.9 (186.4-321.9) | 32587.1 (23022.7-44291.6) | 215.3 (153.2-291.6) | -0.57 (-0.61 - -0.53) |
| Denmark | 4141.8 (1928.9-6905.2) | 86.7 (40.3-144.4) | 670.6 (400.5-973.9) | 9 (5.4-13) | -7.79 (-8.07 - -7.51) |
| Djibouti | 94.1 (62.4-133) | 166.6 (112.3-230.7) | 339.8 (208.4-513.8) | 129.3 (80.5-191.9) | -0.85 (-0.97 - -0.73) |
| Dominica | 49.2 (30-68.4) | 155.9 (95.1-216.4) | 26.3 (11.8-44.9) | 61.9 (27.6-105.6) | -2.9 (-3.34 - -2.45) |
| Dominican Republic | 2447.6 (1671.4-3240.9) | 143.2 (98.5-188.5) | 3787.4 (1401-6688.3) | 70.5 (26.1-124.4) | -1.59 (-1.91 - -1.27) |
| Ecuador | 2980.8 (2089.6-3799.8) | 115.6 (81.1-147.2) | 2988.2 (1778.9-4481.5) | 36.4 (21.8-54.4) | -4.25 (-4.77 - -3.72) |
| Egypt | 47011.9 (36866.1-57600.4) | 446.1 (348.9-546.9) | 93469.5 (70837.6-118952.9) | 378.5 (289.1-475.9) | -0.11 (-0.36 - 0.13) |
| El Salvador | 2114.8 (1679.8-2544.5) | 133.7 (106.4-160.5) | 1787.9 (1073-2584.2) | 48.2 (29-69.8) | -3.37 (-3.54 - -3.2) |
| Equatorial Guinea | 272.4 (197.2-356.2) | 312.7 (229.1-405.6) | 247.4 (126.5-410.7) | 117.6 (61.5-191.8) | -3.82 (-4.51 - -3.12) |
| Eritrea | 1338.3 (969.1-1773) | 272.4 (198.2-355.3) | 2302.5 (1617-3157.5) | 196.5 (140.9-264.2) | -1.01 (-1.09 - -0.93) |
| Estonia | 1691.7 (702.4-2799) | 158.3 (65.9-262) | 173.6 (53.1-344.9) | 9.7 (3-19.2) | -10.18 (-10.83 - -9.53) |
| Eswatini | 258.2 (184.8-339.9) | 209.8 (150.7-274.3) | 356.8 (184.8-586.4) | 147 (77.7-234.9) | -0.67 (-1.21 - -0.11) |
| Ethiopia | 20402.9 (15870.5-27237.8) | 219.7 (170.4-287.7) | 23607.4 (18345.9-29329.7) | 116 (89.6-144.3) | -2.44 (-2.57 - -2.32) |
| Fiji | 466.6 (294.8-622.4) | 273.7 (174.2-363.3) | 458.8 (147.6-814.4) | 133.5 (43.6-234.5) | -2.47 (-2.69 - -2.26) |
| Finland | 1469.7 (256.7-3058.2) | 36.9 (6.5-77) | 338.5 (44.3-741.9) | 3.9 (0.5-8.5) | -7.56 (-8.06 - -7.07) |
| France | 20814.8 (9697.1-34699.3) | 42.6 (19.8-71) | 7205.6 (4587.4-10112.6) | 6.8 (4.4-9.6) | -5.95 (-6.25 - -5.64) |
| Gabon | 385.6 (213.7-590.4) | 141.1 (78-215.8) | 468.3 (268-727.9) | 104.6 (60.4-160.1) | -0.97 (-1.12 - -0.82) |
| Gambia | 382.9 (286.2-493.8) | 244.8 (185.9-310.9) | 1219.2 (892-1562.8) | 271 (200.2-344.4) | 0.27 (0.17 - 0.37) |
| Georgia | 7381.6 (3393.5-11425.6) | 235.6 (108.5-364.5) | 3498.4 (2119.2-5050) | 98.6 (60.1-142.4) | -3.79 (-4.54 - -3.04) |
| Germany | 74447.9 (37783.7-117824.5) | 101.5 (51.5-160.8) | 18382.4 (11977-25364.1) | 14 (9.2-19.3) | -6.36 (-6.57 - -6.14) |
| Ghana | 8482.5 (6565.3-10611.3) | 303.5 (237.2-375.1) | 16845.7 (12174-21719.1) | 229.9 (168.2-293.7) | -0.89 (-1.03 - -0.76) |
| Greece | 8739.6 (4730.6-13670.5) | 110.2 (59.6-172.8) | 4953.7 (3647-6330.6) | 29.3 (21.6-37.4) | -4.74 (-5.2 - -4.28) |
| Greenland | 4.3 (0-11.8) | 31.6 (0.2-85.9) | 2.7 (0.1-7.2) | 8.8 (0.3-23.6) | -4.72 (-5.2 - -4.24) |
| Grenada | 65.1 (37.3-97.8) | 152.2 (87.3-228.8) | 37.1 (14.9-65.4) | 67.9 (27.4-119.7) | -2.64 (-2.93 - -2.34) |
| Guam | 10.5 (0-28.8) | 34.8 (0-95.6) | 27.6 (13-43.4) | 23.4 (11.1-36.7) | -0.42 (-1.21 - 0.38) |
| Guatemala | 2380.3 (1940.4-2791.6) | 168.2 (137-197.2) | 4253.4 (2803.8-5615.7) | 83.4 (55.1-109.6) | -2.38 (-2.65 - -2.1) |
| Guinea | 3604.5 (2703.5-4571.5) | 224 (167.3-283.6) | 6208.2 (4560.4-8315.6) | 235.1 (175.3-311) | 0.42 (0.29 - 0.56) |
| Guinea-Bissau | 666.1 (495-878.8) | 360.3 (272.1-466.1) | 1030.8 (761.2-1342.2) | 333 (249.9-423.6) | -0.13 (-0.19 - -0.06) |
| Guyana | 457.9 (267.9-703) | 242.9 (142.4-373) | 357 (167.8-591.2) | 115.6 (54.4-190.6) | -2.13 (-2.29 - -1.96) |
| Haiti | 6580.4 (5234.3-8091.8) | 453.2 (363.8-552.5) | 10673.1 (7692.6-14381.1) | 333.6 (243-442.9) | -0.86 (-0.92 - -0.8) |
| Honduras | 1560.7 (1213.8-1915.9) | 161.4 (125.4-197.5) | 5021.3 (3605.1-6674.2) | 175.2 (126.6-231.2) | 0.47 (0.18 - 0.75) |
| Hungary | 15076.6 (8295.1-22789.3) | 201.2 (110.8-303.7) | 6263.1 (4370.3-9301.8) | 53 (37-78.7) | -4.3 (-4.56 - -4.03) |
| Iceland | 34.1 (2.7-79.7) | 20.3 (1.6-47.5) | 11.5 (2.1-25.7) | 3 (0.5-6.8) | -6.45 (-6.9 - -5.99) |
| India | 428415.7 (354589.3-506800.5) | 192.1 (158.2-227.5) | 964920.4 (789182.7-1151094.2) | 161.7 (131.9-192.6) | -0.45 (-0.62 - -0.29) |
| Indonesia | 108634.5 (84853.9-133219.2) | 237.2 (182.3-293.5) | 156398.5 (106531-215401.8) | 150.4 (102.6-205.2) | -1.46 (-1.81 - -1.1) |
| Iran (Islamic Republic of) | 20934.8 (16580.4-25243.2) | 200.1 (157.1-241.2) | 38939.7 (30223.9-47130.3) | 105.5 (81.6-127.7) | -2.13 (-2.24 - -2.02) |
| Iraq | 11041.4 (8066.6-14362.7) | 269.2 (197-349.3) | 23193.1 (16030.7-31464.2) | 229.3 (161.2-305) | -0.83 (-0.99 - -0.67) |
| Ireland | 1552.3 (620.9-2720.2) | 71.2 (28.5-124.9) | 328.8 (163.9-518.1) | 7 (3.5-11.1) | -7.53 (-7.83 - -7.23) |
| Israel | 2383.6 (1358.1-3609.1) | 95.2 (54-144.6) | 1350.7 (985.1-1712.4) | 17.5 (12.8-22.1) | -6.03 (-6.22 - -5.84) |
| Italy | 39005.4 (22325.3-57869.7) | 80.8 (46.1-120.3) | 17987.9 (12714.2-23352.7) | 16.6 (11.9-21.5) | -5.27 (-5.5 - -5.04) |
| Jamaica | 1105.8 (708.1-1451.4) | 108.6 (69.6-142.4) | 798.2 (440-1215.2) | 43.5 (24-66.4) | -2.31 (-3.07 - -1.54) |
| Japan | 24333.3 (4903.8-50340.5) | 28.8 (5.8-59.6) | 27456.2 (14283.5-42313.5) | 10 (5.3-15.2) | -3.57 (-3.92 - -3.21) |
| Jordan | 756 (554.1-970.9) | 130 (95.1-166) | 2428.9 (1719.9-3240.5) | 79.5 (56.5-104.8) | -2.1 (-2.43 - -1.76) |
| Kazakhstan | 11060.6 (4857.1-18170.3) | 183 (80.6-300.5) | 10153.9 (7024.1-13738.2) | 134.1 (92.6-181.5) | -2.1 (-2.61 - -1.58) |
| Kenya | 4446.5 (3285.2-5678.6) | 120.2 (88.3-153.4) | 12471.6 (9415.5-16177.5) | 131.4 (98.1-171) | 0.59 (0.41 - 0.78) |
| Kiribati | 59.2 (45.2-74.6) | 328.2 (246.8-416.2) | 75.9 (54.4-103) | 221.6 (160.9-295.7) | -1.33 (-1.54 - -1.13) |
| Kuwait | 478.7 (371.9-580.1) | 171.6 (132.4-208.2) | 1365.2 (990.1-1790.4) | 96.7 (70.3-126) | -1.53 (-2.14 - -0.91) |
| Kyrgyzstan | 4077.7 (3016.3-5185.1) | 280.5 (207.1-356.5) | 4297.7 (3201.8-5477.8) | 204.5 (152.1-260.6) | -1.07 (-1.53 - -0.61) |
| Lao People's Democratic Republic | 4552.4 (3491-5779.5) | 464.1 (357.8-583.2) | 5755.9 (3839.2-7692.3) | 275 (185.1-362.3) | -1.86 (-1.92 - -1.8) |
| Latvia | 4984.8 (2828.2-7152.3) | 260.5 (147.8-373.9) | 1354.8 (881.5-2016.8) | 52.8 (34.3-78.7) | -6.05 (-6.52 - -5.59) |
| Lebanon | 1097.6 (677.4-1621.3) | 107.9 (66.9-158.6) | 1680.5 (1064.6-2457.3) | 46.2 (29.2-67.5) | -2.9 (-3.22 - -2.57) |
| Lesotho | 616.3 (445.3-801.4) | 154.3 (110.5-202) | 1031.8 (685.7-1464.5) | 211.5 (143.2-294.3) | 2.01 (1.47 - 2.55) |
| Liberia | 1290.6 (1002.9-1599.2) | 240.2 (187.8-295.8) | 2105.8 (1544.6-2823.8) | 228.4 (171-300.6) | -0.25 (-0.36 - -0.14) |
| Libya | 1107.8 (757.9-1538) | 118.1 (81-163.5) | 3351.2 (2267.1-4713.6) | 134.7 (91.5-188.1) | 0.94 (0.52 - 1.36) |
| Lithuania | 4452.1 (2287.8-6826.2) | 183.7 (94.3-281.9) | 1504.8 (898.5-2176.7) | 40.1 (23.9-58.1) | -5.35 (-5.7 - -5.01) |
| Luxembourg | 221.2 (96.9-367.6) | 75.7 (33.2-126) | 50.3 (27.7-75) | 7.5 (4.1-11.2) | -7.18 (-7.51 - -6.85) |
| Madagascar | 7186.8 (5758.2-8800.8) | 296 (236.9-361.9) | 13099.1 (9383.2-17516.6) | 267.3 (193-352.9) | -0.47 (-0.53 - -0.41) |
| Malawi | 3541.4 (2717.5-4427.5) | 207.2 (158.2-259.3) | 7008.6 (5285.8-9048.3) | 210.3 (158.9-271) | -0.19 (-0.4 - 0.03) |
| Malaysia | 6786.6 (3271.3-10580.9) | 145.8 (70.2-227.2) | 9683.4 (6458.7-13438.4) | 70 (46.5-97.2) | -2.58 (-2.81 - -2.35) |
| Maldives | 118.2 (88.6-149.6) | 295.1 (220.1-370.1) | 49.7 (27.4-78.6) | 31.6 (17.5-49.9) | -8.36 (-8.76 - -7.95) |
| Mali | 3456.6 (2655.7-4376.2) | 196.9 (151.6-247.8) | 6387.6 (4688.2-8310.8) | 165 (122.7-212.6) | -0.41 (-0.53 - -0.3) |
| Malta | 193.7 (89.1-316) | 88.2 (40.6-144.3) | 115.9 (78.1-156.2) | 18.9 (12.8-25.5) | -5.01 (-5.43 - -4.59) |
| Marshall Islands | 19.8 (13.5-27.4) | 256.4 (174.7-354) | 32 (20.7-48.9) | 202.2 (133.2-301.9) | -0.44 (-0.62 - -0.26) |
| Mauritania | 1355.9 (1026.8-1714.3) | 285.2 (216.3-359.4) | 1958.4 (1403.7-2621) | 196.6 (142.2-260.9) | -1.4 (-1.58 - -1.22) |
| Mauritius | 240.1 (110.4-382.6) | 69.3 (31.8-110.3) | 188.9 (65.1-335.4) | 20.1 (6.9-35.6) | -4.59 (-5.06 - -4.12) |
| Mexico | 17457.5 (11415.2-23379.1) | 93.8 (61.3-125.7) | 25007.4 (15909.5-35604.3) | 39.7 (25.2-56.5) | -3.01 (-3.24 - -2.78) |
| Micronesia (Federated States of) | 88.2 (64.4-115.5) | 368.4 (269-480.8) | 70.2 (43.7-106.1) | 204.7 (129-305.2) | -1.95 (-1.99 - -1.91) |
| Monaco | 12.9 (2-26) | 28.9 (4.6-58.3) | 7.7 (3.8-12.5) | 11.6 (5.7-18.8) | -2.85 (-3.67 - -2.02) |
| Mongolia | 1988.2 (1595.2-2382.8) | 406.8 (325.6-485.6) | 1870.2 (1287-2538.3) | 197.5 (134.7-267) | -2.83 (-3.3 - -2.36) |
| Montenegro | 604.2 (345.4-872.2) | 189.7 (108.4-273.9) | 694.8 (468-1177.1) | 148.9 (100.1-252) | -1.04 (-1.31 - -0.76) |
| Morocco | 14257.9 (10567.7-18325.4) | 204 (151.1-261.8) | 22646.9 (15431.7-30459.3) | 140.1 (96-187.4) | -1.05 (-1.27 - -0.84) |
| Mozambique | 5935.9 (4524.3-7504) | 217 (164.1-275.2) | 12335 (8690.1-16415.3) | 240.4 (169.9-318.8) | 0.82 (0.64 - 1) |
| Myanmar | 46339.1 (35582.7-59106.5) | 420.7 (326.9-530.8) | 57131.8 (41367.1-74955) | 247.6 (180.3-324.2) | -1.97 (-2.13 - -1.81) |
| Namibia | 599.8 (419-777.2) | 218.9 (152.5-282.6) | 714.7 (377.5-1167) | 120.2 (63.8-194.2) | -2.2 (-2.61 - -1.79) |
| Nauru | 1.1 (0-4) | 49.6 (0-180.4) | 1.4 (0.2-3.2) | 49.6 (6.4-109.8) | -1.22 (-2.58 - 0.16) |
| Nepal | 9613.1 (7229.2-12317.3) | 219.6 (166.2-280.2) | 20151.6 (14972.5-26243.9) | 181.8 (135.7-236.2) | -0.4 (-0.59 - -0.21) |
| Netherlands | 8561.3 (4334.8-13456.9) | 76 (38.5-119.6) | 2441.1 (1676-3290.8) | 11.1 (7.6-14.9) | -6.71 (-6.95 - -6.47) |
| New Zealand | 421.8 (9.5-1248.2) | 19.8 (0.4-58.7) | 375.2 (149.2-630.8) | 7.3 (2.9-12.4) | -3.76 (-4.09 - -3.43) |
| Nicaragua | 858.8 (692.6-1027.1) | 117 (94.2-139.8) | 1506.5 (1078.5-2009.3) | 64.9 (46.4-86.2) | -1.63 (-1.92 - -1.35) |
| Niger | 2325.6 (1684.2-3059.8) | 198.5 (143.8-259.2) | 6516 (4603.9-8860.4) | 187.5 (134.9-250.2) | -0.08 (-0.16 - 0) |
| Nigeria | 48512.6 (37733.6-60657) | 239.6 (187.2-298) | 62903.2 (48157.2-81513.8) | 162 (125.6-206.1) | -1.41 (-1.56 - -1.26) |
| Niue | 1.7 (0.6-2.7) | 129.2 (49.2-212.8) | 0.4 (0-0.8) | 32.1 (3.1-71) | -5.67 (-6.21 - -5.13) |
| North Macedonia | 2981.6 (2074.7-3759.7) | 331.9 (230.4-418.5) | 2665.4 (1977.3-3487.5) | 203.1 (151.6-264.3) | -2.01 (-2.66 - -1.36) |
| Northern Mariana Islands | 2.7 (0-7.5) | 34.8 (0-93.9) | 8 (3.7-13) | 34.8 (16.3-56.6) | 0.12 (-0.52 - 0.76) |
| Norway | 1836.6 (559.7-3363.6) | 44.5 (13.6-81.5) | 232 (85.3-412.6) | 3.5 (1.3-6.2) | -8.72 (-9.1 - -8.33) |
| Oman | 856.6 (579.5-1196.7) | 270.6 (184.1-374.4) | 1206.9 (827.1-1608.5) | 154.3 (107.2-204) | -1.33 (-1.51 - -1.16) |
| Pakistan | 57455.4 (43929.9-70355.8) | 208.1 (158.1-255.7) | 122215 (93157.4-155104.2) | 214.3 (164.1-270.4) | -0.06 (-0.24 - 0.13) |
| Palau | 1.7 (0-4.8) | 37.3 (0.2-103.2) | 3.2 (0.4-6.7) | 32.4 (3.5-67.7) | 0.11 (-0.74 - 0.98) |
| Palestine | 832.8 (567.4-1134.1) | 211.5 (144.7-286.1) | 1403.3 (954.2-1852.9) | 139.8 (95.2-183.8) | -1.44 (-1.79 - -1.09) |
| Panama | 624 (362.6-861.3) | 82.6 (48-114.1) | 450.5 (239.4-735.5) | 17.8 (9.5-29.1) | -5.03 (-5.24 - -4.82) |
| Papua New Guinea | 2435.2 (1720.5-3327.7) | 302.8 (219-405.2) | 5901.9 (4121-7975.2) | 259.4 (183.3-345.9) | -0.46 (-0.53 - -0.39) |
| Paraguay | 1570.1 (989.9-2053.6) | 139.1 (87.7-181.7) | 1324.8 (421.3-2437.6) | 44 (14-80.7) | -3.57 (-3.82 - -3.32) |
| Peru | 6975.4 (5387.3-8723) | 113.9 (88.2-142.1) | 6728.3 (4455.6-9716.2) | 36.2 (24-52.2) | -4.35 (-4.68 - -4.01) |
| Philippines | 29502.5 (23945.9-35081.4) | 236.9 (192.6-282.2) | 60328.6 (43824.8-76925.8) | 153 (111.1-194.6) | -1.3 (-1.47 - -1.13) |
| Poland | 55153.2 (36675.7-72534.4) | 246.8 (164.1-324.6) | 23353.6 (17664.8-30243.7) | 54.3 (41.1-70.3) | -5.09 (-5.28 - -4.9) |
| Portugal | 4828.9 (1830.3-8462.9) | 70.2 (26.7-123.2) | 1193.8 (638.6-1832.7) | 7.1 (3.8-10.9) | -7.8 (-8.26 - -7.35) |
| Puerto Rico | 282.7 (0-910.4) | 15.3 (0-49.2) | 218 (55.5-409.4) | 4.7 (1.2-8.8) | -4.26 (-4.75 - -3.77) |
| Qatar | 152.8 (116-192.6) | 430.8 (334.6-530.3) | 386 (259.9-538.3) | 138.6 (97.4-185.3) | -4.39 (-5.15 - -3.62) |
| Republic of Korea | 12650.7 (5240.9-21819.5) | 108.5 (45.1-186.6) | 13526.3 (8916.6-18563.2) | 27.1 (17.8-37.2) | -4.96 (-5.25 - -4.66) |
| Republic of Moldova | 7647.8 (6015.6-9276) | 404.6 (317.7-492.2) | 3297.5 (2078.6-4743.7) | 98.8 (62.3-142) | -5.61 (-6.01 - -5.21) |
| Romania | 31436.3 (18265.6-44456.8) | 250.9 (145.2-354.5) | 15547.3 (11116.9-22149.3) | 69 (49.2-98.3) | -4.91 (-5.29 - -4.52) |
| Russian Federation | 182820.6 (87813-278466.4) | 213.7 (102.6-326) | 78923.9 (46830.9-122428.9) | 59.4 (35.2-92.1) | -4.95 (-5.53 - -4.36) |
| Rwanda | 4605.6 (3442.2-6000.6) | 352 (263.1-455.2) | 4639.7 (3257.8-6304.9) | 172.6 (120.8-234.2) | -3.29 (-3.7 - -2.88) |
| Saint Kitts and Nevis | 14 (3.3-27.4) | 70.8 (16.9-138.4) | 5.4 (1.9-9.7) | 18.1 (6.4-32) | -4.24 (-4.73 - -3.76) |
| Saint Lucia | 67.1 (36.5-102.1) | 167.7 (91.1-253.9) | 58.9 (24.9-100.7) | 46.6 (19.7-79.6) | -4.63 (-5.18 - -4.08) |
| Saint Vincent and the Grenadines | 57.2 (34.1-87.7) | 158.8 (94.8-243.1) | 48.2 (19.3-85.1) | 68.8 (27.5-121.4) | -2.66 (-3.02 - -2.3) |
| Samoa | 121.1 (92.1-153.1) | 303.8 (231.2-381.3) | 162.2 (105.7-218.4) | 231.7 (151.4-310.1) | -0.81 (-0.87 - -0.75) |
| San Marino | 7 (2.6-12.4) | 33.7 (12.6-60) | 3.6 (1.7-6.1) | 6.3 (3-10.8) | -5.05 (-5.61 - -4.49) |
| Sao Tome and Principe | 59 (45.9-72.3) | 186.6 (145.6-228.4) | 71.8 (53.8-94.4) | 144.8 (110-188) | -0.81 (-1.06 - -0.55) |
| Saudi Arabia | 6356 (4455-8592.8) | 235.3 (166.5-314.6) | 17417.6 (12482.8-22992.7) | 196.2 (145.7-250.6) | -0.6 (-0.84 - -0.35) |
| Senegal | 3849.5 (3022.2-4761.7) | 255.3 (201-314.1) | 7996.8 (5879.1-10436.8) | 227 (168-294.5) | -0.43 (-0.5 - -0.37) |
| Serbia | 17286.3 (10543.2-23052.3) | 384.6 (234.2-512.2) | 12658.3 (9350.1-17508.3) | 132.3 (97.7-183) | -4.33 (-4.76 - -3.9) |
| Seychelles | 10 (3-18.1) | 32.5 (9.6-58.6) | 11.5 (4-21.5) | 20.3 (7-38) | -2 (-2.28 - -1.72) |
| Sierra Leone | 2609.3 (1991.9-3281.5) | 262.6 (201.2-328.2) | 4316.7 (3159.7-5653) | 248.3 (185.2-320) | 0.03 (-0.16 - 0.22) |
| Singapore | 1252.2 (500.1-2027.9) | 116.9 (46.6-189.5) | 798.4 (427.3-1211.6) | 17.3 (9.3-26.3) | -5.94 (-6.31 - -5.56) |
| Slovakia | 6226.5 (3362.6-9331.2) | 197.2 (106.3-295.6) | 3469.1 (2514.6-4500.8) | 66.1 (47.9-85.9) | -3.34 (-3.6 - -3.09) |
| Slovenia | 1288.6 (692.2-1953.2) | 96 (51.4-145.6) | 516.7 (360.4-691.1) | 17.3 (12.1-23.2) | -5.71 (-5.91 - -5.51) |
| Solomon Islands | 281.7 (188.8-371.6) | 475.9 (343.5-606.2) | 656.7 (492.4-867.4) | 417.7 (320.2-537.8) | -0.4 (-0.49 - -0.3) |
| Somalia | 2685.2 (1874.4-3715.1) | 253.3 (181.9-338) | 5191.6 (3468.6-7430.9) | 195.5 (133.5-273.4) | -0.81 (-0.85 - -0.78) |
| South Africa | 8577.1 (6089.2-11159.9) | 83.6 (59-108.9) | 14517.2 (10345.8-18988.7) | 68.1 (48.3-89) | -0.8 (-1.33 - -0.26) |
| South Sudan | 2529.2 (1779-3428.7) | 204.3 (144.5-275.3) | 3162.3 (2161.3-4494.5) | 184.2 (128.5-255.7) | -0.54 (-0.77 - -0.31) |
| Spain | 14640.7 (6472.3-25161.9) | 50.1 (22.2-86.2) | 5061.3 (3112.5-7221.9) | 7.3 (4.5-10.4) | -6.18 (-6.53 - -5.84) |
| Sri Lanka | 11982 (9972.7-14166.1) | 260.5 (217.5-306.7) | 12764.9 (6446.4-21373.5) | 97.8 (49.3-163.6) | -2.75 (-3.22 - -2.28) |
| Sudan | 19894.3 (15288.1-25018.8) | 449.1 (345.6-560.8) | 25038.9 (17744.3-33982.6) | 276.1 (198.7-368.8) | -1.75 (-1.82 - -1.68) |
| Suriname | 206.2 (103.2-322) | 158.3 (79.4-246.6) | 272.3 (126.5-452.1) | 81.5 (37.9-135.5) | -2.39 (-2.74 - -2.04) |
| Sweden | 3769 (986.5-7193.7) | 40.7 (10.6-77.7) | 587.8 (171.9-1123.6) | 3.9 (1.1-7.4) | -7.87 (-8.33 - -7.42) |
| Switzerland | 3967.4 (1896.5-6504.3) | 63.6 (30.3-104.1) | 978.1 (591.3-1402.8) | 7.4 (4.5-10.6) | -6.93 (-7.17 - -6.68) |
| Syrian Arab Republic | 5594.5 (3838.8-7608.6) | 224.4 (154.6-303) | 10984.7 (7416.8-15387.4) | 199 (137-274.8) | -0.56 (-0.75 - -0.37) |
| Taiwan (Province of China) | 5795.9 (2485.6-10074.7) | 84.2 (36.1-145.5) | 4300.8 (3230.1-5525) | 17.8 (13.4-22.8) | -4.52 (-4.81 - -4.22) |
| Tajikistan | 4699.3 (3624.4-5744.9) | 348.4 (268.3-426.4) | 5684.6 (4275.8-7191.5) | 247.4 (186.3-311.6) | -1.5 (-1.86 - -1.13) |
| Thailand | 25399.9 (18840.2-32101.3) | 157.3 (116.4-198.2) | 30929.2 (21104.2-43152.5) | 52.1 (35.6-72.7) | -4.32 (-4.68 - -3.95) |
| Timor-Leste | 324 (244.4-410.8) | 269.5 (206.2-336.4) | 937.2 (575.5-1295.4) | 234.2 (144.6-322.2) | -0.61 (-0.77 - -0.44) |
| Togo | 1394.2 (1090.4-1732.3) | 254.4 (200.2-313.4) | 3931.8 (2801.1-5294.8) | 243.6 (177.7-322.4) | -0.23 (-0.39 - -0.08) |
| Tokelau | 0.2 (0-0.7) | 31 (1.5-107.2) | 0.2 (0-0.4) | 19.5 (0.2-49.3) | -2.2 (-2.53 - -1.87) |
| Tonga | 44.1 (32.7-56.3) | 166.4 (123-212.3) | 43.7 (25.8-64.3) | 103.1 (61.1-151.3) | -1.34 (-1.48 - -1.19) |
| Trinidad and Tobago | 521.8 (95.1-1055) | 132.8 (24.2-268.6) | 685.9 (241.3-1259.8) | 66.7 (23.4-122.3) | -2.6 (-2.93 - -2.27) |
| Tunisia | 3066.2 (2160.1-4108.5) | 143.2 (100.5-191.2) | 6104.2 (3844.6-8866.1) | 94.4 (59.4-136.4) | -1.5 (-1.74 - -1.27) |
| Turkey | 23251.9 (16704-30759.9) | 143.3 (102.8-189.1) | 36490.2 (26344-46901) | 79.1 (57.3-101.5) | -1.68 (-2.08 - -1.27) |
| Turkmenistan | 1466.3 (433.5-2766.8) | 167.6 (49.7-317.2) | 3064.5 (1790.7-4740.2) | 165.7 (97-255.5) | -0.64 (-0.94 - -0.34) |
| Tuvalu | 10 (7.4-12.8) | 324.3 (241.2-412.5) | 3.6 (2.1-5.8) | 72.7 (41.5-114.7) | -5.23 (-5.37 - -5.08) |
| Uganda | 6025.8 (4421.7-7888.1) | 204.2 (149.1-266.6) | 9935.2 (7093.2-13301.1) | 151.2 (108.8-201.9) | -1.59 (-1.87 - -1.32) |
| Ukraine | 93298 (50572.8-136450) | 264.1 (143.4-387) | 57816.4 (32636.7-91875.7) | 131.6 (74.1-209.4) | -3.17 (-3.74 - -2.59) |
| United Arab Emirates | 440.5 (314.1-593.4) | 244.4 (177.4-321.2) | 1343.7 (914.2-1828) | 150.7 (107.8-197.5) | 0.2 (-0.42 - 0.83) |
| United Kingdom | 42989.4 (19698.5-70488.3) | 82.6 (37.9-135.5) | 8561.5 (5355.9-11978.8) | 10.4 (6.5-14.6) | -7.29 (-7.57 - -7) |
| United Republic of Tanzania | 8973.4 (6807.5-11554.9) | 178.5 (134.1-231.5) | 20557.4 (14947.4-27408.8) | 177 (129.7-234.2) | -0.11 (-0.19 - -0.03) |
| United States of America | 10.9 (0.2-29.8) | 30 (0.7-81.9) | 12.4 (5.5-21) | 13.1 (5.8-22.3) | -2.61 (-2.96 - -2.26) |
| United States Virgin Islands | 93887.5 (37833.6-164641.2) | 51.4 (20.7-90.1) | 29695.8 (14120.3-48328.7) | 8.6 (4.1-13.9) | -6.33 (-6.75 - -5.91) |
| Uruguay | 1410 (553.8-2415.1) | 66.6 (26.2-114.1) | 570.3 (218.1-988.4) | 16.2 (6.2-28.2) | -4.88 (-5.13 - -4.63) |
| Uzbekistan | 15445.1 (10531.5-20816.2) | 265.8 (180.8-358.6) | 27126.3 (19504-35165.3) | 244.4 (175.6-316.4) | -0.29 (-0.65 - 0.07) |
| Vanuatu | 130 (98.2-168.3) | 455.9 (351.7-576) | 316.7 (242.8-392.9) | 391.8 (303.4-481.7) | -0.58 (-0.62 - -0.53) |
| Venezuela (Bolivarian Republic of) | 4329.7 (2160.8-6973.4) | 90.7 (45.2-146.2) | 7576.4 (4145-11945.8) | 48.4 (26.5-76) | -2.34 (-2.49 - -2.18) |
| Viet Nam | 51696.2 (38238.3-66590.6) | 260.9 (193.2-335.5) | 67067 (46046.5-88779) | 144 (99.4-189.3) | -1.93 (-2.15 - -1.7) |
| Yemen | 9222.6 (6559.1-12248.2) | 416.5 (301.6-544.6) | 17428.1 (11917.3-24189.2) | 277.9 (190.7-380.7) | -1.53 (-1.63 - -1.42) |
| Zambia | 2708 (2020.9-3462.8) | 214.4 (160.1-273) | 6326.2 (4421.7-8414.2) | 211.9 (150.5-277.4) | -0.19 (-0.32 - -0.07) |
| Zimbabwe | 2662.3 (2017.3-3364.3) | 159.3 (120.3-201.3) | 6151.3 (4515.4-8203.6) | 210.1 (156.6-273.9) | 1.74 (1.19 - 2.28) |

Supplementary Table 5 The Deaths and DALYs of CVDs Burden Attributable to PM2.5 Pollution in 1990 and 2021 in Males

|  | Numbers of Deaths (95%UI) | | Age-standardized rate (95%UI) | | Numbers of DALYs (95%UI) | | Age-standardized rate (95%UI) | | Estimated annual percentage changes (95%CI) | |
| --- | --- | --- | --- | --- | --- | --- | --- | --- | --- | --- |
|  | 1990 | 2021 | 1990 | 2021 | 1990 | 2021 | 1990 | 2021 | ASMR | ASDR |
| Cardiovascular diseases | 1695327.5 (1418163.2-1987346.1) | 2468592.3 (1962489.1-2998788.5) | 196.5 (163.1-231.6) | 120.1 (95.3-146) | 43362305 (36483610.4-50524047) | 58299045.1 (46814284.9-70446739.3) | 4296.3 (3601.8-5021) | 2631 (2110-3182.2) | -1.67 (-1.83 - -1.52) | -1.69 (-1.83 - -1.54) |
| Ischemic heart disease | 829335.9 (633079.1-1034562.2) | 1412828.3 (1047855.3-1782889.1) | 96.9 (73.3-121.8) | 68.8 (50.9-86.9) | 21320191 (16412315.4-26480966) | 33494555.1 (25175517.4-42016922.5) | 2102.5 (1609.8-2621.7) | 1509.3 (1132.2-1894.9) | -1.13 (-1.23 - -1.02) | -1.14 (-1.24 - -1.04) |
| Stroke | 865991.6 (695213.8-1049530.9) | 1055764 (797663.1-1335195.2) | 99.6 (79.7-121.3) | 51.3 (38.7-65) | 22042114 (17768616-26600599.3) | 24804490 (18787803.6-31135699.8) | 2193.8 (1765.4-2655.2) | 1121.7 (849.3-1409.7) | -2.29 (-2.51 - -2.06) | -2.3 (-2.51 - -2.1) |
| Intracerebral hemorrhage | 483263 (381679.1-587838.7) | 542639.6 (404397-691594.5) | 51.3 (40.5-62.5) | 25.3 (18.9-32.3) | 13001903.3 (10259155.3-15783435.5) | 13609247.3 (10183015.2-17230685.5) | 1228.9 (970.4-1492.6) | 599.7 (448.6-760.2) | -2.41 (-2.71 - -2.12) | -2.43 (-2.69 - -2.17) |
| Ischemic stroke | 312924.1 (242944-390727.8) | 467599.5 (353053.8-593792.3) | 41.1 (31.9-51.5) | 23.9 (18-30.3) | 6934382.7 (5402568.9-8562897.3) | 9786327.1 (7426317.8-12266450.4) | 774.1 (603.2-958.6) | 460.6 (349.2-578) | -1.86 (-2.05 - -1.67) | -1.79 (-1.97 - -1.61) |
| Subarachnoid hemorrhage | 69804.5 (29601.8-107052.5) | 45525 (29051.7-68479.4) | 7.2 (2.9-11.1) | 2.1 (1.3-3.1) | 2105828 (1011306.8-3156096.9) | 1408915.6 (930530.8-2111718.8) | 190.8 (88.9-287.8) | 61.4 (40.5-91.8) | -4.51 (-4.76 - -4.25) | -4.08 (-4.28 - -3.88) |
| High SDI | 216406.2 (146854-297462.8) | 126324 (91404.8-165012.7) | 92.5 (62.3-127.7) | 24.1 (17.4-31.4) | 4725421.1 (3257809.6-6449438) | 2652597.1 (1958388.1-3443402.2) | 1867 (1281.9-2554.7) | 542.5 (400.8-704.2) | -3.96 (-4.21 - -3.71) | -4.03 (-4.27 - -3.79) |
| High-middle SDI | 448110.8 (347677.8-549342.8) | 479366.6 (364635.9-624031.9) | 236.2 (182.7-290.5) | 110 (83.8-142.4) | 10909532.3 (8496579.6-13337709.2) | 9996546.9 (7634929.6-13036962) | 4793.2 (3728.9-5867.2) | 2117.9 (1619.4-2753.6) | -3.84 (-4.38 - -3.29) | -3.84 (-4.42 - -3.25) |
| Middle SDI | 541928.1 (449972.9-639874.9) | 933789.5 (723900.8-1185215.1) | 246.8 (204.9-290.9) | 157.3 (122-199.3) | 14279507.3 (11868399.8-16859993.8) | 21399104.3 (16707492.9-27090312.5) | 5227.1 (4343.4-6167.5) | 3150.1 (2458.8-3985.6) | -1.41 (-1.64 - -1.19) | -1.22 (-1.48 - -0.96) |
| Low-middle SDI | 352665.5 (295211.8-412544.4) | 681306.2 (557332.1-804276.3) | 235 (196.4-275.6) | 201.8 (165-238.2) | 9744965.3 (8159894-11397087.8) | 17664078.8 (14422078.8-20809199.3) | 5487.1 (4598.4-6417.6) | 4537.6 (3706.7-5343.6) | -1.33 (-1.7 - -0.97) | -1.6 (-2 - -1.21) |
| Low SDI | 133989 (111685.2-157943.4) | 246006.8 (201833.2-290393.9) | 249.8 (207.5-294.4) | 215.9 (177.5-254.4) | 3649582 (3043756.1-4310042.3) | 6544054.4 (5345586.6-7729525.4) | 5671.8 (4730.7-6686.3) | 4696.1 (3850.6-5532.9) | -4.31 (-4.55 - -4.06) | -4.49 (-4.74 - -4.24) |
| Andean Latin America | 6872.8 (5271.9-8552.9) | 6721.9 (4515.3-9559.3) | 137.9 (106-171.4) | 45.9 (30.9-65.2) | 169777.6 (130192-211491.9) | 154332.1 (104105.7-219253.3) | 3005.5 (2310.4-3735.2) | 984.7 (664.6-1396.8) | -2.74 (-2.88 - -2.6) | -2.78 (-2.91 - -2.65) |
| Australasia | 1405.7 (46.8-3845.3) | 1494.8 (843.6-2235.9) | 27.7 (0.9-75.8) | 10.5 (5.9-15.7) | 29733 (985.2-81214.2) | 26879 (15327.4-40068.4) | 530.5 (17.6-1450) | 199.3 (113.7-297.5) | -0.9 (-0.98 - -0.82) | -1.04 (-1.12 - -0.95) |
| Caribbean | 8953.9 (5967.3-12884) | 12074.8 (7852.9-17007.6) | 139.6 (91.8-202.2) | 88.2 (57.3-124.3) | 212869.7 (145895.1-300739.3) | 286268.1 (187633.9-400798.2) | 3076.2 (2094.5-4363.5) | 2052.5 (1344.8-2875) | -1.6 (-1.99 - -1.21) | -1.85 (-2.19 - -1.51) |
| Central Asia | 24920 (15903.9-35475) | 33143.8 (24863.6-42031.4) | 292.9 (187-416.1) | 222.2 (166.5-281) | 618951 (394394.5-885311.1) | 801525.9 (601987.7-1016991.2) | 6064.5 (3867.6-8660.6) | 4337.6 (3256.9-5491.9) | -4.31 (-4.85 - -3.76) | -4.23 (-4.83 - -3.63) |
| Central Europe | 86558.3 (54956.5-118703.8) | 41471.3 (31412.9-55252.3) | 293.3 (186.1-401.7) | 84.1 (63.6-112) | 1973506.1 (1256390.3-2704347.1) | 811735.1 (616615.6-1080262.6) | 5850.8 (3722.7-8012.6) | 1607.1 (1219.4-2139.2) | -0.91 (-0.98 - -0.84) | -1.04 (-1.1 - -0.97) |
| Central Latin America | 20617.3 (13979.1-27340.2) | 29268.7 (20251-39989.4) | 107.1 (72.5-142.1) | 49.5 (34.2-67.6) | 497505.4 (337938.3-660311.1) | 650431.1 (450855.6-890523.6) | 2233 (1515.6-2966.4) | 1027.4 (712.4-1406.5) | -4.58 (-4.67 - -4.5) | -4.18 (-4.25 - -4.11) |
| Central Sub-Saharan Africa | 13106.3 (9791.3-16817.5) | 23320.8 (16683.4-31028.6) | 280 (210.1-356.5) | 225.2 (164.5-296.3) | 357065.1 (266655.3-460294.2) | 655304.3 (464033.2-875487.3) | 6185.1 (4650-7903.6) | 4779.3 (3456.6-6303.3) | -3.45 (-3.75 - -3.15) | -3.1 (-3.38 - -2.82) |
| East Asia | 511921.7 (393469.4-622378.6) | 850427 (629403.9-1123658.7) | 325.3 (256.3-390) | 189.3 (141.7-247.5) | 13031829.1 (9995966.5-15862617.2) | 17509703.5 (12910335-23131281.7) | 6207 (4828.6-7496.6) | 3358 (2494.2-4408.2) | -6.45 (-6.83 - -6.06) | -6.32 (-6.67 - -5.96) |
| Eastern Europe | 120084.3 (63633.7-176858.3) | 64809 (40559.8-96135.3) | 292.4 (155.9-430.2) | 98.4 (61.6-145.9) | 2898987.4 (1529884.1-4289590.5) | 1427069.1 (895301.5-2116977.4) | 5721.9 (3035.9-8456.5) | 2005 (1258-2972.9) | -2.69 (-3 - -2.38) | -2.92 (-3.23 - -2.6) |
| Eastern Sub-Saharan Africa | 41861.9 (34235.3-50025.5) | 69871.3 (56678.6-83247.3) | 239.6 (195.1-286.9) | 191.2 (155-226.9) | 1146796.8 (939836.2-1373229.4) | 1927028.6 (1558303.4-2301317.4) | 5470.4 (4480-6541.9) | 4213.2 (3420.3-5003.9) | -0.46 (-0.55 - -0.36) | -0.68 (-0.75 - -0.6) |
| High-income Asia Pacific | 18536.1 (5131.1-35699.3) | 20849.6 (12460.6-30033) | 46.1 (12.4-89.6) | 17 (10.2-24.4) | 441697.7 (127420.4-840618) | 410495.8 (250577.4-587466) | 946.7 (267.3-1812.1) | 386.3 (237.8-551.3) | -0.46 (-0.56 - -0.37) | -0.59 (-0.68 - -0.49) |
| High-income North America | 49609.1 (19517.7-87883.3) | 16934.6 (7951.1-27700.2) | 64.8 (25.6-115) | 10.4 (4.9-17.1) | 1043364.7 (413145.2-1838205.6) | 340915.4 (160388.9-555174.1) | 1300.8 (515.4-2293.2) | 215.9 (101.7-351.7) | -1.44 (-1.68 - -1.21) | -1.64 (-1.86 - -1.42) |
| North Africa and Middle East | 104355.9 (83002.2-125956.5) | 189231.6 (149299.3-231851) | 263.9 (209.9-318.1) | 173.1 (136.8-211) | 2730070.8 (2166624.9-3302275.1) | 4799206.4 (3778766.1-5917259.7) | 5721 (4548.2-6907.6) | 3672.3 (2899.7-4504.9) | -1.44 (-1.53 - -1.35) | -1.49 (-1.57 - -1.41) |
| Oceania | 2235 (1628.6-2938.5) | 4651.8 (3289.4-6245) | 314.1 (232.7-408.6) | 249.2 (177.5-332.4) | 67924.2 (49077.5-89835.7) | 139655.3 (98273.7-188164.3) | 7475.9 (5488-9785.1) | 5937.2 (4220.7-7938.6) | -0.7 (-0.73 - -0.67) | -0.7 (-0.74 - -0.66) |
| South Asia | 329246.5 (270639.5-392330.2) | 728670.2 (589691-879261.1) | 223.8 (183.4-266.9) | 206.8 (167.4-249.1) | 9359877.3 (7714058.8-11169289.2) | 18861823.6 (15221120.4-22773516.5) | 5320.4 (4385-6340.6) | 4633.8 (3743.9-5589) | -0.2 (-0.31 - -0.08) | -0.42 (-0.51 - -0.34) |
| Southeast Asia | 145778.6 (116829.1-173668.6) | 219334.7 (163011.4-285946.5) | 261 (208.3-312.1) | 150.7 (111.9-196.1) | 4037887.1 (3248489.8-4797457.2) | 5819415.4 (4328928.9-7598463.3) | 5930.4 (4762.7-7057.7) | 3394.2 (2525.4-4426.5) | -1.83 (-2.09 - -1.57) | -1.87 (-2.11 - -1.63) |
| Southern Latin America | 11360.5 (6155.4-17287.6) | 6266.2 (3660.2-9309.2) | 112.7 (61.3-171.5) | 30.8 (18-45.7) | 263488 (141977.4-401359.2) | 138535 (81372.6-205926.6) | 2364.2 (1276.6-3600.1) | 661.6 (388.7-983) | -4.08 (-4.32 - -3.84) | -4.1 (-4.33 - -3.87) |
| Southern Sub-Saharan Africa | 6323.5 (4758.8-8011.9) | 10337.4 (7751.9-13189.1) | 114.5 (86-144.9) | 92.4 (69.1-117.9) | 174896.3 (131968.1-222010.7) | 281699.3 (211330.3-360057) | 2591.5 (1959.7-3282.1) | 2049.6 (1538.1-2612.2) | -0.77 (-1.26 - -0.28) | -0.89 (-1.38 - -0.39) |
| Tropical Latin America | 26098.3 (14967.1-38676.9) | 18743.6 (10867.8-27274.7) | 129.2 (75-189.8) | 31.2 (18-45.4) | 682836.7 (387451.2-1019759.9) | 461462.3 (268008.2-673309.1) | 2830 (1619.3-4202.8) | 714.4 (414.7-1043.4) | -4.52 (-4.67 - -4.36) | -4.48 (-4.63 - -4.32) |
| Western Europe | 117743.1 (58061.9-186982.3) | 37851.1 (25894.5-50881.2) | 97.4 (48-155.1) | 15.2 (10.4-20.5) | 2390157.8 (1179584-3772799.5) | 661137.5 (455247.6-888453.6) | 1867.6 (921.7-2952.5) | 288.9 (198.7-388.4) | -6.17 (-6.37 - -5.98) | -6.2 (-6.38 - -6.02) |
| Western Sub-Saharan Africa | 47738.6 (37183.8-58591.3) | 83118 (65815-102201.1) | 239.2 (186.9-292.4) | 200.8 (159.8-246) | 1233083.2 (964597.5-1513892.9) | 2134422.2 (1690280.5-2617238.5) | 5090.7 (3993.5-6228.8) | 4184 (3329.8-5122.2) | -0.64 (-0.76 - -0.52) | -0.72 (-0.85 - -0.59) |

Supplementary Table 6 The Deaths and DALYs of CVDs Burden Attributable to PM2.5 Pollution in 1990 and 2021in Females

|  | Numbers of Deaths (95%UI) | | Age-standardized rate (95%UI) | | Numbers of DALYs (95%UI) | | Age-standardized rate (95%UI) | | Estimated annual percentage changes (95%CI) | |
| --- | --- | --- | --- | --- | --- | --- | --- | --- | --- | --- |
|  | 1990 | 2021 | 1990 | 2021 | 1990 | 2021 | 1990 | 2021 | ASMR | ASDR |
| Cardiovascular diseases | 1630076.9 (1332136-1950520.5) | 2013903.9 (1592079.2-2443682.7) | 148.6 (120.5-178.7) | 78.3 (61.9-95) | 35510624.2 (29488729.4-42043295.2) | 41338792 (33329848.8-49592790.8) | 3056.3 (2528.1-3627.9) | 1631.1 (1315.8-1956.4) | -2.21 (-2.33 - -2.08) | -2.18 (-2.29 - -2.06) |
| Ischemic heart disease | 741051.3 (552172.2-942908) | 1079981.7 (795496.7-1357633.8) | 68.8 (50.9-88.1) | 42 (30.9-52.7) | 15248620.7 (11548463.4-19187795.1) | 21181115 (15814478.4-26498427.1) | 1325.2 (999.2-1672.6) | 836.1 (624.6-1045.8) | -1.65 (-1.74 - -1.57) | -1.57 (-1.65 - -1.49) |
| Stroke | 889025.6 (706542.6-1088990.3) | 933922.3 (717406.7-1180537.2) | 79.8 (63.2-98) | 36.3 (27.9-45.9) | 20262003.5 (16245050.2-24558599.6) | 20157677 (15662624.4-25168714.6) | 1731 (1385.6-2101.3) | 795.1 (617.9-992.4) | -2.75 (-2.93 - -2.57) | -2.72 (-2.89 - -2.56) |
| Intracerebral hemorrhage | 449170.8 (358474-552816.1) | 453010.9 (342430.7-572708.5) | 39 (31.1-48.1) | 17.7 (13.4-22.4) | 11069237.1 (8867599-13557964.7) | 10406094.5 (7934153.9-13057513.7) | 930.3 (744.9-1140.1) | 412.6 (314.6-517.6) | -2.72 (-2.96 - -2.47) | -2.79 (-3.01 - -2.58) |
| Ischemic stroke | 368258.8 (285359.4-463852.6) | 438002.8 (332922.8-562792.1) | 34.6 (26.6-43.7) | 16.9 (12.9-21.7) | 7206969.1 (5677470.4-8947883.4) | 8509025 (6566894.9-10784992.7) | 635.9 (499.2-791.4) | 332.3 (256.5-421.2) | -2.52 (-2.67 - -2.36) | -2.31 (-2.45 - -2.16) |
| Subarachnoid hemorrhage | 71596 (44156.6-98242) | 42908.5 (31632-58435.1) | 6.2 (3.8-8.5) | 1.7 (1.3-2.3) | 1985797.2 (1319990.9-2662746.6) | 1242557.5 (940888-1668941.4) | 164.9 (109.2-221.4) | 50.2 (38-67.4) | -4.8 (-5.1 - -4.48) | -4.36 (-4.61 - -4.1) |
| High SDI | 229795.3 (153706-320342.6) | 111253 (76252.3-149344.7) | 58.9 (39.4-82) | 13.7 (9.6-18.1) | 3805943.7 (2615429.3-5225263) | 1766791.8 (1254075.6-2326421.7) | 1032.2 (711-1411) | 267.7 (193.4-348.6) | -5.03 (-5.15 - -4.9) | -4.66 (-4.78 - -4.55) |
| High-middle SDI | 492225.5 (370049.2-624839.1) | 443129 (332595.5-570790.1) | 173.4 (129.2-221.1) | 69.3 (52-89.2) | 9527451.3 (7305401.7-11969378.8) | 7521668.8 (5755741.9-9644301.8) | 3172.3 (2422.2-3995.4) | 1222.5 (936.5-1567.4) | -3.34 (-3.64 - -3.03) | -3.47 (-3.8 - -3.15) |
| Middle SDI | 498276.4 (414667.1-598086.3) | 713903.3 (544982.6-886462.9) | 207.2 (171.4-248.8) | 99.6 (75.7-123.9) | 11657160.7 (9741083-13917341) | 14418146.8 (11187729.2-17732535) | 4145.7 (3453.7-4952.2) | 1900.3 (1470.3-2339.5) | -2.39 (-2.57 - -2.21) | -2.58 (-2.73 - -2.43) |
| Low-middle SDI | 296310.9 (244161.1-348597.6) | 545606.7 (443097.6-644449.6) | 210.9 (173.4-248.4) | 147.7 (119.6-174.4) | 7584872.6 (6257275.4-8940045.7) | 12716280.4 (10367477.3-15056163.8) | 4572.9 (3775.2-5382.1) | 3108.6 (2532.2-3676) | -1.09 (-1.17 - -1.02) | -1.21 (-1.28 - -1.14) |
| Low SDI | 111200.5 (89176.6-135212.9) | 198239.7 (160813.7-236733.6) | 220.7 (176.6-266.8) | 169.5 (136.9-202.5) | 2890820.9 (2320281.9-3540791.1) | 4881449.5 (3967478.6-5827805.5) | 4779.3 (3841.4-5813.9) | 3501.9 (2845.3-4173.5) | -0.84 (-0.93 - -0.74) | -1.05 (-1.12 - -0.97) |
| Andean Latin America | 6303.1 (4897.7-7896.4) | 6036.4 (4105.1-8605.9) | 119 (92.6-148.7) | 35.8 (24.3-51) | 142456.1 (110359.8-179316.9) | 119877.6 (82186.6-170255.1) | 2410 (1872.9-3024.3) | 698 (478.9-990.8) | -4.48 (-4.75 - -4.2) | -4.61 (-4.86 - -4.36) |
| Australasia | 1323.4 (47.5-3656.4) | 1296.4 (735.8-1971.6) | 17.3 (0.6-47.8) | 6.1 (3.5-9.3) | 21078.4 (751.2-58201.3) | 17597 (10099.5-26500.7) | 281.3 (10-775.9) | 96.5 (55.6-144.8) | -3.98 (-4.46 - -3.5) | -4.1 (-4.63 - -3.56) |
| Caribbean | 9342.5 (6628.5-12777.5) | 12176.8 (8256.6-16687.1) | 134.2 (94.6-184.1) | 74.6 (50.9-102) | 207954.2 (150402.3-279836) | 264400.6 (182272.5-360739.3) | 2809.7 (2025.4-3788.6) | 1686.3 (1165.1-2300.1) | -1.78 (-1.94 - -1.61) | -1.55 (-1.71 - -1.38) |
| Central Asia | 29632.1 (19117.5-41241.7) | 32455.3 (24151.8-41024.9) | 204.5 (131.4-285) | 148.8 (110.5-188.2) | 576949 (375489.1-802813.5) | 629620.7 (472621.1-793258.2) | 3840.3 (2493.9-5347.8) | 2666.9 (1999.5-3362.4) | -1.61 (-1.96 - -1.27) | -1.86 (-2.24 - -1.49) |
| Central Europe | 90733 (58896.6-121925.8) | 48697.3 (36170.3-66487.7) | 203 (131.6-272.8) | 56.7 (42.1-77.4) | 1598939.9 (1038329.9-2148188.7) | 710294.8 (530646.3-971729.3) | 3454.9 (2242.6-4640.8) | 904.6 (675.9-1239) | -4.51 (-4.78 - -4.25) | -4.78 (-5.05 - -4.5) |
| Central Latin America | 19595.5 (13746.8-25663.9) | 25714.7 (17888.7-34523.7) | 99.3 (69.5-130.5) | 35.3 (24.6-47.4) | 422411.3 (299542.3-551296) | 485346.3 (341941.3-650560.4) | 1841.5 (1301-2410.1) | 654.4 (461-876.8) | -3.46 (-3.62 - -3.31) | -3.55 (-3.69 - -3.4) |
| Central Sub-Saharan Africa | 11177.7 (8270.8-14798.9) | 22002.2 (15068-30372) | 234.4 (174.8-305.5) | 180 (123.9-247.5) | 290503.1 (214273.7-388475.7) | 527334.4 (363328.5-728271.5) | 4816.3 (3596.2-6331) | 3539.4 (2456.4-4855.4) | -1 (-1.1 - -0.91) | -1.15 (-1.25 - -1.04) |
| East Asia | 483713.4 (384510.7-599975.5) | 665682.3 (489615.6-854315.2) | 250.5 (198.9-309.4) | 112 (82.1-143.7) | 11056500.7 (8823578-13727126.1) | 12056520.9 (9004451.2-15425613.4) | 4832.2 (3855.1-5979.2) | 1961.8 (1462.4-2510.3) | -2.52 (-2.87 - -2.17) | -2.88 (-3.18 - -2.57) |
| Eastern Europe | 190459.3 (101156.6-280355.7) | 88664.3 (55485.5-130849) | 198.1 (105-291.4) | 64.1 (40.2-94.7) | 3254507.6 (1731498.4-4800805.6) | 1370437.7 (862774.3-2025323.4) | 3270.6 (1737.9-4824.1) | 1055.7 (665.5-1561.4) | -4.5 (-5.02 - -3.98) | -4.58 (-5.13 - -4.03) |
| Eastern Sub-Saharan Africa | 32546.2 (25174.4-41305.7) | 55829.7 (43619.8-69158.5) | 192.9 (148.1-244.2) | 142.6 (109.5-177.9) | 857140.1 (667785.2-1092138.7) | 1373518.3 (1095324-1681488) | 4208.2 (3274.2-5322.1) | 2928.9 (2316-3598) | -1.1 (-1.15 - -1.04) | -1.34 (-1.4 - -1.27) |
| High-income Asia Pacific | 19714 (5691.3-38027.7) | 20951 (11587.4-31507.1) | 33 (9.4-63.8) | 9 (5.2-13.3) | 366881.2 (110300.9-697335) | 300039.9 (175877.4-439712.5) | 596.6 (178.5-1135.7) | 178.5 (108.4-258) | -4.48 (-4.85 - -4.11) | -4.23 (-4.59 - -3.86) |
| High-income North America | 49883.2 (19967.5-87774) | 14572.6 (6787.4-24013.6) | 38.5 (15.4-67.7) | 6.1 (2.9-10) | 800156.6 (325842.2-1396797.3) | 234322.8 (110892.8-381929.3) | 673.5 (274.9-1174.5) | 113.8 (54.2-184.7) | -6.48 (-6.94 - -6.02) | -6.26 (-6.7 - -5.83) |
| North Africa and Middle East | 87357.1 (70475.1-104346) | 150600.4 (117550.8-183303.4) | 235.8 (189.1-281.6) | 148.6 (116-180.2) | 2044424.7 (1655790.5-2449370.6) | 3333754.4 (2603087-4077784.4) | 4636.8 (3746.8-5543.4) | 2814.5 (2201.3-3426.9) | -1.41 (-1.48 - -1.34) | -1.61 (-1.68 - -1.55) |
| Oceania | 1694.2 (1210.4-2282.7) | 3483.7 (2524.8-4605.5) | 271.8 (198.8-356.7) | 214 (157.8-277.9) | 47770.3 (33503.7-65775.2) | 95633.9 (68265.8-128519.4) | 6009.4 (4330.1-8048.3) | 4679.3 (3414.5-6163.7) | -0.81 (-0.9 - -0.72) | -0.85 (-0.93 - -0.77) |
| South Asia | 233773.3 (185814-279957.1) | 521634.3 (420379.5-624820.5) | 181.8 (144.1-218.6) | 138.9 (111.6-166.6) | 6243393 (4978438.2-7470562.8) | 12401849.2 (10040555.6-14860839.6) | 4063.8 (3243-4862.8) | 2988.5 (2416.4-3579) | -0.79 (-0.95 - -0.63) | -0.93 (-1.08 - -0.79) |
| Southeast Asia | 146630.8 (117867.6-175261) | 195143.6 (143281.9-252667.9) | 230.5 (184.3-276.1) | 115.1 (84.4-148.6) | 3665088.3 (2969629-4368167.5) | 4383347.2 (3234715-5708281.8) | 4921.8 (3977.3-5866.7) | 2328.1 (1718.1-3023.8) | -2.36 (-2.63 - -2.09) | -2.55 (-2.79 - -2.3) |
| Southern Latin America | 10274 (5671.5-15498.4) | 5562 (3167.2-8361.8) | 76 (41.9-114.7) | 18.1 (10.3-27.2) | 191435.7 (105468.4-287854.1) | 95052.6 (54623.9-142216) | 1376.9 (758.3-2071.2) | 338.5 (195.1-506) | -4.5 (-4.75 - -4.24) | -4.48 (-4.74 - -4.22) |
| Southern Sub-Saharan Africa | 6870.4 (5132.2-8693.4) | 12840.7 (9527.7-16302.8) | 91.3 (67.8-115.7) | 81 (59.7-103.1) | 164050.3 (124723.1-205897.7) | 285347.4 (213336.4-362641.8) | 1943.4 (1472.2-2439.9) | 1608.2 (1198.8-2043.4) | -0.15 (-0.68 - 0.37) | -0.27 (-0.76 - 0.22) |
| Tropical Latin America | 22212.3 (13469-31824.7) | 16036.3 (9441-23199.1) | 97.1 (58.9-138.9) | 20.3 (12-29.4) | 510654.9 (310565.6-732619.3) | 337300.3 (200636.4-486234.5) | 1936.6 (1178.5-2774.1) | 430.8 (256.4-620.9) | -5.1 (-5.24 - -4.96) | -5.04 (-5.2 - -4.88) |
| Western Europe | 129655.2 (64140.1-210561.9) | 36601.8 (23975.1-50492.5) | 59.6 (29.4-96.9) | 8.5 (5.7-11.7) | 1971142.6 (978590.4-3177274.2) | 483305.6 (324646.6-661636) | 963.8 (477.9-1552) | 137 (93.2-186.9) | -6.45 (-6.69 - -6.21) | -6.49 (-6.7 - -6.28) |
| Western Sub-Saharan Africa | 47186.3 (36741.2-57869.8) | 77922.1 (61303.2-96888.1) | 233.3 (181.4-285.6) | 177.6 (140.6-218.2) | 1077186.3 (841241.3-1325623) | 1833890.3 (1443420.2-2298623.7) | 4702.7 (3672-5769.1) | 3440.2 (2727.7-4263.3) | -0.86 (-0.93 - -0.79) | -1 (-1.08 - -0.93) |

Supplementary Table 7 DALYs of CVDs Attributable to PM2.5 Pollution by Age Distribution and 204 Countries and Territories, 2021

| Location | 25-29 years | 30-34 years | 35-39 years | 40-44 years | 45-49 years | 50-54 years | 55-59 years | 60-64 years | 65-69 years | 70-74 years | 75-79 years | 80-84 years | 85-89 years | 90-94 years | 95+ years |
| --- | --- | --- | --- | --- | --- | --- | --- | --- | --- | --- | --- | --- | --- | --- | --- |
| Afghanistan | 636 (452.6-921.3) | 991.5 (691.8-1427.8) | 1623.9 (1133.9-2352.1) | 3091.8 (2148.4-4582.8) | 5227.1 (3653.9-7515.1) | 7892.1 (5524.8-10894.2) | 10695.9 (7569.9-14174) | 14401.1 (10751.4-18614.5) | 19349 (14664.1-24508.7) | 25059.8 (19849.4-31441.2) | 30785.1 (24007.9-37881.2) | 35058.1 (27322.9-43742.6) | 41493.5 (31067.1-51559.2) | 46172.5 (33734.7-58754.1) | 40310.1 (27019.9-54206.4) |
| Albania | 45.3 (27.3-69.4) | 90.1 (57.6-141.3) | 172 (102.2-264.2) | 258 (162.6-400.4) | 413.3 (259.7-643.1) | 625.5 (386.9-983.8) | 1072.8 (645.2-1647.1) | 1649 (1034.1-2523.9) | 2529.2 (1553.7-3880) | 4134.8 (2622-6224.9) | 8039.7 (5176.2-12163.1) | 13322.3 (8382.3-20033.4) | 19718.7 (12299-29804.7) | 27022 (16942.1-40282.8) | 36926.6 (22546.7-54566.7) |
| Algeria | 115.3 (71.3-167.4) | 189 (118.3-266.1) | 309.5 (197-452.4) | 521 (341.8-738.6) | 817.2 (536.5-1153.4) | 1247.1 (815.9-1785.5) | 1737.6 (1155.2-2468.7) | 2635.2 (1737-3656.4) | 3424 (2212.6-4682.8) | 4646.3 (3018.4-6487.5) | 7199.5 (4740.5-10122.5) | 9929.4 (6466.2-13410.3) | 33111.1 (22700-45761.7) | 43041.2 (29470.3-58875) | 35188 (22894.7-46757.8) |
| American Samoa | 56.9 (6.2-138.7) | 115.4 (12-278.1) | 179.9 (17.9-431.3) | 323 (30.3-755.5) | 483.5 (48.7-1131.4) | 660.5 (63.4-1549.4) | 808.6 (85.1-1836.4) | 1057.1 (114.5-2411.2) | 1280.8 (131.7-2975.3) | 1586.2 (166.4-3596.4) | 2016.4 (215.3-4611) | 2444.8 (246.5-5427.6) | 3297.3 (348.1-7361.9) | 4025.5 (428.2-8914.8) | 5802.8 (580.1-13402.6) |
| Andorra | 5.5 (3-8.9) | 9.2 (4.9-15.1) | 16.1 (8.3-27.6) | 28.6 (13.6-48.8) | 50.3 (25.1-83.4) | 80.6 (40.7-144.4) | 116.9 (58.5-197.2) | 170.7 (83.9-279.5) | 248.2 (129.3-422.6) | 382.2 (197.8-633.1) | 586.2 (309.2-945.7) | 872.3 (450-1440.5) | 1352.9 (695.5-2181.3) | 1991.1 (976.6-3169) | 2763 (1380.5-4637) |
| Angola | 101 (61.1-146.7) | 197.4 (123.5-284) | 328.3 (206.2-487.7) | 699.2 (414.1-1002.5) | 1279.2 (776.2-1876.5) | 2160.2 (1366.6-3063.5) | 3413.6 (2143.4-4890.5) | 5394.8 (3441.5-7511.7) | 7987 (5146.4-10780.1) | 11045.1 (7316.5-15125.9) | 13589.8 (9297.4-18244.3) | 16155.3 (10836.5-21932.5) | 17018.6 (11076.6-23151) | 18941.8 (12299.9-26011.3) | 18247 (10748.1-26091.7) |
| Antigua and Barbuda | 23.7 (9.3-41.9) | 33.4 (12.5-58.5) | 56.4 (22.5-99.4) | 121.3 (45.7-215.4) | 248.1 (88.2-444.7) | 470.4 (167-837) | 773.3 (282-1376) | 1199.9 (429.1-2122.9) | 1933.8 (696.2-3459.9) | 2915.2 (1073.4-5366.8) | 3777.2 (1357.2-6750.1) | 4582.9 (1646.6-8224.7) | 5844.7 (2091.2-10225.9) | 7737.4 (2896.2-13984.7) | 11153.7 (4208.2-19503.9) |
| Argentina | 30.4 (15.8-48.7) | 44.7 (22.9-71.1) | 72.9 (37.7-115.6) | 126.2 (63.8-197) | 222.5 (117-350.5) | 375.4 (195.4-590.7) | 562 (293.2-889.9) | 817.7 (419.3-1292.3) | 1096.7 (569.6-1768.4) | 1396.6 (721-2222.5) | 1775.4 (926.7-2797.3) | 2245.9 (1165.3-3606.9) | 3273.8 (1710.8-5301) | 4383 (2220.2-7049.6) | 5579.5 (2734.9-9168.3) |
| Armenia | 81.4 (57.2-109.8) | 157.6 (109.9-210.3) | 300.8 (210.3-406.4) | 648.1 (450-886.2) | 1118.9 (778.6-1516.5) | 1751.9 (1220.9-2359.6) | 2715.8 (1896.3-3644.1) | 3687.7 (2570.4-4923.5) | 5440.5 (3764.8-7338.4) | 8048.7 (5588.2-10811.7) | 12174.2 (8550.5-16033.7) | 16525.1 (11378.5-21823.5) | 21460.1 (14595.8-28611.5) | 27732.9 (18473.7-37026.8) | 36750.3 (24022.9-49794.1) |
| Australia | 5.6 (3.3-8.1) | 11.4 (6.7-16.8) | 23.5 (14.1-34.8) | 44 (25.4-64.7) | 75.5 (45-112.6) | 103.9 (61-151.8) | 141.6 (83.7-205.8) | 188.1 (109.8-279.5) | 245 (147.2-358.9) | 355.4 (211.3-515.5) | 555.6 (326.1-812.9) | 890.6 (523.4-1313.6) | 1563.1 (919.5-2291.5) | 2781.7 (1620.3-4107.5) | 4940.7 (2833.8-7437.8) |
| Austria | 7.4 (5.1-10.3) | 13.6 (9.5-18.5) | 26.7 (18.4-36.3) | 43.2 (29.5-59.2) | 84.9 (59.9-114.7) | 143.2 (100.9-191.1) | 238.5 (168.1-321.5) | 380.1 (266.6-508.6) | 527.1 (370.5-697.7) | 817.5 (562.8-1097.9) | 1286.8 (898.5-1730.8) | 1741.3 (1207-2368.6) | 3224.4 (2188.9-4356) | 4819.1 (3212.7-6572.1) | 7258.5 (4741.5-10216.3) |
| Azerbaijan | 88.2 (45.8-147.2) | 168.3 (86.7-270.6) | 270.6 (140.4-430.2) | 486.4 (252.3-769.2) | 880.6 (460.1-1384.7) | 1472.6 (771.2-2314.9) | 2450.4 (1281.1-3846.3) | 4199.6 (2240.8-6507.9) | 6640.3 (3518.8-10355.3) | 10614.8 (5710.2-16372.3) | 13997 (7685.2-21401.7) | 19424.7 (10693.6-29937.4) | 20870.4 (11418.2-32079.1) | 23481.4 (12631.2-35446.8) | 26674.4 (13723-40718.9) |
| Bahamas | 46.1 (16-94.5) | 95.8 (32.6-191.4) | 167 (55.5-327.8) | 317.7 (112.8-620.3) | 479.4 (164.7-945.8) | 832.5 (295.8-1617) | 1077 (385.7-2057.4) | 1353.7 (497.2-2661.9) | 1846.3 (667.9-3504.6) | 2231.4 (793.6-4329.1) | 2848.9 (1010.2-5441.7) | 3937.4 (1411.3-7508.5) | 4813.4 (1735.4-9168.1) | 6286.7 (2264.3-12171.9) | 7585.9 (2690.2-14433.5) |
| Bahrain | 212.2 (155.1-278.4) | 311.2 (233.8-406.6) | 384.6 (292.5-496.4) | 591.5 (450.3-761.3) | 946.7 (689.9-1230.4) | 1455.8 (1089.6-1847.7) | 2303.9 (1730.5-2941.2) | 3330 (2467-4300.5) | 5170.8 (3882.2-6708.4) | 9090.1 (6903.7-11481.3) | 15893.1 (12123.3-19758.3) | 21985.7 (17276.2-26890.2) | 29527.1 (22703.6-36162.9) | 38658.6 (30733.4-47243.5) | 52236.8 (39168.3-64625) |
| Bangladesh | 323.6 (226.8-446.5) | 611.9 (436.7-805.2) | 839.8 (591.1-1109.1) | 1395.7 (968.5-1886.1) | 2244.5 (1562.7-2911.2) | 3915.9 (2860.9-5138.7) | 4745.9 (3360.6-6262) | 7740.7 (5622.1-10013.3) | 10145.7 (7521.1-12974.3) | 12702.6 (9755.6-16040.5) | 14779.2 (11247.6-18484.2) | 24232 (18677.5-29387.7) | 28218.6 (22011.2-34318.1) | 34122.9 (25886.5-41784) | 42274 (31338.9-51883.5) |
| Barbados | 35.1 (14.8-60.6) | 69.3 (29.1-122) | 107 (46.5-186.3) | 198.5 (82.8-343.1) | 364 (153.1-650.6) | 532.9 (222.7-932.8) | 856.7 (352-1499.1) | 1279 (535.6-2214.6) | 1939.5 (830.8-3336) | 2871.1 (1176.5-4934.6) | 3743.3 (1545.3-6390.4) | 4947.5 (2089.6-8309.2) | 7804.9 (3249.6-13329.3) | 10403 (4439.7-17810.7) | 13844 (5912.5-23389.2) |
| Belarus | 51.4 (34.9-70.1) | 141.7 (96.8-200.9) | 278.2 (189-386.1) | 549.4 (371.6-771.7) | 975 (653.5-1372.1) | 1487.2 (997.3-2071.1) | 2285.1 (1528.4-3191.8) | 3559.2 (2412.9-4919.5) | 4967.2 (3375.8-6863.9) | 6687.5 (4571.7-9214.3) | 9408.6 (6465.7-12957.7) | 12550 (8679.5-17237.9) | 16152.6 (10984.1-22398.3) | 22144.2 (15141.8-30520.2) | 29227.2 (19863.5-40577) |
| Belgium | 6.5 (4.4-8.8) | 12.1 (8.3-16.5) | 22.9 (15.8-31.3) | 46.6 (31.9-62.1) | 80.5 (55.9-107.1) | 125.8 (87.5-171.7) | 183.7 (127.6-242.2) | 275.5 (187.4-369.3) | 400.6 (279.2-532.9) | 587.4 (411.5-782.7) | 852.9 (587.1-1118.5) | 1211.7 (816.5-1633.6) | 1807.3 (1200.7-2439.4) | 2595 (1688.9-3561.3) | 3852.6 (2376.9-5354.2) |
| Belize | 71.3 (36.1-113.3) | 90.6 (44.1-143.1) | 182.3 (92.6-289.1) | 348 (174.5-558.5) | 583.7 (291.9-951.8) | 822.1 (423.5-1304.7) | 1327.7 (684.6-2109.7) | 2226.1 (1103.4-3551.5) | 2302.4 (1145.1-3712.6) | 3245 (1620.6-5282.5) | 4442.3 (2170.2-7172.8) | 5386.6 (2648.4-8644.1) | 7116.5 (3527.2-11384.2) | 9203.8 (4548.1-14640.2) | 12626.3 (6137.4-20011.1) |
| Benin | 140.7 (98.1-193.6) | 268.9 (191.3-366.2) | 449.5 (314-585.6) | 850.6 (600.9-1133.5) | 1527.9 (1082.6-2040.3) | 2828 (2051.7-3812.2) | 4158.3 (2959.3-5463.3) | 6802.6 (5022-8866.1) | 10247.6 (7790.1-13186) | 13816.8 (10603.8-17609.7) | 18352.8 (14549.4-22477.6) | 22025.4 (17521.2-27381.3) | 24805.9 (19737.2-29733.4) | 29819.4 (23258.4-37020.8) | 36271.7 (26599-46231.2) |
| Bermuda | 5.2 (1-10.3) | 7.8 (1.4-15.2) | 14.5 (2.6-28.2) | 30.6 (5.8-58.4) | 66.9 (12.3-126.9) | 125.3 (23.1-240.8) | 168.7 (30.3-328.7) | 215.9 (40.1-418.6) | 280.2 (51.7-536.6) | 440.9 (82.3-847.1) | 579.8 (105.8-1111.8) | 794.8 (149.1-1539.3) | 1220.6 (225.5-2330.4) | 1844.2 (350-3577.3) | 2472.6 (467.3-4868.5) |
| Bhutan | 120.6 (66.9-184.4) | 220.4 (124.9-337.6) | 319.7 (191.1-476) | 574.5 (357.2-887.4) | 945 (607.1-1377) | 1533.9 (1032.6-2173.2) | 2346.9 (1516.6-3305.1) | 3334.3 (2284.5-4723.9) | 4784.1 (3227.2-6468.2) | 6456.7 (4560.4-8529.3) | 8342.4 (5860.7-10677.6) | 9821.9 (7062.2-13077.3) | 11178.6 (8228-14386) | 14268.2 (9991.1-18489.4) | 16952.6 (11336.1-23061.7) |
| Bolivia (Plurinational State of) | 115 (68.7-188.9) | 161.2 (103.3-247.5) | 230.1 (145.4-348) | 375.6 (226.4-579.3) | 612.6 (372.1-998.9) | 1006.5 (603.9-1618.9) | 1479 (919.8-2360.8) | 2162.8 (1299.3-3547.1) | 3145.3 (1950.2-5041.7) | 4500.6 (2834.2-7094.2) | 6131.1 (3913.1-9664.3) | 8239.1 (5329.8-12629.8) | 10188.1 (6606.6-14608.6) | 12031.2 (7863.9-17263.1) | 10472.7 (6892.5-15457.2) |
| Bosnia and Herzegovina | 88.7 (60.4-131.1) | 114.6 (75.9-171.5) | 212.1 (137-306.4) | 346.3 (217.3-495.7) | 673.3 (440.7-963.3) | 1229 (847.6-1710.1) | 1960.5 (1348.4-2790.2) | 3126.6 (2150.9-4375.9) | 4588.2 (3260.1-6359.1) | 7339.9 (5103.8-10096.8) | 12369.6 (8726.8-16981.2) | 16690.9 (11662-22657) | 20902.2 (14304.7-28268.7) | 23628.4 (16126.4-32417.5) | 21637.8 (13221.5-30621.7) |
| Botswana | 42.2 (17.4-81.2) | 75.4 (34.2-135.9) | 148.7 (69.7-259.1) | 297.7 (149.2-514.1) | 534 (278-922.9) | 914.6 (473.5-1591.6) | 1374 (726.8-2319.9) | 2199.8 (1189.6-3820.9) | 3265.9 (1834.2-5335.3) | 4660.9 (2674.3-7871.8) | 6237.8 (3471.1-10505.3) | 7890 (4529-13104.1) | 9253.8 (5292.2-15781.8) | 10198.9 (5566.4-17616.9) | 7216.6 (3534.7-13039.3) |
| Brazil | 42.8 (25.4-61.8) | 72.4 (43.1-105.4) | 126.4 (75.4-183.5) | 220.4 (132-316.8) | 363.3 (218.4-531.3) | 549.9 (327.2-797.5) | 754 (440.3-1089.1) | 1009.1 (587.3-1451.3) | 1280.7 (737.3-1854.3) | 1582.1 (919.8-2280.9) | 1951.3 (1147.4-2835.1) | 2268.7 (1324.4-3316.1) | 2737.3 (1520.6-4020.4) | 3290.7 (1843.4-4881.8) | 3867.8 (2152.8-5809.4) |
| Brunei Darussalam | 22.5 (5.1-46.4) | 41.8 (8.3-85.4) | 85 (17.8-170.9) | 112.1 (23.3-225.4) | 201.7 (42.6-403.8) | 276.5 (55.2-563.5) | 307.9 (64.8-621.2) | 412.4 (87-837.7) | 488 (101.4-967.5) | 661.2 (138.4-1320.9) | 1179 (245.2-2337.2) | 1497 (305.9-2995.4) | 1886.8 (415.9-3729.5) | 2466 (519.4-4808.1) | 6379.1 (1384.3-12692.9) |
| Bulgaria | 114.4 (83.6-165.3) | 185.5 (134.3-271) | 331.1 (242.4-481.3) | 552.6 (398.5-799) | 1031 (740.4-1506.5) | 1592.8 (1160.5-2314.9) | 2393.8 (1738.7-3462.8) | 3295.1 (2387.1-4797.4) | 4305.3 (3143.8-6263.9) | 5873.9 (4330.7-8589.8) | 7894.3 (5908.2-11431.6) | 12136.6 (9115.9-17706.5) | 17861.2 (13379.8-25826) | 28538.2 (21315.3-41215.1) | 46575.7 (34969.4-68324.8) |
| Burkina Faso | 121.4 (83.5-174.1) | 247.2 (169.7-345.9) | 422.4 (299-585.4) | 865.6 (606.7-1214.8) | 1546.4 (1056.2-2131.8) | 2792.2 (1961.7-3796) | 4028.9 (2887.1-5423.8) | 6335.9 (4544.9-8447.1) | 9504.1 (7067.2-12468) | 12864.9 (9690.9-16583.7) | 16151.5 (12598.7-20286.7) | 18421.7 (14194.1-22645.4) | 20555.4 (16100.9-25473.2) | 25329.7 (19445-32162.7) | 31124 (22677.6-39800.1) |
| Burundi | 257.2 (175-354.7) | 437.6 (301.6-597.2) | 648 (448.5-866) | 1263 (898.9-1704.8) | 2041.1 (1447.6-2761.6) | 3231.2 (2302.7-4267.1) | 5216.9 (3677.5-6914.8) | 8241.4 (6145.8-10718.7) | 11441.6 (8639.9-14489.3) | 14584.7 (11048.8-18794.4) | 17814.8 (13374.9-22603) | 19739.4 (14997.8-25731.3) | 21020.6 (15687.4-27715) | 24404.3 (17985.7-32794) | 27097.9 (19163.6-35884.1) |
| Cabo Verde | 99.6 (64.9-150.4) | 175.7 (111-262.7) | 294.8 (184.2-432.7) | 599.4 (381.3-880.9) | 1098 (735.3-1542.6) | 1939.5 (1316.6-2768.6) | 2764.7 (1871.8-3823.3) | 4217.7 (2876.3-5725.3) | 6085.1 (4273.5-8151.6) | 8813.6 (6253.9-11728.7) | 12073.6 (8745.9-15918.4) | 16104.9 (11929.1-21193.4) | 20349.2 (14810.5-26455.7) | 24780.9 (18176.2-32056.8) | 28878.7 (20357-37128.8) |
| Cambodia | 235.3 (159.2-352.6) | 399.5 (272.1-587.6) | 676.9 (468.9-959.7) | 1218.7 (847-1705.3) | 2253.5 (1549.7-3082.2) | 3772.5 (2672.3-5013.8) | 5533.8 (3989.5-7479.7) | 7742.6 (5561.4-10294.2) | 10663.5 (7922.3-13730.6) | 15345.5 (11787.7-19266.8) | 20600.8 (15760.6-25649.4) | 25768.7 (19869.5-31596.2) | 30825.7 (23958.8-37734.4) | 36596.5 (28334.5-44755.8) | 43157.7 (32452.3-54502) |
| Cameroon | 192.5 (124.6-279) | 362.6 (239.8-522.1) | 586.3 (393.3-811.6) | 1138.6 (753.5-1692.8) | 1930.4 (1223.8-2841.5) | 3349.4 (2216.8-4804.1) | 4765 (3185.2-6856.4) | 7518.2 (5109.5-11044.5) | 10795.9 (7362.9-14649.6) | 14223.9 (10253.1-20131.7) | 17862 (13699.5-23941) | 21061.6 (16208.9-27535.6) | 23824.5 (18532.4-31835.6) | 28805.3 (22253.6-37998.5) | 34260.9 (24442.8-43949.2) |
| Canada | 3.9 (1.5-7.2) | 6.9 (2.5-12.4) | 11.9 (4.4-21.5) | 20.4 (7.5-37) | 34 (12.4-61.8) | 55.1 (20.4-97.8) | 80.2 (29.4-145) | 112.7 (40.1-200.8) | 148.7 (52.7-263.7) | 200.8 (72.2-362.5) | 284.3 (103.4-502.5) | 423 (150-753.8) | 720.5 (256.5-1307.4) | 1139.8 (403.6-2091.3) | 2172.2 (768.2-3901.7) |
| Central African Republic | 219.3 (138.6-327.9) | 448.8 (274.1-682.6) | 821.5 (500.6-1257.5) | 1892.4 (1125.1-3076.9) | 3628.2 (2233.7-5623.2) | 5808.9 (3691.5-8915.4) | 8851.9 (5956.7-12853.1) | 12639 (8872.7-17520.9) | 17296.4 (12587-23676.9) | 22319.1 (17179.8-29032.6) | 26586.6 (19852.2-34319.3) | 29741.8 (21959.1-38374.6) | 30124.5 (21368.9-40906.7) | 29831.3 (21166.5-40380.1) | 22309.3 (14331.1-32025.9) |
| Chad | 213.1 (146.4-301.2) | 388.1 (273-542.3) | 618.4 (431.5-894.7) | 1239.3 (864.3-1698.7) | 2238.5 (1585.2-3164.2) | 4140 (2933.8-5616.3) | 5962.4 (4159-8126.4) | 9335.7 (6791.9-12636.2) | 13523.6 (9848.3-17862.8) | 17234 (13199.9-22355.4) | 20421.4 (15220.6-25769.3) | 23057.7 (18194.3-28957.6) | 24556.5 (18801.9-30838.3) | 28244.1 (22200-35937) | 35901.7 (26772.5-46117.1) |
| Chile | 39.7 (26.4-55.2) | 63.8 (40.8-87.2) | 104.1 (70.1-142.3) | 186.8 (122.5-252.9) | 305.4 (211.5-412.4) | 473.7 (316.4-638.4) | 685.2 (465-924.9) | 894.7 (611.8-1215.6) | 1230.9 (816.6-1661) | 1643.2 (1100.2-2211) | 2221.8 (1458.9-3019.4) | 2904.4 (1928.8-3997.2) | 3770.2 (2478.5-5201.9) | 5820.2 (3772.1-8000.7) | 10355.7 (6656.5-14282.3) |
| China | 152.4 (118.2-195) | 259.2 (201.3-330.9) | 423.7 (319.5-538.2) | 708.1 (533.9-928.9) | 923.3 (680.2-1221.6) | 1496.1 (1102.4-1944.9) | 2159.2 (1597-2814) | 3315.6 (2473-4305) | 4995.2 (3757.8-6465.1) | 8118.8 (6145.9-10390.7) | 11613 (8792.3-14848.6) | 17537.1 (13383.6-22055.4) | 26309 (20003.1-32738.7) | 35390.1 (26860.7-44142.8) | 41032.2 (29431.2-52066.9) |
| Colombia | 35.3 (22.2-51.5) | 54.9 (35.1-80) | 86.5 (55.2-127.6) | 144.9 (89.4-214.6) | 238.8 (147.7-351.7) | 384.5 (241.8-567) | 576.2 (356.4-840.5) | 901.3 (557.7-1318.8) | 1258.2 (785.4-1839.2) | 1764.3 (1120.3-2573.2) | 2350.8 (1493.7-3375.1) | 3289 (2094.1-4720.3) | 4102.6 (2599-5856) | 5122.3 (3192.5-7443.6) | 4881.3 (2812.9-7230.1) |
| Comoros | 180.3 (123.7-253.4) | 308.3 (213-426.9) | 465.3 (327.3-631.6) | 852.3 (587.3-1180.1) | 1337.1 (931.9-1833.3) | 2111.9 (1458.9-2859.9) | 3393.6 (2352.4-4584.6) | 5417.3 (3790.7-7146.5) | 7867 (5768.1-10383) | 10868.9 (8058.1-14269.3) | 13613.7 (10281.6-17827.8) | 15548.4 (11947.5-19650.8) | 17100.9 (12231.1-22323.4) | 20586.9 (14545.9-27362.2) | 22880.7 (15664.4-30369.6) |
| Congo | 153.1 (94-235.1) | 299.7 (182-475.9) | 505.9 (321.7-807.1) | 1059 (654.7-1649.2) | 1976.2 (1225.2-3039.4) | 3164.7 (2022.1-4589.4) | 4964 (3361.6-6984.8) | 7576 (5053.1-10235.8) | 10870.8 (7840-13917.2) | 14837 (10987.9-18981) | 18529.2 (13334.6-23523.1) | 21768.9 (16383.1-27688.7) | 24071.7 (17602.5-31201.7) | 27560 (19631.4-35739.5) | 28872 (19629.2-38900.2) |
| Cook Islands | 21.4 (0-53) | 41.9 (0-105.1) | 67 (0-170.1) | 123.4 (0-316.9) | 167.4 (0-419) | 234.1 (0-581.7) | 304.1 (0-744.4) | 416.5 (0-1055.2) | 486.4 (0-1243.2) | 594.1 (0-1427.4) | 803.9 (0-1979.9) | 978.6 (0-2395.7) | 1403.3 (0-3511.7) | 1884.6 (0-4622.8) | 2629.2 (0-6397.6) |
| Costa Rica | 31.5 (20.5-45) | 53.6 (34.7-78.4) | 82.8 (53.2-121.6) | 146.6 (94-210) | 225.9 (142.8-322.8) | 312.5 (198.5-446.2) | 454.4 (290.9-639.7) | 604.4 (387.4-862.6) | 816.7 (527.5-1161.4) | 1066.4 (698.6-1516.2) | 1439.5 (941.5-2036.9) | 1980.6 (1292.6-2757) | 2526.7 (1614.5-3549.1) | 3626.1 (2299.1-5134.2) | 4585.7 (2756.5-6623.6) |
| Côte d'Ivoire | 214.6 (144.4-305.4) | 390.3 (268.4-554.2) | 630.8 (437.2-889.2) | 1229.6 (833.3-1755.2) | 2070.8 (1416.6-2922.3) | 3594.3 (2514.9-4961.1) | 5057.9 (3501.8-7036.2) | 7944.5 (5438.2-10989.8) | 11484.2 (8469.5-15504.7) | 14940.4 (10904.6-19894.7) | 18549.5 (14152.2-23707.7) | 22011 (17600.9-27942.6) | 25448 (20087.8-32196.4) | 30848.6 (23938.9-37964.3) | 39043.2 (28963.1-48695.7) |
| Croatia | 17.9 (12.8-24) | 33.5 (24.5-44.7) | 69.1 (50.9-91.8) | 135.5 (97-182.3) | 271.4 (195.5-360.6) | 457.6 (327.8-618.3) | 720 (517.8-959.9) | 1090.2 (783.4-1452.1) | 1608.7 (1166.2-2113.1) | 2415.1 (1750.5-3105.1) | 3766.2 (2750.5-4824.9) | 6033 (4446.7-7702.4) | 9813.3 (7147.4-12571.3) | 14501.1 (10423.3-18730.8) | 23907.6 (16762.1-31349.2) |
| Cuba | 43.2 (20.9-72) | 67.8 (34.2-109.8) | 131.3 (65.6-217.4) | 253.8 (129.1-420.7) | 442.4 (210.7-731.2) | 731.3 (369.4-1221.1) | 1141.1 (559.5-1861.7) | 1790.7 (898.9-2869) | 2587.6 (1290.4-4235) | 3242.4 (1603.5-5326.7) | 4330.1 (2124.4-7138) | 5641.6 (2732.6-9142.5) | 7068.9 (3397.3-11319.2) | 9861.5 (4752.6-15879.8) | 13450 (6427.4-22080.5) |
| Cyprus | 13.6 (9.1-19) | 21.2 (14.4-30.1) | 46.7 (30.6-67.3) | 92.6 (63.1-122.7) | 194.3 (125-270.9) | 286.3 (190.1-389.4) | 431 (291.9-595.3) | 633.1 (439.6-867.6) | 815.3 (553.1-1115.2) | 1324.2 (944.1-1814.5) | 1670.2 (1167.1-2343.6) | 2972.1 (2102.3-4003.6) | 5122.1 (3632.5-6852.8) | 13683.2 (9559.8-18056.7) | 26389.8 (18386.7-35516.4) |
| Czechia | 19 (14.3-24.3) | 34.5 (25.2-44.5) | 60.3 (43.6-79.5) | 103 (74.4-136.6) | 194.9 (139.9-260.6) | 360.4 (262-476.6) | 569.7 (413-756.4) | 981.9 (721.2-1284.4) | 1357.8 (990.9-1775.9) | 2042.8 (1493.4-2631) | 2901.9 (2128.2-3737.1) | 4500.7 (3250-5786.3) | 7973.9 (5715.7-10315.3) | 10455.3 (7394.5-13593.1) | 13377.8 (9141.7-17862.2) |
| Democratic People's Republic of Korea | 511.8 (343.3-786.1) | 779.4 (513.8-1203.7) | 1258 (848-1850.8) | 2167 (1458.5-3148.7) | 3501.7 (2378.6-4756) | 5321.8 (3802-7066.6) | 7410.3 (5455.4-9420) | 10048.4 (7654.6-12766.5) | 13176.9 (10224.6-16272.4) | 16731.5 (13104.9-20312.5) | 20710.4 (16012.1-25472.6) | 25489.9 (19037.4-32002.8) | 31056.6 (22015.7-39687.7) | 39528.4 (26078.9-52512) | 52767.6 (32626.2-68683.7) |
| Democratic Republic of the Congo | 135.7 (91.2-194.7) | 268.6 (174.7-397.2) | 457 (311.4-652.7) | 986 (656.8-1411.7) | 1866.3 (1242.9-2693.3) | 3190.5 (2128.2-4464.2) | 5174 (3606.2-7196.9) | 8160.9 (5791.6-10938.7) | 12082.9 (8833.5-15690.8) | 16435.4 (12139.8-21717.1) | 19826.9 (14697.1-25926.5) | 23090.9 (16755.1-31063.1) | 23672.6 (16853.1-32241.3) | 26029.5 (18151.5-36106.3) | 26007.8 (17592.3-36391) |
| Denmark | 5.7 (3.4-8.3) | 9.7 (5.6-14.2) | 18.9 (11.5-27.2) | 34.2 (20.8-49.7) | 63.9 (37.8-92.8) | 91.8 (55.3-131.1) | 151.9 (90.8-218.1) | 231.6 (139-334.3) | 319.6 (193.2-462.6) | 451.6 (273.9-649.5) | 663.8 (399.9-957.1) | 979.1 (599.9-1403.4) | 1692 (1021.6-2447.8) | 2759.8 (1642.7-4076.5) | 3585.6 (2036.4-5333.3) |
| Djibouti | 137.1 (74.3-220.9) | 245.5 (143.9-388.5) | 377 (210.2-582.8) | 758.5 (457.3-1162.4) | 1240.1 (679.7-1998.8) | 1992.2 (1189.7-3089.5) | 3227.6 (2049.3-4892.3) | 5123.1 (3119.7-7873.7) | 7179.7 (4485-10825.8) | 9638.7 (6152.6-13984.1) | 11704.6 (7596-16901.3) | 13609.5 (9071.8-19460.6) | 14901.9 (9422.7-21081.2) | 17422.3 (10909.2-25327.8) | 18495.8 (10827.1-27917.5) |
| Dominica | 38.5 (16.8-67) | 69.4 (30.1-124.8) | 127.6 (57.1-222.7) | 252.8 (108.1-446.3) | 461.6 (208.3-800.7) | 812.2 (362-1458.9) | 1234.2 (563.6-2146.6) | 1864 (806.7-3269.2) | 2644.2 (1174.6-4450.1) | 3867.9 (1764-6703.7) | 5233.1 (2388.4-8792.1) | 6971.7 (3195.7-11599.7) | 8933.4 (3887-15355.8) | 11064 (4920.1-18757.5) | 13927.7 (5888.1-23193.8) |
| Dominican Republic | 140.7 (48.8-269.5) | 214.7 (77.3-386.1) | 335.6 (121.6-606.4) | 595.8 (208.6-1058.1) | 979.3 (356.9-1692.9) | 1417.8 (497.2-2553.2) | 2022.9 (753.2-3612.4) | 2751.4 (1012.2-4883.2) | 3548.3 (1368.3-6184) | 4301.5 (1649.9-7579.6) | 5239.2 (1901.8-9154.5) | 6392 (2413.2-11167.4) | 8130.6 (2979.5-14487.6) | 9262.6 (3410.5-16120.8) | 16435.6 (6063.3-28835.5) |
| Ecuador | 79.9 (47.8-121) | 115.6 (68.8-171.7) | 149.4 (89.2-227) | 234.5 (136-362.3) | 344 (196.2-541.7) | 510.5 (297.2-802.5) | 740.8 (430.1-1159) | 1077.7 (628.5-1665.8) | 1349.7 (794.6-2037.8) | 1813.9 (1088.8-2697.1) | 2396 (1428.1-3568.3) | 3344 (2011.7-4935.9) | 5434 (3265-7910.9) | 9306.5 (5765.5-13330) | 19755.5 (12304.3-28349.3) |
| Egypt | 622.4 (465.7-787.3) | 903.5 (668.1-1170.6) | 1172.3 (879.7-1493.9) | 1834.2 (1353.3-2382.3) | 2898.2 (2137.1-3815.2) | 5091.3 (3804.9-6616.4) | 7814.6 (5734.2-10121) | 11262.5 (8546.4-14467.6) | 15939.1 (12145.3-20397.4) | 24186.7 (18380.7-30510.2) | 32608.6 (25214.8-41437.7) | 47659.4 (36896.6-58104) | 59532.7 (46527.2-72684.7) | 41755.4 (31793.5-52052.5) | 34627.8 (23850.4-45544.9) |
| El Salvador | 89.1 (50.9-132.9) | 144.2 (83.6-211.5) | 231.4 (132.2-348.9) | 385.3 (234.4-568.6) | 556.2 (327.5-846.2) | 832.6 (510.2-1236.4) | 1169.2 (689.3-1738.3) | 1586.1 (947.8-2312.5) | 2057.8 (1255-2987.8) | 2835.3 (1757.3-4064.2) | 3651.5 (2255.3-5227.8) | 4986.8 (3128.6-7004) | 6507.1 (3839-9294.5) | 9314.2 (5526.7-13075.3) | 9431 (5399.1-13641.7) |
| Equatorial Guinea | 75.5 (33.5-137.9) | 151.5 (71.4-271.7) | 253.6 (115.9-459.9) | 523.7 (248.9-936.4) | 948.1 (442.2-1645.8) | 1539.4 (746.4-2720.2) | 2459.2 (1210.7-4132.5) | 3896.2 (1862.8-6713.9) | 5999.4 (3010.2-9968.1) | 8353.6 (4501.9-13425.9) | 10914.6 (6074.5-17239.8) | 13503.3 (7505.4-21280.8) | 14805.3 (8038.7-23069.4) | 17211.1 (9249.3-27489.9) | 17569.4 (9067.5-28331.9) |
| Eritrea | 250.2 (152.6-376) | 474.4 (287-718.2) | 741.3 (462.4-1107.1) | 1477 (941.9-2224) | 2359.2 (1583.9-3456.1) | 3471.8 (2320.7-5056.1) | 5226.7 (3556.3-7360.8) | 7855.2 (5648.6-10494.2) | 10534.7 (7748.5-13718.2) | 14017.1 (10544.9-18361.4) | 17560.6 (13039.5-22594.5) | 19497.6 (14695.5-25156.5) | 21336 (15549.5-28473.6) | 24693.1 (17521.7-32728.7) | 24350.9 (16207.8-34001) |
| Estonia | 4.8 (1.5-10.1) | 7 (2.1-15.1) | 16.1 (4.8-33.3) | 29.3 (9-59.8) | 56 (17.1-113.5) | 111.7 (34.1-224.7) | 168 (52.5-328.6) | 262.9 (80.2-512.9) | 368.6 (113.2-724.5) | 517.8 (156.6-1021.9) | 668.3 (214.1-1335.4) | 984.4 (295.1-1921.7) | 1670.9 (513.8-3363.5) | 2626.9 (802.8-5252.5) | 4703.5 (1441.6-9481.4) |
| Eswatini | 113.7 (53.8-190.5) | 207.1 (94.3-348.3) | 434.5 (191-773.7) | 947 (437.4-1670.8) | 1782.8 (834.3-3204.1) | 2751.5 (1373.1-4845.4) | 3846.9 (1915.6-6586.1) | 5651.8 (2830.9-9675.6) | 7645 (4257.2-12234.4) | 10025.9 (5425.1-16203.4) | 13390.7 (7353.1-20786.2) | 16793.3 (9419.8-24677.1) | 19562.8 (10779.7-28429.4) | 12630.6 (6383.8-20182.9) | 7080 (3597.8-12018.3) |
| Ethiopia | 154.6 (114.7-198.4) | 268 (204.6-338.7) | 370.3 (282.7-471.6) | 673.4 (513.4-842.2) | 1051.1 (814.3-1312.4) | 1708.1 (1328.9-2143) | 2852.8 (2204.3-3569.2) | 4606 (3608.2-5705.5) | 6549.2 (5219.5-8042.9) | 8795.1 (7123-10661.9) | 10502.3 (8512-12704.6) | 12044.8 (9581.3-14612.4) | 13296.7 (9910.8-16685.7) | 15568.1 (11132.9-19855.6) | 15063.9 (10057.1-19921.7) |
| Fiji | 244.2 (75.3-449.5) | 406.5 (127.2-752.6) | 714.6 (220.8-1332.2) | 1251.1 (393.5-2214.6) | 2079.4 (661.5-3839.4) | 3085.5 (992-5760.1) | 3903.7 (1182.8-7083.2) | 5382.2 (1735.3-9619.7) | 6169 (1999.3-10887.9) | 7859.3 (2577.3-13941.2) | 9426.2 (3072-16331.5) | 11541 (3746.5-19923.6) | 17319.8 (5813.6-29729.7) | 19295.3 (6710.6-33291.2) | 25313.3 (9018.8-43332) |
| Finland | 2.5 (0.3-5.5) | 4.4 (0.6-9.4) | 7.1 (0.9-15.3) | 12.4 (1.7-27.2) | 21.5 (2.9-46.3) | 40.4 (5.4-86.5) | 63.2 (8.3-134.6) | 97.3 (13-206.8) | 139.2 (18.6-296.7) | 199.7 (27-426.7) | 271.8 (36.6-577.2) | 439 (57.3-944) | 769.3 (99.2-1703.9) | 1111.4 (143.6-2456.9) | 1636.4 (212.5-3626.9) |
| France | 5.8 (3.8-8.3) | 11 (7.4-15.3) | 19.9 (13-28.5) | 35.4 (23.1-49.9) | 59.6 (39.3-83.4) | 89.4 (58.9-123) | 126.9 (83.3-177.2) | 178.8 (116.3-250.5) | 246.3 (162.3-346.6) | 343.4 (228-471.6) | 485.3 (323.3-664.1) | 717.7 (474.2-1003.4) | 1239.1 (803.2-1738.8) | 2421.6 (1543.5-3395.3) | 3970.9 (2499.9-5724.1) |
| Gabon | 58.1 (30.3-96.4) | 123.6 (63.1-218.1) | 219 (117.3-379.6) | 476.5 (253.2-827) | 888 (460.3-1480.7) | 1451.1 (808.7-2334) | 2283.8 (1318.6-3707.2) | 3611.1 (2024.2-5622) | 5401 (3139.1-8277.7) | 7404.4 (4355.8-11299.8) | 9489.6 (5640.1-14262) | 11864.3 (6989.5-17756.3) | 13221.3 (7922-19333.1) | 15289.9 (8810.3-22304) | 15302.4 (8705-23276.9) |
| Gambia | 250.7 (170.2-366.6) | 445.5 (299.7-641.4) | 718.4 (496.1-989.7) | 1416.5 (982.2-1948.9) | 2494 (1658.8-3442.4) | 4441.7 (3154.4-5917) | 6418.6 (4537.9-8471.5) | 10031.1 (7222.9-12961.1) | 14280.9 (10501.9-18076) | 19066.6 (14451.7-23945.8) | 23639.3 (18029.5-29204) | 27574.9 (21400.4-33709.6) | 32292.1 (23898.9-40694.1) | 38201.9 (29453.5-47784.9) | 45880.5 (34668.6-57455.8) |
| Georgia | 76.4 (47.8-112.4) | 168.1 (103-241.6) | 322.7 (197.2-463) | 601.8 (374.6-864.5) | 986.2 (613.8-1445.9) | 1443.1 (895.5-2096.6) | 2111.5 (1328.3-3078.9) | 2839.2 (1779.7-4104.3) | 4015 (2499.8-5831.6) | 6459.8 (4014-9273.3) | 9470.9 (5854.8-13604.2) | 13013.7 (7926.8-18500.5) | 12878.3 (7643.9-18462.7) | 12621.6 (7065.1-18483.2) | 11107.4 (5569.8-16787.5) |
| Germany | 7.2 (4.7-10.1) | 14 (9.4-19.1) | 27.5 (19-38.2) | 50.5 (33.9-70.7) | 96.4 (65.2-130.6) | 160.4 (107.8-219.3) | 244.7 (163.8-335.4) | 374 (251.6-509.6) | 534.7 (368-725.6) | 714.1 (492.9-978.2) | 1109.5 (766.4-1496.5) | 1546.4 (1048.4-2119.9) | 2414.1 (1542-3343.5) | 4223.9 (2719.4-5836.8) | 6131.3 (3850.3-8638.9) |
| Ghana | 234.4 (163.4-325) | 450.9 (307.3-604.7) | 711.3 (498.1-974.5) | 1471.7 (1021.9-1965) | 2468.9 (1702.3-3338.5) | 4116.3 (2849.2-5397.4) | 5535.4 (3917-7387) | 8529.2 (6082.7-10991.8) | 11984.2 (8911.3-15144) | 15452.1 (11330.9-19581.4) | 19517.9 (14793-24335) | 23547.8 (17796.8-29464.8) | 28403.4 (21342.6-36024.3) | 34226.7 (25427.5-43111.4) | 43595.1 (32485.8-54560.8) |
| Greece | 29 (21.9-36.8) | 52.1 (38.2-67.3) | 95.9 (70.4-125.3) | 175.4 (128.9-226.8) | 307.6 (225.4-390.1) | 486.1 (354.9-626.5) | 664 (487.2-850.1) | 827.2 (611.5-1056.9) | 1066.1 (795.9-1366.9) | 1380.5 (1041.8-1740.6) | 1900.7 (1438.9-2386.1) | 2915.6 (2161.6-3660.7) | 4698.5 (3478.3-6087.8) | 7879.9 (5720.9-10083.4) | 11919.6 (8700.4-15339) |
| Greenland | 6.6 (0.2-18) | 12.3 (0.4-31.1) | 24.9 (0.9-65.7) | 52.1 (1.8-140.5) | 95.2 (3.5-263.8) | 160.5 (6-431.8) | 220.5 (8.7-607.4) | 315.7 (11.7-854.1) | 390.9 (15.4-1051) | 521.8 (19.1-1386.8) | 670.9 (24.6-1794.7) | 872.9 (33.5-2304.8) | 1232.1 (45.4-3147.4) | 1729.3 (64.4-4638.9) | 2768.2 (98.3-7172.2) |
| Grenada | 54.3 (21.9-101) | 100.8 (40-179.8) | 203 (81.8-362.5) | 300.4 (119.3-539.3) | 575.2 (233-999) | 1024.7 (415.2-1788.8) | 1619.9 (629.6-2853.8) | 2336.6 (946.5-4221.3) | 3084.3 (1237.7-5418.5) | 4188.5 (1638.4-7354.3) | 6831.3 (2737.5-12113.3) | 6842.3 (2795.3-11987.7) | 8064.4 (3230.4-14228.4) | 11316.8 (4648-19829.8) | 14197.1 (5803.2-25293.6) |
| Guam | 61.6 (29.8-98.9) | 148.1 (72.9-233) | 230 (105.9-355.3) | 435.3 (200-688.6) | 696 (322.1-1086.4) | 784.5 (378.6-1209.1) | 1056.8 (512.3-1667.7) | 1229.5 (571.9-1882.4) | 1383.1 (660.7-2206.4) | 1587.9 (758.8-2455.2) | 1596.1 (766.5-2471.5) | 1565.8 (753.3-2418.2) | 1373 (658-2140.9) | 1355.5 (637-2205.9) | 2130.4 (967.2-3479.4) |
| Guatemala | 168.5 (111.2-224.4) | 254.6 (170-340.1) | 369.5 (239.7-497.9) | 534.4 (356.4-733.5) | 755.2 (496.6-1017.2) | 1073.9 (705.1-1431.9) | 1454.5 (961.9-1979.8) | 2106.4 (1370.7-2829.4) | 2838.1 (1909.7-3792.9) | 4018.1 (2648.2-5365) | 5383.2 (3582.4-7049) | 8115.6 (5320.6-10462.6) | 11859.5 (7780.7-15392) | 23656.5 (15534.9-30203.3) | 49119.5 (33165.3-62883.9) |
| Guinea | 244.6 (172.3-337.8) | 442.4 (305.8-624.8) | 660.1 (455.5-910.7) | 1258.7 (871.7-1754.5) | 2169 (1488-3064.3) | 3958.1 (2797.7-5403.7) | 5735.5 (3954.4-7918.1) | 9051.5 (6237.8-12378.6) | 12966.9 (9571.4-17805) | 17030.5 (12728.5-22736.4) | 20655.5 (16079-26422.5) | 23712.9 (18657.1-29977.3) | 26482.6 (20951.6-32872.5) | 31468.6 (25124.6-39123.1) | 37699.6 (28547-46961.3) |
| Guinea-Bissau | 371.5 (251.6-524.7) | 701.2 (486.4-1000.5) | 1104.2 (755.2-1634.6) | 2246.3 (1514.7-3269) | 3931.1 (2679-5493.6) | 6487.2 (4675.8-8807.9) | 9078.5 (6527.6-11927.1) | 13500 (9960.9-17917.5) | 18337 (14329.9-23370.9) | 23321.8 (17880.8-28826.7) | 28095.5 (21379.1-33906) | 31652.7 (24890.5-38329.4) | 36367.4 (27547.1-45392.6) | 40735.2 (30942.1-51173) | 40148.2 (28137.4-53622.6) |
| Guyana | 143.4 (67.6-243.9) | 238.4 (112.4-403.6) | 388.8 (177.4-665.8) | 711.9 (334.2-1228.3) | 1294.9 (599.2-2228) | 2280.4 (1047.7-3845.1) | 3400.5 (1581.3-5824.6) | 4730.7 (2258.3-7955.8) | 6231.5 (2922.9-10189.9) | 7995.8 (3784.3-13068.9) | 8907.3 (4230.5-14458.6) | 10437 (4860.8-16700.7) | 11152.2 (5281.9-17997) | 17081.2 (8063.6-27484) | 21740.3 (9724.3-38336.2) |
| Haiti | 454.5 (297.9-643.7) | 711.6 (471.6-1036.4) | 1188.4 (831.5-1684.5) | 2172.2 (1491.8-3151.9) | 3635.8 (2506-5337.1) | 5927 (4135.6-8368.3) | 8772 (6173.6-12368.9) | 12729.3 (8822.1-17170.7) | 17365.6 (12567.2-22901.3) | 22893.1 (17122.7-29785.8) | 27599.2 (20559.1-36140.2) | 32312 (24671.1-41269.7) | 36187.7 (27357.6-46423.5) | 42516.8 (31700.2-54512.2) | 50112.3 (30297.7-68136.7) |
| Honduras | 133.1 (66.4-214.2) | 214.7 (111.2-353.5) | 367.4 (202-567.2) | 718.6 (433.7-1077.7) | 1334.7 (906.9-1917.5) | 2326.2 (1605.3-3220.9) | 3732.6 (2661.2-5071.1) | 5527.9 (3915-7326.2) | 7947.6 (5757.8-10439.2) | 11068.3 (8233.4-14193.2) | 14691.8 (10696.8-19343.8) | 18731.1 (13820.8-24309.1) | 23072.8 (17029-30133.5) | 30912.2 (22608-40226.8) | 42030.2 (30348.2-53342.9) |
| Hungary | 24.8 (17.6-37.3) | 49.9 (35.2-73.6) | 95.6 (67.6-144.5) | 185.6 (129.9-279.2) | 350.7 (249.6-526.2) | 681.2 (483.4-1010.5) | 1097.2 (768.2-1628.4) | 1664.6 (1174.7-2470.1) | 2139.9 (1533.8-3183.1) | 2866.1 (2077.8-4315.7) | 4065.2 (2881.7-6044.5) | 6149 (4297.5-9272.4) | 9178.3 (6422.6-13622.5) | 10123 (6880.8-14726.8) | 13952.2 (9372.6-20298.8) |
| Iceland | 3.3 (0.6-7.2) | 5.2 (1-11.4) | 6.8 (1.3-15.1) | 15.7 (2.8-34.7) | 23.4 (4.2-51) | 25.5 (4.7-57) | 54.9 (10.2-120.6) | 62.2 (11.2-137.4) | 96.2 (17.4-207.5) | 132.3 (24.2-291) | 235 (42.8-508.6) | 322.4 (59.1-731.1) | 596.8 (109.4-1311.1) | 1028.7 (190.3-2325.1) | 980.1 (178.8-2232.6) |
| India | 242.5 (195.4-289.4) | 493.2 (404-589.1) | 737.1 (605.2-878.9) | 1427.3 (1170.7-1718.1) | 2170.1 (1760.6-2595.7) | 3339.5 (2735.3-4021.1) | 5575 (4570.8-6694.3) | 6849.7 (5686.1-8216.8) | 9223.2 (7624.2-10930.1) | 11251 (9318.8-13391.3) | 12729.8 (10465.5-14960.4) | 13407.5 (10867.3-15796.6) | 14464.2 (11715.7-17164.1) | 19364.9 (15486.1-23038.7) | 25150.8 (19742.7-30103) |
| Indonesia | 233.6 (162.2-339.3) | 390.7 (269.1-564.4) | 642 (449.3-918.2) | 1090.9 (770.1-1563) | 1727.4 (1180.2-2462.8) | 2672.1 (1837.6-3778.3) | 3791.7 (2578.7-5318.7) | 5243.7 (3481.9-7299.1) | 7170.6 (4850.1-9938.9) | 9588.1 (6561.9-12860.9) | 12384.9 (8535.6-16618.4) | 15408.6 (10735.4-20438.3) | 18663.7 (12835.3-25014.1) | 22935.5 (15544.2-30653.1) | 28299.9 (18768.7-39111) |
| Iran (Islamic Republic of) | 146.4 (118.1-176.2) | 208 (166.7-252) | 301.5 (238.9-363) | 493.8 (388.6-597.1) | 817.3 (638.7-998.9) | 1329.2 (1042-1618.5) | 2011.5 (1579.3-2448.3) | 2964.1 (2330.1-3560.4) | 4122.3 (3274.3-4942.5) | 5675.5 (4494.2-6812.9) | 8909.5 (7098.5-10612.9) | 12228.6 (9438.3-14700.9) | 16193.5 (12440.9-19686) | 22036.7 (16351.9-26931.7) | 26214.8 (18297.5-33027.6) |
| Iraq | 158.8 (103.7-241.1) | 248.9 (169.3-374) | 454.1 (307.4-685.9) | 944.9 (637.9-1389.6) | 1911.1 (1275.9-2844.5) | 3336.1 (2229.9-4834.9) | 5179.3 (3408.7-7494.2) | 7513.8 (4905.8-10398.5) | 10615 (7133.2-14338.6) | 14515.3 (10192.9-19282.2) | 19124.4 (13564.6-25012.9) | 24171.9 (17796.7-30893) | 30356.3 (22714.2-38608.7) | 39407.7 (28747.8-50300.6) | 52260.1 (35233.8-69589) |
| Ireland | 4 (2-6.4) | 8 (4.1-12.8) | 14.8 (7.3-23.3) | 28.8 (14.4-45.5) | 52.7 (26.7-83.9) | 77.9 (39.1-123) | 119.6 (60-186.3) | 162.4 (82.2-254.3) | 214.5 (108.4-335.8) | 343.2 (173.8-535.7) | 516.7 (260.5-805.9) | 813.4 (407.4-1264) | 1297.6 (653.4-2052.7) | 1991.1 (985.8-3142.2) | 3094.5 (1549.2-4986.5) |
| Israel | 10.9 (8.3-14.1) | 20.6 (15.6-26) | 32.7 (24.3-41.8) | 59 (45.1-75.1) | 106.7 (81.5-134.5) | 202.8 (157.5-254.4) | 320.4 (245.2-399.2) | 431.8 (331.1-537) | 598.8 (458.3-737.7) | 873.5 (671.1-1086.4) | 1313.2 (991.4-1644.5) | 2110.6 (1586.6-2657.1) | 3546.8 (2571.7-4513.8) | 5363.6 (3838.7-6916.2) | 7808.6 (5451-10018.3) |
| Italy | 13.7 (10.3-17.5) | 21.1 (15.7-27) | 33.6 (25.2-42.9) | 59.7 (44.4-75.7) | 99.8 (74.4-126.7) | 156.9 (117-199.8) | 231.4 (172.8-291.4) | 337.4 (253-424.1) | 487.8 (362.2-613.6) | 727.7 (542.4-922.6) | 1158.7 (858.1-1473.4) | 1802.8 (1319.1-2303.5) | 3179.4 (2295-4145.9) | 5596.4 (3863.8-7378.1) | 9250.6 (6030.7-12288.6) |
| Jamaica | 38.3 (21.5-59.7) | 67.1 (36.5-105.9) | 121.1 (65.8-187.5) | 223.6 (120.4-346.2) | 386.5 (208.7-621.9) | 601.2 (329.2-951.1) | 922.3 (499.3-1466) | 1380.2 (759.3-2136.2) | 2004.1 (1122.3-3090.4) | 3006.6 (1701.2-4607.5) | 3839.9 (2126.3-5872.5) | 4819.2 (2645-7162.6) | 5710.1 (3176-8487.5) | 7174.7 (3999.2-10520.6) | 7773.7 (4278.3-11717.6) |
| Japan | 14.5 (7.9-21.9) | 26.7 (14.6-39.9) | 46 (25.3-69.4) | 82.7 (46.1-124) | 137.6 (77.4-206.4) | 200.3 (111.5-301.9) | 264.8 (148-399.2) | 333.7 (185.9-502.3) | 425.4 (236.1-641.1) | 552.8 (302.5-832.2) | 771.5 (422.7-1164.9) | 1056 (559.8-1599.4) | 1600.9 (844.2-2432.5) | 2710.8 (1397.6-4211.4) | 3725 (1842.2-5891.8) |
| Jordan | 103 (73.3-135.1) | 163.3 (117.6-215.8) | 255.6 (182.8-341.1) | 433.3 (304.3-572.4) | 733.2 (506.6-1010.9) | 1102.7 (772.2-1490.4) | 1560.8 (1092.6-2103.6) | 2379.8 (1676-3190.8) | 3288.1 (2314.2-4414) | 5062.5 (3691.5-6668.8) | 6198.2 (4558.4-8009.4) | 8326.6 (6008.4-10782.6) | 13701.4 (10025.3-17297.9) | 17304.7 (12254.2-22306) | 17709.7 (11996.2-23459.4) |
| Kazakhstan | 47.2 (30.5-68.7) | 110.2 (70.7-157.9) | 218.8 (141-315.4) | 432.1 (290.5-592.1) | 773.6 (521.6-1052.3) | 1298.7 (883.6-1768.5) | 2094 (1440.2-2869.5) | 3480.1 (2403.7-4711.1) | 5091.6 (3564.3-6835.3) | 7938.9 (5548.7-10637.6) | 10862.5 (7548.8-14568) | 16400.1 (11380-22020.5) | 21239.3 (14615.6-28774.7) | 28771.6 (19690.6-39125.9) | 43509.4 (29328.9-59702.7) |
| Kenya | 111.3 (83.2-144.8) | 202.2 (151.8-264.7) | 311.9 (234.2-407.7) | 620.3 (463.1-810) | 1040.6 (778.4-1343.7) | 1669.3 (1277.6-2155.4) | 2607.9 (2011.7-3343.9) | 4464.2 (3451.1-5763.5) | 6469.2 (5040.9-8279.6) | 9292.6 (7206.6-11833.1) | 12659 (9910.6-16076.6) | 15632.6 (11907.1-20048.9) | 17859.5 (12964.9-23443.4) | 20739.2 (14584.4-27459.6) | 20777.2 (13939.7-27662.9) |
| Kiribati | 730.7 (488.5-1014.6) | 1230.6 (826.5-1718) | 1938.5 (1282.5-2738.3) | 3143.5 (2049.2-4412.8) | 4477.3 (3027-6321.9) | 6071.9 (4345.8-8598.8) | 7805.3 (5660.9-11020.8) | 9677.2 (7138-12635.6) | 10867.8 (8079.9-14585.5) | 12754.8 (9541.7-16294.8) | 15696.8 (11822.9-20296.1) | 17910.1 (13208.7-23389.1) | 22320.2 (16640.5-28409.8) | 24732.9 (18551.5-31688.7) | 32532.9 (23236.6-42902.2) |
| Kuwait | 200 (152.4-248.8) | 314.2 (239.9-393.9) | 448.3 (331-574.4) | 707.6 (532.7-921) | 1066 (773.7-1401.5) | 1620.9 (1167.5-2132.5) | 2308.6 (1656.7-3038.3) | 3319.2 (2400.5-4323.7) | 4374 (3198.6-5730.3) | 5889.4 (4427.9-7589.4) | 7543.6 (5600.5-9713.7) | 10187.4 (7589.2-12931.5) | 14048.8 (10728.8-17895.9) | 18433.9 (13393.9-23271.3) | 20698.9 (13489-27086.2) |
| Kyrgyzstan | 131.3 (95.4-171.1) | 300.8 (224.5-391.1) | 546.2 (403.5-709.7) | 890.1 (652-1160.5) | 1482.2 (1093.6-1901.4) | 2475.6 (1825.6-3157.9) | 4015.3 (2991.1-5115.2) | 6334.9 (4735-8110.8) | 9241.6 (6951.3-11627.1) | 12527.2 (9525.3-15765.2) | 18209.3 (13695-22949.5) | 22680.4 (17052.2-28689.7) | 26399.3 (19414.2-33775.2) | 34245.3 (24831.1-44046.9) | 59310.1 (42419-77393.4) |
| Lao People's Democratic Republic | 525.3 (314.4-765.2) | 789.3 (508.6-1156.3) | 1237.8 (773.8-1779.2) | 2069.9 (1332.5-2895.6) | 3169 (2114.8-4432.2) | 4909.8 (3201.5-6702.7) | 7052.8 (4757-9570) | 9870.3 (6278.1-13445.4) | 13517.6 (9072.1-18136.1) | 17897.9 (12093.4-23353.9) | 22591.7 (15811.6-29026.3) | 27429.1 (18587.2-34863.5) | 32108.3 (22282.5-40853) | 39117.3 (26725.4-50327.5) | 48868.8 (32955.6-61722.8) |
| Latvia | 19.2 (12.3-28.6) | 46 (29.3-70.3) | 94.5 (59.2-141.3) | 194.4 (123.2-293) | 357.1 (230.6-527.1) | 654.4 (426.2-979) | 1007.4 (654.3-1491.2) | 1521.3 (1008.2-2277.5) | 2134.6 (1385.2-3166.6) | 2943.3 (1942.4-4386.4) | 3919.1 (2556.9-5909.3) | 5697.3 (3720.8-8431.8) | 8673.1 (5621.2-12860.1) | 12120.1 (7883.1-17785.5) | 17512 (11026.9-26130.6) |
| Lebanon | 71 (42.9-105.3) | 105.6 (65.9-154) | 159 (100.6-230.4) | 265 (165.7-382.1) | 428.1 (269.2-627.5) | 630.5 (406.2-913) | 887.5 (546.5-1276.5) | 1248.1 (770.9-1823.8) | 1710.5 (1087.7-2475.6) | 2387.8 (1535.3-3532.2) | 3629.3 (2324.6-5313.7) | 5293.3 (3360.4-7707.9) | 7558.9 (4767.4-11045.9) | 11022.3 (7179-15584.6) | 14406.4 (9239.5-21313.3) |
| Lesotho | 143.8 (89.4-208.9) | 256.9 (151.5-376) | 548.2 (336.7-825.6) | 1205.7 (677.3-2043.5) | 2386.7 (1396.1-4011.1) | 3892.3 (2247.5-6301.2) | 5622.6 (3519.6-8680.4) | 8494 (5466.9-12338.9) | 11507.2 (7867.4-15859.8) | 14974.9 (10321-19762.9) | 19578.3 (13940.3-26592.1) | 23510.3 (16856.5-30930.8) | 26222.7 (19159.1-33713) | 16354.3 (11072.6-22353.8) | 8280.8 (5259-12143.6) |
| Liberia | 240.8 (164.8-335.8) | 409 (280.1-562.5) | 658.2 (462.5-922.6) | 1254.6 (872.3-1755.2) | 2152.3 (1486.6-3025) | 3668.1 (2605-5162.9) | 5445.4 (3899.7-7563.8) | 8332.3 (5944.3-11461) | 12163.4 (8901.8-16023.8) | 15596.2 (11618.6-20463.9) | 20018.4 (15359.8-25959.2) | 23604.7 (18667.1-29564.4) | 26691.1 (20961-33678.2) | 32439.4 (25606.4-40717.5) | 41588.7 (31958.4-51533) |
| Libya | 321.1 (207.3-456) | 463 (300.9-675.1) | 675.3 (445.6-960.5) | 1018.7 (702.2-1451.4) | 1597.5 (1073.8-2289) | 2126.9 (1418.5-3066.5) | 3026.3 (2028.1-4337.1) | 4842.4 (3299-6855.5) | 6636.8 (4480.8-9286.6) | 8324.7 (5735.5-11662.6) | 11102.9 (7776.1-15262.2) | 13048.6 (9083.3-17913.3) | 17203.5 (12038.3-23312.4) | 21387.4 (14370.5-28899.5) | 20820.3 (13117.7-29645.4) |
| Lithuania | 9.6 (5.6-13.8) | 31.9 (18.7-47.1) | 74.8 (45.6-111) | 162.6 (96.9-238.8) | 290.2 (176-428.1) | 451.2 (269.1-651.3) | 673.3 (407.7-976) | 1034.8 (618.4-1508.2) | 1489.1 (875.2-2146.3) | 2034.6 (1235.2-2928.4) | 2812.4 (1703.8-4048.6) | 4103.3 (2465.8-5884.7) | 6736.9 (4018.7-9693.2) | 10629.1 (6276.9-15344.8) | 17597.8 (10332.8-25366.2) |
| Luxembourg | 3 (1.7-4.5) | 5.5 (3.1-8.3) | 11.6 (6.6-17.4) | 25 (13.9-38.1) | 43.5 (24.5-65.7) | 71.7 (41-108.7) | 114.2 (62.9-169.7) | 170.2 (95.6-255.2) | 262.2 (147.9-387.9) | 393.1 (221.5-580.6) | 546.5 (305.5-804.7) | 899.7 (501.4-1325.7) | 1434.3 (790-2118.4) | 2236.9 (1243.2-3331.8) | 2658 (1422.6-3988.4) |
| Madagascar | 501.2 (340.7-702.4) | 818.7 (535.3-1138.6) | 1156.9 (783.5-1604.8) | 2066.3 (1433.3-2856.9) | 3224.9 (2281.9-4657) | 4958.3 (3535.2-6859) | 7487.2 (5378.4-10183.8) | 11086.8 (8003.7-14672) | 15001.7 (11364.4-18993) | 19374.4 (14469.4-24529.3) | 23526 (17659.9-30233.1) | 25576.8 (18894.7-32525.3) | 27139.1 (19538.2-36181.6) | 31086.9 (22714.9-40937.6) | 32562.1 (22226.6-42307.2) |
| Malawi | 259.2 (178.8-357.8) | 466.1 (327.4-637.4) | 696.8 (492.5-949.3) | 1411.3 (1011.9-1901) | 2324 (1671.1-3138.1) | 3645.8 (2616.8-4851.5) | 5509.8 (4141.6-6902.4) | 8366.3 (6271.9-10630.1) | 11458.7 (8960.7-14714.3) | 15220 (12138.4-19088.2) | 19112.8 (14671.3-23715.4) | 21899.3 (17179.1-27834.2) | 23552.9 (17766.7-30472.4) | 25648.1 (18122.3-33966.8) | 17669.3 (11251.6-24481.8) |
| Malaysia | 78.6 (51.1-111.1) | 155.1 (95.5-222.9) | 282.7 (184.5-401.1) | 521.5 (338.5-735.1) | 864 (562.3-1193.2) | 1368.5 (930.3-1887.5) | 1975.2 (1302.8-2761.6) | 2662.5 (1786.3-3677.7) | 3440.7 (2323.1-4749.8) | 4498.1 (3059.3-6187.8) | 6104.9 (4168.8-8359.5) | 7109.1 (4801.4-9848) | 8039.9 (5285.1-11033.6) | 10039.7 (6465-14217.3) | 12259.4 (7709.1-17176.5) |
| Maldives | 58.9 (31-93.4) | 91.3 (48.9-148.7) | 129 (69.4-206.4) | 183 (102.6-296.9) | 253.9 (143.6-397.8) | 396.2 (217.1-637.6) | 537 (296.5-843.9) | 778.1 (434.5-1228.6) | 1120.2 (629.9-1823.1) | 1880.6 (1041.8-2965.2) | 2637 (1497.3-4145.5) | 3863.3 (2151.6-6102.9) | 5106.7 (2800.2-7892.4) | 6831.2 (3774.2-10586.3) | 8107 (4392.6-12934.9) |
| Mali | 218.7 (146.8-304.8) | 382.2 (265.5-515.1) | 534.7 (385.3-728.4) | 910.2 (634.9-1220.8) | 1521.7 (1083.6-2037.5) | 2704.7 (1908.4-3551.4) | 3738 (2663.3-4985.8) | 6002.8 (4343.2-7720) | 8898.6 (6599-11515.1) | 12096.1 (8913-15593.6) | 15395.8 (12129.9-19361.4) | 17426.9 (13588.1-21727.4) | 18918.1 (14656.9-23611.8) | 21743.5 (16496.9-27324.1) | 22865.5 (16234.3-30220.2) |
| Malta | 7.8 (5.5-10.4) | 25.4 (18.3-34.3) | 37.6 (26.4-51.4) | 99.4 (69.6-131.5) | 160.9 (110.1-219.6) | 201.8 (142-271.7) | 359.1 (247-479.4) | 470.3 (328.5-626.2) | 621.9 (430.4-821.7) | 976.6 (682.7-1296.1) | 1317.5 (909-1749.5) | 2426.4 (1685.9-3208.9) | 3742.4 (2496-5016.3) | 3950.1 (2541.1-5404.9) | 5563.6 (3470.1-7777.7) |
| Marshall Islands | 558.5 (351.8-870.5) | 988 (606.6-1529.1) | 1615.3 (1023.6-2499.6) | 2738.2 (1730-4247.6) | 3987.6 (2479.9-6240.2) | 5214.6 (3430.5-8124.9) | 6516.7 (4118-9994) | 8213.4 (5309.8-12380.1) | 9634.3 (6331.8-14397) | 11798 (7984-17881.8) | 14252.2 (9394.4-21305.7) | 16656.7 (11428.8-23465) | 21102.6 (14494.1-29649.9) | 24392.8 (16340.5-34261.4) | 33805 (22387.4-48031.5) |
| Mauritania | 139.2 (86.1-220.2) | 261.2 (156.1-401.9) | 409.5 (240.5-640) | 827.9 (507.9-1243.1) | 1532.6 (980.3-2258.7) | 2834.5 (1911.5-4035.1) | 4155.8 (2897.2-5610.5) | 6643.7 (4763.5-8906.2) | 9846.6 (7021.9-12955.3) | 13615.9 (10307.2-17634.6) | 17494.1 (13215.7-22691.6) | 21350.3 (15986.7-27563.3) | 25843.8 (19345.4-33485) | 31039.1 (22877.2-39828) | 39707.9 (28679.8-51454.5) |
| Mauritius | 55.5 (18.4-97.6) | 105.6 (37.5-186) | 160.4 (55.3-283.5) | 213.6 (73.4-383.8) | 307 (106.5-549.2) | 386.2 (138.6-697.6) | 491.1 (169.2-894.8) | 670.5 (226.2-1169.4) | 896.6 (310.9-1610.7) | 1138.1 (402.2-2055.7) | 1490.8 (527.2-2674.6) | 2139.2 (732.3-3759) | 2481.8 (851.2-4382.1) | 3613.7 (1254.5-6478.2) | 3658.9 (1234.3-6612.3) |
| Mexico | 78.1 (51.1-109.8) | 114.3 (74.8-161.8) | 165.6 (108.5-233.9) | 252.7 (161.6-357.5) | 384.4 (245-547.6) | 563.3 (359.1-790) | 797.3 (506.3-1138.1) | 1097.9 (694.8-1571.5) | 1485.8 (942.5-2120) | 2008.1 (1274-2892.2) | 2752.8 (1766.1-3974) | 4026.7 (2598.3-5707) | 6157.7 (3924.2-8811) | 9654.4 (6162.4-13858.7) | 15012.3 (9322.6-21719) |
| Micronesia (Federated States of) | 552.3 (334.6-844.2) | 1006.2 (596.1-1539.1) | 1617.3 (982.1-2505.1) | 2807.7 (1681.2-4360.1) | 4039.5 (2411.9-6338.8) | 5322.7 (3283.5-8243.8) | 6622.4 (4059.3-10076.7) | 8378.1 (5314-12524.1) | 9784.8 (6264.8-14838.6) | 11703.1 (7415.6-17462) | 14240.2 (9046.6-20369.8) | 16916.2 (10987.2-24554) | 21617.7 (14169.1-31459.5) | 24900.7 (16198.8-35656.9) | 33969.6 (21508-49087.9) |
| Monaco | 12 (5.7-21.7) | 22 (9.7-39.1) | 37.7 (16.7-68.7) | 62.4 (29.9-110.5) | 92.8 (44-159.4) | 132.5 (63.7-227.7) | 184.3 (89-306.1) | 254.8 (125.9-417.3) | 366 (184.6-600.5) | 557.8 (279.2-888.2) | 869.1 (440.4-1398.2) | 1361.2 (666.9-2170.3) | 2219.4 (1131.9-3498.6) | 3303.8 (1669.1-5166.1) | 4091 (1978.7-6694.3) |
| Mongolia | 123.5 (81-173.9) | 269.5 (176-383.3) | 575.3 (383.3-800.8) | 1170.5 (795.8-1710.2) | 2051.7 (1390.9-2853.7) | 3067.2 (2140.4-4224.4) | 4252.1 (2921.5-5771.7) | 6203.2 (4331-8444.7) | 8118 (5753.5-10959.7) | 10661.7 (7332.9-14240.2) | 15425.6 (10735.2-20829.3) | 22888.6 (15947.6-30415) | 30629.6 (20726.1-40972.4) | 37559.8 (24309.6-50847.7) | 35184.4 (20816.6-49099.4) |
| Montenegro | 65.6 (41.2-111.6) | 120.5 (79-201.6) | 202.6 (131.6-328.6) | 377.4 (247-641.1) | 718.2 (472.5-1209.6) | 1019.8 (656.1-1691.4) | 1883.1 (1250.6-3130.7) | 2975.5 (2005.5-4981.6) | 4615.6 (3126-7564.2) | 6660.9 (4577.1-11229.6) | 11387.4 (7669.5-19539.4) | 17806.6 (12014.8-30717.9) | 31141.1 (20962.2-52575) | 43125.1 (28934.3-72356.2) | 34485.2 (22577-57540.2) |
| Morocco | 120.8 (75.5-207.5) | 202.2 (125.6-333) | 351.4 (219.3-554.9) | 682.3 (440.4-1084.2) | 1251.6 (813.9-1875.8) | 2072.7 (1359.1-3023.2) | 3208.2 (2091.5-4533.2) | 4659.9 (3057.7-6314.8) | 6544.5 (4470.6-8525.4) | 8608.2 (5870.5-11191.3) | 11466.1 (8068.5-14920.8) | 14278 (10230.1-18632.2) | 18090.1 (12633.1-23684.2) | 24013.4 (16953.7-31953.8) | 35317 (23729.7-47323.8) |
| Mozambique | 220.2 (144.3-336.1) | 426.7 (261-612.3) | 746.1 (468.7-1131.7) | 1678.3 (1042.6-2448.2) | 2964.8 (1913.2-4259.2) | 4617.2 (3095.8-6250.1) | 7306.3 (5133.7-9789.9) | 11160.8 (8195.1-14808.8) | 14945.1 (10841.7-18983.4) | 18474.4 (14019.7-23468.7) | 22332.3 (16237.6-29005.8) | 22047.5 (16137.4-28494.7) | 21016.4 (14364.3-28310.6) | 23325.8 (15672.2-33303.8) | 16383.1 (9739.1-24961.1) |
| Myanmar | 439.5 (298.5-601.4) | 660.7 (457-907.4) | 1049.7 (756.2-1417.4) | 1756.9 (1205.8-2373.1) | 2730 (1957.8-3710.5) | 4444.9 (3190.7-5795) | 6284.6 (4469.3-8189.5) | 8727.3 (6317-11421.9) | 12046.6 (8591.1-15795.8) | 16398.2 (12132.9-21259.8) | 20732.7 (15523.8-26480.9) | 25554.7 (19035.7-33128.1) | 28797.3 (21490-37957.3) | 34506.2 (25371.2-44470) | 42962.2 (30742.5-55436.7) |
| Namibia | 61.1 (25.3-116.7) | 117 (49.6-231.8) | 244.7 (112.4-451.4) | 530.1 (253.5-937.8) | 1028 (523.9-1712.4) | 1783.9 (910.5-3010.9) | 2671.9 (1386.3-4519) | 4194.4 (2181.9-7123.7) | 5848.3 (3072.8-9494.9) | 8038.1 (4459.4-12838.2) | 10990.8 (5996.4-17565.3) | 14056.2 (7606.1-22058.9) | 16962.4 (8947.3-26782.4) | 17465.1 (9307.7-27302.9) | 10959.2 (5243.8-18801) |
| Nauru | 143.2 (17.3-332.1) | 269.8 (32.7-655.3) | 431.6 (52.3-1028) | 761 (92.7-1821.2) | 1116.8 (130.6-2565.4) | 1453.1 (177.7-3213.7) | 1783.1 (212.1-3986) | 2115.3 (276.6-4759) | 2405.8 (298.6-5464.6) | 2831 (369-6046.1) | 3356.1 (439-7285.7) | 3729.5 (490.8-8122.1) | 4499.7 (641.3-9608.2) | 5183.6 (749.9-11026.1) | 7462.4 (984.4-16490.9) |
| Nepal | 233.1 (151.7-340.8) | 419.3 (267.2-611.5) | 634 (408-888.6) | 1168.8 (797.5-1613.7) | 1967.3 (1366.1-2708.7) | 3165.2 (2251.1-4255.9) | 4872.9 (3489.7-6279.7) | 6767 (5034.5-8857.7) | 9346.4 (6937.1-11922.2) | 12095 (9194.5-15319.4) | 15261 (11917.2-19253.4) | 17603.9 (13586.3-22758.5) | 20700.9 (15648.5-26565.1) | 26406.2 (19735.3-33747.7) | 30882.9 (22072.8-40704.2) |
| Netherlands | 6.3 (4.2-8.6) | 11.3 (7.9-15.7) | 20.9 (14.6-28.5) | 37.7 (26.6-51.8) | 66.3 (46.2-91.1) | 103.9 (72.7-140.4) | 153.2 (108-207.4) | 226.8 (160.2-305.6) | 339.9 (237.2-451.5) | 505.4 (354.6-666.6) | 853.1 (598.3-1136.5) | 1271.6 (876.3-1718.5) | 2204.4 (1529.6-3007) | 3735.9 (2543-5067.3) | 5590.4 (3743.1-7565.5) |
| New Zealand | 3.8 (1.5-6.3) | 8.7 (3.5-14.5) | 17.6 (6.9-29.7) | 35.1 (13.6-58.6) | 55.9 (22.7-93.3) | 91.4 (36.3-154.5) | 124.4 (49.4-212.6) | 166.3 (64.9-277.8) | 226.6 (90-378.6) | 311.4 (124.3-526.1) | 497.2 (200.7-832.5) | 775 (307.3-1293.9) | 1393 (562-2358.4) | 2316.7 (910-3917.8) | 3607.9 (1405.7-6196.7) |
| Nicaragua | 96.8 (67.1-129.4) | 144.8 (100.8-194.8) | 258.1 (182.4-349.6) | 349.3 (245.6-474.6) | 548.3 (384.5-735.8) | 859.1 (609.3-1166.5) | 1346.5 (977-1825.1) | 2090.4 (1544.6-2853.9) | 2803.1 (2054.6-3664.7) | 3803 (2833.7-5052.4) | 4849.5 (3582.5-6331.8) | 6202.7 (4436-8058.5) | 7863.2 (5581.9-10379.1) | 12911.8 (8895.1-16886.9) | 41150.9 (29717-52875.6) |
| Niger | 144.3 (96.2-204) | 262.6 (174.9-371.9) | 416.1 (286.3-580.8) | 833 (548.9-1185) | 1501.2 (1002.7-2149) | 2927.9 (1998.7-4100.2) | 4242.5 (2907.2-6000.8) | 6885.5 (4738.9-9591.7) | 10105.7 (7214-13800.3) | 13247.1 (9627.9-17884.3) | 17443.4 (13266-22402.6) | 20267 (15243.8-25590) | 21807.2 (16388.7-27897.3) | 25789.2 (19176.8-32949.8) | 32579.9 (23487.3-41818.8) |
| Nigeria | 105.3 (76.7-139.1) | 217.4 (152.6-285.3) | 363 (267-498.4) | 690.1 (492.2-956.6) | 1194.1 (877.3-1628.1) | 2176 (1599.4-2973.1) | 3124.1 (2297.8-4277.1) | 5176.2 (3776.1-7022) | 7804.3 (5888.7-10417.8) | 11027.7 (8586.6-14080.2) | 15010.3 (12067.3-18400.4) | 18281.3 (14864.6-22379.4) | 22102.7 (17598.2-26571.8) | 26695.5 (21044.7-32566.7) | 30601.6 (22703.2-37641) |
| Niue | 73.6 (7.7-166.2) | 118.9 (10.5-262.7) | 168.2 (15.7-386.8) | 292.2 (26.7-676.2) | 450.4 (40.3-1061.8) | 662.7 (60.4-1473) | 880.2 (79.2-1991.6) | 1194.7 (113-2671.6) | 1476.2 (143.3-3232.6) | 1907.2 (186.6-4286.2) | 2437.1 (248.8-5400.4) | 2999.1 (308.8-6599.6) | 3881.1 (384.1-8313.6) | 4682.5 (481.5-10072.5) | 6561.7 (685.1-14322) |
| North Macedonia | 62 (43.7-83.4) | 119 (85-169.4) | 230.2 (166.1-316.4) | 437.4 (312.6-595) | 781.2 (537.7-1077) | 1358.8 (901.9-1892) | 2298.5 (1645.6-3127.7) | 3845.8 (2808.6-5135.1) | 5893.7 (4352.9-7837) | 8422.5 (6208.9-10942.4) | 14423.6 (10786.8-18557.8) | 26014.4 (19619.6-33938.7) | 44779.1 (34085.5-57095.9) | 63071.9 (47824-81118.7) | 47707.6 (35166.6-62379.2) |
| Northern Mariana Islands | 50 (22.8-86.1) | 101.2 (46.3-169.9) | 178.4 (80.4-305) | 369.7 (169.5-598.5) | 459 (212.9-750.9) | 849.8 (399.2-1384.5) | 938.6 (431.8-1534.9) | 1399.5 (666.1-2251.4) | 1601.6 (755-2653.2) | 1989.6 (929.5-3206.7) | 2640.8 (1239-4223.9) | 3055.8 (1436.9-4900.4) | 4467.1 (2076.1-7367.1) | 5323.3 (2527.5-8723.2) | 8037 (3824.7-13209) |
| Norway | 1.6 (0.6-3) | 2.7 (1-4.9) | 5.8 (2.1-10.6) | 12.1 (4.7-21.6) | 22.6 (8.7-39.8) | 36.5 (13.4-64.4) | 59.3 (21.6-104.3) | 81.5 (30.1-145.1) | 118 (43-210.3) | 173.7 (63.6-310.6) | 272.3 (100.6-481.2) | 425.7 (155.9-750.9) | 703.6 (259.9-1245.1) | 1134.2 (413-2023.3) | 1532.4 (568.7-2784.8) |
| Oman | 149.6 (106.4-206.9) | 205.1 (141.8-287.3) | 317.2 (221.6-423.1) | 568.4 (383.3-768) | 1004.7 (681.3-1370.5) | 1819.4 (1169.5-2430) | 3001.2 (1989.1-4075.4) | 4551.2 (3006.6-6025.9) | 7460.3 (5049.7-9880.9) | 10447.4 (7431.7-13778.3) | 14183.2 (10114.1-18322.7) | 16205 (11482.8-21248.2) | 25005.4 (18015.6-32606.4) | 23553.8 (16130.6-31043.9) | 22882.6 (14430.3-31505.6) |
| Pakistan | 437.3 (322.7-583.8) | 732.9 (541.5-950.2) | 1102.2 (793.3-1431) | 1769.1 (1297-2311.7) | 2652 (1942-3466.4) | 4023 (3035.9-5174.4) | 5995 (4493.3-7793.9) | 8413.7 (6429.6-10665) | 11651.5 (9027.8-14599.6) | 14778.2 (11793.7-18191.8) | 17791.2 (13942.9-21865.6) | 19730.9 (15382.9-24723.7) | 21407.5 (16380.9-26743.2) | 27420.7 (20917.4-34218.3) | 35331.5 (25558.2-45176.5) |
| Palau | 101.3 (11.8-210.5) | 193.2 (20.6-420.5) | 287.1 (30.1-617.4) | 424.8 (50.6-909.7) | 569.3 (63.9-1189.3) | 728.4 (87.5-1539) | 854.5 (95.3-1778.4) | 1153.7 (125.6-2424.7) | 1342.1 (147.8-2773.5) | 1823.8 (202.6-3872.7) | 2236.1 (232.9-4696.6) | 2769.9 (275.1-5747.1) | 4004.4 (422.4-8164) | 5244.5 (554.8-10592.2) | 8058 (793.7-16658.2) |
| Palestine | 140.8 (91.6-199.3) | 196.1 (128.8-264.1) | 285.3 (188.1-392.9) | 474.1 (318.3-632.8) | 822 (556.9-1110) | 1513 (1024.6-2022.1) | 2521.9 (1727.1-3345.9) | 4043 (2713.2-5323.4) | 5386.4 (3685.8-7086.6) | 8112.5 (5590.3-10626.2) | 10432.9 (7160.4-13759.9) | 15134.9 (10400.6-19699.5) | 21369.7 (14360.6-27934.9) | 32757.4 (22910.4-42792) | 45832.2 (30405.6-60490.7) |
| Panama | 25.5 (13.5-41.8) | 46.6 (25.7-74.8) | 72.3 (38.7-116.3) | 109.8 (58.1-182.3) | 171.1 (89.3-279.3) | 277.8 (148.4-459) | 404.5 (214.8-665) | 550.1 (296.2-895.7) | 769 (409.4-1275.1) | 1081.8 (583.2-1752.6) | 1511.4 (815.7-2415.9) | 1949.6 (1067.4-3126.8) | 2422.8 (1313.8-3908.5) | 3425.4 (1816.8-5574.8) | 4502.8 (2315.6-7558.4) |
| Papua New Guinea | 463.5 (284.2-696.9) | 780.2 (472.4-1199.7) | 1286.2 (836.4-1873.6) | 2390.8 (1613.8-3406.4) | 3703.8 (2508-5177.3) | 5567 (3763.1-7601) | 7879.3 (5504.4-10664.3) | 10746.4 (7580-14318.4) | 13784.2 (9826.8-18289.6) | 17749.2 (13042.1-22969.1) | 20753.4 (15191.3-27047.9) | 23051.3 (17062.3-29808.3) | 26238.3 (18530.3-34822.3) | 29413 (21214.7-38292.7) | 37065.8 (23909.9-50600.1) |
| Paraguay | 37.9 (11.2-73.3) | 70.8 (22.9-132) | 129.1 (41.1-246.6) | 252.1 (78.1-483.6) | 441.1 (140.7-815.4) | 751 (242.5-1400.5) | 1120.6 (367.4-2105.1) | 1578.9 (505.8-2955.9) | 2115.2 (680.8-3945.5) | 2903.9 (951.6-5345.6) | 3678 (1217-6722.7) | 4507.9 (1383.5-8079) | 5596 (1726.8-9992.7) | 6993.1 (2188.9-12203.7) | 7832.4 (2311-14089.5) |
| Peru | 119.2 (82.1-173.9) | 156.2 (100.3-230.2) | 203.8 (134.2-288.7) | 291 (188.9-436.2) | 409.3 (264.7-608.6) | 599.1 (394-885.6) | 812 (531.4-1217.2) | 1154.4 (762.3-1643.9) | 1563.6 (1049.6-2214.7) | 2217.7 (1518.5-3159.2) | 2925.9 (1903-4154.2) | 4123.4 (2851.3-5708.8) | 4943.7 (3262.7-7028.4) | 6693.3 (4518.5-9304.7) | 8739.7 (5771.3-12618.7) |
| Philippines | 397.6 (295.2-510.1) | 626.6 (466.6-802.6) | 972.9 (722.7-1251.9) | 1527.8 (1119.9-1976.9) | 2290.6 (1649.8-2966.6) | 3284.4 (2359.1-4236.1) | 4352.8 (3115.5-5618.2) | 5767.2 (4161.4-7368.5) | 7498.8 (5471.9-9516.3) | 9658.7 (7098.8-12161.3) | 12096.5 (8880.5-15113.6) | 14465.2 (10727.8-18176.6) | 16374.4 (11964.7-20682.6) | 21995.9 (15856.2-28129.1) | 23972.2 (16885.4-30711.9) |
| Poland | 23.3 (18.1-30.4) | 51.8 (40.4-67.4) | 93.1 (73.6-119.6) | 185 (144.7-235.8) | 355.8 (274.6-458) | 631.5 (485.8-804.7) | 979 (752.5-1259.9) | 1515.7 (1160.4-1932) | 2135.8 (1635.8-2741.1) | 2859.6 (2174.3-3666.7) | 4033 (3070.5-5142.9) | 6033.3 (4605.1-7767.1) | 9456.3 (7176.7-12317.1) | 13450.8 (10083.2-17511.4) | 20411 (14740.7-26813) |
| Portugal | 6.1 (3.4-9.6) | 12.3 (6.6-18.9) | 20.5 (11.3-31.9) | 34.1 (18.4-52.5) | 63.6 (33.6-97) | 98.7 (53.8-149.6) | 139.4 (75-212.8) | 190.5 (102.7-290.2) | 251.3 (136.2-382.9) | 336.3 (184.9-511.3) | 500.4 (269.7-759.8) | 762.6 (407.2-1168.8) | 1192.9 (634.4-1877.8) | 1983.6 (1058-3073.1) | 3213.3 (1703-5018.1) |
| Puerto Rico | 6.1 (1.6-11.4) | 11.7 (3.1-21.8) | 21 (5.1-39.7) | 34.8 (8.7-64.5) | 58.5 (14.9-111.4) | 95.5 (23.1-181.5) | 130.1 (32.9-247.3) | 167.5 (42.1-304.5) | 211.9 (53.8-394.4) | 280.5 (73-536.9) | 382.2 (99.2-711.3) | 463.2 (120.3-859.9) | 665.1 (169.3-1233.1) | 850.9 (218.4-1573.5) | 857.8 (217.2-1613.8) |
| Qatar | 117.3 (82.4-164.4) | 161.3 (113.9-225.2) | 252.5 (172.6-334.7) | 449.9 (316.5-615) | 761 (514.4-1067.7) | 1231.2 (806.3-1716.2) | 1901.8 (1245.3-2719.6) | 2815.2 (1845-4043.1) | 4665.6 (3188.8-6456.2) | 8693.2 (5963.9-11666.5) | 12652.5 (8819.8-16774.6) | 17082.6 (12569.7-21672.9) | 24191 (18218.3-31514.7) | 31202 (22351.1-40117.6) | 40258.6 (27171.7-53369.4) |
| Republic of Korea | 30.7 (20.2-43.9) | 51.5 (35.4-72.1) | 92 (60.7-130.7) | 142.9 (94.2-193.7) | 232.7 (157.8-315.7) | 324.6 (214.8-441) | 438.7 (302.1-606.9) | 576.4 (391.4-769.5) | 807.1 (551-1099.5) | 1230.4 (839.2-1635.7) | 2182.6 (1477.4-2971.1) | 3484.5 (2362.4-4677.5) | 5318.6 (3452.5-7384.2) | 8631.9 (5491.6-11856) | 12944.2 (8145.4-18216.3) |
| Republic of Moldova | 74.4 (46.3-105.9) | 139.9 (90.6-198.7) | 237.7 (150.6-338.3) | 498.3 (318.3-699.8) | 746.7 (483.5-1084.7) | 1229.4 (801-1765) | 1944.6 (1252.9-2788.1) | 3287.8 (2116.8-4714.5) | 4981.9 (3202.5-7007.5) | 7769.6 (4961.3-11027.7) | 9085 (5798.5-12872.5) | 10711.5 (6879.1-15414.6) | 12713.6 (7944-18517.5) | 11610.5 (6572.4-17512.4) | 11357.2 (6377.8-17601.7) |
| Romania | 48.8 (34.6-69.6) | 91 (65.5-128.4) | 155.2 (108-219) | 280.1 (198.9-397.5) | 501.4 (357-703.2) | 849.7 (597.2-1179.9) | 1323.5 (934.4-1868.1) | 1974.1 (1395.2-2843.6) | 2610.2 (1876.8-3718.2) | 3729 (2680.1-5351.4) | 5643.9 (4068.3-8044.9) | 8178.7 (5863.4-11591.2) | 11448.7 (8261.7-16144.3) | 13580.6 (9618-19295.2) | 17731.2 (11929.3-25110.9) |
| Russian Federation | 41.6 (25.3-63.5) | 93.9 (55.9-143.9) | 176 (105.6-273.9) | 335.5 (200.4-523.4) | 536.8 (319.5-847.6) | 836.1 (496.5-1331.6) | 1276.3 (749.3-2014.2) | 1861.7 (1096.6-2940) | 2497.3 (1476.4-3913.4) | 3340.2 (1984.1-5231) | 4972 (2985.4-7754) | 6792.7 (4082-10444.6) | 8207.3 (4943.6-12508.3) | 10554.5 (6190.5-16065.1) | 13428.7 (7638.9-20558.3) |
| Rwanda | 163.6 (106.5-248.7) | 292 (194.2-442.4) | 445.8 (288.3-634.9) | 841.8 (575.6-1185.5) | 1419.2 (966.6-2030.3) | 2385 (1653.5-3294.8) | 3846 (2610.2-5258.5) | 6344.3 (4471.5-8596.8) | 8933.8 (6468.4-11919) | 12439.9 (9201.3-16283.8) | 16219.2 (11987.9-21047.8) | 19031.8 (13736.3-24955.1) | 21038.5 (14463.1-28909.4) | 25245.1 (16537.2-35501.3) | 26509.7 (16041.1-37674.1) |
| Saint Kitts and Nevis | 8 (2.5-14.7) | 13 (4-24.1) | 27.1 (8.4-50.4) | 63 (21.2-116.6) | 133 (46.5-240.8) | 255.2 (92.1-469.1) | 364.8 (129.4-655.5) | 556.8 (194.9-1007.2) | 780.4 (281.9-1424.9) | 1143.3 (401.1-2069.1) | 1566.2 (569.5-2681.9) | 2031.8 (723.3-3560.3) | 2608.1 (892.2-4442.5) | 3268.3 (1125.8-5686) | 4017.3 (1378.9-6893.8) |
| Saint Lucia | 44.6 (18.9-77.2) | 74 (31.4-127.6) | 109.8 (48-189.6) | 236.4 (97-411) | 428 (176.3-730.7) | 648.9 (273-1128.5) | 878.1 (363.1-1505.5) | 1328.9 (563.4-2269.6) | 1758.3 (742.1-2985.3) | 2619.4 (1116.4-4500.3) | 3301.2 (1406.6-5568.6) | 4564.6 (1930.2-7698.2) | 7112 (2973.9-11886.2) | 11780.4 (4856.7-20240.6) | 20525.8 (8432.4-35301.1) |
| Saint Vincent and the Grenadines | 66.2 (26.6-114.7) | 96.1 (39.8-173.1) | 219.2 (88.3-388) | 405.7 (162-706.6) | 684.3 (273.2-1203.7) | 1035.2 (411.9-1841.3) | 1499.5 (602.7-2626.2) | 1821.7 (738.2-3204.7) | 2293.1 (943.2-4016.6) | 3495.7 (1387.5-6154.4) | 5272.2 (2107.8-9348.3) | 6086.9 (2409.8-10847.9) | 11089.2 (4413.7-19717.2) | 16601.5 (6566-28838.4) | 28363.2 (11331.1-50226.8) |
| Samoa | 468 (266.7-717.6) | 849 (465.7-1282.3) | 1352.7 (799.7-2022) | 2442.8 (1452.2-3486.6) | 3658.3 (2291.1-5157.9) | 5094.7 (3298.1-7022.1) | 6625.2 (4313-9037.4) | 8863.9 (5871.9-11896.7) | 11023.8 (7429.3-14651) | 13883.3 (9226-18189.3) | 17426.9 (11664-22622.6) | 20722.2 (13869.7-27048.2) | 27255.7 (17307.8-35713.3) | 33134 (22200.7-43883.8) | 46173.6 (29800.1-61115.6) |
| San Marino | 5.5 (2.7-9.1) | 8.7 (4.2-14.4) | 14.3 (6.6-23.7) | 24.2 (10.7-43) | 41.2 (18.8-73.3) | 67.9 (31.4-118.9) | 101.7 (46.8-178.4) | 154.5 (71.7-267.3) | 231.9 (108.2-404.5) | 375.8 (185.6-633.9) | 591.6 (301.5-969.4) | 860.3 (439.7-1403.3) | 1158.7 (589.2-1892.3) | 1532.6 (755.2-2514.7) | 2784.8 (1384.5-4512.2) |
| Sao Tome and Principe | 161.5 (98.9-253) | 247.5 (150.8-401.2) | 404.3 (252.7-626.7) | 747.3 (482.8-1099.6) | 1408.5 (951.4-1971.7) | 2357.7 (1703.2-3149.8) | 3428.4 (2514.3-4527.6) | 5087.8 (3827.9-6800.9) | 7326.3 (5578.5-9203.8) | 9676.2 (7626.3-12342) | 12361.9 (9597.1-15330.6) | 15094.3 (11822.7-19547.4) | 18562.5 (14513.9-23348.9) | 23461.6 (18254.7-30331.2) | 33205.3 (24997.3-43378.9) |
| Saudi Arabia | 338.5 (215.4-484) | 590.4 (366.6-902.5) | 983.5 (636.7-1439.7) | 1881.6 (1210.9-2722.4) | 2986.6 (2035.3-4111.7) | 4345.8 (3095.1-5821) | 5898.3 (4298.4-7467.1) | 7505.7 (5661.1-9605.6) | 9323.6 (7082.1-11494.3) | 11269.8 (8553.6-13975.8) | 13918.1 (10727.7-17162.7) | 17268.5 (13042.4-21651.9) | 23041.1 (17340.9-28932) | 31536.9 (23580-40155.1) | 41906.2 (30952.6-54873.3) |
| Senegal | 187.6 (130.5-258) | 340.9 (240.1-460.5) | 541.9 (388.2-735.9) | 1078.9 (786.1-1486.3) | 1929.7 (1371.3-2659.8) | 3448.7 (2428.8-4601.2) | 5001.5 (3531.2-6698.5) | 7951.5 (5829.7-10515.1) | 11568.2 (8600.1-14975.3) | 15684.8 (11699.7-19986.2) | 19978.1 (15248.3-25521.1) | 24403.3 (19046.4-30792.6) | 28682.1 (21675.6-36786.4) | 35054.3 (26176.4-44951.4) | 43359.4 (32031.3-55414.1) |
| Serbia | 44.4 (31.9-64) | 83.6 (57.7-117.4) | 174.5 (124.2-245.9) | 332.9 (238.9-464.4) | 648.9 (461.5-892.1) | 1171.8 (850.2-1624.6) | 1864 (1372.6-2577) | 2904.7 (2146-4023.4) | 4517.7 (3310.3-6221.1) | 7632.3 (5540.6-10595.7) | 9814.3 (7362.1-13430) | 17617.8 (13338.8-24477.5) | 24287.8 (18381.5-33440.3) | 29771.4 (21609.9-40690) | 35137.2 (24034.8-50203.7) |
| Seychelles | 51.7 (16.8-97.1) | 55.1 (17.7-101) | 94.4 (32.3-170.4) | 216 (72.6-398.4) | 259.3 (91-475.9) | 364.7 (125.7-674.7) | 538.6 (187.9-1007.1) | 572.3 (192.7-1034) | 832.5 (297.3-1565.4) | 1214.4 (414.1-2215.8) | 1721.8 (601.2-3315.6) | 2081.3 (726-3858.6) | 2674.3 (928.8-4940.6) | 3648.9 (1267.2-6846.6) | 4686 (1580.3-8704.5) |
| Sierra Leone | 272.4 (182.6-385) | 487.8 (327.7-686.3) | 752.4 (503.8-1021.1) | 1440.8 (989.1-2008.5) | 2407.8 (1628.6-3365.7) | 4279 (2904.4-5917.5) | 6103.9 (4216.8-8270.7) | 9424 (6682.9-12592.9) | 13500.6 (9631.9-17779.5) | 17150.8 (12709.5-22450.7) | 21648.9 (16930-26992.6) | 25173.7 (19897.2-31009.2) | 28384 (22988.1-34480.3) | 33543.9 (26234.3-40702.6) | 42690 (32892.8-53001.2) |
| Singapore | 25.3 (13.2-38.1) | 36.3 (19.2-55) | 74.3 (39.3-111.2) | 93.1 (50.2-140.8) | 227 (121.6-342.2) | 293.5 (156.4-434.4) | 436.9 (230-651.1) | 635.2 (331.4-961.4) | 799.6 (429.4-1185) | 1116.2 (593-1663.5) | 1496.8 (803.5-2265.2) | 2046.8 (1089.8-3122.2) | 2456 (1320.3-3750.6) | 3210.3 (1690-5029.8) | 4664.5 (2536.4-7318.7) |
| Slovakia | 31.2 (22.3-42.1) | 56.3 (39.5-74.6) | 98 (68.2-134.5) | 181.4 (124.3-250.8) | 338.7 (236.5-461) | 623.8 (448.3-821.6) | 1024.5 (737.6-1349) | 1681 (1188.7-2228.1) | 2393.4 (1749.7-3121.6) | 3579.4 (2660.3-4599.8) | 5393.9 (3995.7-6854.2) | 8102 (5923.8-10308.3) | 12589.8 (9220.6-16171.9) | 15591.6 (11150.6-20060.9) | 15707 (10679.7-20908.9) |
| Slovenia | 8.3 (5.6-11.6) | 14.8 (10.4-19.8) | 24.5 (17.1-33) | 54 (36.6-74.4) | 98.1 (66.2-136.4) | 164.7 (111.4-225.7) | 243.5 (166.8-334.8) | 399.9 (273.3-542.9) | 606.8 (430.7-807.7) | 991.1 (703.4-1325) | 1428.4 (1014.4-1926.5) | 2191.9 (1553.9-2909.3) | 3397 (2404.4-4498.8) | 4455.1 (3100.5-5879.5) | 6764.3 (4522.7-9179.9) |
| Solomon Islands | 649.6 (453.1-899.6) | 1265.7 (844.8-1751.3) | 2245.8 (1605.4-3058.1) | 4336.5 (3021.3-5826.1) | 6973.4 (4853.3-9842.5) | 9963.7 (7002.6-13496.1) | 13073.5 (9766.7-17725) | 16430.6 (12476-21867.3) | 20628.2 (15889.8-27024.8) | 25848.5 (20504.5-33179.2) | 32288.3 (25609-40332.6) | 37658.1 (29564.4-45905.7) | 45104.6 (36710.5-54545.3) | 51465 (40518.4-61678) | 70272.8 (54320.8-84517.9) |
| Somalia | 219.2 (141.3-311.9) | 410.9 (257.9-640.6) | 642.6 (392.9-965) | 1386 (816.8-2152) | 2227.9 (1371.3-3650.5) | 3678.9 (2337.1-5552) | 5891.6 (3905.9-8696.8) | 9060.1 (6249.3-12632.3) | 11996.7 (8415.3-16187.6) | 15394.6 (10884.6-20716.6) | 18357.5 (12886.7-24965.6) | 18356.8 (13009.7-24696.7) | 18666.7 (13559.4-24848.8) | 15736.2 (10309.8-22399.3) | 7848.2 (4874.4-11786.9) |
| South Africa | 72.9 (52.5-95.6) | 154.6 (115-204.6) | 256.5 (189.9-334.9) | 427.7 (311.6-568.1) | 660.2 (462.9-915) | 1108.2 (792.6-1474.5) | 1595.4 (1155.1-2099) | 2412.2 (1738.2-3188.1) | 3230.4 (2357.9-4230.2) | 3751.9 (2740.8-4875.9) | 4953.2 (3579.6-6441.7) | 7554 (5276.3-9636.5) | 10402.7 (7348.8-13487) | 14055.3 (9820.9-18366.7) | 14501.1 (9818.4-19572.5) |
| South Sudan | 198.5 (132.6-295.7) | 352.7 (219.3-533.3) | 563.9 (367.4-860.9) | 1134.9 (730.7-1715.1) | 1874.3 (1229.9-2737) | 3143.3 (2053.4-4680.5) | 5117.6 (3457.3-7333.8) | 8062.8 (5485.2-11785.6) | 11206.5 (7653-15652.1) | 14173.7 (10177.1-19511.1) | 16616.8 (12040.5-22778) | 17784.6 (13289.6-23529.7) | 17999.3 (13131.9-23130.6) | 20173.8 (15091.1-25268) | 20560.1 (14099-27146.1) |
| Spain | 6.9 (4.2-9.8) | 13.9 (8.7-20) | 22.6 (14.3-32.4) | 36.3 (22.7-51.3) | 64.3 (40.4-92.2) | 104.6 (65.6-149.2) | 152.8 (96-215.5) | 215.8 (136.9-302.6) | 280.2 (178.3-395.6) | 369.7 (232.5-518.4) | 539.3 (340.9-760.5) | 792.3 (491.1-1137.2) | 1267.4 (794.8-1801.2) | 2060 (1275-2974.9) | 3269.8 (1932.2-4781) |
| Sri Lanka | 143.4 (76.3-235.7) | 209.8 (109.6-347.1) | 325 (169.6-570) | 505.9 (253.4-868) | 836.8 (418.1-1415.2) | 1325.4 (667.1-2297.7) | 1791.5 (880.8-2969.9) | 2631.6 (1309.3-4472.6) | 3888.5 (2019.4-6421.9) | 6089.2 (3189.6-9985.8) | 7868.9 (4091.3-13110.7) | 11504.1 (6031.2-18763.1) | 14964.5 (7586.4-25070.5) | 18310 (9022-30388.5) | 28682.4 (13899.9-48278.6) |
| Sudan | 471.1 (286.4-698.8) | 715.6 (400.3-1062.1) | 1115.2 (649.5-1625.1) | 1873.6 (1159.8-2721.7) | 2986.9 (1951.6-4312.8) | 4738.7 (3264.5-6643.9) | 6882.2 (4744.9-9485) | 9700.4 (6899.5-13566.3) | 13361.6 (9616.8-18201.2) | 17882.8 (13596-24062.7) | 22752.5 (17143.1-29597.3) | 26925.6 (19768.2-34866.9) | 32802.4 (24524.4-41345) | 41530.5 (30162.3-53299) | 52265.9 (35384.6-67640.9) |
| Suriname | 107.4 (47.5-180.6) | 219.5 (100.4-363.6) | 381 (170.7-632.6) | 632.2 (299.6-1035.7) | 1110.9 (526.8-1794.5) | 1793.2 (850.4-2899.8) | 2702.2 (1217.8-4391.3) | 3446.6 (1567.8-5664.2) | 4499.2 (2035.2-7461.7) | 4969.6 (2304.6-8201.6) | 5952.5 (2840.5-9791.3) | 7338.7 (3526.4-11924.9) | 9356.7 (4311.1-15533.1) | 10674 (4900.4-18406.7) | 10768.8 (4637.7-19242.4) |
| Sweden | 1.9 (0.5-3.8) | 2.7 (0.7-5.3) | 4.5 (1.3-8.5) | 9.1 (2.7-17.4) | 17.7 (5.2-33.6) | 33.9 (9.9-63.9) | 54.9 (16-106.1) | 88.8 (26-167.8) | 136.3 (39.5-259) | 191.6 (55.3-358.7) | 282.8 (81.1-531.9) | 466.2 (136.7-877.4) | 806 (234.9-1525) | 1370.9 (400.6-2607.5) | 1690.4 (488.6-3306.8) |
| Switzerland | 4.4 (2.8-6.3) | 6.8 (4.3-9.8) | 11.1 (7.1-15.8) | 22.1 (13.9-31.6) | 37.1 (23.6-53.4) | 62.3 (39.5-88.1) | 97.4 (61-138.7) | 149.8 (94-213.8) | 223.7 (143.2-313.9) | 332.7 (211.2-465.7) | 508.3 (323.5-708.7) | 832.9 (513.9-1202.4) | 1542.3 (943.1-2203.8) | 2602.4 (1564.6-3792.7) | 3854.3 (2242.3-5566.6) |
| Syrian Arab Republic | 312.7 (207.8-463.1) | 445.3 (299.6-628.2) | 640 (422.7-916) | 1041.6 (672.3-1510.1) | 1837.1 (1210.9-2675.4) | 2910.1 (1889.6-4230.3) | 4185.2 (2701.6-6059.7) | 6263.6 (4140-8893.6) | 8125.2 (5457.7-11491.4) | 10327.9 (6954.6-14499) | 13772.9 (9355.3-19165.7) | 19464.7 (13764.4-26164.4) | 32036.6 (22931.2-42594.4) | 42549.7 (31046.9-57109.8) | 53736.9 (38325.4-70687.8) |
| Taiwan (Province of China) | 31.5 (24.1-40) | 53.7 (40.9-69) | 96 (74.1-121.5) | 166.8 (128.8-213.8) | 253.1 (198.1-319.4) | 363.3 (276.6-456) | 459.1 (359.2-578.4) | 590.2 (459.5-745) | 767.5 (598.6-969.4) | 1002.2 (778.1-1256.2) | 1555.7 (1202.1-1963.9) | 2125.1 (1623.7-2706.9) | 3099.9 (2338.9-3929.9) | 4294.2 (3174.8-5560.7) | 4143.3 (2856.8-5404) |
| Tajikistan | 178.9 (130.5-241.7) | 347.8 (245.8-468.2) | 535 (380.5-710.4) | 918.9 (661.3-1216.4) | 1538 (1119-2026.5) | 2585.8 (1908-3375.3) | 4293 (3223.2-5458.8) | 6998 (5296.7-8805.9) | 11262.4 (8642.5-13988) | 16844.2 (13054.7-20980.5) | 22910.8 (17757.3-28438.2) | 29829.9 (22675.4-37227.1) | 31845.4 (23540.1-39856.7) | 42000.4 (30829.6-53257.7) | 54646.9 (38354.7-70384.7) |
| Thailand | 168 (116.4-242.5) | 307.2 (211.4-435.1) | 461.9 (317-651.1) | 648.2 (435.9-937.5) | 827.9 (557.8-1175.6) | 1107.8 (745.6-1588) | 1334.2 (893-1887.4) | 1742.2 (1210-2451.2) | 2305.8 (1589-3174.7) | 3150.4 (2225.3-4224.1) | 4282.3 (3052.1-5886.2) | 5685.7 (4028.2-7588.3) | 6359.8 (4487.1-8639.8) | 8571.4 (6015.3-11753.6) | 10648.8 (6969.9-14622.8) |
| Timor-Leste | 358.4 (202.4-554) | 518.1 (277.3-800.5) | 822.5 (456.3-1247.8) | 1444.1 (814.1-2107) | 2404.3 (1415.9-3545.4) | 3976.5 (2456.4-5633.1) | 5913.6 (3595.8-8132) | 8265.3 (5038.8-11448) | 11232.3 (6770.5-15324.6) | 15189.7 (9440.5-20745.3) | 19473 (12201.7-26250.6) | 24031.4 (15252.3-32258.7) | 28767.8 (17880.4-38776.8) | 34919.1 (21990-47557.8) | 43487.1 (26733.4-58663.8) |
| Togo | 221.7 (147.5-317.7) | 411.7 (276.4-585) | 650.8 (447.1-909.1) | 1341.2 (899-1860.9) | 2371.8 (1595.9-3271.8) | 4212.9 (2886.4-5861.9) | 6092.9 (4205.7-8372.1) | 9264.4 (6600.6-12807.4) | 13197 (9466.4-17540.5) | 16801.9 (12421.4-22128.2) | 21400.6 (15864.3-27384.1) | 24953.4 (19263.8-31886.7) | 27889.9 (21383.8-35225.4) | 32365.2 (25089-41743.5) | 41144.8 (30101.9-52564.8) |
| Tokelau | 55.4 (0.5-133.8) | 94.3 (0.8-234.5) | 117.2 (1-315.1) | 188.5 (1.5-502.8) | 294.6 (2.3-778.4) | 410.4 (3.4-1087.1) | 553.3 (4.4-1407.5) | 719.7 (6.1-1833.5) | 924 (7.4-2327.5) | 1119.5 (9.8-2843.4) | 1423.8 (12.8-3506.1) | 1781 (15.4-4510.3) | 2222.3 (19.5-5503.9) | 2985.8 (25.1-7376) | 4239.7 (35.9-10285.4) |
| Tonga | 185.8 (102.6-306.2) | 347.4 (199.8-566.9) | 527.5 (306.5-863.5) | 958.8 (543.4-1525.4) | 1458.9 (840.8-2316.8) | 2124.2 (1194.4-3248.3) | 2889 (1654.6-4409.7) | 3790.4 (2159.8-5597.8) | 4876 (2940-7082.5) | 6301.5 (3761.2-8982.4) | 8142.1 (4791.6-11626.5) | 9893.6 (6212.4-13929.5) | 12714.2 (7658-18249) | 15765.6 (9416.8-22631) | 21791.4 (13591.1-31056.5) |
| Trinidad and Tobago | 104.1 (36.2-196) | 162 (58.3-301.1) | 329.8 (117.3-613.6) | 500 (179.2-919.2) | 863.1 (299.7-1597.5) | 1254 (434.9-2301.7) | 1725.4 (616.7-3229.8) | 2348.4 (817.6-4341.6) | 2829.9 (1003.7-5223.5) | 4288.7 (1532.4-7951.4) | 5324.6 (1905.2-9747.2) | 6859.7 (2382-12446.8) | 7910.4 (2758-14390.1) | 10678.4 (3713.7-19097.3) | 14870 (5236.3-26740.3) |
| Tunisia | 99.5 (61.7-153) | 172.7 (105.9-260.1) | 260.7 (162.2-388.6) | 456.1 (281.2-686.6) | 727.2 (446.8-1084.1) | 1102.1 (680.8-1668.6) | 1757.9 (1110-2669.4) | 2590.4 (1594.8-3828.4) | 3737.1 (2361.7-5501) | 5333.9 (3477.2-7696.9) | 7358.9 (4768.6-10528.5) | 11084.4 (7221.7-15847) | 14791.3 (9272.7-20660.5) | 19690.4 (12181.8-27142.7) | 22595.3 (13272-32732.8) |
| Turkey | 68.6 (50.4-93.4) | 108.5 (78.6-143.8) | 182.4 (126.6-237.7) | 317.6 (223.4-413.4) | 564.7 (394-731.5) | 953.1 (670.9-1260.7) | 1409.4 (977.3-1904.6) | 2118.3 (1484.5-2788.2) | 2991 (2130.6-3792) | 4169.9 (3000.9-5324) | 6434.5 (4719.6-8145.6) | 9032.3 (6741.4-11428.3) | 13762.6 (10417.8-17152.8) | 17525.5 (12959.8-22267.2) | 19980.9 (13619.4-26419.2) |
| Turkmenistan | 168 (99-264.3) | 271.9 (158-429.3) | 476.1 (276.7-738.9) | 878.6 (516.8-1380.1) | 1523 (884-2397.8) | 2377.3 (1369.8-3731.2) | 3772.1 (2201.4-5949.2) | 6007.3 (3506.5-9310) | 8163.4 (4784.4-12617.6) | 12127.3 (7161-18633.6) | 14404 (8498.5-22003.6) | 18855.1 (11070.9-28840.8) | 18275.5 (10742-27927.9) | 21633.5 (12459.6-33252.7) | 22867.1 (12500.7-36613.8) |
| Tuvalu | 181.7 (98.7-289.5) | 328.6 (175.5-543.4) | 533.5 (288.4-860.6) | 917.6 (507.2-1535.1) | 1324.9 (735.9-2128.9) | 1770.8 (984.1-2853.8) | 2227.9 (1265.8-3643.3) | 2850.7 (1608.1-4529.4) | 3412.5 (1962.4-5339.7) | 4206.6 (2459.7-6525.4) | 5180.8 (3032.7-7846.3) | 6196.4 (3550-9519.7) | 8031.7 (4664.2-12628) | 9510.2 (5530.8-14766.6) | 13328.1 (7625.7-21408.4) |
| Uganda | 178.2 (119.1-248.5) | 317.1 (211.6-450.4) | 474.1 (314.2-651.5) | 904.5 (599.2-1254.2) | 1431.6 (979-1999) | 2340.6 (1593.7-3163.1) | 3690.7 (2601-4937.3) | 5824.1 (4205.7-7668) | 8248.2 (5926.6-10835) | 11094.3 (8289.2-14312) | 14119.6 (10604.7-18095.7) | 16389.9 (12468.1-21367.5) | 17673.9 (12961.3-23459.4) | 21250.1 (15021-28704) | 21169.7 (13959.6-30147.4) |
| Ukraine | 98 (52-152.2) | 197.9 (110.6-309) | 311.7 (166.3-500.1) | 524.8 (276-875.1) | 844 (441.6-1409.2) | 1376.3 (723-2251.6) | 2250 (1205.1-3700.7) | 3669.8 (1993.9-5939.8) | 5305.5 (2968.8-8393.9) | 7624.3 (4360.7-11733.5) | 10801.1 (6227.1-16957.1) | 14470.8 (8374.3-22790.3) | 20235.5 (11541.9-32152.5) | 28945.8 (16282.5-45840.6) | 31608.5 (17357.7-52177.6) |
| United Arab Emirates | 157.6 (105.7-215.1) | 143.1 (89.1-204.5) | 162.9 (106.2-225.7) | 307.7 (206.7-412.1) | 540 (360-738.6) | 952.9 (635-1293.1) | 1598.1 (1111.3-2131.7) | 2903.7 (1999-3906.8) | 3193.7 (2260.8-4282.6) | 15709.1 (11271.2-20937.8) | 19059.7 (13633.1-24382.4) | 17392.1 (12927.6-22060.8) | 20114.3 (14700-25690.8) | 24196.3 (17374.7-32203.1) | 38491.3 (27142-51589.8) |
| United Kingdom | 7.7 (5.2-10.8) | 14.3 (9.4-20) | 29.4 (19.3-41.3) | 54.3 (35.2-76.4) | 94.6 (60.8-132.8) | 153.9 (98.7-216.7) | 211.1 (132.9-295.6) | 299.3 (190.6-423) | 391.7 (249.2-553.1) | 537 (344.4-761.8) | 776.5 (495-1093.3) | 1130.3 (717.6-1562.1) | 1737.4 (1088.6-2411.8) | 2587.4 (1616.3-3619) | 4271.8 (2631.3-5988.3) |
| United Republic of Tanzania | 220.7 (143.8-308.8) | 379.4 (258.3-526) | 552.2 (383.6-773.8) | 955.5 (658.8-1323.7) | 1466.4 (987.3-2059) | 2428.1 (1718.5-3353.5) | 3886.6 (2725.9-5146.1) | 6509.8 (4604.2-8816.6) | 9721.7 (7094-12840.1) | 13333.7 (9971.1-17537.3) | 16900.6 (12808.7-21281.1) | 19067 (14831.6-24635) | 20517.7 (15250.6-26597.3) | 24700.2 (17982.6-32107.1) | 26008.9 (17499.5-34574.4) |
| United States of America | 8.8 (4.3-14.2) | 16.6 (8.2-26.9) | 29.6 (14.6-47.8) | 54 (26.1-86.7) | 93 (45.6-150.8) | 150.7 (73-242) | 219.7 (106.4-353.6) | 292 (139.8-469.1) | 351.6 (168.2-562.5) | 450.9 (215.4-724.3) | 608.4 (292.9-977.9) | 847.9 (406.3-1354.9) | 1268.7 (594.6-2071.7) | 2007.3 (930.7-3330.4) | 3372.2 (1530.3-5683.5) |
| United States Virgin Islands | 27.2 (10.7-52.4) | 39.6 (16.5-69.6) | 62.2 (26.3-114.8) | 61.7 (26.9-106.5) | 181.6 (76.5-317.2) | 139.3 (60.4-240.8) | 294.4 (127.5-504.4) | 448.7 (197.4-761.2) | 426.6 (185.3-730.8) | 717.1 (315.7-1222.7) | 872.6 (387.9-1466.7) | 1163.3 (513.9-1943.7) | 1737.8 (787.3-2895.8) | 3161.4 (1408.1-5256.3) | 7919.1 (3590.6-13155.7) |
| Uruguay | 18.5 (7.1-33) | 29.6 (11.5-52) | 49.9 (19.1-91.3) | 102.7 (39-179.6) | 164.1 (62.6-291.2) | 253.6 (100.4-456.8) | 376.4 (148.3-670.3) | 521.9 (206.1-918.8) | 716.1 (281.7-1264.1) | 972.6 (375.8-1688.9) | 1264.9 (497.8-2175) | 1665.4 (638-2854.1) | 2291.4 (864.7-3926.6) | 3383.4 (1238.1-5830.8) | 4793.8 (1776.2-8351.6) |
| Uzbekistan | 239.4 (174.5-317.7) | 393.4 (288.9-501.6) | 582.9 (420.3-753.1) | 940.7 (680.6-1228.3) | 1620.3 (1175-2116.6) | 2810.7 (2013.4-3677.1) | 4478.9 (3228.5-5835.1) | 7979.4 (5825.8-10346.4) | 10572.5 (7622.2-13796.2) | 16586 (12051.9-21428) | 22979.6 (16693.4-29566.2) | 29362 (21388.4-37629.7) | 30741.7 (22037.2-39670.2) | 36004.7 (25197.2-46572.9) | 51386 (35708.4-67064.9) |
| Vanuatu | 1035.6 (700.2-1386.9) | 1827.1 (1232.5-2394.8) | 2933.9 (2142.3-3804.5) | 5076.4 (3634.8-6397.2) | 7288.8 (5239-9229.9) | 9766 (7394.5-12359) | 12292.5 (9449.6-15335.3) | 15726.5 (12189.3-19528) | 18981.3 (15138.8-22990) | 23321.6 (18857-28154.1) | 28035.3 (22425.8-33826.8) | 33326.1 (26631.4-40210.1) | 41677.6 (33482.5-49623.1) | 49167.6 (37415.5-59846.1) | 67844.6 (45979.8-85169.6) |
| Venezuela (Bolivarian Republic of) | 76 (41.2-120.5) | 124 (69.4-195) | 205 (114.2-329) | 353.9 (190-586.1) | 567.1 (302.3-908.4) | 887.3 (480.9-1465.8) | 1281.1 (691.3-2072.7) | 1726.8 (943.7-2750.5) | 2192.9 (1216.3-3476.7) | 2971.2 (1633.7-4622.5) | 3841 (2146.8-6001.8) | 4945.9 (2731.2-7583.8) | 6204 (3413.7-9593.4) | 7249.4 (3927-11219.6) | 7258.6 (3861.4-11647.9) |
| Viet Nam | 100.8 (65.6-146.3) | 189 (121.8-272.4) | 373.5 (240.4-541.4) | 730.8 (466.2-1086.3) | 1280.2 (822.1-1833.8) | 2145.6 (1390.2-3032.6) | 3165 (2101.4-4242.8) | 4431.1 (3003.8-5954.9) | 6451.4 (4458.4-8508.6) | 9403.6 (6690-12185.2) | 12995.7 (9081.2-16761.5) | 16155.5 (11390.8-20514.4) | 19142.4 (13625.2-24412.8) | 23535.9 (16221-30747.6) | 27822.5 (18525.8-37112.7) |
| Yemen | 262.3 (158.5-412.4) | 453.7 (275.6-681) | 869.8 (553.3-1280.9) | 1755.5 (1147.9-2544.4) | 3041.2 (2010.8-4548.6) | 4837.5 (3217.1-6904.6) | 6971.5 (4741.3-10077.6) | 10011.7 (7010.6-13866.2) | 13635 (9547.6-18776) | 17870.9 (12366.3-24119.1) | 22702.6 (15922-30636.2) | 27580.5 (19533.4-36528.2) | 32972.1 (21955.8-43963.9) | 40640.7 (27781.8-54386.7) | 50541.7 (32995.5-69115.4) |
| Zambia | 193.8 (119.3-293.5) | 369.5 (238.9-528.9) | 584.2 (375.1-857.6) | 1197 (767.6-1732.9) | 2035.8 (1338.1-3011.1) | 3241.6 (2143.9-4572.7) | 5168.7 (3459.7-7159.6) | 8103.4 (5728.4-10646.9) | 11136 (7965.4-14229.5) | 15353.2 (10948.8-19473.3) | 19946.7 (14933.4-25303.4) | 23297.9 (17474-29257.7) | 26262.8 (19293.8-33634.9) | 25487 (18156.5-33344.3) | 15564.8 (9890.9-22467.1) |
| Zimbabwe | 209.3 (139.6-303.7) | 307.3 (200.7-452.8) | 531.6 (335.6-813.2) | 1006.9 (668.8-1524) | 1999 (1321.3-2910.7) | 3917.7 (2753.9-5627.6) | 5847.7 (4059.7-8085.2) | 8245.1 (6058.1-11307) | 10939.5 (8353.5-14271.6) | 14352.9 (11011.6-18066.2) | 18916 (14749-24023.5) | 23164.1 (18061.2-28444.4) | 27215 (21069.2-33473.8) | 21822.1 (15848.8-28747.4) | 13241.7 (8710.2-18351.9) |

Supplementary Table 8 Deaths of CVDs Attributable to PM2.5 Pollution by Age Distribution and 204 Countries and Territories, 2021

| Location | 25-29 years | 30-34 years | 35-39 years | 40-44 years | 45-49 years | 50-54 years | 55-59 years | 60-64 years | 65-69 years | 70-74 years | 75-79 years | 80-84 years | 85-89 years | 90-94 years | 95+ years |
| --- | --- | --- | --- | --- | --- | --- | --- | --- | --- | --- | --- | --- | --- | --- | --- |
| Afghanistan | 636 (452.6-921.3) | 991.5 (691.8-1427.8) | 1623.9 (1133.9-2352.1) | 3091.8 (2148.4-4582.8) | 5227.1 (3653.9-7515.1) | 7892.1 (5524.8-10894.2) | 10695.9 (7569.9-14174) | 14401.1 (10751.4-18614.5) | 19349 (14664.1-24508.7) | 25059.8 (19849.4-31441.2) | 30785.1 (24007.9-37881.2) | 35058.1 (27322.9-43742.6) | 41493.5 (31067.1-51559.2) | 46172.5 (33734.7-58754.1) | 40310.1 (27019.9-54206.4) |
| Albania | 45.3 (27.3-69.4) | 90.1 (57.6-141.3) | 172 (102.2-264.2) | 258 (162.6-400.4) | 413.3 (259.7-643.1) | 625.5 (386.9-983.8) | 1072.8 (645.2-1647.1) | 1649 (1034.1-2523.9) | 2529.2 (1553.7-3880) | 4134.8 (2622-6224.9) | 8039.7 (5176.2-12163.1) | 13322.3 (8382.3-20033.4) | 19718.7 (12299-29804.7) | 27022 (16942.1-40282.8) | 36926.6 (22546.7-54566.7) |
| Algeria | 115.3 (71.3-167.4) | 189 (118.3-266.1) | 309.5 (197-452.4) | 521 (341.8-738.6) | 817.2 (536.5-1153.4) | 1247.1 (815.9-1785.5) | 1737.6 (1155.2-2468.7) | 2635.2 (1737-3656.4) | 3424 (2212.6-4682.8) | 4646.3 (3018.4-6487.5) | 7199.5 (4740.5-10122.5) | 9929.4 (6466.2-13410.3) | 33111.1 (22700-45761.7) | 43041.2 (29470.3-58875) | 35188 (22894.7-46757.8) |
| American Samoa | 56.9 (6.2-138.7) | 115.4 (12-278.1) | 179.9 (17.9-431.3) | 323 (30.3-755.5) | 483.5 (48.7-1131.4) | 660.5 (63.4-1549.4) | 808.6 (85.1-1836.4) | 1057.1 (114.5-2411.2) | 1280.8 (131.7-2975.3) | 1586.2 (166.4-3596.4) | 2016.4 (215.3-4611) | 2444.8 (246.5-5427.6) | 3297.3 (348.1-7361.9) | 4025.5 (428.2-8914.8) | 5802.8 (580.1-13402.6) |
| Andorra | 5.5 (3-8.9) | 9.2 (4.9-15.1) | 16.1 (8.3-27.6) | 28.6 (13.6-48.8) | 50.3 (25.1-83.4) | 80.6 (40.7-144.4) | 116.9 (58.5-197.2) | 170.7 (83.9-279.5) | 248.2 (129.3-422.6) | 382.2 (197.8-633.1) | 586.2 (309.2-945.7) | 872.3 (450-1440.5) | 1352.9 (695.5-2181.3) | 1991.1 (976.6-3169) | 2763 (1380.5-4637) |
| Angola | 101 (61.1-146.7) | 197.4 (123.5-284) | 328.3 (206.2-487.7) | 699.2 (414.1-1002.5) | 1279.2 (776.2-1876.5) | 2160.2 (1366.6-3063.5) | 3413.6 (2143.4-4890.5) | 5394.8 (3441.5-7511.7) | 7987 (5146.4-10780.1) | 11045.1 (7316.5-15125.9) | 13589.8 (9297.4-18244.3) | 16155.3 (10836.5-21932.5) | 17018.6 (11076.6-23151) | 18941.8 (12299.9-26011.3) | 18247 (10748.1-26091.7) |
| Antigua and Barbuda | 23.7 (9.3-41.9) | 33.4 (12.5-58.5) | 56.4 (22.5-99.4) | 121.3 (45.7-215.4) | 248.1 (88.2-444.7) | 470.4 (167-837) | 773.3 (282-1376) | 1199.9 (429.1-2122.9) | 1933.8 (696.2-3459.9) | 2915.2 (1073.4-5366.8) | 3777.2 (1357.2-6750.1) | 4582.9 (1646.6-8224.7) | 5844.7 (2091.2-10225.9) | 7737.4 (2896.2-13984.7) | 11153.7 (4208.2-19503.9) |
| Argentina | 30.4 (15.8-48.7) | 44.7 (22.9-71.1) | 72.9 (37.7-115.6) | 126.2 (63.8-197) | 222.5 (117-350.5) | 375.4 (195.4-590.7) | 562 (293.2-889.9) | 817.7 (419.3-1292.3) | 1096.7 (569.6-1768.4) | 1396.6 (721-2222.5) | 1775.4 (926.7-2797.3) | 2245.9 (1165.3-3606.9) | 3273.8 (1710.8-5301) | 4383 (2220.2-7049.6) | 5579.5 (2734.9-9168.3) |
| Armenia | 81.4 (57.2-109.8) | 157.6 (109.9-210.3) | 300.8 (210.3-406.4) | 648.1 (450-886.2) | 1118.9 (778.6-1516.5) | 1751.9 (1220.9-2359.6) | 2715.8 (1896.3-3644.1) | 3687.7 (2570.4-4923.5) | 5440.5 (3764.8-7338.4) | 8048.7 (5588.2-10811.7) | 12174.2 (8550.5-16033.7) | 16525.1 (11378.5-21823.5) | 21460.1 (14595.8-28611.5) | 27732.9 (18473.7-37026.8) | 36750.3 (24022.9-49794.1) |
| Australia | 5.6 (3.3-8.1) | 11.4 (6.7-16.8) | 23.5 (14.1-34.8) | 44 (25.4-64.7) | 75.5 (45-112.6) | 103.9 (61-151.8) | 141.6 (83.7-205.8) | 188.1 (109.8-279.5) | 245 (147.2-358.9) | 355.4 (211.3-515.5) | 555.6 (326.1-812.9) | 890.6 (523.4-1313.6) | 1563.1 (919.5-2291.5) | 2781.7 (1620.3-4107.5) | 4940.7 (2833.8-7437.8) |
| Austria | 7.4 (5.1-10.3) | 13.6 (9.5-18.5) | 26.7 (18.4-36.3) | 43.2 (29.5-59.2) | 84.9 (59.9-114.7) | 143.2 (100.9-191.1) | 238.5 (168.1-321.5) | 380.1 (266.6-508.6) | 527.1 (370.5-697.7) | 817.5 (562.8-1097.9) | 1286.8 (898.5-1730.8) | 1741.3 (1207-2368.6) | 3224.4 (2188.9-4356) | 4819.1 (3212.7-6572.1) | 7258.5 (4741.5-10216.3) |
| Azerbaijan | 88.2 (45.8-147.2) | 168.3 (86.7-270.6) | 270.6 (140.4-430.2) | 486.4 (252.3-769.2) | 880.6 (460.1-1384.7) | 1472.6 (771.2-2314.9) | 2450.4 (1281.1-3846.3) | 4199.6 (2240.8-6507.9) | 6640.3 (3518.8-10355.3) | 10614.8 (5710.2-16372.3) | 13997 (7685.2-21401.7) | 19424.7 (10693.6-29937.4) | 20870.4 (11418.2-32079.1) | 23481.4 (12631.2-35446.8) | 26674.4 (13723-40718.9) |
| Bahamas | 46.1 (16-94.5) | 95.8 (32.6-191.4) | 167 (55.5-327.8) | 317.7 (112.8-620.3) | 479.4 (164.7-945.8) | 832.5 (295.8-1617) | 1077 (385.7-2057.4) | 1353.7 (497.2-2661.9) | 1846.3 (667.9-3504.6) | 2231.4 (793.6-4329.1) | 2848.9 (1010.2-5441.7) | 3937.4 (1411.3-7508.5) | 4813.4 (1735.4-9168.1) | 6286.7 (2264.3-12171.9) | 7585.9 (2690.2-14433.5) |
| Bahrain | 212.2 (155.1-278.4) | 311.2 (233.8-406.6) | 384.6 (292.5-496.4) | 591.5 (450.3-761.3) | 946.7 (689.9-1230.4) | 1455.8 (1089.6-1847.7) | 2303.9 (1730.5-2941.2) | 3330 (2467-4300.5) | 5170.8 (3882.2-6708.4) | 9090.1 (6903.7-11481.3) | 15893.1 (12123.3-19758.3) | 21985.7 (17276.2-26890.2) | 29527.1 (22703.6-36162.9) | 38658.6 (30733.4-47243.5) | 52236.8 (39168.3-64625) |
| Bangladesh | 323.6 (226.8-446.5) | 611.9 (436.7-805.2) | 839.8 (591.1-1109.1) | 1395.7 (968.5-1886.1) | 2244.5 (1562.7-2911.2) | 3915.9 (2860.9-5138.7) | 4745.9 (3360.6-6262) | 7740.7 (5622.1-10013.3) | 10145.7 (7521.1-12974.3) | 12702.6 (9755.6-16040.5) | 14779.2 (11247.6-18484.2) | 24232 (18677.5-29387.7) | 28218.6 (22011.2-34318.1) | 34122.9 (25886.5-41784) | 42274 (31338.9-51883.5) |
| Barbados | 35.1 (14.8-60.6) | 69.3 (29.1-122) | 107 (46.5-186.3) | 198.5 (82.8-343.1) | 364 (153.1-650.6) | 532.9 (222.7-932.8) | 856.7 (352-1499.1) | 1279 (535.6-2214.6) | 1939.5 (830.8-3336) | 2871.1 (1176.5-4934.6) | 3743.3 (1545.3-6390.4) | 4947.5 (2089.6-8309.2) | 7804.9 (3249.6-13329.3) | 10403 (4439.7-17810.7) | 13844 (5912.5-23389.2) |
| Belarus | 51.4 (34.9-70.1) | 141.7 (96.8-200.9) | 278.2 (189-386.1) | 549.4 (371.6-771.7) | 975 (653.5-1372.1) | 1487.2 (997.3-2071.1) | 2285.1 (1528.4-3191.8) | 3559.2 (2412.9-4919.5) | 4967.2 (3375.8-6863.9) | 6687.5 (4571.7-9214.3) | 9408.6 (6465.7-12957.7) | 12550 (8679.5-17237.9) | 16152.6 (10984.1-22398.3) | 22144.2 (15141.8-30520.2) | 29227.2 (19863.5-40577) |
| Belgium | 6.5 (4.4-8.8) | 12.1 (8.3-16.5) | 22.9 (15.8-31.3) | 46.6 (31.9-62.1) | 80.5 (55.9-107.1) | 125.8 (87.5-171.7) | 183.7 (127.6-242.2) | 275.5 (187.4-369.3) | 400.6 (279.2-532.9) | 587.4 (411.5-782.7) | 852.9 (587.1-1118.5) | 1211.7 (816.5-1633.6) | 1807.3 (1200.7-2439.4) | 2595 (1688.9-3561.3) | 3852.6 (2376.9-5354.2) |
| Belize | 71.3 (36.1-113.3) | 90.6 (44.1-143.1) | 182.3 (92.6-289.1) | 348 (174.5-558.5) | 583.7 (291.9-951.8) | 822.1 (423.5-1304.7) | 1327.7 (684.6-2109.7) | 2226.1 (1103.4-3551.5) | 2302.4 (1145.1-3712.6) | 3245 (1620.6-5282.5) | 4442.3 (2170.2-7172.8) | 5386.6 (2648.4-8644.1) | 7116.5 (3527.2-11384.2) | 9203.8 (4548.1-14640.2) | 12626.3 (6137.4-20011.1) |
| Benin | 140.7 (98.1-193.6) | 268.9 (191.3-366.2) | 449.5 (314-585.6) | 850.6 (600.9-1133.5) | 1527.9 (1082.6-2040.3) | 2828 (2051.7-3812.2) | 4158.3 (2959.3-5463.3) | 6802.6 (5022-8866.1) | 10247.6 (7790.1-13186) | 13816.8 (10603.8-17609.7) | 18352.8 (14549.4-22477.6) | 22025.4 (17521.2-27381.3) | 24805.9 (19737.2-29733.4) | 29819.4 (23258.4-37020.8) | 36271.7 (26599-46231.2) |
| Bermuda | 5.2 (1-10.3) | 7.8 (1.4-15.2) | 14.5 (2.6-28.2) | 30.6 (5.8-58.4) | 66.9 (12.3-126.9) | 125.3 (23.1-240.8) | 168.7 (30.3-328.7) | 215.9 (40.1-418.6) | 280.2 (51.7-536.6) | 440.9 (82.3-847.1) | 579.8 (105.8-1111.8) | 794.8 (149.1-1539.3) | 1220.6 (225.5-2330.4) | 1844.2 (350-3577.3) | 2472.6 (467.3-4868.5) |
| Bhutan | 120.6 (66.9-184.4) | 220.4 (124.9-337.6) | 319.7 (191.1-476) | 574.5 (357.2-887.4) | 945 (607.1-1377) | 1533.9 (1032.6-2173.2) | 2346.9 (1516.6-3305.1) | 3334.3 (2284.5-4723.9) | 4784.1 (3227.2-6468.2) | 6456.7 (4560.4-8529.3) | 8342.4 (5860.7-10677.6) | 9821.9 (7062.2-13077.3) | 11178.6 (8228-14386) | 14268.2 (9991.1-18489.4) | 16952.6 (11336.1-23061.7) |
| Bolivia (Plurinational State of) | 115 (68.7-188.9) | 161.2 (103.3-247.5) | 230.1 (145.4-348) | 375.6 (226.4-579.3) | 612.6 (372.1-998.9) | 1006.5 (603.9-1618.9) | 1479 (919.8-2360.8) | 2162.8 (1299.3-3547.1) | 3145.3 (1950.2-5041.7) | 4500.6 (2834.2-7094.2) | 6131.1 (3913.1-9664.3) | 8239.1 (5329.8-12629.8) | 10188.1 (6606.6-14608.6) | 12031.2 (7863.9-17263.1) | 10472.7 (6892.5-15457.2) |
| Bosnia and Herzegovina | 88.7 (60.4-131.1) | 114.6 (75.9-171.5) | 212.1 (137-306.4) | 346.3 (217.3-495.7) | 673.3 (440.7-963.3) | 1229 (847.6-1710.1) | 1960.5 (1348.4-2790.2) | 3126.6 (2150.9-4375.9) | 4588.2 (3260.1-6359.1) | 7339.9 (5103.8-10096.8) | 12369.6 (8726.8-16981.2) | 16690.9 (11662-22657) | 20902.2 (14304.7-28268.7) | 23628.4 (16126.4-32417.5) | 21637.8 (13221.5-30621.7) |
| Botswana | 42.2 (17.4-81.2) | 75.4 (34.2-135.9) | 148.7 (69.7-259.1) | 297.7 (149.2-514.1) | 534 (278-922.9) | 914.6 (473.5-1591.6) | 1374 (726.8-2319.9) | 2199.8 (1189.6-3820.9) | 3265.9 (1834.2-5335.3) | 4660.9 (2674.3-7871.8) | 6237.8 (3471.1-10505.3) | 7890 (4529-13104.1) | 9253.8 (5292.2-15781.8) | 10198.9 (5566.4-17616.9) | 7216.6 (3534.7-13039.3) |
| Brazil | 42.8 (25.4-61.8) | 72.4 (43.1-105.4) | 126.4 (75.4-183.5) | 220.4 (132-316.8) | 363.3 (218.4-531.3) | 549.9 (327.2-797.5) | 754 (440.3-1089.1) | 1009.1 (587.3-1451.3) | 1280.7 (737.3-1854.3) | 1582.1 (919.8-2280.9) | 1951.3 (1147.4-2835.1) | 2268.7 (1324.4-3316.1) | 2737.3 (1520.6-4020.4) | 3290.7 (1843.4-4881.8) | 3867.8 (2152.8-5809.4) |
| Brunei Darussalam | 22.5 (5.1-46.4) | 41.8 (8.3-85.4) | 85 (17.8-170.9) | 112.1 (23.3-225.4) | 201.7 (42.6-403.8) | 276.5 (55.2-563.5) | 307.9 (64.8-621.2) | 412.4 (87-837.7) | 488 (101.4-967.5) | 661.2 (138.4-1320.9) | 1179 (245.2-2337.2) | 1497 (305.9-2995.4) | 1886.8 (415.9-3729.5) | 2466 (519.4-4808.1) | 6379.1 (1384.3-12692.9) |
| Bulgaria | 114.4 (83.6-165.3) | 185.5 (134.3-271) | 331.1 (242.4-481.3) | 552.6 (398.5-799) | 1031 (740.4-1506.5) | 1592.8 (1160.5-2314.9) | 2393.8 (1738.7-3462.8) | 3295.1 (2387.1-4797.4) | 4305.3 (3143.8-6263.9) | 5873.9 (4330.7-8589.8) | 7894.3 (5908.2-11431.6) | 12136.6 (9115.9-17706.5) | 17861.2 (13379.8-25826) | 28538.2 (21315.3-41215.1) | 46575.7 (34969.4-68324.8) |
| Burkina Faso | 121.4 (83.5-174.1) | 247.2 (169.7-345.9) | 422.4 (299-585.4) | 865.6 (606.7-1214.8) | 1546.4 (1056.2-2131.8) | 2792.2 (1961.7-3796) | 4028.9 (2887.1-5423.8) | 6335.9 (4544.9-8447.1) | 9504.1 (7067.2-12468) | 12864.9 (9690.9-16583.7) | 16151.5 (12598.7-20286.7) | 18421.7 (14194.1-22645.4) | 20555.4 (16100.9-25473.2) | 25329.7 (19445-32162.7) | 31124 (22677.6-39800.1) |
| Burundi | 257.2 (175-354.7) | 437.6 (301.6-597.2) | 648 (448.5-866) | 1263 (898.9-1704.8) | 2041.1 (1447.6-2761.6) | 3231.2 (2302.7-4267.1) | 5216.9 (3677.5-6914.8) | 8241.4 (6145.8-10718.7) | 11441.6 (8639.9-14489.3) | 14584.7 (11048.8-18794.4) | 17814.8 (13374.9-22603) | 19739.4 (14997.8-25731.3) | 21020.6 (15687.4-27715) | 24404.3 (17985.7-32794) | 27097.9 (19163.6-35884.1) |
| Cabo Verde | 99.6 (64.9-150.4) | 175.7 (111-262.7) | 294.8 (184.2-432.7) | 599.4 (381.3-880.9) | 1098 (735.3-1542.6) | 1939.5 (1316.6-2768.6) | 2764.7 (1871.8-3823.3) | 4217.7 (2876.3-5725.3) | 6085.1 (4273.5-8151.6) | 8813.6 (6253.9-11728.7) | 12073.6 (8745.9-15918.4) | 16104.9 (11929.1-21193.4) | 20349.2 (14810.5-26455.7) | 24780.9 (18176.2-32056.8) | 28878.7 (20357-37128.8) |
| Cambodia | 235.3 (159.2-352.6) | 399.5 (272.1-587.6) | 676.9 (468.9-959.7) | 1218.7 (847-1705.3) | 2253.5 (1549.7-3082.2) | 3772.5 (2672.3-5013.8) | 5533.8 (3989.5-7479.7) | 7742.6 (5561.4-10294.2) | 10663.5 (7922.3-13730.6) | 15345.5 (11787.7-19266.8) | 20600.8 (15760.6-25649.4) | 25768.7 (19869.5-31596.2) | 30825.7 (23958.8-37734.4) | 36596.5 (28334.5-44755.8) | 43157.7 (32452.3-54502) |
| Cameroon | 192.5 (124.6-279) | 362.6 (239.8-522.1) | 586.3 (393.3-811.6) | 1138.6 (753.5-1692.8) | 1930.4 (1223.8-2841.5) | 3349.4 (2216.8-4804.1) | 4765 (3185.2-6856.4) | 7518.2 (5109.5-11044.5) | 10795.9 (7362.9-14649.6) | 14223.9 (10253.1-20131.7) | 17862 (13699.5-23941) | 21061.6 (16208.9-27535.6) | 23824.5 (18532.4-31835.6) | 28805.3 (22253.6-37998.5) | 34260.9 (24442.8-43949.2) |
| Canada | 3.9 (1.5-7.2) | 6.9 (2.5-12.4) | 11.9 (4.4-21.5) | 20.4 (7.5-37) | 34 (12.4-61.8) | 55.1 (20.4-97.8) | 80.2 (29.4-145) | 112.7 (40.1-200.8) | 148.7 (52.7-263.7) | 200.8 (72.2-362.5) | 284.3 (103.4-502.5) | 423 (150-753.8) | 720.5 (256.5-1307.4) | 1139.8 (403.6-2091.3) | 2172.2 (768.2-3901.7) |
| Central African Republic | 219.3 (138.6-327.9) | 448.8 (274.1-682.6) | 821.5 (500.6-1257.5) | 1892.4 (1125.1-3076.9) | 3628.2 (2233.7-5623.2) | 5808.9 (3691.5-8915.4) | 8851.9 (5956.7-12853.1) | 12639 (8872.7-17520.9) | 17296.4 (12587-23676.9) | 22319.1 (17179.8-29032.6) | 26586.6 (19852.2-34319.3) | 29741.8 (21959.1-38374.6) | 30124.5 (21368.9-40906.7) | 29831.3 (21166.5-40380.1) | 22309.3 (14331.1-32025.9) |
| Chad | 213.1 (146.4-301.2) | 388.1 (273-542.3) | 618.4 (431.5-894.7) | 1239.3 (864.3-1698.7) | 2238.5 (1585.2-3164.2) | 4140 (2933.8-5616.3) | 5962.4 (4159-8126.4) | 9335.7 (6791.9-12636.2) | 13523.6 (9848.3-17862.8) | 17234 (13199.9-22355.4) | 20421.4 (15220.6-25769.3) | 23057.7 (18194.3-28957.6) | 24556.5 (18801.9-30838.3) | 28244.1 (22200-35937) | 35901.7 (26772.5-46117.1) |
| Chile | 39.7 (26.4-55.2) | 63.8 (40.8-87.2) | 104.1 (70.1-142.3) | 186.8 (122.5-252.9) | 305.4 (211.5-412.4) | 473.7 (316.4-638.4) | 685.2 (465-924.9) | 894.7 (611.8-1215.6) | 1230.9 (816.6-1661) | 1643.2 (1100.2-2211) | 2221.8 (1458.9-3019.4) | 2904.4 (1928.8-3997.2) | 3770.2 (2478.5-5201.9) | 5820.2 (3772.1-8000.7) | 10355.7 (6656.5-14282.3) |
| China | 152.4 (118.2-195) | 259.2 (201.3-330.9) | 423.7 (319.5-538.2) | 708.1 (533.9-928.9) | 923.3 (680.2-1221.6) | 1496.1 (1102.4-1944.9) | 2159.2 (1597-2814) | 3315.6 (2473-4305) | 4995.2 (3757.8-6465.1) | 8118.8 (6145.9-10390.7) | 11613 (8792.3-14848.6) | 17537.1 (13383.6-22055.4) | 26309 (20003.1-32738.7) | 35390.1 (26860.7-44142.8) | 41032.2 (29431.2-52066.9) |
| Colombia | 35.3 (22.2-51.5) | 54.9 (35.1-80) | 86.5 (55.2-127.6) | 144.9 (89.4-214.6) | 238.8 (147.7-351.7) | 384.5 (241.8-567) | 576.2 (356.4-840.5) | 901.3 (557.7-1318.8) | 1258.2 (785.4-1839.2) | 1764.3 (1120.3-2573.2) | 2350.8 (1493.7-3375.1) | 3289 (2094.1-4720.3) | 4102.6 (2599-5856) | 5122.3 (3192.5-7443.6) | 4881.3 (2812.9-7230.1) |
| Comoros | 180.3 (123.7-253.4) | 308.3 (213-426.9) | 465.3 (327.3-631.6) | 852.3 (587.3-1180.1) | 1337.1 (931.9-1833.3) | 2111.9 (1458.9-2859.9) | 3393.6 (2352.4-4584.6) | 5417.3 (3790.7-7146.5) | 7867 (5768.1-10383) | 10868.9 (8058.1-14269.3) | 13613.7 (10281.6-17827.8) | 15548.4 (11947.5-19650.8) | 17100.9 (12231.1-22323.4) | 20586.9 (14545.9-27362.2) | 22880.7 (15664.4-30369.6) |
| Congo | 153.1 (94-235.1) | 299.7 (182-475.9) | 505.9 (321.7-807.1) | 1059 (654.7-1649.2) | 1976.2 (1225.2-3039.4) | 3164.7 (2022.1-4589.4) | 4964 (3361.6-6984.8) | 7576 (5053.1-10235.8) | 10870.8 (7840-13917.2) | 14837 (10987.9-18981) | 18529.2 (13334.6-23523.1) | 21768.9 (16383.1-27688.7) | 24071.7 (17602.5-31201.7) | 27560 (19631.4-35739.5) | 28872 (19629.2-38900.2) |
| Cook Islands | 21.4 (0-53) | 41.9 (0-105.1) | 67 (0-170.1) | 123.4 (0-316.9) | 167.4 (0-419) | 234.1 (0-581.7) | 304.1 (0-744.4) | 416.5 (0-1055.2) | 486.4 (0-1243.2) | 594.1 (0-1427.4) | 803.9 (0-1979.9) | 978.6 (0-2395.7) | 1403.3 (0-3511.7) | 1884.6 (0-4622.8) | 2629.2 (0-6397.6) |
| Costa Rica | 31.5 (20.5-45) | 53.6 (34.7-78.4) | 82.8 (53.2-121.6) | 146.6 (94-210) | 225.9 (142.8-322.8) | 312.5 (198.5-446.2) | 454.4 (290.9-639.7) | 604.4 (387.4-862.6) | 816.7 (527.5-1161.4) | 1066.4 (698.6-1516.2) | 1439.5 (941.5-2036.9) | 1980.6 (1292.6-2757) | 2526.7 (1614.5-3549.1) | 3626.1 (2299.1-5134.2) | 4585.7 (2756.5-6623.6) |
| Côte d'Ivoire | 214.6 (144.4-305.4) | 390.3 (268.4-554.2) | 630.8 (437.2-889.2) | 1229.6 (833.3-1755.2) | 2070.8 (1416.6-2922.3) | 3594.3 (2514.9-4961.1) | 5057.9 (3501.8-7036.2) | 7944.5 (5438.2-10989.8) | 11484.2 (8469.5-15504.7) | 14940.4 (10904.6-19894.7) | 18549.5 (14152.2-23707.7) | 22011 (17600.9-27942.6) | 25448 (20087.8-32196.4) | 30848.6 (23938.9-37964.3) | 39043.2 (28963.1-48695.7) |
| Croatia | 17.9 (12.8-24) | 33.5 (24.5-44.7) | 69.1 (50.9-91.8) | 135.5 (97-182.3) | 271.4 (195.5-360.6) | 457.6 (327.8-618.3) | 720 (517.8-959.9) | 1090.2 (783.4-1452.1) | 1608.7 (1166.2-2113.1) | 2415.1 (1750.5-3105.1) | 3766.2 (2750.5-4824.9) | 6033 (4446.7-7702.4) | 9813.3 (7147.4-12571.3) | 14501.1 (10423.3-18730.8) | 23907.6 (16762.1-31349.2) |
| Cuba | 43.2 (20.9-72) | 67.8 (34.2-109.8) | 131.3 (65.6-217.4) | 253.8 (129.1-420.7) | 442.4 (210.7-731.2) | 731.3 (369.4-1221.1) | 1141.1 (559.5-1861.7) | 1790.7 (898.9-2869) | 2587.6 (1290.4-4235) | 3242.4 (1603.5-5326.7) | 4330.1 (2124.4-7138) | 5641.6 (2732.6-9142.5) | 7068.9 (3397.3-11319.2) | 9861.5 (4752.6-15879.8) | 13450 (6427.4-22080.5) |
| Cyprus | 13.6 (9.1-19) | 21.2 (14.4-30.1) | 46.7 (30.6-67.3) | 92.6 (63.1-122.7) | 194.3 (125-270.9) | 286.3 (190.1-389.4) | 431 (291.9-595.3) | 633.1 (439.6-867.6) | 815.3 (553.1-1115.2) | 1324.2 (944.1-1814.5) | 1670.2 (1167.1-2343.6) | 2972.1 (2102.3-4003.6) | 5122.1 (3632.5-6852.8) | 13683.2 (9559.8-18056.7) | 26389.8 (18386.7-35516.4) |
| Czechia | 19 (14.3-24.3) | 34.5 (25.2-44.5) | 60.3 (43.6-79.5) | 103 (74.4-136.6) | 194.9 (139.9-260.6) | 360.4 (262-476.6) | 569.7 (413-756.4) | 981.9 (721.2-1284.4) | 1357.8 (990.9-1775.9) | 2042.8 (1493.4-2631) | 2901.9 (2128.2-3737.1) | 4500.7 (3250-5786.3) | 7973.9 (5715.7-10315.3) | 10455.3 (7394.5-13593.1) | 13377.8 (9141.7-17862.2) |
| Democratic People's Republic of Korea | 511.8 (343.3-786.1) | 779.4 (513.8-1203.7) | 1258 (848-1850.8) | 2167 (1458.5-3148.7) | 3501.7 (2378.6-4756) | 5321.8 (3802-7066.6) | 7410.3 (5455.4-9420) | 10048.4 (7654.6-12766.5) | 13176.9 (10224.6-16272.4) | 16731.5 (13104.9-20312.5) | 20710.4 (16012.1-25472.6) | 25489.9 (19037.4-32002.8) | 31056.6 (22015.7-39687.7) | 39528.4 (26078.9-52512) | 52767.6 (32626.2-68683.7) |
| Democratic Republic of the Congo | 135.7 (91.2-194.7) | 268.6 (174.7-397.2) | 457 (311.4-652.7) | 986 (656.8-1411.7) | 1866.3 (1242.9-2693.3) | 3190.5 (2128.2-4464.2) | 5174 (3606.2-7196.9) | 8160.9 (5791.6-10938.7) | 12082.9 (8833.5-15690.8) | 16435.4 (12139.8-21717.1) | 19826.9 (14697.1-25926.5) | 23090.9 (16755.1-31063.1) | 23672.6 (16853.1-32241.3) | 26029.5 (18151.5-36106.3) | 26007.8 (17592.3-36391) |
| Denmark | 5.7 (3.4-8.3) | 9.7 (5.6-14.2) | 18.9 (11.5-27.2) | 34.2 (20.8-49.7) | 63.9 (37.8-92.8) | 91.8 (55.3-131.1) | 151.9 (90.8-218.1) | 231.6 (139-334.3) | 319.6 (193.2-462.6) | 451.6 (273.9-649.5) | 663.8 (399.9-957.1) | 979.1 (599.9-1403.4) | 1692 (1021.6-2447.8) | 2759.8 (1642.7-4076.5) | 3585.6 (2036.4-5333.3) |
| Djibouti | 137.1 (74.3-220.9) | 245.5 (143.9-388.5) | 377 (210.2-582.8) | 758.5 (457.3-1162.4) | 1240.1 (679.7-1998.8) | 1992.2 (1189.7-3089.5) | 3227.6 (2049.3-4892.3) | 5123.1 (3119.7-7873.7) | 7179.7 (4485-10825.8) | 9638.7 (6152.6-13984.1) | 11704.6 (7596-16901.3) | 13609.5 (9071.8-19460.6) | 14901.9 (9422.7-21081.2) | 17422.3 (10909.2-25327.8) | 18495.8 (10827.1-27917.5) |
| Dominica | 38.5 (16.8-67) | 69.4 (30.1-124.8) | 127.6 (57.1-222.7) | 252.8 (108.1-446.3) | 461.6 (208.3-800.7) | 812.2 (362-1458.9) | 1234.2 (563.6-2146.6) | 1864 (806.7-3269.2) | 2644.2 (1174.6-4450.1) | 3867.9 (1764-6703.7) | 5233.1 (2388.4-8792.1) | 6971.7 (3195.7-11599.7) | 8933.4 (3887-15355.8) | 11064 (4920.1-18757.5) | 13927.7 (5888.1-23193.8) |
| Dominican Republic | 140.7 (48.8-269.5) | 214.7 (77.3-386.1) | 335.6 (121.6-606.4) | 595.8 (208.6-1058.1) | 979.3 (356.9-1692.9) | 1417.8 (497.2-2553.2) | 2022.9 (753.2-3612.4) | 2751.4 (1012.2-4883.2) | 3548.3 (1368.3-6184) | 4301.5 (1649.9-7579.6) | 5239.2 (1901.8-9154.5) | 6392 (2413.2-11167.4) | 8130.6 (2979.5-14487.6) | 9262.6 (3410.5-16120.8) | 16435.6 (6063.3-28835.5) |
| Ecuador | 79.9 (47.8-121) | 115.6 (68.8-171.7) | 149.4 (89.2-227) | 234.5 (136-362.3) | 344 (196.2-541.7) | 510.5 (297.2-802.5) | 740.8 (430.1-1159) | 1077.7 (628.5-1665.8) | 1349.7 (794.6-2037.8) | 1813.9 (1088.8-2697.1) | 2396 (1428.1-3568.3) | 3344 (2011.7-4935.9) | 5434 (3265-7910.9) | 9306.5 (5765.5-13330) | 19755.5 (12304.3-28349.3) |
| Egypt | 622.4 (465.7-787.3) | 903.5 (668.1-1170.6) | 1172.3 (879.7-1493.9) | 1834.2 (1353.3-2382.3) | 2898.2 (2137.1-3815.2) | 5091.3 (3804.9-6616.4) | 7814.6 (5734.2-10121) | 11262.5 (8546.4-14467.6) | 15939.1 (12145.3-20397.4) | 24186.7 (18380.7-30510.2) | 32608.6 (25214.8-41437.7) | 47659.4 (36896.6-58104) | 59532.7 (46527.2-72684.7) | 41755.4 (31793.5-52052.5) | 34627.8 (23850.4-45544.9) |
| El Salvador | 89.1 (50.9-132.9) | 144.2 (83.6-211.5) | 231.4 (132.2-348.9) | 385.3 (234.4-568.6) | 556.2 (327.5-846.2) | 832.6 (510.2-1236.4) | 1169.2 (689.3-1738.3) | 1586.1 (947.8-2312.5) | 2057.8 (1255-2987.8) | 2835.3 (1757.3-4064.2) | 3651.5 (2255.3-5227.8) | 4986.8 (3128.6-7004) | 6507.1 (3839-9294.5) | 9314.2 (5526.7-13075.3) | 9431 (5399.1-13641.7) |
| Equatorial Guinea | 75.5 (33.5-137.9) | 151.5 (71.4-271.7) | 253.6 (115.9-459.9) | 523.7 (248.9-936.4) | 948.1 (442.2-1645.8) | 1539.4 (746.4-2720.2) | 2459.2 (1210.7-4132.5) | 3896.2 (1862.8-6713.9) | 5999.4 (3010.2-9968.1) | 8353.6 (4501.9-13425.9) | 10914.6 (6074.5-17239.8) | 13503.3 (7505.4-21280.8) | 14805.3 (8038.7-23069.4) | 17211.1 (9249.3-27489.9) | 17569.4 (9067.5-28331.9) |
| Eritrea | 250.2 (152.6-376) | 474.4 (287-718.2) | 741.3 (462.4-1107.1) | 1477 (941.9-2224) | 2359.2 (1583.9-3456.1) | 3471.8 (2320.7-5056.1) | 5226.7 (3556.3-7360.8) | 7855.2 (5648.6-10494.2) | 10534.7 (7748.5-13718.2) | 14017.1 (10544.9-18361.4) | 17560.6 (13039.5-22594.5) | 19497.6 (14695.5-25156.5) | 21336 (15549.5-28473.6) | 24693.1 (17521.7-32728.7) | 24350.9 (16207.8-34001) |
| Estonia | 4.8 (1.5-10.1) | 7 (2.1-15.1) | 16.1 (4.8-33.3) | 29.3 (9-59.8) | 56 (17.1-113.5) | 111.7 (34.1-224.7) | 168 (52.5-328.6) | 262.9 (80.2-512.9) | 368.6 (113.2-724.5) | 517.8 (156.6-1021.9) | 668.3 (214.1-1335.4) | 984.4 (295.1-1921.7) | 1670.9 (513.8-3363.5) | 2626.9 (802.8-5252.5) | 4703.5 (1441.6-9481.4) |
| Eswatini | 113.7 (53.8-190.5) | 207.1 (94.3-348.3) | 434.5 (191-773.7) | 947 (437.4-1670.8) | 1782.8 (834.3-3204.1) | 2751.5 (1373.1-4845.4) | 3846.9 (1915.6-6586.1) | 5651.8 (2830.9-9675.6) | 7645 (4257.2-12234.4) | 10025.9 (5425.1-16203.4) | 13390.7 (7353.1-20786.2) | 16793.3 (9419.8-24677.1) | 19562.8 (10779.7-28429.4) | 12630.6 (6383.8-20182.9) | 7080 (3597.8-12018.3) |
| Ethiopia | 154.6 (114.7-198.4) | 268 (204.6-338.7) | 370.3 (282.7-471.6) | 673.4 (513.4-842.2) | 1051.1 (814.3-1312.4) | 1708.1 (1328.9-2143) | 2852.8 (2204.3-3569.2) | 4606 (3608.2-5705.5) | 6549.2 (5219.5-8042.9) | 8795.1 (7123-10661.9) | 10502.3 (8512-12704.6) | 12044.8 (9581.3-14612.4) | 13296.7 (9910.8-16685.7) | 15568.1 (11132.9-19855.6) | 15063.9 (10057.1-19921.7) |
| Fiji | 244.2 (75.3-449.5) | 406.5 (127.2-752.6) | 714.6 (220.8-1332.2) | 1251.1 (393.5-2214.6) | 2079.4 (661.5-3839.4) | 3085.5 (992-5760.1) | 3903.7 (1182.8-7083.2) | 5382.2 (1735.3-9619.7) | 6169 (1999.3-10887.9) | 7859.3 (2577.3-13941.2) | 9426.2 (3072-16331.5) | 11541 (3746.5-19923.6) | 17319.8 (5813.6-29729.7) | 19295.3 (6710.6-33291.2) | 25313.3 (9018.8-43332) |
| Finland | 2.5 (0.3-5.5) | 4.4 (0.6-9.4) | 7.1 (0.9-15.3) | 12.4 (1.7-27.2) | 21.5 (2.9-46.3) | 40.4 (5.4-86.5) | 63.2 (8.3-134.6) | 97.3 (13-206.8) | 139.2 (18.6-296.7) | 199.7 (27-426.7) | 271.8 (36.6-577.2) | 439 (57.3-944) | 769.3 (99.2-1703.9) | 1111.4 (143.6-2456.9) | 1636.4 (212.5-3626.9) |
| France | 5.8 (3.8-8.3) | 11 (7.4-15.3) | 19.9 (13-28.5) | 35.4 (23.1-49.9) | 59.6 (39.3-83.4) | 89.4 (58.9-123) | 126.9 (83.3-177.2) | 178.8 (116.3-250.5) | 246.3 (162.3-346.6) | 343.4 (228-471.6) | 485.3 (323.3-664.1) | 717.7 (474.2-1003.4) | 1239.1 (803.2-1738.8) | 2421.6 (1543.5-3395.3) | 3970.9 (2499.9-5724.1) |
| Gabon | 58.1 (30.3-96.4) | 123.6 (63.1-218.1) | 219 (117.3-379.6) | 476.5 (253.2-827) | 888 (460.3-1480.7) | 1451.1 (808.7-2334) | 2283.8 (1318.6-3707.2) | 3611.1 (2024.2-5622) | 5401 (3139.1-8277.7) | 7404.4 (4355.8-11299.8) | 9489.6 (5640.1-14262) | 11864.3 (6989.5-17756.3) | 13221.3 (7922-19333.1) | 15289.9 (8810.3-22304) | 15302.4 (8705-23276.9) |
| Gambia | 250.7 (170.2-366.6) | 445.5 (299.7-641.4) | 718.4 (496.1-989.7) | 1416.5 (982.2-1948.9) | 2494 (1658.8-3442.4) | 4441.7 (3154.4-5917) | 6418.6 (4537.9-8471.5) | 10031.1 (7222.9-12961.1) | 14280.9 (10501.9-18076) | 19066.6 (14451.7-23945.8) | 23639.3 (18029.5-29204) | 27574.9 (21400.4-33709.6) | 32292.1 (23898.9-40694.1) | 38201.9 (29453.5-47784.9) | 45880.5 (34668.6-57455.8) |
| Georgia | 76.4 (47.8-112.4) | 168.1 (103-241.6) | 322.7 (197.2-463) | 601.8 (374.6-864.5) | 986.2 (613.8-1445.9) | 1443.1 (895.5-2096.6) | 2111.5 (1328.3-3078.9) | 2839.2 (1779.7-4104.3) | 4015 (2499.8-5831.6) | 6459.8 (4014-9273.3) | 9470.9 (5854.8-13604.2) | 13013.7 (7926.8-18500.5) | 12878.3 (7643.9-18462.7) | 12621.6 (7065.1-18483.2) | 11107.4 (5569.8-16787.5) |
| Germany | 7.2 (4.7-10.1) | 14 (9.4-19.1) | 27.5 (19-38.2) | 50.5 (33.9-70.7) | 96.4 (65.2-130.6) | 160.4 (107.8-219.3) | 244.7 (163.8-335.4) | 374 (251.6-509.6) | 534.7 (368-725.6) | 714.1 (492.9-978.2) | 1109.5 (766.4-1496.5) | 1546.4 (1048.4-2119.9) | 2414.1 (1542-3343.5) | 4223.9 (2719.4-5836.8) | 6131.3 (3850.3-8638.9) |
| Ghana | 234.4 (163.4-325) | 450.9 (307.3-604.7) | 711.3 (498.1-974.5) | 1471.7 (1021.9-1965) | 2468.9 (1702.3-3338.5) | 4116.3 (2849.2-5397.4) | 5535.4 (3917-7387) | 8529.2 (6082.7-10991.8) | 11984.2 (8911.3-15144) | 15452.1 (11330.9-19581.4) | 19517.9 (14793-24335) | 23547.8 (17796.8-29464.8) | 28403.4 (21342.6-36024.3) | 34226.7 (25427.5-43111.4) | 43595.1 (32485.8-54560.8) |
| Greece | 29 (21.9-36.8) | 52.1 (38.2-67.3) | 95.9 (70.4-125.3) | 175.4 (128.9-226.8) | 307.6 (225.4-390.1) | 486.1 (354.9-626.5) | 664 (487.2-850.1) | 827.2 (611.5-1056.9) | 1066.1 (795.9-1366.9) | 1380.5 (1041.8-1740.6) | 1900.7 (1438.9-2386.1) | 2915.6 (2161.6-3660.7) | 4698.5 (3478.3-6087.8) | 7879.9 (5720.9-10083.4) | 11919.6 (8700.4-15339) |
| Greenland | 6.6 (0.2-18) | 12.3 (0.4-31.1) | 24.9 (0.9-65.7) | 52.1 (1.8-140.5) | 95.2 (3.5-263.8) | 160.5 (6-431.8) | 220.5 (8.7-607.4) | 315.7 (11.7-854.1) | 390.9 (15.4-1051) | 521.8 (19.1-1386.8) | 670.9 (24.6-1794.7) | 872.9 (33.5-2304.8) | 1232.1 (45.4-3147.4) | 1729.3 (64.4-4638.9) | 2768.2 (98.3-7172.2) |
| Grenada | 54.3 (21.9-101) | 100.8 (40-179.8) | 203 (81.8-362.5) | 300.4 (119.3-539.3) | 575.2 (233-999) | 1024.7 (415.2-1788.8) | 1619.9 (629.6-2853.8) | 2336.6 (946.5-4221.3) | 3084.3 (1237.7-5418.5) | 4188.5 (1638.4-7354.3) | 6831.3 (2737.5-12113.3) | 6842.3 (2795.3-11987.7) | 8064.4 (3230.4-14228.4) | 11316.8 (4648-19829.8) | 14197.1 (5803.2-25293.6) |
| Guam | 61.6 (29.8-98.9) | 148.1 (72.9-233) | 230 (105.9-355.3) | 435.3 (200-688.6) | 696 (322.1-1086.4) | 784.5 (378.6-1209.1) | 1056.8 (512.3-1667.7) | 1229.5 (571.9-1882.4) | 1383.1 (660.7-2206.4) | 1587.9 (758.8-2455.2) | 1596.1 (766.5-2471.5) | 1565.8 (753.3-2418.2) | 1373 (658-2140.9) | 1355.5 (637-2205.9) | 2130.4 (967.2-3479.4) |
| Guatemala | 168.5 (111.2-224.4) | 254.6 (170-340.1) | 369.5 (239.7-497.9) | 534.4 (356.4-733.5) | 755.2 (496.6-1017.2) | 1073.9 (705.1-1431.9) | 1454.5 (961.9-1979.8) | 2106.4 (1370.7-2829.4) | 2838.1 (1909.7-3792.9) | 4018.1 (2648.2-5365) | 5383.2 (3582.4-7049) | 8115.6 (5320.6-10462.6) | 11859.5 (7780.7-15392) | 23656.5 (15534.9-30203.3) | 49119.5 (33165.3-62883.9) |
| Guinea | 244.6 (172.3-337.8) | 442.4 (305.8-624.8) | 660.1 (455.5-910.7) | 1258.7 (871.7-1754.5) | 2169 (1488-3064.3) | 3958.1 (2797.7-5403.7) | 5735.5 (3954.4-7918.1) | 9051.5 (6237.8-12378.6) | 12966.9 (9571.4-17805) | 17030.5 (12728.5-22736.4) | 20655.5 (16079-26422.5) | 23712.9 (18657.1-29977.3) | 26482.6 (20951.6-32872.5) | 31468.6 (25124.6-39123.1) | 37699.6 (28547-46961.3) |
| Guinea-Bissau | 371.5 (251.6-524.7) | 701.2 (486.4-1000.5) | 1104.2 (755.2-1634.6) | 2246.3 (1514.7-3269) | 3931.1 (2679-5493.6) | 6487.2 (4675.8-8807.9) | 9078.5 (6527.6-11927.1) | 13500 (9960.9-17917.5) | 18337 (14329.9-23370.9) | 23321.8 (17880.8-28826.7) | 28095.5 (21379.1-33906) | 31652.7 (24890.5-38329.4) | 36367.4 (27547.1-45392.6) | 40735.2 (30942.1-51173) | 40148.2 (28137.4-53622.6) |
| Guyana | 143.4 (67.6-243.9) | 238.4 (112.4-403.6) | 388.8 (177.4-665.8) | 711.9 (334.2-1228.3) | 1294.9 (599.2-2228) | 2280.4 (1047.7-3845.1) | 3400.5 (1581.3-5824.6) | 4730.7 (2258.3-7955.8) | 6231.5 (2922.9-10189.9) | 7995.8 (3784.3-13068.9) | 8907.3 (4230.5-14458.6) | 10437 (4860.8-16700.7) | 11152.2 (5281.9-17997) | 17081.2 (8063.6-27484) | 21740.3 (9724.3-38336.2) |
| Haiti | 454.5 (297.9-643.7) | 711.6 (471.6-1036.4) | 1188.4 (831.5-1684.5) | 2172.2 (1491.8-3151.9) | 3635.8 (2506-5337.1) | 5927 (4135.6-8368.3) | 8772 (6173.6-12368.9) | 12729.3 (8822.1-17170.7) | 17365.6 (12567.2-22901.3) | 22893.1 (17122.7-29785.8) | 27599.2 (20559.1-36140.2) | 32312 (24671.1-41269.7) | 36187.7 (27357.6-46423.5) | 42516.8 (31700.2-54512.2) | 50112.3 (30297.7-68136.7) |
| Honduras | 133.1 (66.4-214.2) | 214.7 (111.2-353.5) | 367.4 (202-567.2) | 718.6 (433.7-1077.7) | 1334.7 (906.9-1917.5) | 2326.2 (1605.3-3220.9) | 3732.6 (2661.2-5071.1) | 5527.9 (3915-7326.2) | 7947.6 (5757.8-10439.2) | 11068.3 (8233.4-14193.2) | 14691.8 (10696.8-19343.8) | 18731.1 (13820.8-24309.1) | 23072.8 (17029-30133.5) | 30912.2 (22608-40226.8) | 42030.2 (30348.2-53342.9) |
| Hungary | 24.8 (17.6-37.3) | 49.9 (35.2-73.6) | 95.6 (67.6-144.5) | 185.6 (129.9-279.2) | 350.7 (249.6-526.2) | 681.2 (483.4-1010.5) | 1097.2 (768.2-1628.4) | 1664.6 (1174.7-2470.1) | 2139.9 (1533.8-3183.1) | 2866.1 (2077.8-4315.7) | 4065.2 (2881.7-6044.5) | 6149 (4297.5-9272.4) | 9178.3 (6422.6-13622.5) | 10123 (6880.8-14726.8) | 13952.2 (9372.6-20298.8) |
| Iceland | 3.3 (0.6-7.2) | 5.2 (1-11.4) | 6.8 (1.3-15.1) | 15.7 (2.8-34.7) | 23.4 (4.2-51) | 25.5 (4.7-57) | 54.9 (10.2-120.6) | 62.2 (11.2-137.4) | 96.2 (17.4-207.5) | 132.3 (24.2-291) | 235 (42.8-508.6) | 322.4 (59.1-731.1) | 596.8 (109.4-1311.1) | 1028.7 (190.3-2325.1) | 980.1 (178.8-2232.6) |
| India | 242.5 (195.4-289.4) | 493.2 (404-589.1) | 737.1 (605.2-878.9) | 1427.3 (1170.7-1718.1) | 2170.1 (1760.6-2595.7) | 3339.5 (2735.3-4021.1) | 5575 (4570.8-6694.3) | 6849.7 (5686.1-8216.8) | 9223.2 (7624.2-10930.1) | 11251 (9318.8-13391.3) | 12729.8 (10465.5-14960.4) | 13407.5 (10867.3-15796.6) | 14464.2 (11715.7-17164.1) | 19364.9 (15486.1-23038.7) | 25150.8 (19742.7-30103) |
| Indonesia | 233.6 (162.2-339.3) | 390.7 (269.1-564.4) | 642 (449.3-918.2) | 1090.9 (770.1-1563) | 1727.4 (1180.2-2462.8) | 2672.1 (1837.6-3778.3) | 3791.7 (2578.7-5318.7) | 5243.7 (3481.9-7299.1) | 7170.6 (4850.1-9938.9) | 9588.1 (6561.9-12860.9) | 12384.9 (8535.6-16618.4) | 15408.6 (10735.4-20438.3) | 18663.7 (12835.3-25014.1) | 22935.5 (15544.2-30653.1) | 28299.9 (18768.7-39111) |
| Iran (Islamic Republic of) | 146.4 (118.1-176.2) | 208 (166.7-252) | 301.5 (238.9-363) | 493.8 (388.6-597.1) | 817.3 (638.7-998.9) | 1329.2 (1042-1618.5) | 2011.5 (1579.3-2448.3) | 2964.1 (2330.1-3560.4) | 4122.3 (3274.3-4942.5) | 5675.5 (4494.2-6812.9) | 8909.5 (7098.5-10612.9) | 12228.6 (9438.3-14700.9) | 16193.5 (12440.9-19686) | 22036.7 (16351.9-26931.7) | 26214.8 (18297.5-33027.6) |
| Iraq | 158.8 (103.7-241.1) | 248.9 (169.3-374) | 454.1 (307.4-685.9) | 944.9 (637.9-1389.6) | 1911.1 (1275.9-2844.5) | 3336.1 (2229.9-4834.9) | 5179.3 (3408.7-7494.2) | 7513.8 (4905.8-10398.5) | 10615 (7133.2-14338.6) | 14515.3 (10192.9-19282.2) | 19124.4 (13564.6-25012.9) | 24171.9 (17796.7-30893) | 30356.3 (22714.2-38608.7) | 39407.7 (28747.8-50300.6) | 52260.1 (35233.8-69589) |
| Ireland | 4 (2-6.4) | 8 (4.1-12.8) | 14.8 (7.3-23.3) | 28.8 (14.4-45.5) | 52.7 (26.7-83.9) | 77.9 (39.1-123) | 119.6 (60-186.3) | 162.4 (82.2-254.3) | 214.5 (108.4-335.8) | 343.2 (173.8-535.7) | 516.7 (260.5-805.9) | 813.4 (407.4-1264) | 1297.6 (653.4-2052.7) | 1991.1 (985.8-3142.2) | 3094.5 (1549.2-4986.5) |
| Israel | 10.9 (8.3-14.1) | 20.6 (15.6-26) | 32.7 (24.3-41.8) | 59 (45.1-75.1) | 106.7 (81.5-134.5) | 202.8 (157.5-254.4) | 320.4 (245.2-399.2) | 431.8 (331.1-537) | 598.8 (458.3-737.7) | 873.5 (671.1-1086.4) | 1313.2 (991.4-1644.5) | 2110.6 (1586.6-2657.1) | 3546.8 (2571.7-4513.8) | 5363.6 (3838.7-6916.2) | 7808.6 (5451-10018.3) |
| Italy | 13.7 (10.3-17.5) | 21.1 (15.7-27) | 33.6 (25.2-42.9) | 59.7 (44.4-75.7) | 99.8 (74.4-126.7) | 156.9 (117-199.8) | 231.4 (172.8-291.4) | 337.4 (253-424.1) | 487.8 (362.2-613.6) | 727.7 (542.4-922.6) | 1158.7 (858.1-1473.4) | 1802.8 (1319.1-2303.5) | 3179.4 (2295-4145.9) | 5596.4 (3863.8-7378.1) | 9250.6 (6030.7-12288.6) |
| Jamaica | 38.3 (21.5-59.7) | 67.1 (36.5-105.9) | 121.1 (65.8-187.5) | 223.6 (120.4-346.2) | 386.5 (208.7-621.9) | 601.2 (329.2-951.1) | 922.3 (499.3-1466) | 1380.2 (759.3-2136.2) | 2004.1 (1122.3-3090.4) | 3006.6 (1701.2-4607.5) | 3839.9 (2126.3-5872.5) | 4819.2 (2645-7162.6) | 5710.1 (3176-8487.5) | 7174.7 (3999.2-10520.6) | 7773.7 (4278.3-11717.6) |
| Japan | 14.5 (7.9-21.9) | 26.7 (14.6-39.9) | 46 (25.3-69.4) | 82.7 (46.1-124) | 137.6 (77.4-206.4) | 200.3 (111.5-301.9) | 264.8 (148-399.2) | 333.7 (185.9-502.3) | 425.4 (236.1-641.1) | 552.8 (302.5-832.2) | 771.5 (422.7-1164.9) | 1056 (559.8-1599.4) | 1600.9 (844.2-2432.5) | 2710.8 (1397.6-4211.4) | 3725 (1842.2-5891.8) |
| Jordan | 103 (73.3-135.1) | 163.3 (117.6-215.8) | 255.6 (182.8-341.1) | 433.3 (304.3-572.4) | 733.2 (506.6-1010.9) | 1102.7 (772.2-1490.4) | 1560.8 (1092.6-2103.6) | 2379.8 (1676-3190.8) | 3288.1 (2314.2-4414) | 5062.5 (3691.5-6668.8) | 6198.2 (4558.4-8009.4) | 8326.6 (6008.4-10782.6) | 13701.4 (10025.3-17297.9) | 17304.7 (12254.2-22306) | 17709.7 (11996.2-23459.4) |
| Kazakhstan | 47.2 (30.5-68.7) | 110.2 (70.7-157.9) | 218.8 (141-315.4) | 432.1 (290.5-592.1) | 773.6 (521.6-1052.3) | 1298.7 (883.6-1768.5) | 2094 (1440.2-2869.5) | 3480.1 (2403.7-4711.1) | 5091.6 (3564.3-6835.3) | 7938.9 (5548.7-10637.6) | 10862.5 (7548.8-14568) | 16400.1 (11380-22020.5) | 21239.3 (14615.6-28774.7) | 28771.6 (19690.6-39125.9) | 43509.4 (29328.9-59702.7) |
| Kenya | 111.3 (83.2-144.8) | 202.2 (151.8-264.7) | 311.9 (234.2-407.7) | 620.3 (463.1-810) | 1040.6 (778.4-1343.7) | 1669.3 (1277.6-2155.4) | 2607.9 (2011.7-3343.9) | 4464.2 (3451.1-5763.5) | 6469.2 (5040.9-8279.6) | 9292.6 (7206.6-11833.1) | 12659 (9910.6-16076.6) | 15632.6 (11907.1-20048.9) | 17859.5 (12964.9-23443.4) | 20739.2 (14584.4-27459.6) | 20777.2 (13939.7-27662.9) |
| Kiribati | 730.7 (488.5-1014.6) | 1230.6 (826.5-1718) | 1938.5 (1282.5-2738.3) | 3143.5 (2049.2-4412.8) | 4477.3 (3027-6321.9) | 6071.9 (4345.8-8598.8) | 7805.3 (5660.9-11020.8) | 9677.2 (7138-12635.6) | 10867.8 (8079.9-14585.5) | 12754.8 (9541.7-16294.8) | 15696.8 (11822.9-20296.1) | 17910.1 (13208.7-23389.1) | 22320.2 (16640.5-28409.8) | 24732.9 (18551.5-31688.7) | 32532.9 (23236.6-42902.2) |
| Kuwait | 200 (152.4-248.8) | 314.2 (239.9-393.9) | 448.3 (331-574.4) | 707.6 (532.7-921) | 1066 (773.7-1401.5) | 1620.9 (1167.5-2132.5) | 2308.6 (1656.7-3038.3) | 3319.2 (2400.5-4323.7) | 4374 (3198.6-5730.3) | 5889.4 (4427.9-7589.4) | 7543.6 (5600.5-9713.7) | 10187.4 (7589.2-12931.5) | 14048.8 (10728.8-17895.9) | 18433.9 (13393.9-23271.3) | 20698.9 (13489-27086.2) |
| Kyrgyzstan | 131.3 (95.4-171.1) | 300.8 (224.5-391.1) | 546.2 (403.5-709.7) | 890.1 (652-1160.5) | 1482.2 (1093.6-1901.4) | 2475.6 (1825.6-3157.9) | 4015.3 (2991.1-5115.2) | 6334.9 (4735-8110.8) | 9241.6 (6951.3-11627.1) | 12527.2 (9525.3-15765.2) | 18209.3 (13695-22949.5) | 22680.4 (17052.2-28689.7) | 26399.3 (19414.2-33775.2) | 34245.3 (24831.1-44046.9) | 59310.1 (42419-77393.4) |
| Lao People's Democratic Republic | 525.3 (314.4-765.2) | 789.3 (508.6-1156.3) | 1237.8 (773.8-1779.2) | 2069.9 (1332.5-2895.6) | 3169 (2114.8-4432.2) | 4909.8 (3201.5-6702.7) | 7052.8 (4757-9570) | 9870.3 (6278.1-13445.4) | 13517.6 (9072.1-18136.1) | 17897.9 (12093.4-23353.9) | 22591.7 (15811.6-29026.3) | 27429.1 (18587.2-34863.5) | 32108.3 (22282.5-40853) | 39117.3 (26725.4-50327.5) | 48868.8 (32955.6-61722.8) |
| Latvia | 19.2 (12.3-28.6) | 46 (29.3-70.3) | 94.5 (59.2-141.3) | 194.4 (123.2-293) | 357.1 (230.6-527.1) | 654.4 (426.2-979) | 1007.4 (654.3-1491.2) | 1521.3 (1008.2-2277.5) | 2134.6 (1385.2-3166.6) | 2943.3 (1942.4-4386.4) | 3919.1 (2556.9-5909.3) | 5697.3 (3720.8-8431.8) | 8673.1 (5621.2-12860.1) | 12120.1 (7883.1-17785.5) | 17512 (11026.9-26130.6) |
| Lebanon | 71 (42.9-105.3) | 105.6 (65.9-154) | 159 (100.6-230.4) | 265 (165.7-382.1) | 428.1 (269.2-627.5) | 630.5 (406.2-913) | 887.5 (546.5-1276.5) | 1248.1 (770.9-1823.8) | 1710.5 (1087.7-2475.6) | 2387.8 (1535.3-3532.2) | 3629.3 (2324.6-5313.7) | 5293.3 (3360.4-7707.9) | 7558.9 (4767.4-11045.9) | 11022.3 (7179-15584.6) | 14406.4 (9239.5-21313.3) |
| Lesotho | 143.8 (89.4-208.9) | 256.9 (151.5-376) | 548.2 (336.7-825.6) | 1205.7 (677.3-2043.5) | 2386.7 (1396.1-4011.1) | 3892.3 (2247.5-6301.2) | 5622.6 (3519.6-8680.4) | 8494 (5466.9-12338.9) | 11507.2 (7867.4-15859.8) | 14974.9 (10321-19762.9) | 19578.3 (13940.3-26592.1) | 23510.3 (16856.5-30930.8) | 26222.7 (19159.1-33713) | 16354.3 (11072.6-22353.8) | 8280.8 (5259-12143.6) |
| Liberia | 240.8 (164.8-335.8) | 409 (280.1-562.5) | 658.2 (462.5-922.6) | 1254.6 (872.3-1755.2) | 2152.3 (1486.6-3025) | 3668.1 (2605-5162.9) | 5445.4 (3899.7-7563.8) | 8332.3 (5944.3-11461) | 12163.4 (8901.8-16023.8) | 15596.2 (11618.6-20463.9) | 20018.4 (15359.8-25959.2) | 23604.7 (18667.1-29564.4) | 26691.1 (20961-33678.2) | 32439.4 (25606.4-40717.5) | 41588.7 (31958.4-51533) |
| Libya | 321.1 (207.3-456) | 463 (300.9-675.1) | 675.3 (445.6-960.5) | 1018.7 (702.2-1451.4) | 1597.5 (1073.8-2289) | 2126.9 (1418.5-3066.5) | 3026.3 (2028.1-4337.1) | 4842.4 (3299-6855.5) | 6636.8 (4480.8-9286.6) | 8324.7 (5735.5-11662.6) | 11102.9 (7776.1-15262.2) | 13048.6 (9083.3-17913.3) | 17203.5 (12038.3-23312.4) | 21387.4 (14370.5-28899.5) | 20820.3 (13117.7-29645.4) |
| Lithuania | 9.6 (5.6-13.8) | 31.9 (18.7-47.1) | 74.8 (45.6-111) | 162.6 (96.9-238.8) | 290.2 (176-428.1) | 451.2 (269.1-651.3) | 673.3 (407.7-976) | 1034.8 (618.4-1508.2) | 1489.1 (875.2-2146.3) | 2034.6 (1235.2-2928.4) | 2812.4 (1703.8-4048.6) | 4103.3 (2465.8-5884.7) | 6736.9 (4018.7-9693.2) | 10629.1 (6276.9-15344.8) | 17597.8 (10332.8-25366.2) |
| Luxembourg | 3 (1.7-4.5) | 5.5 (3.1-8.3) | 11.6 (6.6-17.4) | 25 (13.9-38.1) | 43.5 (24.5-65.7) | 71.7 (41-108.7) | 114.2 (62.9-169.7) | 170.2 (95.6-255.2) | 262.2 (147.9-387.9) | 393.1 (221.5-580.6) | 546.5 (305.5-804.7) | 899.7 (501.4-1325.7) | 1434.3 (790-2118.4) | 2236.9 (1243.2-3331.8) | 2658 (1422.6-3988.4) |
| Madagascar | 501.2 (340.7-702.4) | 818.7 (535.3-1138.6) | 1156.9 (783.5-1604.8) | 2066.3 (1433.3-2856.9) | 3224.9 (2281.9-4657) | 4958.3 (3535.2-6859) | 7487.2 (5378.4-10183.8) | 11086.8 (8003.7-14672) | 15001.7 (11364.4-18993) | 19374.4 (14469.4-24529.3) | 23526 (17659.9-30233.1) | 25576.8 (18894.7-32525.3) | 27139.1 (19538.2-36181.6) | 31086.9 (22714.9-40937.6) | 32562.1 (22226.6-42307.2) |
| Malawi | 259.2 (178.8-357.8) | 466.1 (327.4-637.4) | 696.8 (492.5-949.3) | 1411.3 (1011.9-1901) | 2324 (1671.1-3138.1) | 3645.8 (2616.8-4851.5) | 5509.8 (4141.6-6902.4) | 8366.3 (6271.9-10630.1) | 11458.7 (8960.7-14714.3) | 15220 (12138.4-19088.2) | 19112.8 (14671.3-23715.4) | 21899.3 (17179.1-27834.2) | 23552.9 (17766.7-30472.4) | 25648.1 (18122.3-33966.8) | 17669.3 (11251.6-24481.8) |
| Malaysia | 78.6 (51.1-111.1) | 155.1 (95.5-222.9) | 282.7 (184.5-401.1) | 521.5 (338.5-735.1) | 864 (562.3-1193.2) | 1368.5 (930.3-1887.5) | 1975.2 (1302.8-2761.6) | 2662.5 (1786.3-3677.7) | 3440.7 (2323.1-4749.8) | 4498.1 (3059.3-6187.8) | 6104.9 (4168.8-8359.5) | 7109.1 (4801.4-9848) | 8039.9 (5285.1-11033.6) | 10039.7 (6465-14217.3) | 12259.4 (7709.1-17176.5) |
| Maldives | 58.9 (31-93.4) | 91.3 (48.9-148.7) | 129 (69.4-206.4) | 183 (102.6-296.9) | 253.9 (143.6-397.8) | 396.2 (217.1-637.6) | 537 (296.5-843.9) | 778.1 (434.5-1228.6) | 1120.2 (629.9-1823.1) | 1880.6 (1041.8-2965.2) | 2637 (1497.3-4145.5) | 3863.3 (2151.6-6102.9) | 5106.7 (2800.2-7892.4) | 6831.2 (3774.2-10586.3) | 8107 (4392.6-12934.9) |
| Mali | 218.7 (146.8-304.8) | 382.2 (265.5-515.1) | 534.7 (385.3-728.4) | 910.2 (634.9-1220.8) | 1521.7 (1083.6-2037.5) | 2704.7 (1908.4-3551.4) | 3738 (2663.3-4985.8) | 6002.8 (4343.2-7720) | 8898.6 (6599-11515.1) | 12096.1 (8913-15593.6) | 15395.8 (12129.9-19361.4) | 17426.9 (13588.1-21727.4) | 18918.1 (14656.9-23611.8) | 21743.5 (16496.9-27324.1) | 22865.5 (16234.3-30220.2) |
| Malta | 7.8 (5.5-10.4) | 25.4 (18.3-34.3) | 37.6 (26.4-51.4) | 99.4 (69.6-131.5) | 160.9 (110.1-219.6) | 201.8 (142-271.7) | 359.1 (247-479.4) | 470.3 (328.5-626.2) | 621.9 (430.4-821.7) | 976.6 (682.7-1296.1) | 1317.5 (909-1749.5) | 2426.4 (1685.9-3208.9) | 3742.4 (2496-5016.3) | 3950.1 (2541.1-5404.9) | 5563.6 (3470.1-7777.7) |
| Marshall Islands | 558.5 (351.8-870.5) | 988 (606.6-1529.1) | 1615.3 (1023.6-2499.6) | 2738.2 (1730-4247.6) | 3987.6 (2479.9-6240.2) | 5214.6 (3430.5-8124.9) | 6516.7 (4118-9994) | 8213.4 (5309.8-12380.1) | 9634.3 (6331.8-14397) | 11798 (7984-17881.8) | 14252.2 (9394.4-21305.7) | 16656.7 (11428.8-23465) | 21102.6 (14494.1-29649.9) | 24392.8 (16340.5-34261.4) | 33805 (22387.4-48031.5) |
| Mauritania | 139.2 (86.1-220.2) | 261.2 (156.1-401.9) | 409.5 (240.5-640) | 827.9 (507.9-1243.1) | 1532.6 (980.3-2258.7) | 2834.5 (1911.5-4035.1) | 4155.8 (2897.2-5610.5) | 6643.7 (4763.5-8906.2) | 9846.6 (7021.9-12955.3) | 13615.9 (10307.2-17634.6) | 17494.1 (13215.7-22691.6) | 21350.3 (15986.7-27563.3) | 25843.8 (19345.4-33485) | 31039.1 (22877.2-39828) | 39707.9 (28679.8-51454.5) |
| Mauritius | 55.5 (18.4-97.6) | 105.6 (37.5-186) | 160.4 (55.3-283.5) | 213.6 (73.4-383.8) | 307 (106.5-549.2) | 386.2 (138.6-697.6) | 491.1 (169.2-894.8) | 670.5 (226.2-1169.4) | 896.6 (310.9-1610.7) | 1138.1 (402.2-2055.7) | 1490.8 (527.2-2674.6) | 2139.2 (732.3-3759) | 2481.8 (851.2-4382.1) | 3613.7 (1254.5-6478.2) | 3658.9 (1234.3-6612.3) |
| Mexico | 78.1 (51.1-109.8) | 114.3 (74.8-161.8) | 165.6 (108.5-233.9) | 252.7 (161.6-357.5) | 384.4 (245-547.6) | 563.3 (359.1-790) | 797.3 (506.3-1138.1) | 1097.9 (694.8-1571.5) | 1485.8 (942.5-2120) | 2008.1 (1274-2892.2) | 2752.8 (1766.1-3974) | 4026.7 (2598.3-5707) | 6157.7 (3924.2-8811) | 9654.4 (6162.4-13858.7) | 15012.3 (9322.6-21719) |
| Micronesia (Federated States of) | 552.3 (334.6-844.2) | 1006.2 (596.1-1539.1) | 1617.3 (982.1-2505.1) | 2807.7 (1681.2-4360.1) | 4039.5 (2411.9-6338.8) | 5322.7 (3283.5-8243.8) | 6622.4 (4059.3-10076.7) | 8378.1 (5314-12524.1) | 9784.8 (6264.8-14838.6) | 11703.1 (7415.6-17462) | 14240.2 (9046.6-20369.8) | 16916.2 (10987.2-24554) | 21617.7 (14169.1-31459.5) | 24900.7 (16198.8-35656.9) | 33969.6 (21508-49087.9) |
| Monaco | 12 (5.7-21.7) | 22 (9.7-39.1) | 37.7 (16.7-68.7) | 62.4 (29.9-110.5) | 92.8 (44-159.4) | 132.5 (63.7-227.7) | 184.3 (89-306.1) | 254.8 (125.9-417.3) | 366 (184.6-600.5) | 557.8 (279.2-888.2) | 869.1 (440.4-1398.2) | 1361.2 (666.9-2170.3) | 2219.4 (1131.9-3498.6) | 3303.8 (1669.1-5166.1) | 4091 (1978.7-6694.3) |
| Mongolia | 123.5 (81-173.9) | 269.5 (176-383.3) | 575.3 (383.3-800.8) | 1170.5 (795.8-1710.2) | 2051.7 (1390.9-2853.7) | 3067.2 (2140.4-4224.4) | 4252.1 (2921.5-5771.7) | 6203.2 (4331-8444.7) | 8118 (5753.5-10959.7) | 10661.7 (7332.9-14240.2) | 15425.6 (10735.2-20829.3) | 22888.6 (15947.6-30415) | 30629.6 (20726.1-40972.4) | 37559.8 (24309.6-50847.7) | 35184.4 (20816.6-49099.4) |
| Montenegro | 65.6 (41.2-111.6) | 120.5 (79-201.6) | 202.6 (131.6-328.6) | 377.4 (247-641.1) | 718.2 (472.5-1209.6) | 1019.8 (656.1-1691.4) | 1883.1 (1250.6-3130.7) | 2975.5 (2005.5-4981.6) | 4615.6 (3126-7564.2) | 6660.9 (4577.1-11229.6) | 11387.4 (7669.5-19539.4) | 17806.6 (12014.8-30717.9) | 31141.1 (20962.2-52575) | 43125.1 (28934.3-72356.2) | 34485.2 (22577-57540.2) |
| Morocco | 120.8 (75.5-207.5) | 202.2 (125.6-333) | 351.4 (219.3-554.9) | 682.3 (440.4-1084.2) | 1251.6 (813.9-1875.8) | 2072.7 (1359.1-3023.2) | 3208.2 (2091.5-4533.2) | 4659.9 (3057.7-6314.8) | 6544.5 (4470.6-8525.4) | 8608.2 (5870.5-11191.3) | 11466.1 (8068.5-14920.8) | 14278 (10230.1-18632.2) | 18090.1 (12633.1-23684.2) | 24013.4 (16953.7-31953.8) | 35317 (23729.7-47323.8) |
| Mozambique | 220.2 (144.3-336.1) | 426.7 (261-612.3) | 746.1 (468.7-1131.7) | 1678.3 (1042.6-2448.2) | 2964.8 (1913.2-4259.2) | 4617.2 (3095.8-6250.1) | 7306.3 (5133.7-9789.9) | 11160.8 (8195.1-14808.8) | 14945.1 (10841.7-18983.4) | 18474.4 (14019.7-23468.7) | 22332.3 (16237.6-29005.8) | 22047.5 (16137.4-28494.7) | 21016.4 (14364.3-28310.6) | 23325.8 (15672.2-33303.8) | 16383.1 (9739.1-24961.1) |
| Myanmar | 439.5 (298.5-601.4) | 660.7 (457-907.4) | 1049.7 (756.2-1417.4) | 1756.9 (1205.8-2373.1) | 2730 (1957.8-3710.5) | 4444.9 (3190.7-5795) | 6284.6 (4469.3-8189.5) | 8727.3 (6317-11421.9) | 12046.6 (8591.1-15795.8) | 16398.2 (12132.9-21259.8) | 20732.7 (15523.8-26480.9) | 25554.7 (19035.7-33128.1) | 28797.3 (21490-37957.3) | 34506.2 (25371.2-44470) | 42962.2 (30742.5-55436.7) |
| Namibia | 61.1 (25.3-116.7) | 117 (49.6-231.8) | 244.7 (112.4-451.4) | 530.1 (253.5-937.8) | 1028 (523.9-1712.4) | 1783.9 (910.5-3010.9) | 2671.9 (1386.3-4519) | 4194.4 (2181.9-7123.7) | 5848.3 (3072.8-9494.9) | 8038.1 (4459.4-12838.2) | 10990.8 (5996.4-17565.3) | 14056.2 (7606.1-22058.9) | 16962.4 (8947.3-26782.4) | 17465.1 (9307.7-27302.9) | 10959.2 (5243.8-18801) |
| Nauru | 143.2 (17.3-332.1) | 269.8 (32.7-655.3) | 431.6 (52.3-1028) | 761 (92.7-1821.2) | 1116.8 (130.6-2565.4) | 1453.1 (177.7-3213.7) | 1783.1 (212.1-3986) | 2115.3 (276.6-4759) | 2405.8 (298.6-5464.6) | 2831 (369-6046.1) | 3356.1 (439-7285.7) | 3729.5 (490.8-8122.1) | 4499.7 (641.3-9608.2) | 5183.6 (749.9-11026.1) | 7462.4 (984.4-16490.9) |
| Nepal | 233.1 (151.7-340.8) | 419.3 (267.2-611.5) | 634 (408-888.6) | 1168.8 (797.5-1613.7) | 1967.3 (1366.1-2708.7) | 3165.2 (2251.1-4255.9) | 4872.9 (3489.7-6279.7) | 6767 (5034.5-8857.7) | 9346.4 (6937.1-11922.2) | 12095 (9194.5-15319.4) | 15261 (11917.2-19253.4) | 17603.9 (13586.3-22758.5) | 20700.9 (15648.5-26565.1) | 26406.2 (19735.3-33747.7) | 30882.9 (22072.8-40704.2) |
| Netherlands | 6.3 (4.2-8.6) | 11.3 (7.9-15.7) | 20.9 (14.6-28.5) | 37.7 (26.6-51.8) | 66.3 (46.2-91.1) | 103.9 (72.7-140.4) | 153.2 (108-207.4) | 226.8 (160.2-305.6) | 339.9 (237.2-451.5) | 505.4 (354.6-666.6) | 853.1 (598.3-1136.5) | 1271.6 (876.3-1718.5) | 2204.4 (1529.6-3007) | 3735.9 (2543-5067.3) | 5590.4 (3743.1-7565.5) |
| New Zealand | 3.8 (1.5-6.3) | 8.7 (3.5-14.5) | 17.6 (6.9-29.7) | 35.1 (13.6-58.6) | 55.9 (22.7-93.3) | 91.4 (36.3-154.5) | 124.4 (49.4-212.6) | 166.3 (64.9-277.8) | 226.6 (90-378.6) | 311.4 (124.3-526.1) | 497.2 (200.7-832.5) | 775 (307.3-1293.9) | 1393 (562-2358.4) | 2316.7 (910-3917.8) | 3607.9 (1405.7-6196.7) |
| Nicaragua | 96.8 (67.1-129.4) | 144.8 (100.8-194.8) | 258.1 (182.4-349.6) | 349.3 (245.6-474.6) | 548.3 (384.5-735.8) | 859.1 (609.3-1166.5) | 1346.5 (977-1825.1) | 2090.4 (1544.6-2853.9) | 2803.1 (2054.6-3664.7) | 3803 (2833.7-5052.4) | 4849.5 (3582.5-6331.8) | 6202.7 (4436-8058.5) | 7863.2 (5581.9-10379.1) | 12911.8 (8895.1-16886.9) | 41150.9 (29717-52875.6) |
| Niger | 144.3 (96.2-204) | 262.6 (174.9-371.9) | 416.1 (286.3-580.8) | 833 (548.9-1185) | 1501.2 (1002.7-2149) | 2927.9 (1998.7-4100.2) | 4242.5 (2907.2-6000.8) | 6885.5 (4738.9-9591.7) | 10105.7 (7214-13800.3) | 13247.1 (9627.9-17884.3) | 17443.4 (13266-22402.6) | 20267 (15243.8-25590) | 21807.2 (16388.7-27897.3) | 25789.2 (19176.8-32949.8) | 32579.9 (23487.3-41818.8) |
| Nigeria | 105.3 (76.7-139.1) | 217.4 (152.6-285.3) | 363 (267-498.4) | 690.1 (492.2-956.6) | 1194.1 (877.3-1628.1) | 2176 (1599.4-2973.1) | 3124.1 (2297.8-4277.1) | 5176.2 (3776.1-7022) | 7804.3 (5888.7-10417.8) | 11027.7 (8586.6-14080.2) | 15010.3 (12067.3-18400.4) | 18281.3 (14864.6-22379.4) | 22102.7 (17598.2-26571.8) | 26695.5 (21044.7-32566.7) | 30601.6 (22703.2-37641) |
| Niue | 73.6 (7.7-166.2) | 118.9 (10.5-262.7) | 168.2 (15.7-386.8) | 292.2 (26.7-676.2) | 450.4 (40.3-1061.8) | 662.7 (60.4-1473) | 880.2 (79.2-1991.6) | 1194.7 (113-2671.6) | 1476.2 (143.3-3232.6) | 1907.2 (186.6-4286.2) | 2437.1 (248.8-5400.4) | 2999.1 (308.8-6599.6) | 3881.1 (384.1-8313.6) | 4682.5 (481.5-10072.5) | 6561.7 (685.1-14322) |
| North Macedonia | 62 (43.7-83.4) | 119 (85-169.4) | 230.2 (166.1-316.4) | 437.4 (312.6-595) | 781.2 (537.7-1077) | 1358.8 (901.9-1892) | 2298.5 (1645.6-3127.7) | 3845.8 (2808.6-5135.1) | 5893.7 (4352.9-7837) | 8422.5 (6208.9-10942.4) | 14423.6 (10786.8-18557.8) | 26014.4 (19619.6-33938.7) | 44779.1 (34085.5-57095.9) | 63071.9 (47824-81118.7) | 47707.6 (35166.6-62379.2) |
| Northern Mariana Islands | 50 (22.8-86.1) | 101.2 (46.3-169.9) | 178.4 (80.4-305) | 369.7 (169.5-598.5) | 459 (212.9-750.9) | 849.8 (399.2-1384.5) | 938.6 (431.8-1534.9) | 1399.5 (666.1-2251.4) | 1601.6 (755-2653.2) | 1989.6 (929.5-3206.7) | 2640.8 (1239-4223.9) | 3055.8 (1436.9-4900.4) | 4467.1 (2076.1-7367.1) | 5323.3 (2527.5-8723.2) | 8037 (3824.7-13209) |
| Norway | 1.6 (0.6-3) | 2.7 (1-4.9) | 5.8 (2.1-10.6) | 12.1 (4.7-21.6) | 22.6 (8.7-39.8) | 36.5 (13.4-64.4) | 59.3 (21.6-104.3) | 81.5 (30.1-145.1) | 118 (43-210.3) | 173.7 (63.6-310.6) | 272.3 (100.6-481.2) | 425.7 (155.9-750.9) | 703.6 (259.9-1245.1) | 1134.2 (413-2023.3) | 1532.4 (568.7-2784.8) |
| Oman | 149.6 (106.4-206.9) | 205.1 (141.8-287.3) | 317.2 (221.6-423.1) | 568.4 (383.3-768) | 1004.7 (681.3-1370.5) | 1819.4 (1169.5-2430) | 3001.2 (1989.1-4075.4) | 4551.2 (3006.6-6025.9) | 7460.3 (5049.7-9880.9) | 10447.4 (7431.7-13778.3) | 14183.2 (10114.1-18322.7) | 16205 (11482.8-21248.2) | 25005.4 (18015.6-32606.4) | 23553.8 (16130.6-31043.9) | 22882.6 (14430.3-31505.6) |
| Pakistan | 437.3 (322.7-583.8) | 732.9 (541.5-950.2) | 1102.2 (793.3-1431) | 1769.1 (1297-2311.7) | 2652 (1942-3466.4) | 4023 (3035.9-5174.4) | 5995 (4493.3-7793.9) | 8413.7 (6429.6-10665) | 11651.5 (9027.8-14599.6) | 14778.2 (11793.7-18191.8) | 17791.2 (13942.9-21865.6) | 19730.9 (15382.9-24723.7) | 21407.5 (16380.9-26743.2) | 27420.7 (20917.4-34218.3) | 35331.5 (25558.2-45176.5) |
| Palau | 101.3 (11.8-210.5) | 193.2 (20.6-420.5) | 287.1 (30.1-617.4) | 424.8 (50.6-909.7) | 569.3 (63.9-1189.3) | 728.4 (87.5-1539) | 854.5 (95.3-1778.4) | 1153.7 (125.6-2424.7) | 1342.1 (147.8-2773.5) | 1823.8 (202.6-3872.7) | 2236.1 (232.9-4696.6) | 2769.9 (275.1-5747.1) | 4004.4 (422.4-8164) | 5244.5 (554.8-10592.2) | 8058 (793.7-16658.2) |
| Palestine | 140.8 (91.6-199.3) | 196.1 (128.8-264.1) | 285.3 (188.1-392.9) | 474.1 (318.3-632.8) | 822 (556.9-1110) | 1513 (1024.6-2022.1) | 2521.9 (1727.1-3345.9) | 4043 (2713.2-5323.4) | 5386.4 (3685.8-7086.6) | 8112.5 (5590.3-10626.2) | 10432.9 (7160.4-13759.9) | 15134.9 (10400.6-19699.5) | 21369.7 (14360.6-27934.9) | 32757.4 (22910.4-42792) | 45832.2 (30405.6-60490.7) |
| Panama | 25.5 (13.5-41.8) | 46.6 (25.7-74.8) | 72.3 (38.7-116.3) | 109.8 (58.1-182.3) | 171.1 (89.3-279.3) | 277.8 (148.4-459) | 404.5 (214.8-665) | 550.1 (296.2-895.7) | 769 (409.4-1275.1) | 1081.8 (583.2-1752.6) | 1511.4 (815.7-2415.9) | 1949.6 (1067.4-3126.8) | 2422.8 (1313.8-3908.5) | 3425.4 (1816.8-5574.8) | 4502.8 (2315.6-7558.4) |
| Papua New Guinea | 463.5 (284.2-696.9) | 780.2 (472.4-1199.7) | 1286.2 (836.4-1873.6) | 2390.8 (1613.8-3406.4) | 3703.8 (2508-5177.3) | 5567 (3763.1-7601) | 7879.3 (5504.4-10664.3) | 10746.4 (7580-14318.4) | 13784.2 (9826.8-18289.6) | 17749.2 (13042.1-22969.1) | 20753.4 (15191.3-27047.9) | 23051.3 (17062.3-29808.3) | 26238.3 (18530.3-34822.3) | 29413 (21214.7-38292.7) | 37065.8 (23909.9-50600.1) |
| Paraguay | 37.9 (11.2-73.3) | 70.8 (22.9-132) | 129.1 (41.1-246.6) | 252.1 (78.1-483.6) | 441.1 (140.7-815.4) | 751 (242.5-1400.5) | 1120.6 (367.4-2105.1) | 1578.9 (505.8-2955.9) | 2115.2 (680.8-3945.5) | 2903.9 (951.6-5345.6) | 3678 (1217-6722.7) | 4507.9 (1383.5-8079) | 5596 (1726.8-9992.7) | 6993.1 (2188.9-12203.7) | 7832.4 (2311-14089.5) |
| Peru | 119.2 (82.1-173.9) | 156.2 (100.3-230.2) | 203.8 (134.2-288.7) | 291 (188.9-436.2) | 409.3 (264.7-608.6) | 599.1 (394-885.6) | 812 (531.4-1217.2) | 1154.4 (762.3-1643.9) | 1563.6 (1049.6-2214.7) | 2217.7 (1518.5-3159.2) | 2925.9 (1903-4154.2) | 4123.4 (2851.3-5708.8) | 4943.7 (3262.7-7028.4) | 6693.3 (4518.5-9304.7) | 8739.7 (5771.3-12618.7) |
| Philippines | 397.6 (295.2-510.1) | 626.6 (466.6-802.6) | 972.9 (722.7-1251.9) | 1527.8 (1119.9-1976.9) | 2290.6 (1649.8-2966.6) | 3284.4 (2359.1-4236.1) | 4352.8 (3115.5-5618.2) | 5767.2 (4161.4-7368.5) | 7498.8 (5471.9-9516.3) | 9658.7 (7098.8-12161.3) | 12096.5 (8880.5-15113.6) | 14465.2 (10727.8-18176.6) | 16374.4 (11964.7-20682.6) | 21995.9 (15856.2-28129.1) | 23972.2 (16885.4-30711.9) |
| Poland | 23.3 (18.1-30.4) | 51.8 (40.4-67.4) | 93.1 (73.6-119.6) | 185 (144.7-235.8) | 355.8 (274.6-458) | 631.5 (485.8-804.7) | 979 (752.5-1259.9) | 1515.7 (1160.4-1932) | 2135.8 (1635.8-2741.1) | 2859.6 (2174.3-3666.7) | 4033 (3070.5-5142.9) | 6033.3 (4605.1-7767.1) | 9456.3 (7176.7-12317.1) | 13450.8 (10083.2-17511.4) | 20411 (14740.7-26813) |
| Portugal | 6.1 (3.4-9.6) | 12.3 (6.6-18.9) | 20.5 (11.3-31.9) | 34.1 (18.4-52.5) | 63.6 (33.6-97) | 98.7 (53.8-149.6) | 139.4 (75-212.8) | 190.5 (102.7-290.2) | 251.3 (136.2-382.9) | 336.3 (184.9-511.3) | 500.4 (269.7-759.8) | 762.6 (407.2-1168.8) | 1192.9 (634.4-1877.8) | 1983.6 (1058-3073.1) | 3213.3 (1703-5018.1) |
| Puerto Rico | 6.1 (1.6-11.4) | 11.7 (3.1-21.8) | 21 (5.1-39.7) | 34.8 (8.7-64.5) | 58.5 (14.9-111.4) | 95.5 (23.1-181.5) | 130.1 (32.9-247.3) | 167.5 (42.1-304.5) | 211.9 (53.8-394.4) | 280.5 (73-536.9) | 382.2 (99.2-711.3) | 463.2 (120.3-859.9) | 665.1 (169.3-1233.1) | 850.9 (218.4-1573.5) | 857.8 (217.2-1613.8) |
| Qatar | 117.3 (82.4-164.4) | 161.3 (113.9-225.2) | 252.5 (172.6-334.7) | 449.9 (316.5-615) | 761 (514.4-1067.7) | 1231.2 (806.3-1716.2) | 1901.8 (1245.3-2719.6) | 2815.2 (1845-4043.1) | 4665.6 (3188.8-6456.2) | 8693.2 (5963.9-11666.5) | 12652.5 (8819.8-16774.6) | 17082.6 (12569.7-21672.9) | 24191 (18218.3-31514.7) | 31202 (22351.1-40117.6) | 40258.6 (27171.7-53369.4) |
| Republic of Korea | 30.7 (20.2-43.9) | 51.5 (35.4-72.1) | 92 (60.7-130.7) | 142.9 (94.2-193.7) | 232.7 (157.8-315.7) | 324.6 (214.8-441) | 438.7 (302.1-606.9) | 576.4 (391.4-769.5) | 807.1 (551-1099.5) | 1230.4 (839.2-1635.7) | 2182.6 (1477.4-2971.1) | 3484.5 (2362.4-4677.5) | 5318.6 (3452.5-7384.2) | 8631.9 (5491.6-11856) | 12944.2 (8145.4-18216.3) |
| Republic of Moldova | 74.4 (46.3-105.9) | 139.9 (90.6-198.7) | 237.7 (150.6-338.3) | 498.3 (318.3-699.8) | 746.7 (483.5-1084.7) | 1229.4 (801-1765) | 1944.6 (1252.9-2788.1) | 3287.8 (2116.8-4714.5) | 4981.9 (3202.5-7007.5) | 7769.6 (4961.3-11027.7) | 9085 (5798.5-12872.5) | 10711.5 (6879.1-15414.6) | 12713.6 (7944-18517.5) | 11610.5 (6572.4-17512.4) | 11357.2 (6377.8-17601.7) |
| Romania | 48.8 (34.6-69.6) | 91 (65.5-128.4) | 155.2 (108-219) | 280.1 (198.9-397.5) | 501.4 (357-703.2) | 849.7 (597.2-1179.9) | 1323.5 (934.4-1868.1) | 1974.1 (1395.2-2843.6) | 2610.2 (1876.8-3718.2) | 3729 (2680.1-5351.4) | 5643.9 (4068.3-8044.9) | 8178.7 (5863.4-11591.2) | 11448.7 (8261.7-16144.3) | 13580.6 (9618-19295.2) | 17731.2 (11929.3-25110.9) |
| Russian Federation | 41.6 (25.3-63.5) | 93.9 (55.9-143.9) | 176 (105.6-273.9) | 335.5 (200.4-523.4) | 536.8 (319.5-847.6) | 836.1 (496.5-1331.6) | 1276.3 (749.3-2014.2) | 1861.7 (1096.6-2940) | 2497.3 (1476.4-3913.4) | 3340.2 (1984.1-5231) | 4972 (2985.4-7754) | 6792.7 (4082-10444.6) | 8207.3 (4943.6-12508.3) | 10554.5 (6190.5-16065.1) | 13428.7 (7638.9-20558.3) |
| Rwanda | 163.6 (106.5-248.7) | 292 (194.2-442.4) | 445.8 (288.3-634.9) | 841.8 (575.6-1185.5) | 1419.2 (966.6-2030.3) | 2385 (1653.5-3294.8) | 3846 (2610.2-5258.5) | 6344.3 (4471.5-8596.8) | 8933.8 (6468.4-11919) | 12439.9 (9201.3-16283.8) | 16219.2 (11987.9-21047.8) | 19031.8 (13736.3-24955.1) | 21038.5 (14463.1-28909.4) | 25245.1 (16537.2-35501.3) | 26509.7 (16041.1-37674.1) |
| Saint Kitts and Nevis | 8 (2.5-14.7) | 13 (4-24.1) | 27.1 (8.4-50.4) | 63 (21.2-116.6) | 133 (46.5-240.8) | 255.2 (92.1-469.1) | 364.8 (129.4-655.5) | 556.8 (194.9-1007.2) | 780.4 (281.9-1424.9) | 1143.3 (401.1-2069.1) | 1566.2 (569.5-2681.9) | 2031.8 (723.3-3560.3) | 2608.1 (892.2-4442.5) | 3268.3 (1125.8-5686) | 4017.3 (1378.9-6893.8) |
| Saint Lucia | 44.6 (18.9-77.2) | 74 (31.4-127.6) | 109.8 (48-189.6) | 236.4 (97-411) | 428 (176.3-730.7) | 648.9 (273-1128.5) | 878.1 (363.1-1505.5) | 1328.9 (563.4-2269.6) | 1758.3 (742.1-2985.3) | 2619.4 (1116.4-4500.3) | 3301.2 (1406.6-5568.6) | 4564.6 (1930.2-7698.2) | 7112 (2973.9-11886.2) | 11780.4 (4856.7-20240.6) | 20525.8 (8432.4-35301.1) |
| Saint Vincent and the Grenadines | 66.2 (26.6-114.7) | 96.1 (39.8-173.1) | 219.2 (88.3-388) | 405.7 (162-706.6) | 684.3 (273.2-1203.7) | 1035.2 (411.9-1841.3) | 1499.5 (602.7-2626.2) | 1821.7 (738.2-3204.7) | 2293.1 (943.2-4016.6) | 3495.7 (1387.5-6154.4) | 5272.2 (2107.8-9348.3) | 6086.9 (2409.8-10847.9) | 11089.2 (4413.7-19717.2) | 16601.5 (6566-28838.4) | 28363.2 (11331.1-50226.8) |
| Samoa | 468 (266.7-717.6) | 849 (465.7-1282.3) | 1352.7 (799.7-2022) | 2442.8 (1452.2-3486.6) | 3658.3 (2291.1-5157.9) | 5094.7 (3298.1-7022.1) | 6625.2 (4313-9037.4) | 8863.9 (5871.9-11896.7) | 11023.8 (7429.3-14651) | 13883.3 (9226-18189.3) | 17426.9 (11664-22622.6) | 20722.2 (13869.7-27048.2) | 27255.7 (17307.8-35713.3) | 33134 (22200.7-43883.8) | 46173.6 (29800.1-61115.6) |
| San Marino | 5.5 (2.7-9.1) | 8.7 (4.2-14.4) | 14.3 (6.6-23.7) | 24.2 (10.7-43) | 41.2 (18.8-73.3) | 67.9 (31.4-118.9) | 101.7 (46.8-178.4) | 154.5 (71.7-267.3) | 231.9 (108.2-404.5) | 375.8 (185.6-633.9) | 591.6 (301.5-969.4) | 860.3 (439.7-1403.3) | 1158.7 (589.2-1892.3) | 1532.6 (755.2-2514.7) | 2784.8 (1384.5-4512.2) |
| Sao Tome and Principe | 161.5 (98.9-253) | 247.5 (150.8-401.2) | 404.3 (252.7-626.7) | 747.3 (482.8-1099.6) | 1408.5 (951.4-1971.7) | 2357.7 (1703.2-3149.8) | 3428.4 (2514.3-4527.6) | 5087.8 (3827.9-6800.9) | 7326.3 (5578.5-9203.8) | 9676.2 (7626.3-12342) | 12361.9 (9597.1-15330.6) | 15094.3 (11822.7-19547.4) | 18562.5 (14513.9-23348.9) | 23461.6 (18254.7-30331.2) | 33205.3 (24997.3-43378.9) |
| Saudi Arabia | 338.5 (215.4-484) | 590.4 (366.6-902.5) | 983.5 (636.7-1439.7) | 1881.6 (1210.9-2722.4) | 2986.6 (2035.3-4111.7) | 4345.8 (3095.1-5821) | 5898.3 (4298.4-7467.1) | 7505.7 (5661.1-9605.6) | 9323.6 (7082.1-11494.3) | 11269.8 (8553.6-13975.8) | 13918.1 (10727.7-17162.7) | 17268.5 (13042.4-21651.9) | 23041.1 (17340.9-28932) | 31536.9 (23580-40155.1) | 41906.2 (30952.6-54873.3) |
| Senegal | 187.6 (130.5-258) | 340.9 (240.1-460.5) | 541.9 (388.2-735.9) | 1078.9 (786.1-1486.3) | 1929.7 (1371.3-2659.8) | 3448.7 (2428.8-4601.2) | 5001.5 (3531.2-6698.5) | 7951.5 (5829.7-10515.1) | 11568.2 (8600.1-14975.3) | 15684.8 (11699.7-19986.2) | 19978.1 (15248.3-25521.1) | 24403.3 (19046.4-30792.6) | 28682.1 (21675.6-36786.4) | 35054.3 (26176.4-44951.4) | 43359.4 (32031.3-55414.1) |
| Serbia | 44.4 (31.9-64) | 83.6 (57.7-117.4) | 174.5 (124.2-245.9) | 332.9 (238.9-464.4) | 648.9 (461.5-892.1) | 1171.8 (850.2-1624.6) | 1864 (1372.6-2577) | 2904.7 (2146-4023.4) | 4517.7 (3310.3-6221.1) | 7632.3 (5540.6-10595.7) | 9814.3 (7362.1-13430) | 17617.8 (13338.8-24477.5) | 24287.8 (18381.5-33440.3) | 29771.4 (21609.9-40690) | 35137.2 (24034.8-50203.7) |
| Seychelles | 51.7 (16.8-97.1) | 55.1 (17.7-101) | 94.4 (32.3-170.4) | 216 (72.6-398.4) | 259.3 (91-475.9) | 364.7 (125.7-674.7) | 538.6 (187.9-1007.1) | 572.3 (192.7-1034) | 832.5 (297.3-1565.4) | 1214.4 (414.1-2215.8) | 1721.8 (601.2-3315.6) | 2081.3 (726-3858.6) | 2674.3 (928.8-4940.6) | 3648.9 (1267.2-6846.6) | 4686 (1580.3-8704.5) |
| Sierra Leone | 272.4 (182.6-385) | 487.8 (327.7-686.3) | 752.4 (503.8-1021.1) | 1440.8 (989.1-2008.5) | 2407.8 (1628.6-3365.7) | 4279 (2904.4-5917.5) | 6103.9 (4216.8-8270.7) | 9424 (6682.9-12592.9) | 13500.6 (9631.9-17779.5) | 17150.8 (12709.5-22450.7) | 21648.9 (16930-26992.6) | 25173.7 (19897.2-31009.2) | 28384 (22988.1-34480.3) | 33543.9 (26234.3-40702.6) | 42690 (32892.8-53001.2) |
| Singapore | 25.3 (13.2-38.1) | 36.3 (19.2-55) | 74.3 (39.3-111.2) | 93.1 (50.2-140.8) | 227 (121.6-342.2) | 293.5 (156.4-434.4) | 436.9 (230-651.1) | 635.2 (331.4-961.4) | 799.6 (429.4-1185) | 1116.2 (593-1663.5) | 1496.8 (803.5-2265.2) | 2046.8 (1089.8-3122.2) | 2456 (1320.3-3750.6) | 3210.3 (1690-5029.8) | 4664.5 (2536.4-7318.7) |
| Slovakia | 31.2 (22.3-42.1) | 56.3 (39.5-74.6) | 98 (68.2-134.5) | 181.4 (124.3-250.8) | 338.7 (236.5-461) | 623.8 (448.3-821.6) | 1024.5 (737.6-1349) | 1681 (1188.7-2228.1) | 2393.4 (1749.7-3121.6) | 3579.4 (2660.3-4599.8) | 5393.9 (3995.7-6854.2) | 8102 (5923.8-10308.3) | 12589.8 (9220.6-16171.9) | 15591.6 (11150.6-20060.9) | 15707 (10679.7-20908.9) |
| Slovenia | 8.3 (5.6-11.6) | 14.8 (10.4-19.8) | 24.5 (17.1-33) | 54 (36.6-74.4) | 98.1 (66.2-136.4) | 164.7 (111.4-225.7) | 243.5 (166.8-334.8) | 399.9 (273.3-542.9) | 606.8 (430.7-807.7) | 991.1 (703.4-1325) | 1428.4 (1014.4-1926.5) | 2191.9 (1553.9-2909.3) | 3397 (2404.4-4498.8) | 4455.1 (3100.5-5879.5) | 6764.3 (4522.7-9179.9) |
| Solomon Islands | 649.6 (453.1-899.6) | 1265.7 (844.8-1751.3) | 2245.8 (1605.4-3058.1) | 4336.5 (3021.3-5826.1) | 6973.4 (4853.3-9842.5) | 9963.7 (7002.6-13496.1) | 13073.5 (9766.7-17725) | 16430.6 (12476-21867.3) | 20628.2 (15889.8-27024.8) | 25848.5 (20504.5-33179.2) | 32288.3 (25609-40332.6) | 37658.1 (29564.4-45905.7) | 45104.6 (36710.5-54545.3) | 51465 (40518.4-61678) | 70272.8 (54320.8-84517.9) |
| Somalia | 219.2 (141.3-311.9) | 410.9 (257.9-640.6) | 642.6 (392.9-965) | 1386 (816.8-2152) | 2227.9 (1371.3-3650.5) | 3678.9 (2337.1-5552) | 5891.6 (3905.9-8696.8) | 9060.1 (6249.3-12632.3) | 11996.7 (8415.3-16187.6) | 15394.6 (10884.6-20716.6) | 18357.5 (12886.7-24965.6) | 18356.8 (13009.7-24696.7) | 18666.7 (13559.4-24848.8) | 15736.2 (10309.8-22399.3) | 7848.2 (4874.4-11786.9) |
| South Africa | 72.9 (52.5-95.6) | 154.6 (115-204.6) | 256.5 (189.9-334.9) | 427.7 (311.6-568.1) | 660.2 (462.9-915) | 1108.2 (792.6-1474.5) | 1595.4 (1155.1-2099) | 2412.2 (1738.2-3188.1) | 3230.4 (2357.9-4230.2) | 3751.9 (2740.8-4875.9) | 4953.2 (3579.6-6441.7) | 7554 (5276.3-9636.5) | 10402.7 (7348.8-13487) | 14055.3 (9820.9-18366.7) | 14501.1 (9818.4-19572.5) |
| South Sudan | 198.5 (132.6-295.7) | 352.7 (219.3-533.3) | 563.9 (367.4-860.9) | 1134.9 (730.7-1715.1) | 1874.3 (1229.9-2737) | 3143.3 (2053.4-4680.5) | 5117.6 (3457.3-7333.8) | 8062.8 (5485.2-11785.6) | 11206.5 (7653-15652.1) | 14173.7 (10177.1-19511.1) | 16616.8 (12040.5-22778) | 17784.6 (13289.6-23529.7) | 17999.3 (13131.9-23130.6) | 20173.8 (15091.1-25268) | 20560.1 (14099-27146.1) |
| Spain | 6.9 (4.2-9.8) | 13.9 (8.7-20) | 22.6 (14.3-32.4) | 36.3 (22.7-51.3) | 64.3 (40.4-92.2) | 104.6 (65.6-149.2) | 152.8 (96-215.5) | 215.8 (136.9-302.6) | 280.2 (178.3-395.6) | 369.7 (232.5-518.4) | 539.3 (340.9-760.5) | 792.3 (491.1-1137.2) | 1267.4 (794.8-1801.2) | 2060 (1275-2974.9) | 3269.8 (1932.2-4781) |
| Sri Lanka | 143.4 (76.3-235.7) | 209.8 (109.6-347.1) | 325 (169.6-570) | 505.9 (253.4-868) | 836.8 (418.1-1415.2) | 1325.4 (667.1-2297.7) | 1791.5 (880.8-2969.9) | 2631.6 (1309.3-4472.6) | 3888.5 (2019.4-6421.9) | 6089.2 (3189.6-9985.8) | 7868.9 (4091.3-13110.7) | 11504.1 (6031.2-18763.1) | 14964.5 (7586.4-25070.5) | 18310 (9022-30388.5) | 28682.4 (13899.9-48278.6) |
| Sudan | 471.1 (286.4-698.8) | 715.6 (400.3-1062.1) | 1115.2 (649.5-1625.1) | 1873.6 (1159.8-2721.7) | 2986.9 (1951.6-4312.8) | 4738.7 (3264.5-6643.9) | 6882.2 (4744.9-9485) | 9700.4 (6899.5-13566.3) | 13361.6 (9616.8-18201.2) | 17882.8 (13596-24062.7) | 22752.5 (17143.1-29597.3) | 26925.6 (19768.2-34866.9) | 32802.4 (24524.4-41345) | 41530.5 (30162.3-53299) | 52265.9 (35384.6-67640.9) |
| Suriname | 107.4 (47.5-180.6) | 219.5 (100.4-363.6) | 381 (170.7-632.6) | 632.2 (299.6-1035.7) | 1110.9 (526.8-1794.5) | 1793.2 (850.4-2899.8) | 2702.2 (1217.8-4391.3) | 3446.6 (1567.8-5664.2) | 4499.2 (2035.2-7461.7) | 4969.6 (2304.6-8201.6) | 5952.5 (2840.5-9791.3) | 7338.7 (3526.4-11924.9) | 9356.7 (4311.1-15533.1) | 10674 (4900.4-18406.7) | 10768.8 (4637.7-19242.4) |
| Sweden | 1.9 (0.5-3.8) | 2.7 (0.7-5.3) | 4.5 (1.3-8.5) | 9.1 (2.7-17.4) | 17.7 (5.2-33.6) | 33.9 (9.9-63.9) | 54.9 (16-106.1) | 88.8 (26-167.8) | 136.3 (39.5-259) | 191.6 (55.3-358.7) | 282.8 (81.1-531.9) | 466.2 (136.7-877.4) | 806 (234.9-1525) | 1370.9 (400.6-2607.5) | 1690.4 (488.6-3306.8) |
| Switzerland | 4.4 (2.8-6.3) | 6.8 (4.3-9.8) | 11.1 (7.1-15.8) | 22.1 (13.9-31.6) | 37.1 (23.6-53.4) | 62.3 (39.5-88.1) | 97.4 (61-138.7) | 149.8 (94-213.8) | 223.7 (143.2-313.9) | 332.7 (211.2-465.7) | 508.3 (323.5-708.7) | 832.9 (513.9-1202.4) | 1542.3 (943.1-2203.8) | 2602.4 (1564.6-3792.7) | 3854.3 (2242.3-5566.6) |
| Syrian Arab Republic | 312.7 (207.8-463.1) | 445.3 (299.6-628.2) | 640 (422.7-916) | 1041.6 (672.3-1510.1) | 1837.1 (1210.9-2675.4) | 2910.1 (1889.6-4230.3) | 4185.2 (2701.6-6059.7) | 6263.6 (4140-8893.6) | 8125.2 (5457.7-11491.4) | 10327.9 (6954.6-14499) | 13772.9 (9355.3-19165.7) | 19464.7 (13764.4-26164.4) | 32036.6 (22931.2-42594.4) | 42549.7 (31046.9-57109.8) | 53736.9 (38325.4-70687.8) |
| Taiwan (Province of China) | 31.5 (24.1-40) | 53.7 (40.9-69) | 96 (74.1-121.5) | 166.8 (128.8-213.8) | 253.1 (198.1-319.4) | 363.3 (276.6-456) | 459.1 (359.2-578.4) | 590.2 (459.5-745) | 767.5 (598.6-969.4) | 1002.2 (778.1-1256.2) | 1555.7 (1202.1-1963.9) | 2125.1 (1623.7-2706.9) | 3099.9 (2338.9-3929.9) | 4294.2 (3174.8-5560.7) | 4143.3 (2856.8-5404) |
| Tajikistan | 178.9 (130.5-241.7) | 347.8 (245.8-468.2) | 535 (380.5-710.4) | 918.9 (661.3-1216.4) | 1538 (1119-2026.5) | 2585.8 (1908-3375.3) | 4293 (3223.2-5458.8) | 6998 (5296.7-8805.9) | 11262.4 (8642.5-13988) | 16844.2 (13054.7-20980.5) | 22910.8 (17757.3-28438.2) | 29829.9 (22675.4-37227.1) | 31845.4 (23540.1-39856.7) | 42000.4 (30829.6-53257.7) | 54646.9 (38354.7-70384.7) |
| Thailand | 168 (116.4-242.5) | 307.2 (211.4-435.1) | 461.9 (317-651.1) | 648.2 (435.9-937.5) | 827.9 (557.8-1175.6) | 1107.8 (745.6-1588) | 1334.2 (893-1887.4) | 1742.2 (1210-2451.2) | 2305.8 (1589-3174.7) | 3150.4 (2225.3-4224.1) | 4282.3 (3052.1-5886.2) | 5685.7 (4028.2-7588.3) | 6359.8 (4487.1-8639.8) | 8571.4 (6015.3-11753.6) | 10648.8 (6969.9-14622.8) |
| Timor-Leste | 358.4 (202.4-554) | 518.1 (277.3-800.5) | 822.5 (456.3-1247.8) | 1444.1 (814.1-2107) | 2404.3 (1415.9-3545.4) | 3976.5 (2456.4-5633.1) | 5913.6 (3595.8-8132) | 8265.3 (5038.8-11448) | 11232.3 (6770.5-15324.6) | 15189.7 (9440.5-20745.3) | 19473 (12201.7-26250.6) | 24031.4 (15252.3-32258.7) | 28767.8 (17880.4-38776.8) | 34919.1 (21990-47557.8) | 43487.1 (26733.4-58663.8) |
| Togo | 221.7 (147.5-317.7) | 411.7 (276.4-585) | 650.8 (447.1-909.1) | 1341.2 (899-1860.9) | 2371.8 (1595.9-3271.8) | 4212.9 (2886.4-5861.9) | 6092.9 (4205.7-8372.1) | 9264.4 (6600.6-12807.4) | 13197 (9466.4-17540.5) | 16801.9 (12421.4-22128.2) | 21400.6 (15864.3-27384.1) | 24953.4 (19263.8-31886.7) | 27889.9 (21383.8-35225.4) | 32365.2 (25089-41743.5) | 41144.8 (30101.9-52564.8) |
| Tokelau | 55.4 (0.5-133.8) | 94.3 (0.8-234.5) | 117.2 (1-315.1) | 188.5 (1.5-502.8) | 294.6 (2.3-778.4) | 410.4 (3.4-1087.1) | 553.3 (4.4-1407.5) | 719.7 (6.1-1833.5) | 924 (7.4-2327.5) | 1119.5 (9.8-2843.4) | 1423.8 (12.8-3506.1) | 1781 (15.4-4510.3) | 2222.3 (19.5-5503.9) | 2985.8 (25.1-7376) | 4239.7 (35.9-10285.4) |
| Tonga | 185.8 (102.6-306.2) | 347.4 (199.8-566.9) | 527.5 (306.5-863.5) | 958.8 (543.4-1525.4) | 1458.9 (840.8-2316.8) | 2124.2 (1194.4-3248.3) | 2889 (1654.6-4409.7) | 3790.4 (2159.8-5597.8) | 4876 (2940-7082.5) | 6301.5 (3761.2-8982.4) | 8142.1 (4791.6-11626.5) | 9893.6 (6212.4-13929.5) | 12714.2 (7658-18249) | 15765.6 (9416.8-22631) | 21791.4 (13591.1-31056.5) |
| Trinidad and Tobago | 104.1 (36.2-196) | 162 (58.3-301.1) | 329.8 (117.3-613.6) | 500 (179.2-919.2) | 863.1 (299.7-1597.5) | 1254 (434.9-2301.7) | 1725.4 (616.7-3229.8) | 2348.4 (817.6-4341.6) | 2829.9 (1003.7-5223.5) | 4288.7 (1532.4-7951.4) | 5324.6 (1905.2-9747.2) | 6859.7 (2382-12446.8) | 7910.4 (2758-14390.1) | 10678.4 (3713.7-19097.3) | 14870 (5236.3-26740.3) |
| Tunisia | 99.5 (61.7-153) | 172.7 (105.9-260.1) | 260.7 (162.2-388.6) | 456.1 (281.2-686.6) | 727.2 (446.8-1084.1) | 1102.1 (680.8-1668.6) | 1757.9 (1110-2669.4) | 2590.4 (1594.8-3828.4) | 3737.1 (2361.7-5501) | 5333.9 (3477.2-7696.9) | 7358.9 (4768.6-10528.5) | 11084.4 (7221.7-15847) | 14791.3 (9272.7-20660.5) | 19690.4 (12181.8-27142.7) | 22595.3 (13272-32732.8) |
| Turkey | 68.6 (50.4-93.4) | 108.5 (78.6-143.8) | 182.4 (126.6-237.7) | 317.6 (223.4-413.4) | 564.7 (394-731.5) | 953.1 (670.9-1260.7) | 1409.4 (977.3-1904.6) | 2118.3 (1484.5-2788.2) | 2991 (2130.6-3792) | 4169.9 (3000.9-5324) | 6434.5 (4719.6-8145.6) | 9032.3 (6741.4-11428.3) | 13762.6 (10417.8-17152.8) | 17525.5 (12959.8-22267.2) | 19980.9 (13619.4-26419.2) |
| Turkmenistan | 168 (99-264.3) | 271.9 (158-429.3) | 476.1 (276.7-738.9) | 878.6 (516.8-1380.1) | 1523 (884-2397.8) | 2377.3 (1369.8-3731.2) | 3772.1 (2201.4-5949.2) | 6007.3 (3506.5-9310) | 8163.4 (4784.4-12617.6) | 12127.3 (7161-18633.6) | 14404 (8498.5-22003.6) | 18855.1 (11070.9-28840.8) | 18275.5 (10742-27927.9) | 21633.5 (12459.6-33252.7) | 22867.1 (12500.7-36613.8) |
| Tuvalu | 181.7 (98.7-289.5) | 328.6 (175.5-543.4) | 533.5 (288.4-860.6) | 917.6 (507.2-1535.1) | 1324.9 (735.9-2128.9) | 1770.8 (984.1-2853.8) | 2227.9 (1265.8-3643.3) | 2850.7 (1608.1-4529.4) | 3412.5 (1962.4-5339.7) | 4206.6 (2459.7-6525.4) | 5180.8 (3032.7-7846.3) | 6196.4 (3550-9519.7) | 8031.7 (4664.2-12628) | 9510.2 (5530.8-14766.6) | 13328.1 (7625.7-21408.4) |
| Uganda | 178.2 (119.1-248.5) | 317.1 (211.6-450.4) | 474.1 (314.2-651.5) | 904.5 (599.2-1254.2) | 1431.6 (979-1999) | 2340.6 (1593.7-3163.1) | 3690.7 (2601-4937.3) | 5824.1 (4205.7-7668) | 8248.2 (5926.6-10835) | 11094.3 (8289.2-14312) | 14119.6 (10604.7-18095.7) | 16389.9 (12468.1-21367.5) | 17673.9 (12961.3-23459.4) | 21250.1 (15021-28704) | 21169.7 (13959.6-30147.4) |
| Ukraine | 98 (52-152.2) | 197.9 (110.6-309) | 311.7 (166.3-500.1) | 524.8 (276-875.1) | 844 (441.6-1409.2) | 1376.3 (723-2251.6) | 2250 (1205.1-3700.7) | 3669.8 (1993.9-5939.8) | 5305.5 (2968.8-8393.9) | 7624.3 (4360.7-11733.5) | 10801.1 (6227.1-16957.1) | 14470.8 (8374.3-22790.3) | 20235.5 (11541.9-32152.5) | 28945.8 (16282.5-45840.6) | 31608.5 (17357.7-52177.6) |
| United Arab Emirates | 157.6 (105.7-215.1) | 143.1 (89.1-204.5) | 162.9 (106.2-225.7) | 307.7 (206.7-412.1) | 540 (360-738.6) | 952.9 (635-1293.1) | 1598.1 (1111.3-2131.7) | 2903.7 (1999-3906.8) | 3193.7 (2260.8-4282.6) | 15709.1 (11271.2-20937.8) | 19059.7 (13633.1-24382.4) | 17392.1 (12927.6-22060.8) | 20114.3 (14700-25690.8) | 24196.3 (17374.7-32203.1) | 38491.3 (27142-51589.8) |
| United Kingdom | 7.7 (5.2-10.8) | 14.3 (9.4-20) | 29.4 (19.3-41.3) | 54.3 (35.2-76.4) | 94.6 (60.8-132.8) | 153.9 (98.7-216.7) | 211.1 (132.9-295.6) | 299.3 (190.6-423) | 391.7 (249.2-553.1) | 537 (344.4-761.8) | 776.5 (495-1093.3) | 1130.3 (717.6-1562.1) | 1737.4 (1088.6-2411.8) | 2587.4 (1616.3-3619) | 4271.8 (2631.3-5988.3) |
| United Republic of Tanzania | 220.7 (143.8-308.8) | 379.4 (258.3-526) | 552.2 (383.6-773.8) | 955.5 (658.8-1323.7) | 1466.4 (987.3-2059) | 2428.1 (1718.5-3353.5) | 3886.6 (2725.9-5146.1) | 6509.8 (4604.2-8816.6) | 9721.7 (7094-12840.1) | 13333.7 (9971.1-17537.3) | 16900.6 (12808.7-21281.1) | 19067 (14831.6-24635) | 20517.7 (15250.6-26597.3) | 24700.2 (17982.6-32107.1) | 26008.9 (17499.5-34574.4) |
| United States of America | 8.8 (4.3-14.2) | 16.6 (8.2-26.9) | 29.6 (14.6-47.8) | 54 (26.1-86.7) | 93 (45.6-150.8) | 150.7 (73-242) | 219.7 (106.4-353.6) | 292 (139.8-469.1) | 351.6 (168.2-562.5) | 450.9 (215.4-724.3) | 608.4 (292.9-977.9) | 847.9 (406.3-1354.9) | 1268.7 (594.6-2071.7) | 2007.3 (930.7-3330.4) | 3372.2 (1530.3-5683.5) |
| United States Virgin Islands | 27.2 (10.7-52.4) | 39.6 (16.5-69.6) | 62.2 (26.3-114.8) | 61.7 (26.9-106.5) | 181.6 (76.5-317.2) | 139.3 (60.4-240.8) | 294.4 (127.5-504.4) | 448.7 (197.4-761.2) | 426.6 (185.3-730.8) | 717.1 (315.7-1222.7) | 872.6 (387.9-1466.7) | 1163.3 (513.9-1943.7) | 1737.8 (787.3-2895.8) | 3161.4 (1408.1-5256.3) | 7919.1 (3590.6-13155.7) |
| Uruguay | 18.5 (7.1-33) | 29.6 (11.5-52) | 49.9 (19.1-91.3) | 102.7 (39-179.6) | 164.1 (62.6-291.2) | 253.6 (100.4-456.8) | 376.4 (148.3-670.3) | 521.9 (206.1-918.8) | 716.1 (281.7-1264.1) | 972.6 (375.8-1688.9) | 1264.9 (497.8-2175) | 1665.4 (638-2854.1) | 2291.4 (864.7-3926.6) | 3383.4 (1238.1-5830.8) | 4793.8 (1776.2-8351.6) |
| Uzbekistan | 239.4 (174.5-317.7) | 393.4 (288.9-501.6) | 582.9 (420.3-753.1) | 940.7 (680.6-1228.3) | 1620.3 (1175-2116.6) | 2810.7 (2013.4-3677.1) | 4478.9 (3228.5-5835.1) | 7979.4 (5825.8-10346.4) | 10572.5 (7622.2-13796.2) | 16586 (12051.9-21428) | 22979.6 (16693.4-29566.2) | 29362 (21388.4-37629.7) | 30741.7 (22037.2-39670.2) | 36004.7 (25197.2-46572.9) | 51386 (35708.4-67064.9) |
| Vanuatu | 1035.6 (700.2-1386.9) | 1827.1 (1232.5-2394.8) | 2933.9 (2142.3-3804.5) | 5076.4 (3634.8-6397.2) | 7288.8 (5239-9229.9) | 9766 (7394.5-12359) | 12292.5 (9449.6-15335.3) | 15726.5 (12189.3-19528) | 18981.3 (15138.8-22990) | 23321.6 (18857-28154.1) | 28035.3 (22425.8-33826.8) | 33326.1 (26631.4-40210.1) | 41677.6 (33482.5-49623.1) | 49167.6 (37415.5-59846.1) | 67844.6 (45979.8-85169.6) |
| Venezuela (Bolivarian Republic of) | 76 (41.2-120.5) | 124 (69.4-195) | 205 (114.2-329) | 353.9 (190-586.1) | 567.1 (302.3-908.4) | 887.3 (480.9-1465.8) | 1281.1 (691.3-2072.7) | 1726.8 (943.7-2750.5) | 2192.9 (1216.3-3476.7) | 2971.2 (1633.7-4622.5) | 3841 (2146.8-6001.8) | 4945.9 (2731.2-7583.8) | 6204 (3413.7-9593.4) | 7249.4 (3927-11219.6) | 7258.6 (3861.4-11647.9) |
| Viet Nam | 100.8 (65.6-146.3) | 189 (121.8-272.4) | 373.5 (240.4-541.4) | 730.8 (466.2-1086.3) | 1280.2 (822.1-1833.8) | 2145.6 (1390.2-3032.6) | 3165 (2101.4-4242.8) | 4431.1 (3003.8-5954.9) | 6451.4 (4458.4-8508.6) | 9403.6 (6690-12185.2) | 12995.7 (9081.2-16761.5) | 16155.5 (11390.8-20514.4) | 19142.4 (13625.2-24412.8) | 23535.9 (16221-30747.6) | 27822.5 (18525.8-37112.7) |
| Yemen | 262.3 (158.5-412.4) | 453.7 (275.6-681) | 869.8 (553.3-1280.9) | 1755.5 (1147.9-2544.4) | 3041.2 (2010.8-4548.6) | 4837.5 (3217.1-6904.6) | 6971.5 (4741.3-10077.6) | 10011.7 (7010.6-13866.2) | 13635 (9547.6-18776) | 17870.9 (12366.3-24119.1) | 22702.6 (15922-30636.2) | 27580.5 (19533.4-36528.2) | 32972.1 (21955.8-43963.9) | 40640.7 (27781.8-54386.7) | 50541.7 (32995.5-69115.4) |
| Zambia | 193.8 (119.3-293.5) | 369.5 (238.9-528.9) | 584.2 (375.1-857.6) | 1197 (767.6-1732.9) | 2035.8 (1338.1-3011.1) | 3241.6 (2143.9-4572.7) | 5168.7 (3459.7-7159.6) | 8103.4 (5728.4-10646.9) | 11136 (7965.4-14229.5) | 15353.2 (10948.8-19473.3) | 19946.7 (14933.4-25303.4) | 23297.9 (17474-29257.7) | 26262.8 (19293.8-33634.9) | 25487 (18156.5-33344.3) | 15564.8 (9890.9-22467.1) |
| Zimbabwe | 209.3 (139.6-303.7) | 307.3 (200.7-452.8) | 531.6 (335.6-813.2) | 1006.9 (668.8-1524) | 1999 (1321.3-2910.7) | 3917.7 (2753.9-5627.6) | 5847.7 (4059.7-8085.2) | 8245.1 (6058.1-11307) | 10939.5 (8353.5-14271.6) | 14352.9 (11011.6-18066.2) | 18916 (14749-24023.5) | 23164.1 (18061.2-28444.4) | 27215 (21069.2-33473.8) | 21822.1 (15848.8-28747.4) | 13241.7 (8710.2-18351.9) |

Supplementary Table 9 DALYs of CVDs Attributable to PM2.5 Pollution by Age Distribution, Gender, 5 SDI Regions, and CVDs Subtypes, 2021

|  | 25-29 years | 30-34 years | 35-39 years | 40-44 years | 45-49 years | 50-54 years | 55-59 years | 60-64 years | 65-69 years | 70-74 years | 75-79 years | 80-84 years | 85-89 years | 90-94 years | 95+ years |
| --- | --- | --- | --- | --- | --- | --- | --- | --- | --- | --- | --- | --- | --- | --- | --- |
| Male | 227.6 (187.4-267.2) | 417.6 (339.9-499.8) | 638.6 (520.8-770.1) | 1124.8 (906.3-1352.9) | 1610 (1302.4-1933.9) | 2449.2 (1995-2949) | 3375.8 (2737.9-4060.9) | 4593.6 (3732.2-5539.2) | 5970.1 (4756.5-7221.1) | 7591.8 (6013.5-9157.5) | 9360 (7476.6-11343.9) | 11331.6 (8948.6-13905.1) | 15014.7 (11920.7-18394) | 16798.4 (13090-20674.7) | 13932.9 (10544.2-17231.1) |
| Female | 139.1 (113.1-166.1) | 207.7 (169.9-246) | 310.3 (255.5-369.1) | 529.2 (434.3-635) | 829.6 (682.5-985.1) | 1239 (1025.1-1492.6) | 1927.8 (1582.6-2302.5) | 2679.4 (2196.5-3153.9) | 3844.7 (3155.9-4540.2) | 5141.9 (4182.6-6117.3) | 6833.5 (5455.8-8205.4) | 8572.6 (6777.7-10430.7) | 9763.9 (7429.9-12077.5) | 11501.5 (8521.6-14472.4) | 13056.7 (9296.3-16802.8) |
| Cardiovascular diseases | 183.8 (151.8-216.9) | 313.8 (257-371.2) | 476 (392.9-564.8) | 829.4 (684.9-985) | 1221.6 (1004.7-1445.5) | 1842.8 (1526.9-2175) | 2640.3 (2160.7-3102.6) | 3609.7 (2963-4260.2) | 4860.5 (3963.9-5762.9) | 6289.1 (5094.1-7526.6) | 7978.8 (6444.4-9627.3) | 9727.2 (7732.6-11841.9) | 11745.2 (9088-14392.6) | 13227.3 (10068-16330.7) | 13299.8 (9586.7-16919.6) |
| Ischemic heart disease | 109.9 (85.3-134.8) | 195.2 (151.1-242.7) | 294.7 (228.4-363.8) | 515.5 (397.8-636.3) | 733.2 (567.7-901.1) | 1050.6 (809.7-1297.1) | 1492.8 (1142.2-1856.2) | 1978.5 (1515.6-2433) | 2534.9 (1928.6-3149.5) | 3193.2 (2402.5-3968.6) | 4004.7 (3006-5024.9) | 5189.2 (3795-6521.7) | 6454.2 (4703.1-8138.1) | 7959.7 (5684.2-10177) | 8534.6 (5805.2-11124.1) |
| stroke | 73.9 (58.2-90.4) | 118.6 (93.4-144.4) | 181.4 (142.1-221.3) | 314 (244.3-381) | 488.4 (379.7-599.4) | 792.2 (610.9-979.3) | 1147.5 (891.1-1411.2) | 1631.1 (1273.9-2003.4) | 2325.6 (1796.6-2871.1) | 3095.9 (2383.7-3854) | 3974.1 (3076.5-4906.1) | 4538 (3473.2-5700) | 5291 (4011.2-6760.2) | 5267.6 (3903.7-6769.5) | 4765.2 (3372.5-6270.8) |
| Intracerebral hemorrhage | 45.4 (34.6-56.2) | 76.4 (58.6-94.2) | 119.6 (93-147.1) | 214.8 (165.2-265.7) | 338.1 (261.3-419.2) | 542.5 (420.9-673.4) | 764.7 (590.7-946.9) | 967.9 (742.2-1191.9) | 1312.2 (997.7-1626) | 1419.3 (1083.5-1774.7) | 1734.1 (1332.6-2191.8) | 1584.5 (1183.7-2034.2) | 1795.2 (1339-2327.7) | 1648.5 (1226.6-2156.7) | 1317.6 (926.5-1735.3) |
| Ischemic stroke | 15.7 (11.7-20.2) | 23.4 (17.6-29.8) | 34.4 (26.3-43.5) | 55.7 (43.2-69.4) | 98.6 (75.8-122.9) | 178.9 (137.9-222.1) | 290.5 (226-361.3) | 576.9 (455.4-712.4) | 906.2 (708.1-1119) | 1566.9 (1229.8-1935.2) | 2115.4 (1641-2617.6) | 2829.3 (2171.5-3539.9) | 3356.6 (2558.4-4282.5) | 3480.4 (2592-4504.8) | 3321.1 (2345.1-4408.5) |
| Subarachnoid hemorrhage | 12.8 (9.2-17.7) | 18.8 (13.4-26.2) | 27.4 (20.1-37.2) | 43.4 (31.3-59.4) | 51.7 (38-70.6) | 70.8 (51.6-97.4) | 92.3 (69.2-121.4) | 86.4 (64-115.4) | 107.2 (80.3-139.9) | 109.6 (80.9-142.9) | 124.6 (92.3-164.8) | 124.1 (90-162.9) | 139.3 (99-181.7) | 138.7 (96.7-182.3) | 126.5 (88.1-171.5) |
| High SDI | 37.4 (27.6-48.3) | 72 (53.8-95.2) | 122.1 (91.3-158.6) | 204.2 (150.5-267.1) | 282.8 (212.8-356) | 355.3 (270.1-448.6) | 450 (339.1-572.3) | 576.7 (429.3-739.2) | 781.2 (589.5-997.8) | 977.1 (727.1-1258) | 1318.7 (973.5-1698) | 1890.2 (1379.6-2483.5) | 2847.9 (2022.2-3779.7) | 3952.9 (2637.9-5388.9) | 5027 (3082.6-7074.1) |
| High-middle SDI | 93.2 (75-117.1) | 171.1 (135.1-217.3) | 276.3 (212.4-352) | 463.9 (360.7-594.1) | 678 (526.9-885.2) | 1072 (825.4-1384.4) | 1580.4 (1215.1-2024.5) | 2337.8 (1819.5-2958.3) | 3448 (2679.7-4372.9) | 5044.7 (3956.7-6414.3) | 7222.2 (5653.9-9085.7) | 10229.5 (7910.3-13020.7) | 14055.9 (10776.8-17666.8) | 17400 (13045.8-21967.1) | 20064.9 (14511.1-25620.1) |
| Middle SDI | 178.2 (142.8-217.6) | 301.2 (242.1-366.2) | 470.8 (375.4-570.1) | 802.9 (639.8-986.2) | 1198.5 (957.4-1474.8) | 1840.1 (1462.4-2285.8) | 2681.5 (2137.7-3304.1) | 3881.1 (3091.4-4745.9) | 5484 (4402.8-6787.1) | 7770.5 (6135.3-9704) | 10279.5 (8103-12675.5) | 13698.7 (10564.8-16952.7) | 18605.9 (14167.4-22841.4) | 22560.3 (17080.9-28085.9) | 23148.9 (16887.7-29343.6) |
| Low-middle SDI | 278 (225.3-325.9) | 509.3 (419.5-602.6) | 762.7 (629.2-901.5) | 1385.2 (1143.8-1648.4) | 2124.3 (1726.2-2506.8) | 3445.2 (2860.2-4052.8) | 5279.8 (4388.2-6265.1) | 6984 (5799.2-8136.2) | 9387.6 (7778.7-10890.8) | 11882.3 (9871.1-13844.4) | 13995.6 (11596.2-16219.7) | 16338.6 (13477.2-18943.5) | 17764.7 (14559-20767.6) | 21050.3 (16764.3-24760.8) | 22190.9 (17275.6-26566.4) |
| Low SDI | 229 (188.1-272.6) | 418.8 (342.5-494.1) | 649.8 (535.1-763.1) | 1243.7 (1013.9-1476) | 2094.7 (1722.5-2477.3) | 3403.6 (2784.9-3990.2) | 5233.5 (4356.4-6133.8) | 7621 (6340.6-8926.5) | 10491.3 (8734.9-12227.3) | 13741.4 (11444.9-15870.3) | 16234.8 (13670-18764.1) | 18596.3 (15657.8-21880) | 20599.1 (17060.5-24060.3) | 25064.3 (20206.3-29912.7) | 28627.9 (21955.4-34441.5) |

Supplementary Table 10 Deaths of CVDs Attributable to PM2.5 Pollution by Age Distribution, Gender, 5 SDI Regions, and CVDs Subtypes, 2021

|  | 25-29 years | 30-34 years | 35-39 years | 40-44 years | 45-49 years | 50-54 years | 55-59 years | 60-64 years | 65-69 years | 70-74 years | 75-79 years | 80-84 years | 85-89 years | 90-94 years | 95+ years |
| --- | --- | --- | --- | --- | --- | --- | --- | --- | --- | --- | --- | --- | --- | --- | --- |
| Male | 3.4 (2.8-4) | 6.9 (5.6-8.3) | 11.6 (9.4-13.9) | 22.7 (18.2-27.4) | 36 (29-43.5) | 61.7 (49.8-74.4) | 96.7 (77.8-117.1) | 152.9 (123.3-183.4) | 234.7 (185.8-284.9) | 362 (287-437.8) | 557.3 (440.5-678.7) | 867.5 (683.4-1063.3) | 1468.9 (1166.4-1798.7) | 1901.4 (1480-2342.8) | 1661.3 (1249.6-2057.9) |
| Female | 1.9 (1.5-2.3) | 3.2 (2.6-3.8) | 5.3 (4.3-6.4) | 10.2 (8.3-12.3) | 17.9 (14.7-21.3) | 30.2 (24.9-36.5) | 54 (44.2-64.6) | 87.5 (71.8-103.9) | 149.3 (122-177.3) | 243.2 (196.9-291) | 407.4 (323.1-489.3) | 658.3 (518.1-802.4) | 945.7 (717-1174.1) | 1291.6 (950.3-1626.3) | 1565.2 (1099.6-2023.1) |
| Cardiovascular diseases | 2.7 (2.2-3.2) | 5.1 (4.1-6) | 8.5 (6.9-10.1) | 16.5 (13.5-19.6) | 27 (22.3-32.1) | 45.9 (37.7-54.4) | 75 (61.1-88.5) | 119.3 (96.8-141) | 190.1 (154.2-226.1) | 298.8 (242.4-358.1) | 475.4 (382.4-574.7) | 745.9 (589.4-910.5) | 1143.1 (882.2-1401.1) | 1490.3 (1129.2-1838.7) | 1591.9 (1139.5-2029.1) |
| Ischemic heart disease | 1.7 (1.3-2.1) | 3.3 (2.6-4.2) | 5.5 (4.3-6.8) | 10.6 (8.2-13.1) | 16.8 (13.1-20.6) | 27.1 (20.9-33.5) | 43.8 (33.5-54.4) | 67.1 (51.5-82.5) | 101.6 (77.4-126.4) | 155.1 (116.2-192.7) | 244.4 (182.5-307.5) | 407.3 (297.5-513) | 639.5 (464.8-806.8) | 909.7 (647.1-1163.1) | 1035 (699.1-1349.8) |
| stroke | 0.9 (0.7-1.1) | 1.7 (1.4-2.1) | 2.9 (2.3-3.6) | 5.8 (4.6-7.2) | 10.2 (8-12.5) | 18.8 (14.5-23.3) | 31.2 (24.3-38.4) | 52.1 (40.7-64.1) | 88.5 (68.5-109.6) | 143.6 (110.6-178.7) | 230.9 (178.7-288.9) | 338.5 (259.3-432.2) | 503.6 (380.1-650.2) | 580.6 (428.3-749) | 556.9 (386.9-738.1) |
| Intracerebral hemorrhage | 0.7 (0.5-0.8) | 1.2 (0.9-1.5) | 2.1 (1.6-2.6) | 4.3 (3.3-5.3) | 7.5 (5.8-9.4) | 13.7 (10.6-17) | 22.1 (17-27.2) | 32.6 (24.9-40.1) | 52.9 (40-65.5) | 69.6 (53.2-86.9) | 106.7 (82-134.8) | 124.8 (93.2-160.2) | 178.3 (132.9-231.6) | 187.8 (139-245) | 158.3 (110.6-209.3) |
| Ischemic stroke | 0.1 (0.1-0.1) | 0.2 (0.2-0.3) | 0.4 (0.3-0.5) | 0.7 (0.6-0.9) | 1.6 (1.2-2) | 3.5 (2.7-4.4) | 6.7 (5.1-8.4) | 16.8 (13.1-20.9) | 31.6 (24.4-39.2) | 69.1 (53.6-86.3) | 117.1 (90.6-145.8) | 204.6 (156.4-261.5) | 312.2 (236.5-400.3) | 377.7 (277-491.4) | 384.2 (266.8-513.8) |
| Subarachnoid hemorrhage | 0.2 (0.1-0.3) | 0.3 (0.2-0.4) | 0.5 (0.3-0.6) | 0.8 (0.6-1.1) | 1.1 (0.8-1.5) | 1.7 (1.2-2.3) | 2.5 (1.8-3.3) | 2.7 (2-3.6) | 4 (2.9-5.3) | 5 (3.6-6.6) | 7.1 (5.2-9.5) | 9.1 (6.6-12) | 13.1 (9.4-17.2) | 15 (10.4-19.9) | 14.4 (9.7-19.7) |
| High SDI | 0.5 (0.4-0.7) | 1.1 (0.8-1.5) | 2.1 (1.5-2.8) | 3.9 (2.9-5.2) | 6.1 (4.5-7.7) | 8.5 (6.4-11) | 12.3 (9.1-15.7) | 18.1 (13.3-23.2) | 28.8 (21.6-36.7) | 43.8 (32.2-56.5) | 74.7 (54.6-96.3) | 139.1 (100.5-182.2) | 268.9 (189.6-356.4) | 434.7 (287.2-597.8) | 598.7 (363.4-848.8) |
| High-middle SDI | 1.3 (1-1.6) | 2.7 (2.1-3.4) | 4.8 (3.6-6.1) | 9 (7-11.5) | 14.7 (11.4-19.3) | 26.1 (20.1-33.8) | 43.8 (33.7-56.2) | 75.8 (58.6-96.8) | 132 (102.2-169.3) | 235.8 (184.7-300.4) | 426.4 (332.6-540.4) | 786.2 (605.7-998.1) | 1374.9 (1048.8-1728.4) | 1972.3 (1479.2-2489.9) | 2411.6 (1730.3-3085.2) |
| Middle SDI | 2.6 (2-3.2) | 4.8 (3.8-5.9) | 8.4 (6.6-10.2) | 15.9 (12.6-19.7) | 26.4 (20.9-32.6) | 45.6 (36.2-57) | 75.8 (60-93.8) | 127.8 (101.3-157.6) | 213.6 (171-265.5) | 368.3 (291.7-463.4) | 611.3 (477.9-760.5) | 1049.8 (801.1-1303.7) | 1816.7 (1382.5-2232.7) | 2548.7 (1930-3179.2) | 2776.6 (2013.8-3542.4) |
| Low-middle SDI | 4.1 (3.3-4.8) | 8.4 (6.9-10) | 13.7 (11.2-16.3) | 27.8 (22.8-33.2) | 47.5 (38.5-56.3) | 86.9 (72.1-102.4) | 152.3 (126.5-181.1) | 233.7 (193.7-272.3) | 373.6 (308.7-433.2) | 573.7 (476.8-667.5) | 846 (700-978.7) | 1263.9 (1038.5-1463.8) | 1731.2 (1413.7-2029.9) | 2378.1 (1887.3-2806.3) | 2649.5 (2051.9-3183.6) |
| Low SDI | 3.3 (2.7-3.9) | 6.7 (5.5-7.9) | 11.5 (9.4-13.6) | 24.7 (20-29.2) | 46.4 (38-55.3) | 85.2 (69.6-100.1) | 149.9 (124.6-176.8) | 254.2 (212.4-298.8) | 416.5 (348-486.3) | 661.7 (551.9-765.6) | 976.1 (823.3-1124.2) | 1426.7 (1196.6-1680.3) | 1987.6 (1636.2-2324.6) | 2803.8 (2243.1-3356.7) | 3350.2 (2557.5-4053) |
