# Supplementary figures and images for: Global, Regional, and National Burden of Cardiovascular Diseases Associated with Particulate Matter Pollution: A Systematic Analysis of Deaths and Disability-Adjusted Life Years with Projections to 2030
Source: Rev Cardiovasc Med. 2025 Apr 17;26(4):27056. doi: 10.31083/RCM27056 (PMC12059744; doi:10.31083/RCM27056)

A

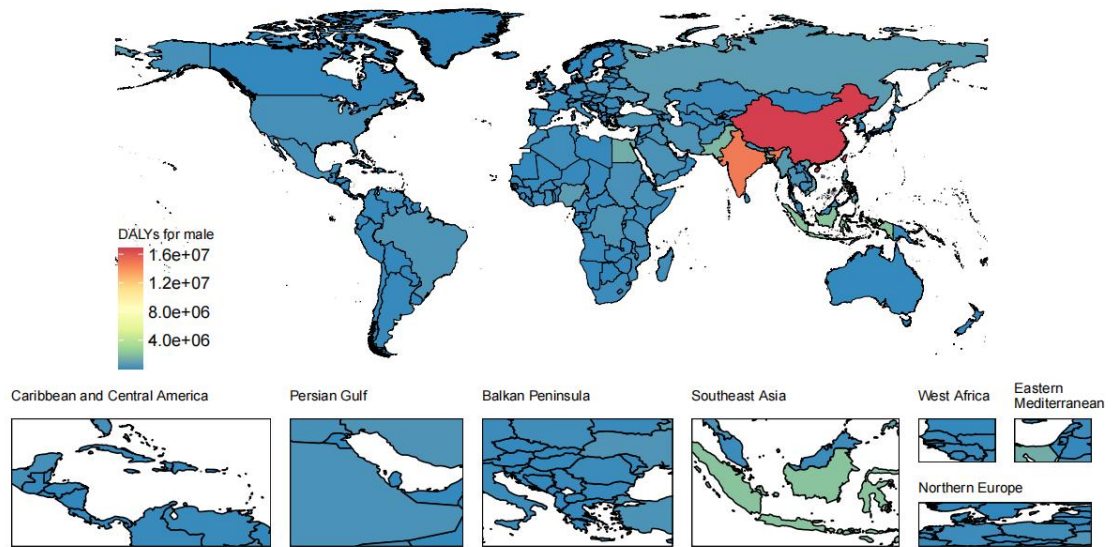

B

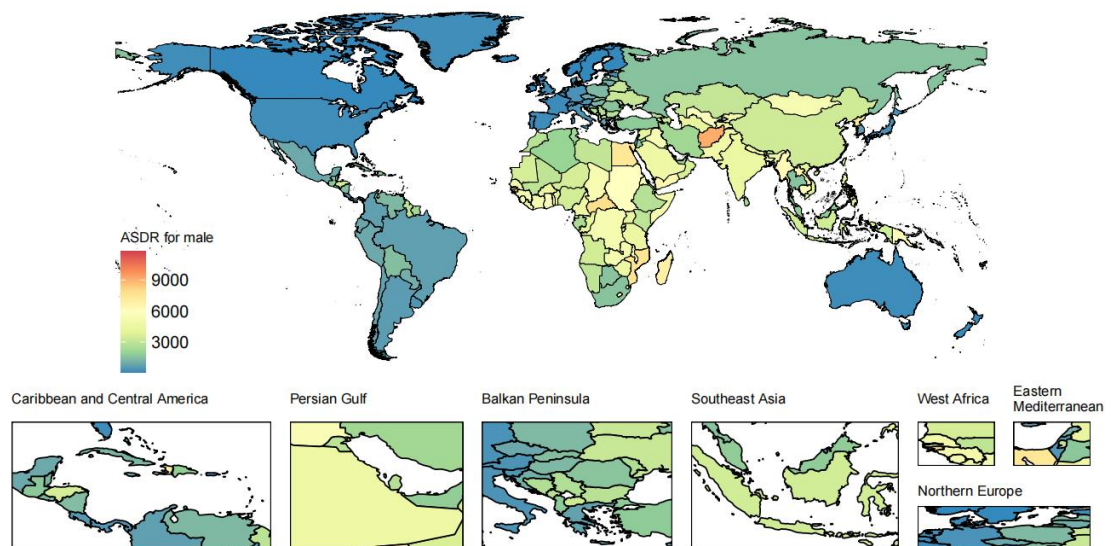

C

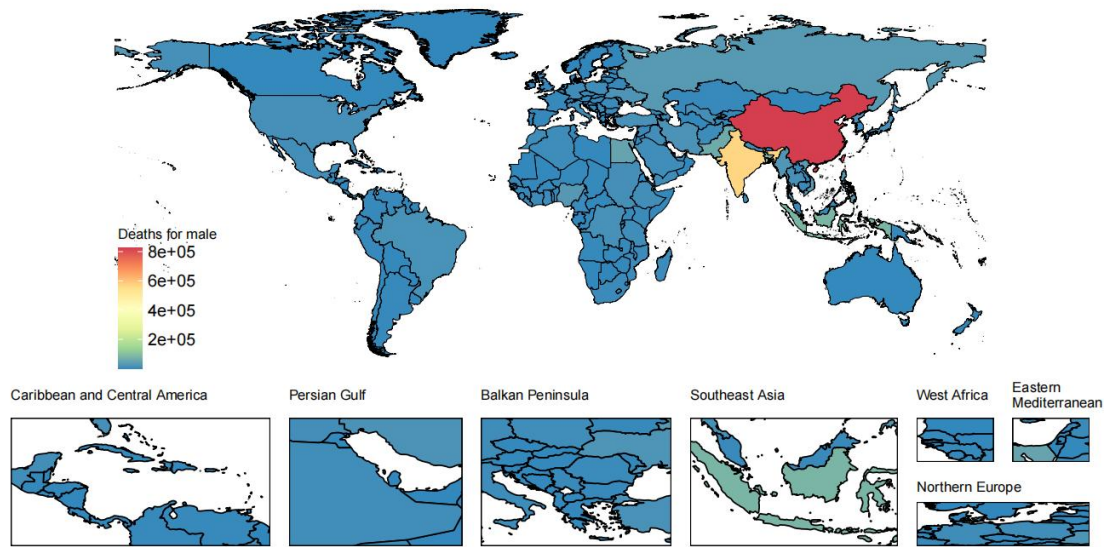

D

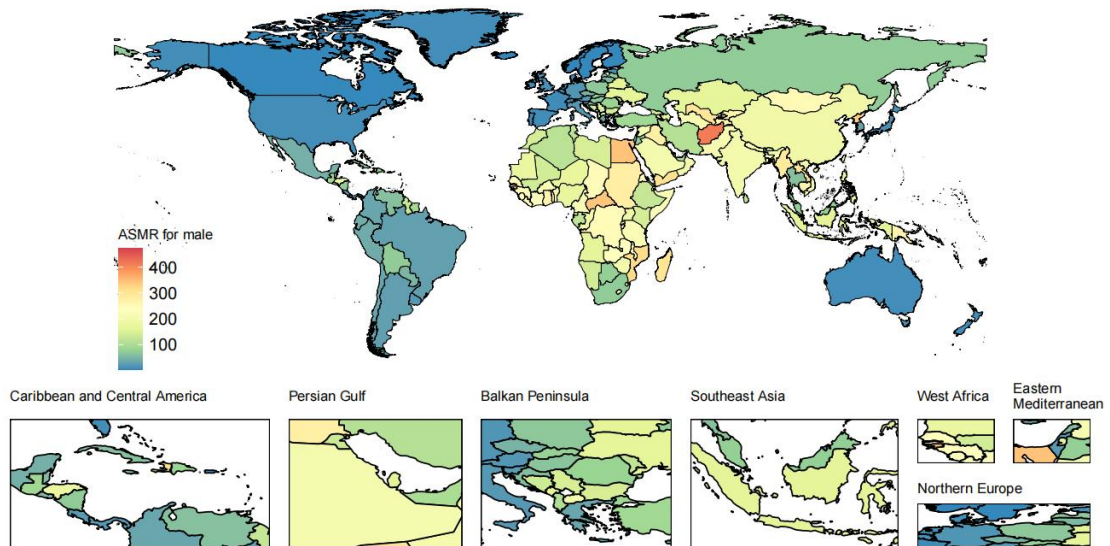

E

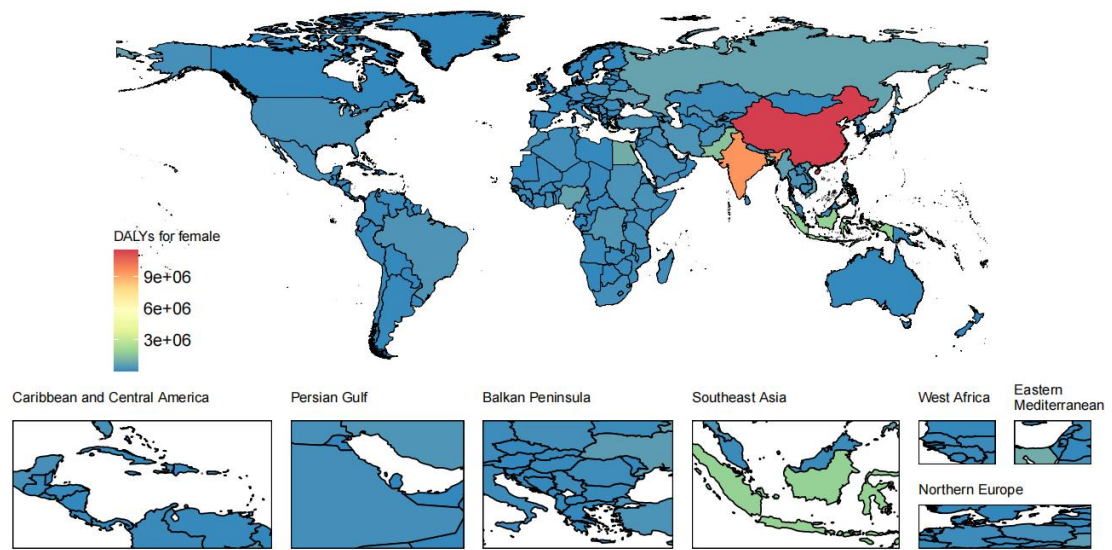

F

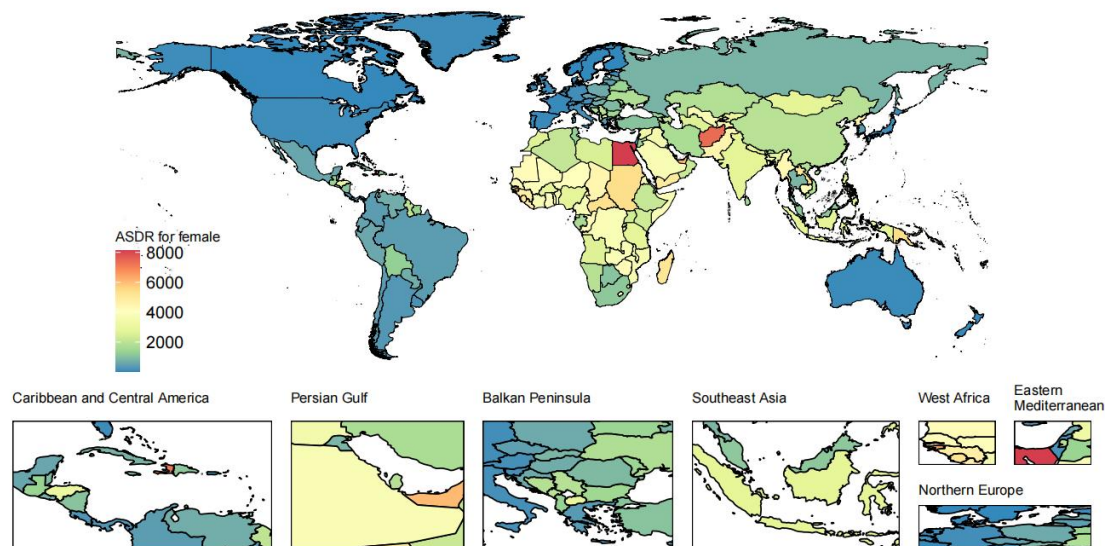

G

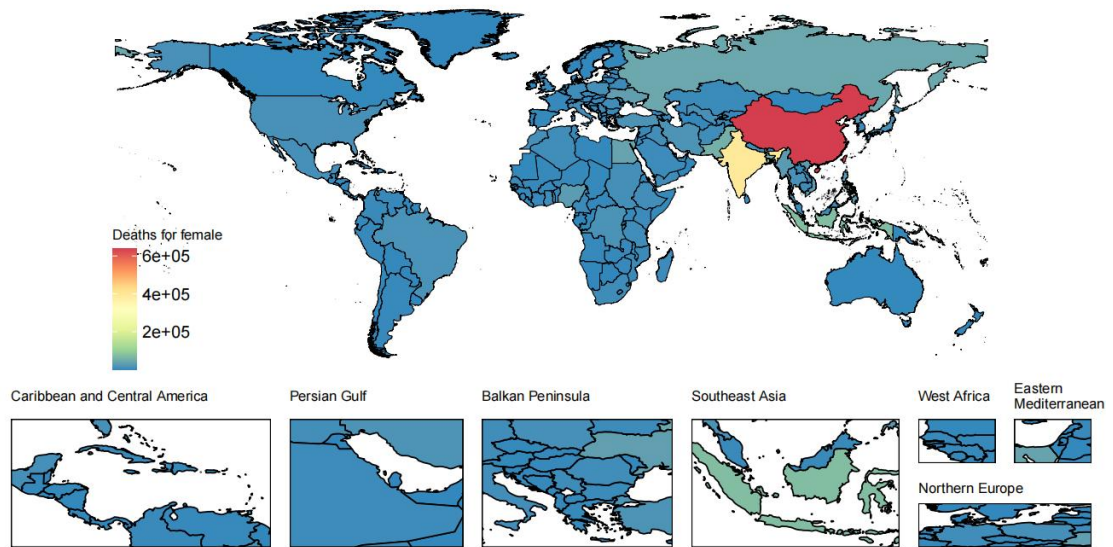

H

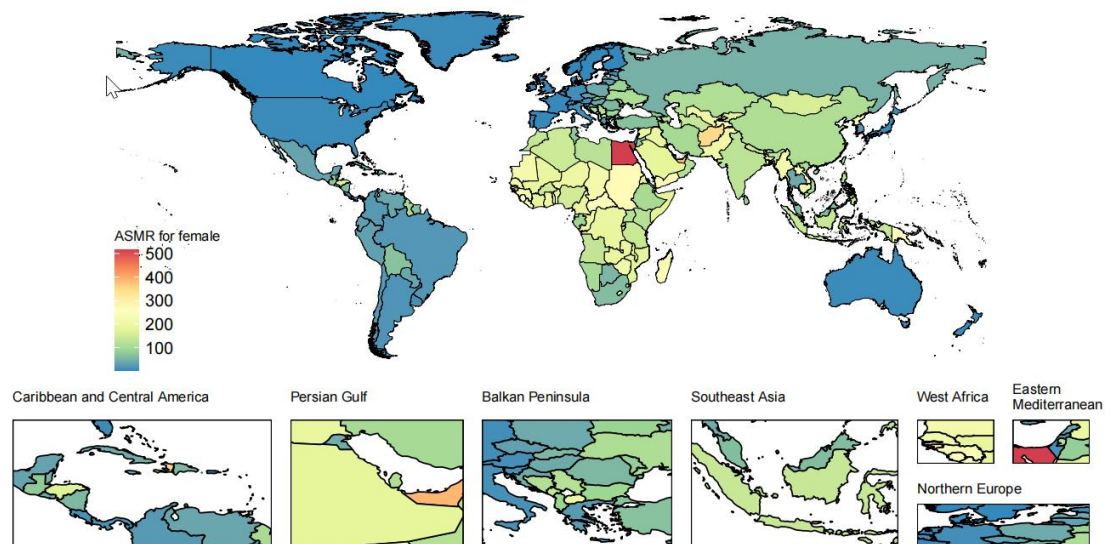

Supplement: Supplementary file 1 [file 2153-8174-26-4-27056-s1.zip › Supplementary figure 1.pdf]

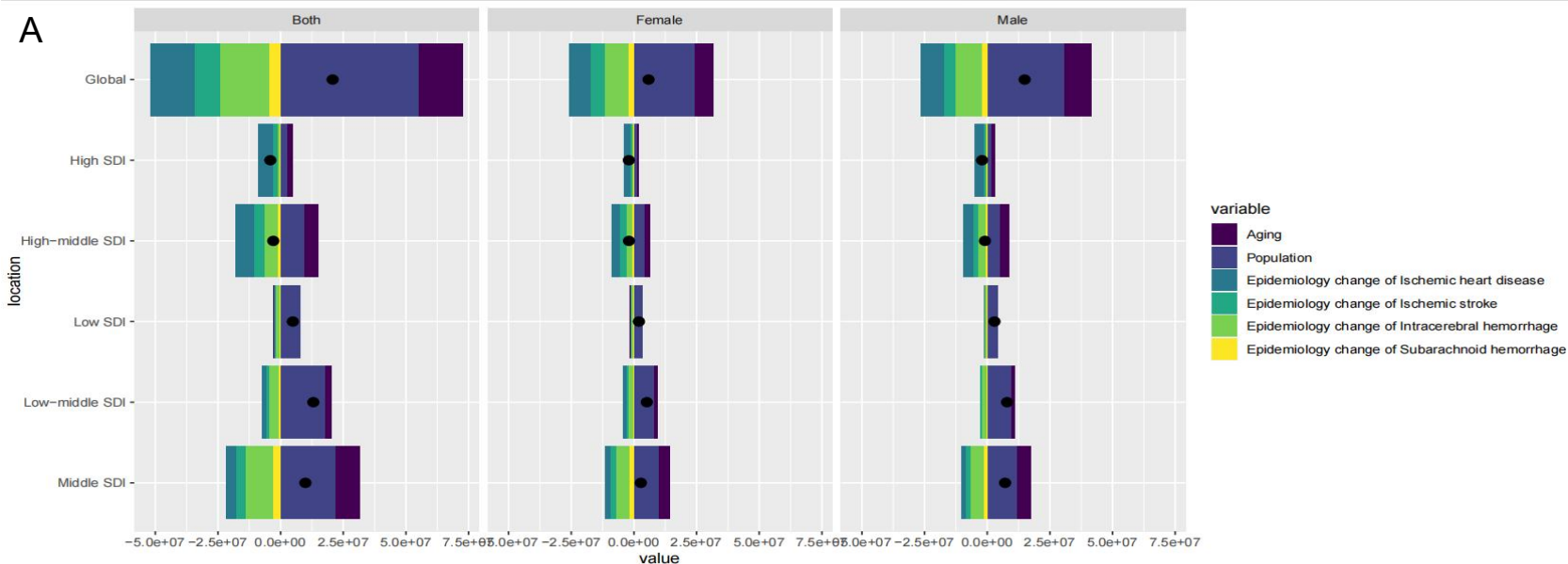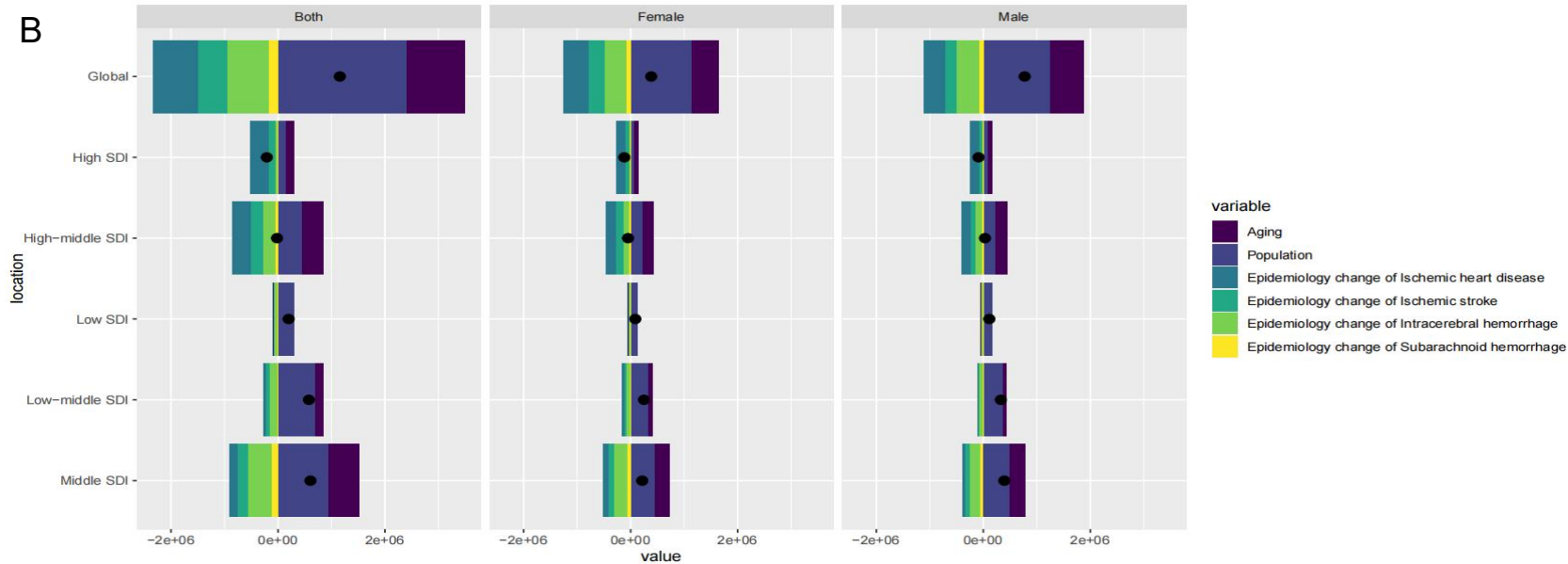

Supplement: Supplementary file 1 [file 2153-8174-26-4-27056-s1.zip › Supplementary Figure 4.pdf]

A

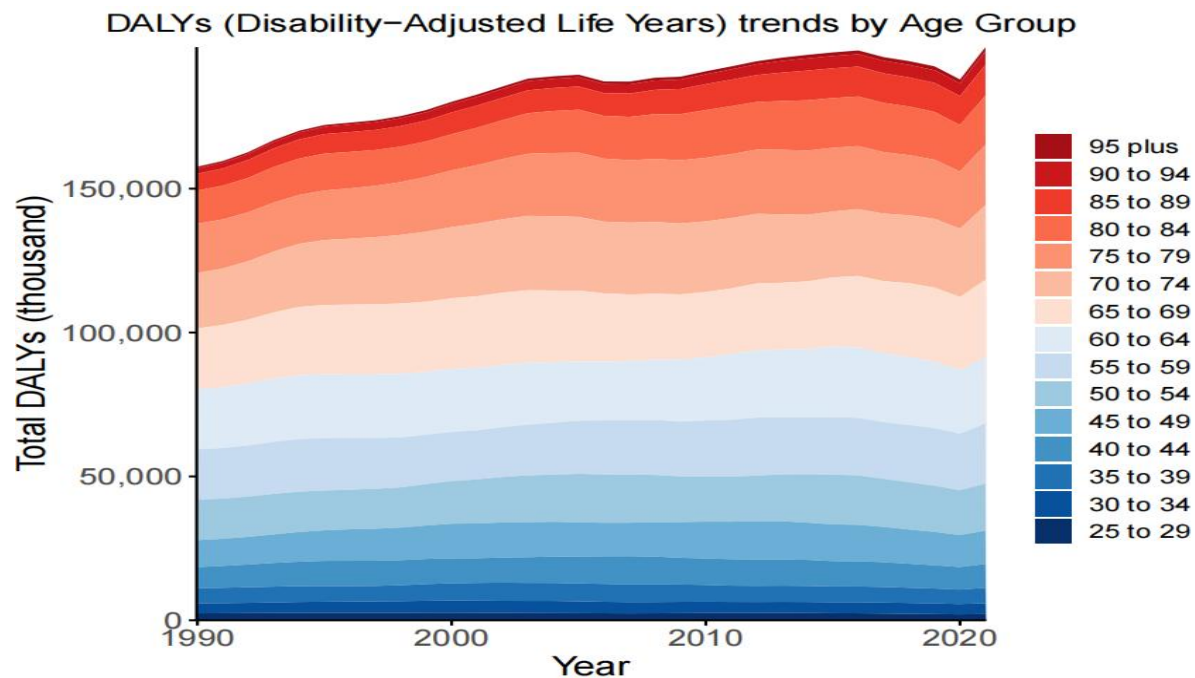

B

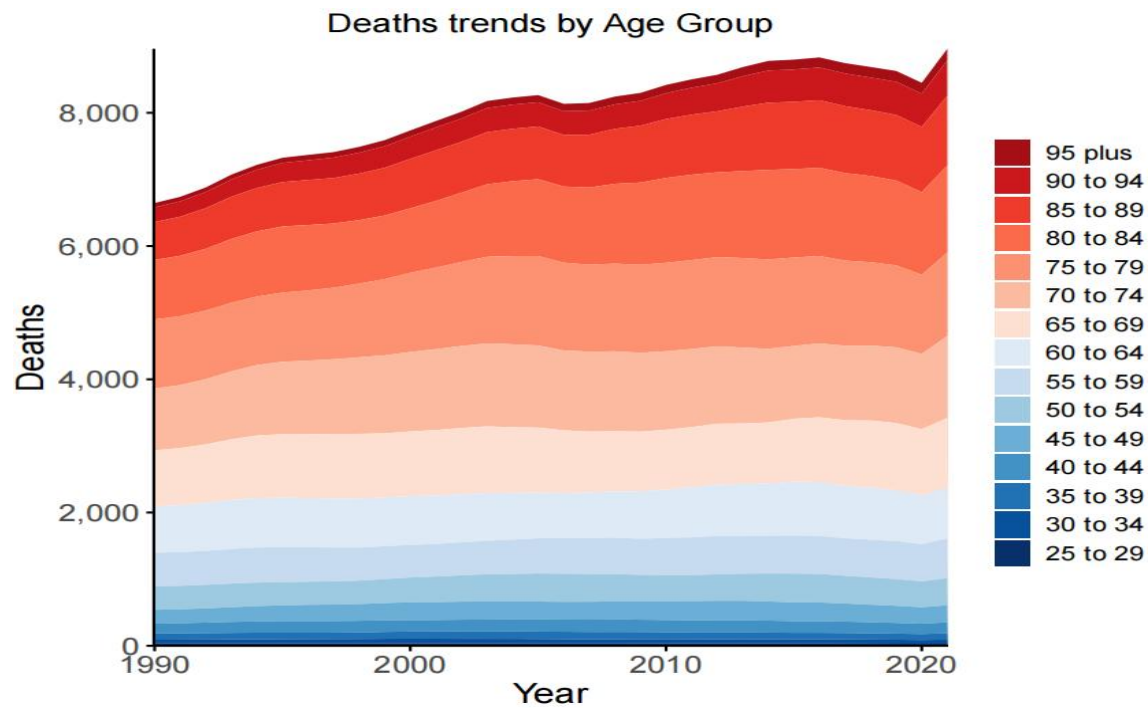

Supplement: Supplementary file 1 [file 2153-8174-26-4-27056-s1.zip › Supplementary figure 5.pdf]

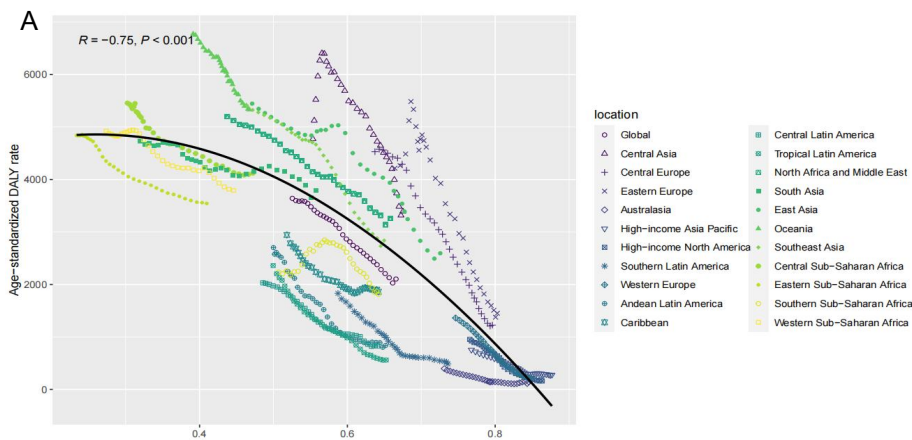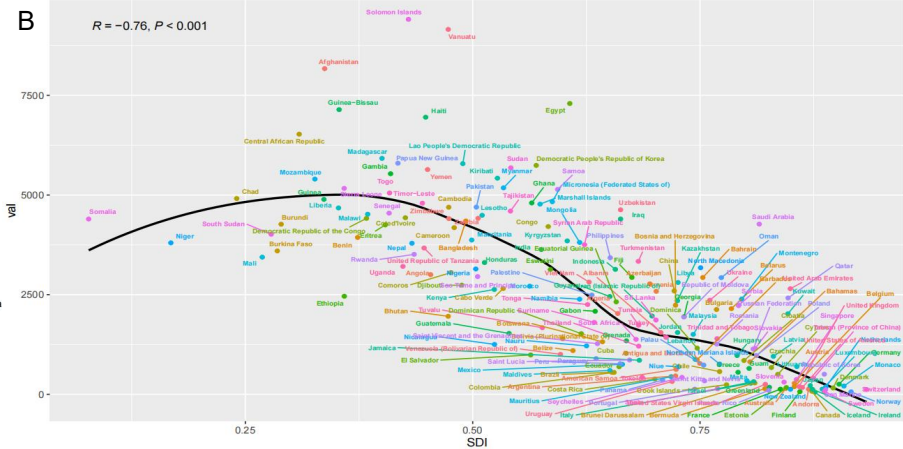

Supplement: Supplementary file 1 [file 2153-8174-26-4-27056-s1.zip › Supplementary figure2.pdf]

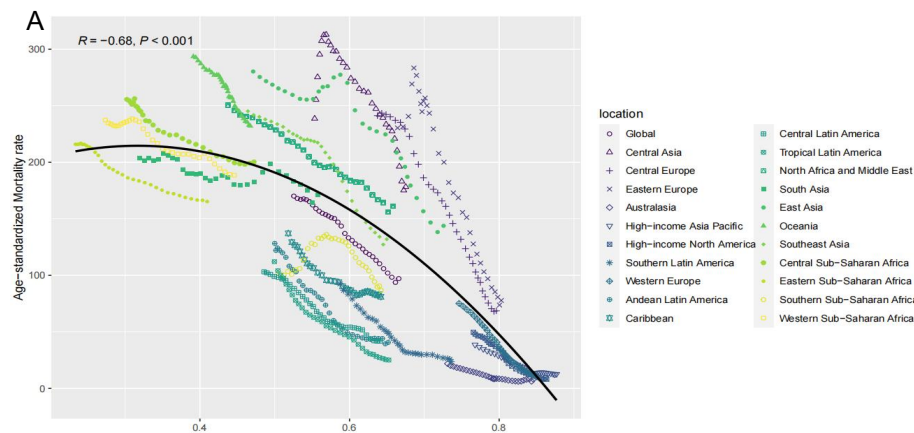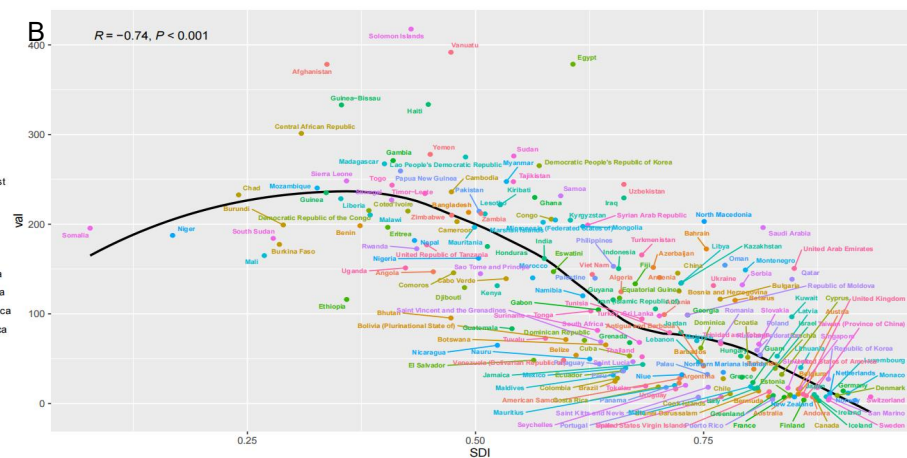

Supplement: Supplementary file 1 [file 2153-8174-26-4-27056-s1.zip › Supplementary figure3.pdf]

A

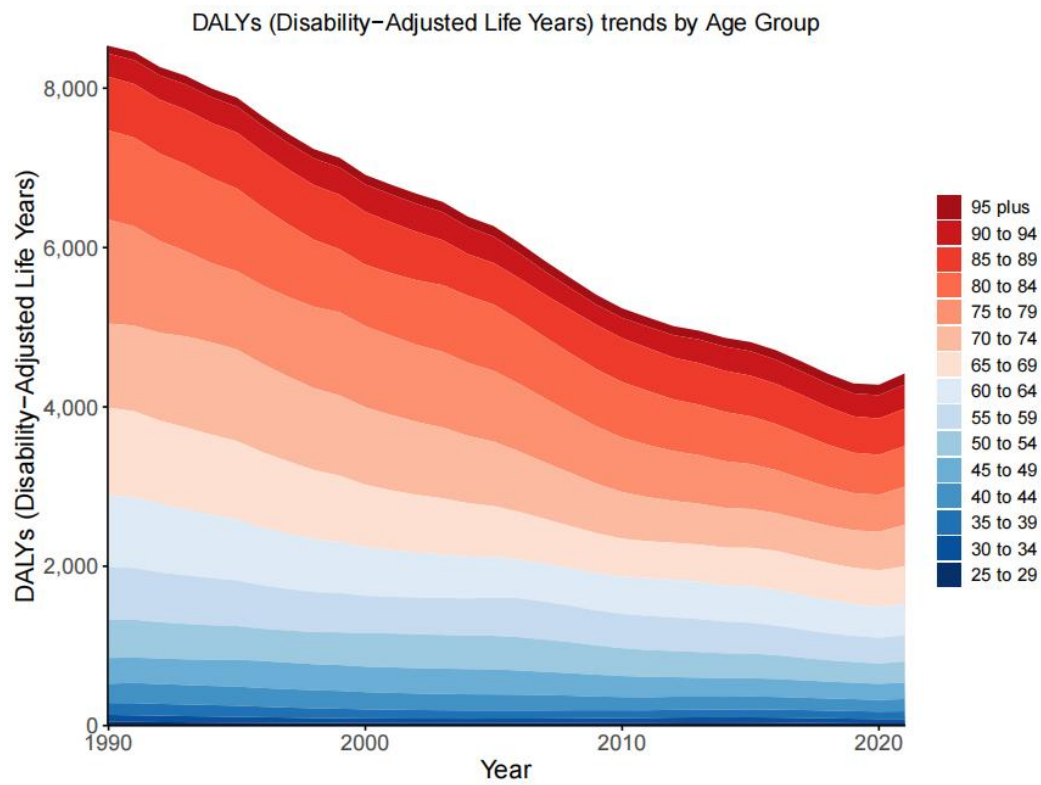

B

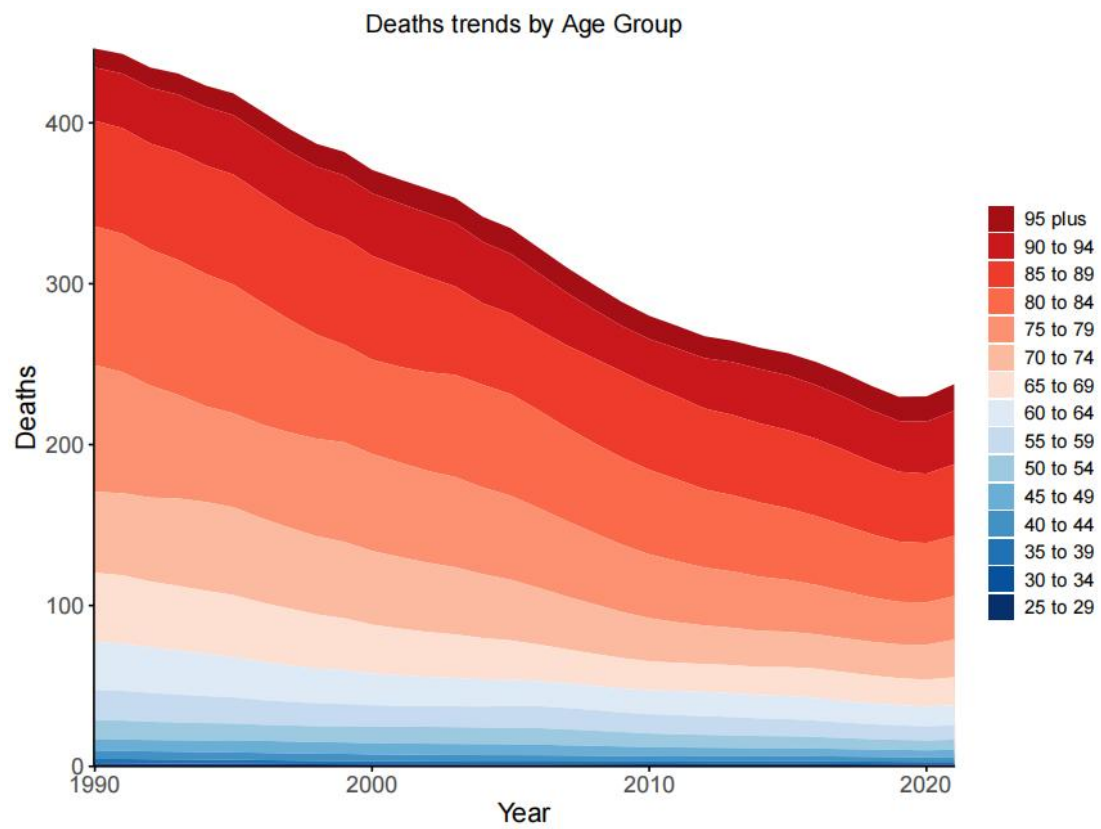

C

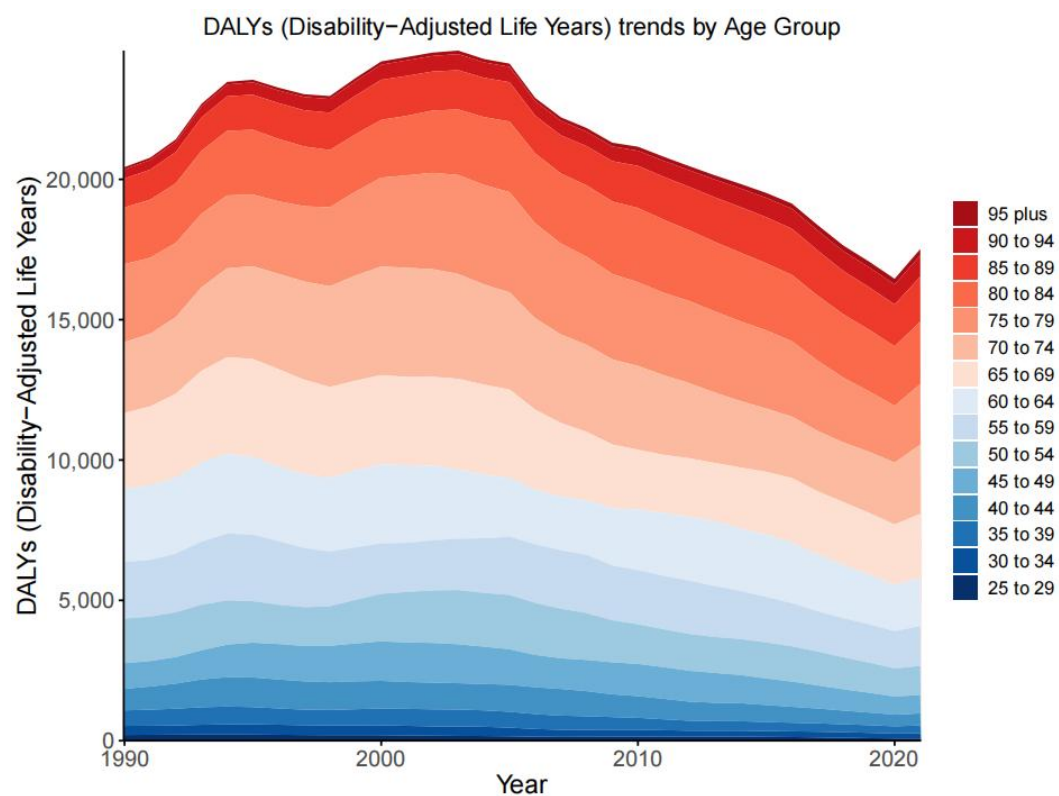

D

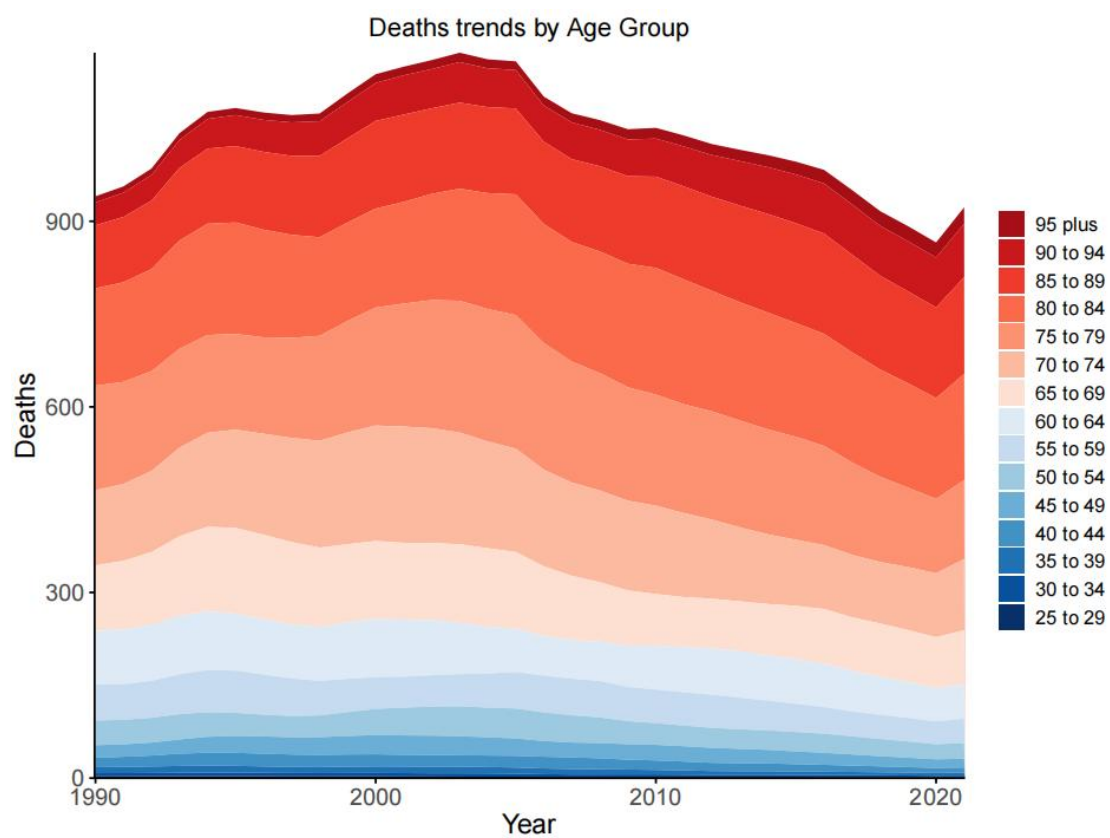

E

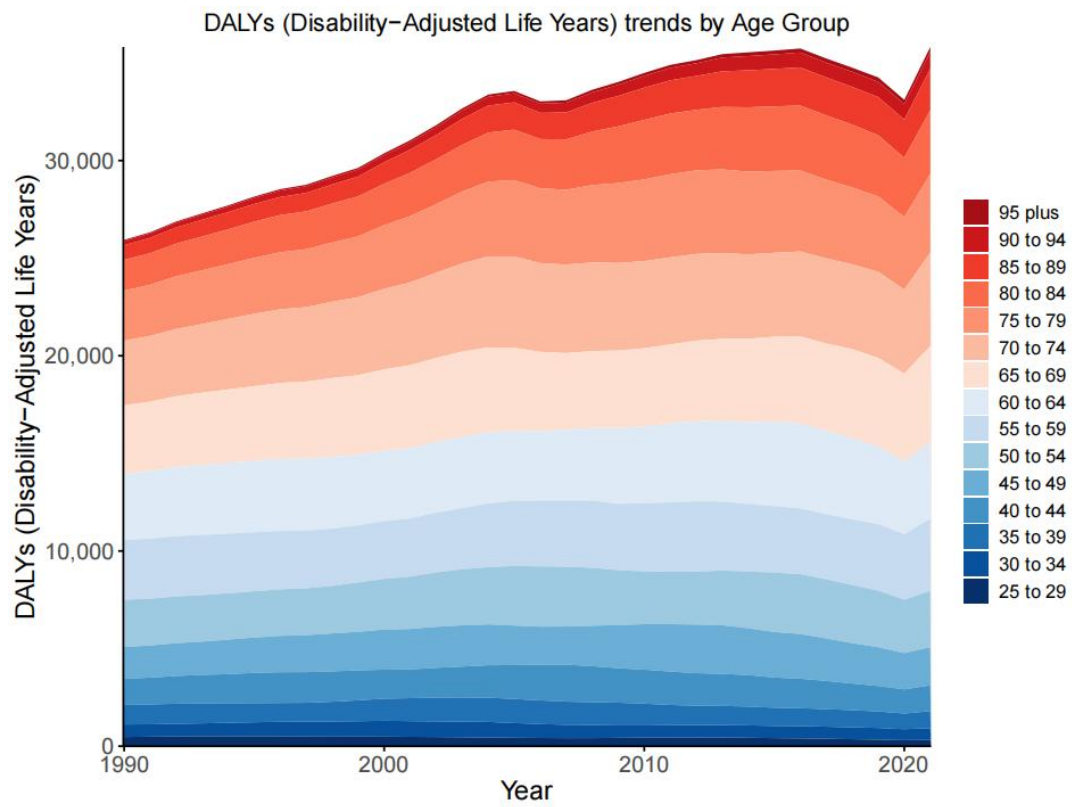

F

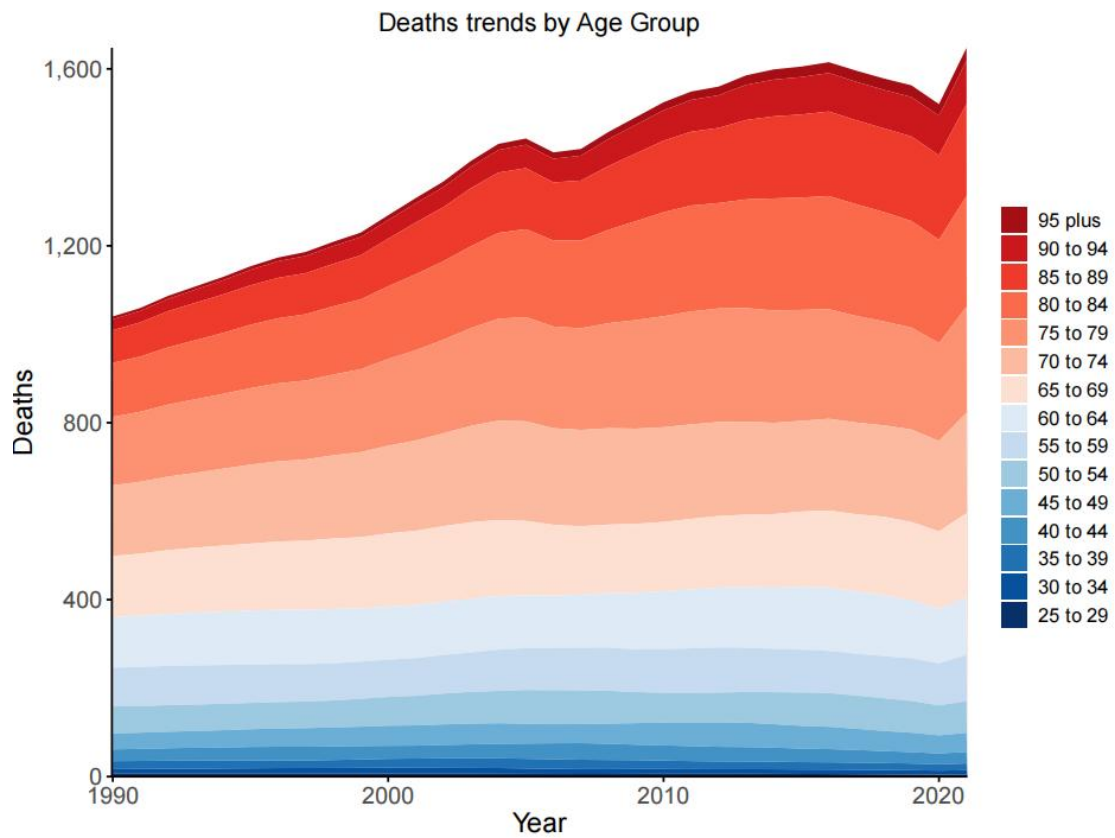

## G

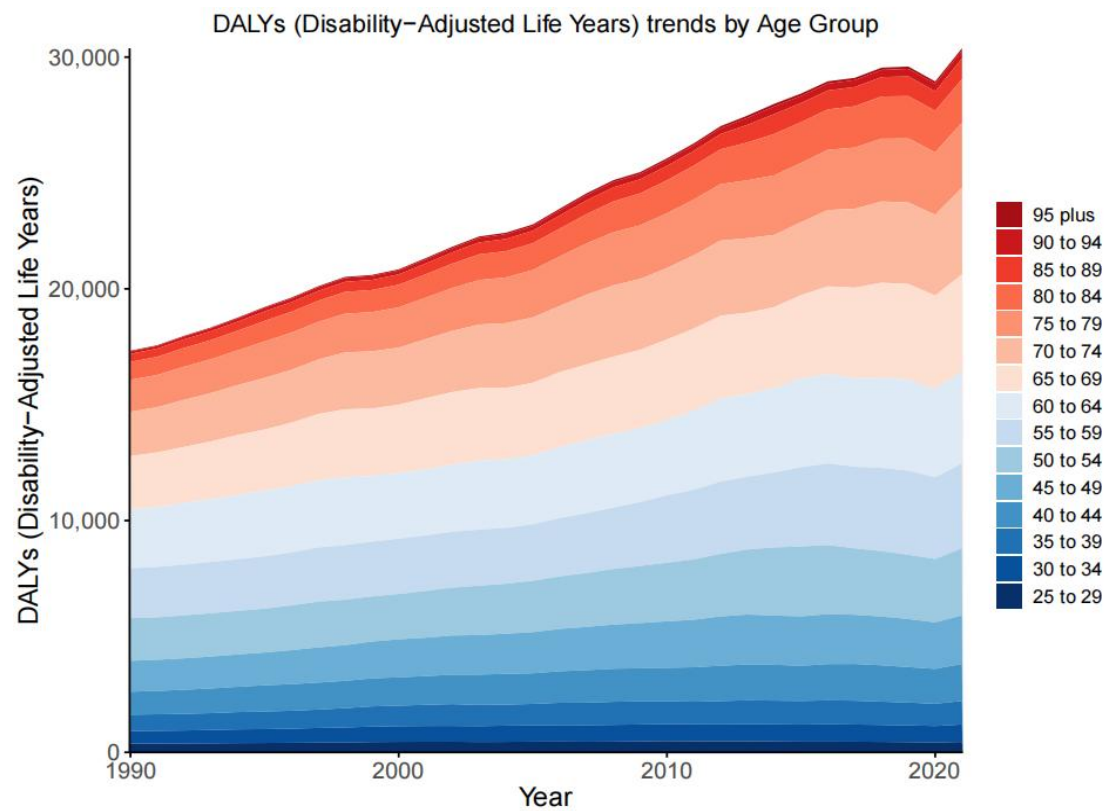

H

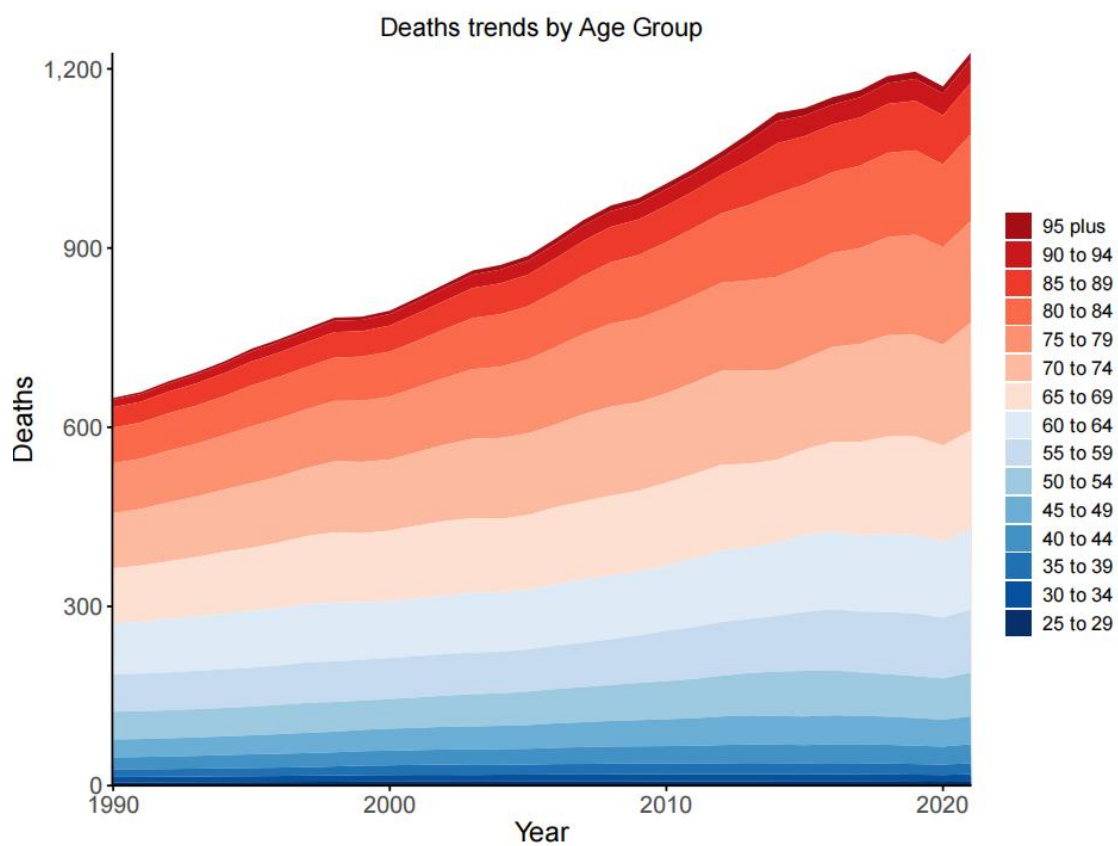

1

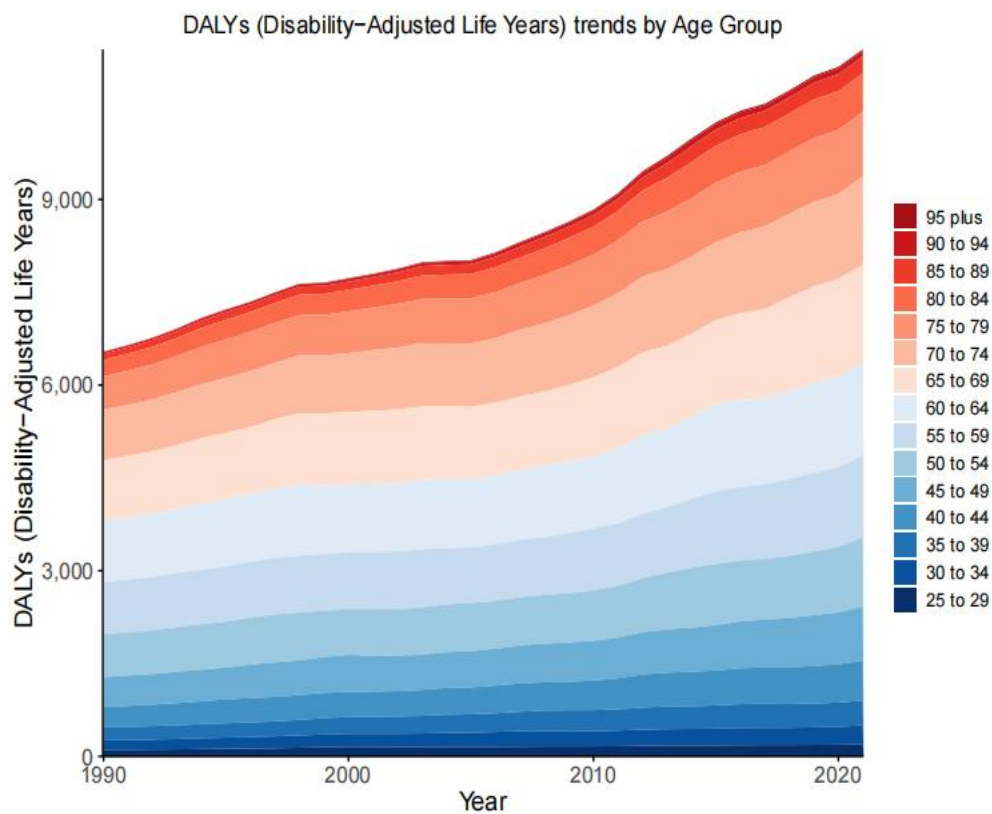

J

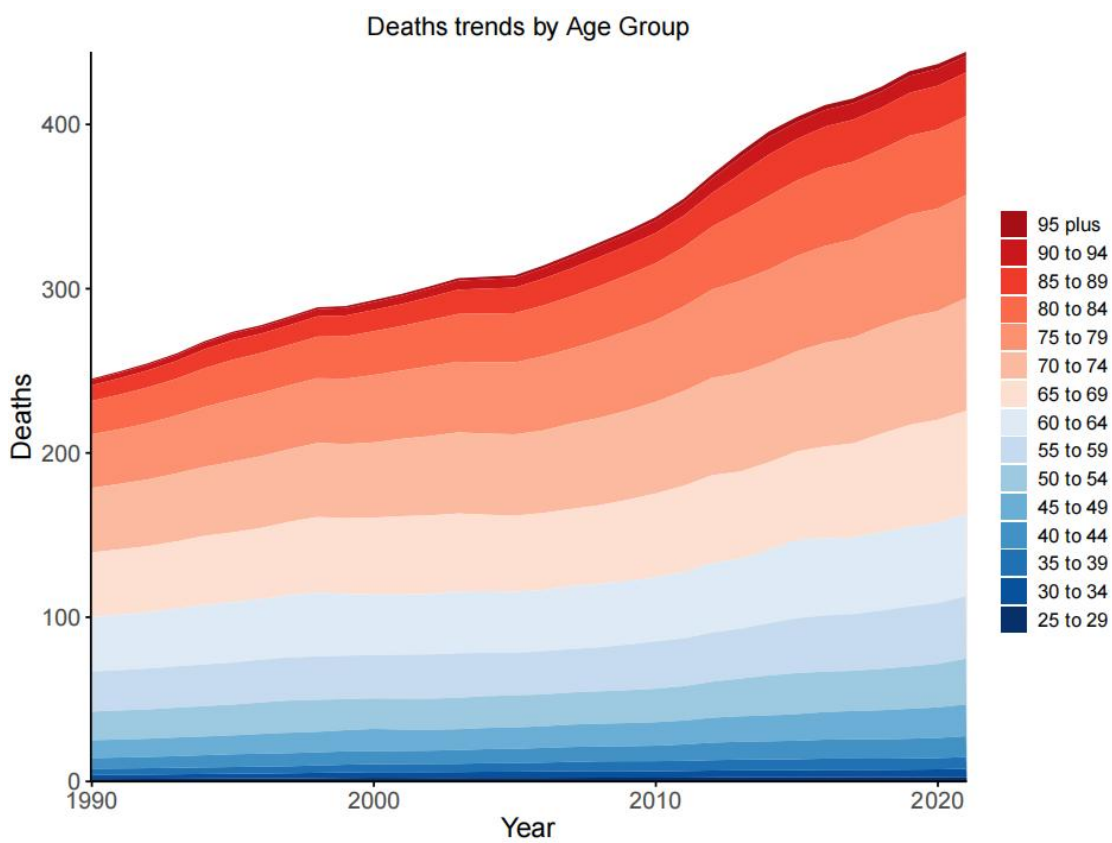

Supplement: Supplementary file 1 [file 2153-8174-26-4-27056-s1.zip › Supplementary figure6.pdf]
